# Anti-VEGF Monotherapy vs Anti-VEGF and Steroid Combination Therapy for Diabetic Macular Edema: A Meta-analysis

**DOI:** 10.1177/24741264241280597

**Published:** 2024-10-10

**Authors:** Justin Grad, Amin Hatamnejad, Rohan Dadak, Simrat Sodhi, Niveditha Pattathil, Netan Choudhry

**Affiliations:** 1 Michael DeGroote School of Medicine, McMaster University, Hamilton, ON, Canada; 2 University of Cambridge, Cambridge, UK; 3 Vitreous Retina Macula Specialists of Toronto, Etobicoke, ON, Canada; 4 Department of Ophthalmology and Vision Sciences, University of Toronto, Toronto, ON, Canada; 5 Cleveland Clinic Canada, Toronto, Ontario, Canada; 6 Retina Consultants of Texas, Blanton Eye Institute, Houston Methodist Hospital, Houston, TX, USA

**Keywords:** diabetic macular edema, anti-VEGF, steroid, combination therapy, monotherapy, treatment resistant, treatment naïve

## Abstract

**Introduction:** To compare the safety and efficacy of antivascular endothelial growth factor (anti-VEGF) monotherapy vs anti-VEGF and steroid combination therapy in treatment-naïve and treatment-resistant patients with diabetic macular edema (DME). **Methods**: A systematic literature search was conducted from January 2005 to December 2022. Sixteen randomized control trials (RCTs) published in English that reported the efficacy or safety of monotherapy and combination therapy in patients with DME were included. **Results:** The 16 RCTs included 1166 eyes. Monotherapy was associated with a significantly better best-corrected visual acuity (BCVA) at the final follow-up (weighted mean difference [WMD], −0.04 logMAR; 95% CI, −0.07 to −0.02; *P* = .002; *I*^2^ = 0%). No significant differences were observed in the change in BCVA between groups at the final observation. Monotherapy was associated with a significantly smaller change in retinal thickness at the final follow-up (WMD, 37.63 μm; 95% CI, 11.67-63.60; *P* = .005; *I*^2^ = 78%) and with a significantly lower risk for intraocular pressure–related adverse events (AEs) (risk ratio, 0.27; 95% CI, 0.15-0.46; *P* ≤ .001; *I*^2^ = 0%). The risk for cataract-related AEs was not significantly different between groups (*P* = .06). The results in treatment-naïve patients were similar. In treatment-resistant patients, the change in retinal thickness at the final follow-up was similar between groups (*P* = .14) but the risk for cataract-related AEs was significantly lower in the monotherapy group in 2 RCTs (risk ratio, 0.09; 95% CI, 0.01-0.66; *P* = .02; *I*^2^ = 0%). **Conclusions:** The changes in BCVA were similar despite combination therapy being associated with greater changes in retinal thickness. However, increased complications were seen with combination therapy. Most results in treatment-naïve patients and treatment-resistant patients were similar.

## Introduction

Diabetic macular edema (DME) is the accumulation of fluid in the retina extracellular space and is a serious complication of diabetic retinopathy that can occur at any stage of the disease.^1^ Chronic hyperglycemia in diabetes leads to significant vascular and inflammatory changes, notably pericyte loss and endothelial dysfunction, which compromise the blood–retinal barrier.^2,3^ Roughly 10% to 25% of diabetic patients will develop DME over a 10-year period, and it is the leading cause of blindness in this population.^4,5^

Laser photocoagulation was the first treatment for DME that substantially reduced the risk for vision loss and would remain the first-line treatment until the advent of antivascular endothelial growth factor (anti-VEGF).^6,7^ Subsequently, anti-VEGF agents have led to better functional and anatomic outcomes than laser treatment and they were the first treatment to achieve true reversal of vision loss.^8–11^

Despite anti-VEGF being considered the first-line treatment for DME, more than 40% of patients will still have persistent DME after adequate treatment.^12,13^ Steroids are an effective alternative option and have gained a substantial role in the management of patients who are resistant to anti-VEGF treatment.^14,15^ The use of steroids results in similar visual acuity (VA) outcomes and improved final retinal thickness compared with anti-VEGF agents; however, they have also been associated with a greater risk for increased intraocular pressure (IOP) and cataract formation.^16^

To our knowledge, to date no systematic reviews or meta-analyses have analyzed the efficacy or safety of anti-VEGF monotherapy vs concurrent anti-VEGF and steroid treatment, known as combination therapy, in treatment-resistant or treatment-naïve populations. A well-established definition for patients who are treatment resistant is lacking. Therefore, any study that labels patients as treatment resistant is doing so based on the authors’ own criteria. In contrast, a treatment-naïve patient specifically refers to a patient with no previous treatment history.

The purpose of this meta-analysis was to assess the safety and efficacy of the 2 treatment modalities and to determine whether a patient’s previous treatment status is an important factor in his or her outcomes. This study aimed to update recommendations and guidelines for the use of anti-VEGF and steroid combination therapy vs anti-VEGF monotherapy for the treatment of DME.

## Methods

### Search Strategy and Eligibility Criteria

A systematic literature search was conducted from January 2005 to December 2022 using Ovid MEDLINE, Embase, and Cochrane CENTRAL (Supplemental Table 1). RCTs comparing anti-VEGF monotherapy with anti-VEGF and steroid combination therapy for patients with DME were included if they reported efficacy or safety outcomes. This study adhered to the Declaration of Helsinki and did not require ethics approval or informed consent because it was a meta-analysis. The meta-analysis was registered in the International Prospective Register of Systematic Review (PROSPERO) database (ID: CRD42023382418)

### Study Selection and Data Collection

Screening was done in 2 stages. First, the title and abstract were screened; this was followed by full-text screening. Screening was completed using the Covidence online application (Veritas Health Innovation). Screening and data collection were completed by at least 2 independent reviewers. Any conflicts that arose were resolved in consultation with another reviewer. All data were compiled using Excel software (Microsoft Corp).

The following baseline characteristic data were extracted from studies when available: publication year, country of study, number of eyes, number of patients, proportion of men, age, study design, treatment-resistance type, number of phakic eyes, initial best-corrected VA (BCVA), initial retinal thickness, drug dose, and treatment regimen.

The primary outcome was the final change in BCVA. Secondary outcomes were the final BCVA, change in retinal thickness, final retinal thickness, and adverse events (AEs). The BCVA and retinal thickness outcomes were collected at different timepoints as available. The change in BCVA was collected in logMAR notation and Early Treatment Diabetic Retinopathy Study (ETDRS) letters. The BCVA at different timepoints was collected in logMAR notation. When the BCVA was published in ETDRS letters, it was converted to logMAR notation as outlined by Khoshnood et al.^17^

Included studies were classified as treatment resistant if the study’s methods mentioned that the patients were refractive or resistant to previous treatment for DME. All remaining studies were classified as treatment naïve. For study arms to be considered combination therapy, patients must have received overlapping anti-VEGF and steroid therapy; however, both therapies were not required to be administered at the same visit.

### Risk for Bias Assessment and Certainty of Evidence Assessment

Risk for bias for the included studies was assessed using the Cochrane Risk of Bias Tool 2.^18^ Certainty of evidence was evaluated using the Grading of Recommendations, Assessment, Development, and Evaluation (GRADE) framework.^19^ Both tools were completed independently by 2 reviewers, with discrepancies resolved by a third reviewer.

### Statistical Analysis

The inverse variance approach was used for reporting continuous data as weighted mean differences (WMDs) and 95% CIs. The weighted variable considered was the number of eyes in the comparison. The Mantel-Haenszel method was used for dichotomous data outcomes, with the data being presented as risk ratios and 95% CIs. Statistical heterogeneity was assessed by calculating the *I*^2^ statistic, with any value greater than 75% considered significant. Statistical significance was set at *P* < .05. A random-effects model was used for all comparisons in the meta-analysis. RevMan bespoke software (version 5.4, Nordic Cochrane Centre) was used for the data analysis.

## Results

**Figure 1.**
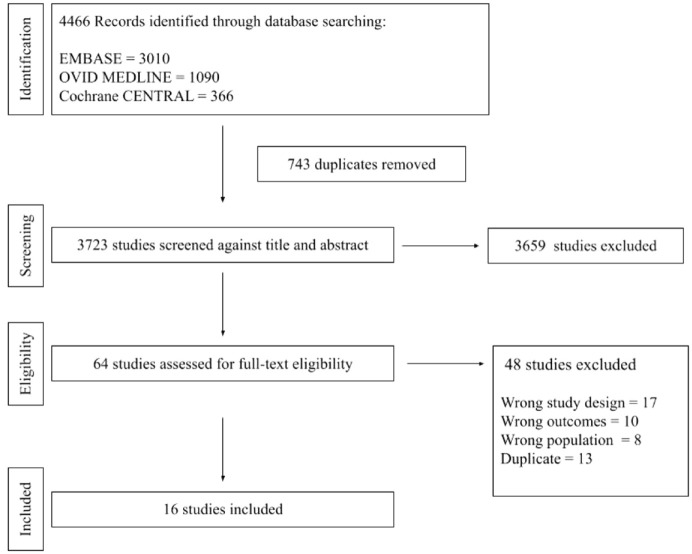
Flowchart summarizing the study screening and selection process.

The systematic search resulted in 4466 studies with title and abstract screening and 64 with full-text screening. Ultimately, 16 RCTs comprising 1166 eyes were included (Figure 1). Anti-VEGF dosing regimens of study arms included single dose,^20–22^ single dose with retreatment if indicated,^23^ fixed interval,^24,25^ and pro re nata (PRN).^26–35^ Steroid dosing regimens of study arms included a single dose,^20–22,24–26,29,35^ a single dose with retreatment if indicated,^23,28,33^ 2 doses at a fixed interval,^31^ and PRN.^27,30,32,34^ Table 1 shows the baseline data of the included studies.^20-35^

**Table 1.** Baseline Characteristics of Included Studies.

			Mean ± SD											
First Author/Year Published: Country	Eyes (n)	Men (%)	Age (Y)	Initial LogMAR BCVA	Initial Retinal Thickness (µm)	Anti-VEGF Agent	Steroid	Treatment Status	Refractory Defintion	Anti-VEGF Treatment Regimen	Mean Anti-VEGF Injections (n) ± SD	Steroid Treatment Regimen	Mean Steroid Injections (n) ± SD	FU (Mo)
Faghihi^23^/2008: Iran														
Treatment arm														
Monotherapy	42	54.80	59 ± 6	0.70 ± 0.31	N/A	BEV	TA	Naive	N/A	Single dose w/ 1 retreatment if indicated	N/A	N/A	N/A	4
Combination therapy	41	46.30	56 ± 7	0.77 ± 0.33	N/A	BEV	TA	Naïve	N/A	Single dose w/ 1 retreatment if indicated	N/A	Single dose w/ 1 retreatment if indicated beyond 16 wk	N/A	4
Wang^22^/2011: China														
Treatment arm														
Monotherapy	21	57.10	54.19 ± 9.90	0.7318 ± 0.36	525.76 ± 184.10	BEV	TA	Naïve	N/A	Single dose	1	N/A	N/A	3
Combination therapy	19	52.60	52.11 ± 12.54	0.7606 ± 0.32	554.50 ± 169.05	BEV	TA	Naïve	N/A	Single dose	1	Single dose	1	3
Lim^29^/2012: Korea														
Treatment arm														
Monotherapy	38	50.00	61.4 ± 6.7	0.62 ± 0.23	447 ± 110	BEV	TA	Naïve	N/A	PRN	1.04 ± 0.98	N/A	N/A	12
Combination therapy	36	44.40	58.4 ± 5.9	0.64 ± 0.25	458 ± 92	BEV	TA	Naïve	N/A	PRN	1.28 ± 1.05	Single dose	N/A	12
Soheilian^32^/2012: Iran														
Treatment arm														
Monotherapy	50	46	60.5 ± 5.9	0.71 ± 0.28	341 ± 148	BEV	TA	Naïve	N/A	PRN	3.1 ± 1.6	N/A	3.1 ± 1.6	24
Combination therapy	50	56	62.3 ± 6.8	0.73 ± 0.28	359 ± 137	BEV	TA	Naïve	N/A	PRN	2.6 ± 1.5	PRN, assessed every 12 wk	2.6 ± 1.5	24
Marey^20^/2011: Egypt														
Treatment arm														
Monotherapy	30	53.30	57.60 ± 7.30	0.22 ± 0.12	445.06 ± 123.87	BEV	TA	Naïve	N/A	Single dose	1	N/A	N/A	3
Combination therapy	30	63.30	57.66 ± 7.44	0.19 ± 0.13	477.70 ± 153.38	BEV	TA	Naïve	N/A	Single dose	1	Single dose	1	3
Neto^27^/2017: Brazil														
Treatment arm														
Monotherapy	39	59.00	N/A	N/A	412.6	BEV	TA	Naïve	N/A	PRN	3.2	N/A	N/A	6
Combination therapy	34	52.90	N/A	N/A	407.33	BEV	TA	Naïve	N/A	PRN	2.4	PRN, assessed monthly	2.4	6
Riazi-Esfahani^30^/2018: Iran			62 ± 8.6^a^											
Treatment arm														
Monotherapy	46	45.60		0.35 ± 0.25	N/A	BEV	TA	Naïve	N/A	PRN	1.71	N/A	N/A	6
Combination therapy	46	45.60		0.38 ± 0.3	N/A	BEV	TA	Naïve	N/A	PRN	1.56	PRN, assessed every 6 wk	1.56	6
Kaya^28^/2021: Turkey														
Treatment arm														
Monotherapy	34	35.30	66.2 ± 8.8	0.66 ± 0.28	N/A	RAN	DEX	Naïve	N/A	PRN	10.4	N/A	N/A	12
Combination therapy	34	35.30	64.6 ± 10.5	0.74 ± 0.46	N/A	RAN	DEX	Naïve	N/A	PRN	8.3	Single dose with 1 retreatment if indicated at 6 mo	1.7	12
Sever^26^/2019: Turkey			56.2 ± 11.5^a^											
Treatment arm														
Monotherapy	44	N/A		0.64 ± 0.07	372.6 ± 33.0	RAN	DEX	Resistant	Resistant to 3 RAN loading doses	PRN	7.1 ± 0.7	N/A	N/A	12
Combination therapy	40	N/A		0.68 ± 0.06	376.6 ± 34.9	RAN	DEX	Resistant	Resistant to 3 RAN loading doses	PRN	7.1 ± 0.8	Single dose	1	12
Ozkan^21^/2019: Turkey														
Treatment arm														
Monotherapy	21	N/A	65.4 ± 8.9	0.59 ± 0.3	494.7 ± 114.4	BEV	TA	Naïve	N/A	Single dose	1	N/A	N/A	6
Combination therapy	21	N/A	65.4 ± 8.9	0.59 ± 0.17	546.8 ± 165.6	BEV	TA	Naïve	N/A	Single dose	1	Single dose	1	6
Barakat^31^/2021: USA														
Treatment arm														
Monotherapy	35	68.60	59.2 ± 12.89	0.54 ± 0.04	506.6 ± 24.55	AFL	TA	Naïve	N/A	PRN	4.6	N/A	N/A	6
Combination therapy	36	72.20	59.8 ± 10.16	0.54 ± 0.04	496.1 ± 25.82	AFL	TA	Naive	N/A	PRN	2.6	Fixed (baseline & 12 wk)	2	6
Synek^24^/2011: Czech Republic			59.7 ± 8.3^a^											
Treatment arm														
Monotherapy	30	N/A		N/A	N/A	BEV	TA	Resistant	Eyes w/ CSME unresponsive to previous ME laser photocoagulation	Fixed	3	N/A	N/A	24
Combination therapy	30	N/A		N/A	N/A	BEV	TA	Resistant	Eyes w/ CSME unresponsive to previous ME laser photocoagulation	Fixed	3	Single dose	1	24
Maturi^33^/2018: USA														
Treatment arm														
Monotherapy	64	43.80	66	N/A	N/A	RAN	DEX	Resistant	Persistent DME after 3 anti-VEGF injections in 12-wk run-in phase	PRN	5.7 ± 0.7	N/A	N/A	6
Combination therapy	65	52.30	64	N/A	N/A	RAN	DEX	Resistant	Persistent DME after 3 anti-VEGF injections in 12-wk run-in phase	PRN	5.6 ± 0.7	Single dose with 1 retreatment if indicated between 12 wk & 20 wk	1.98	6
Maturi^34^/2015: USA														
Treatment arm														
Monotherapy	21	N/A	N/A	0.38 ± 0.302	344 ± 39	BEV	DEX	Resistant	Incomplete response to multiple anti-VEGF injections; CST 250 µm	PRN	9	N/A	N/A	12
Combination therapy	19	N/A	N/A	0.38 ± 0.268	423 ± 14.2	BEV	DEX	Resistant	Incomplete response to multiple anti-VEGF injections; CST 250 µm	PRN	6	PRN, assessed every 4 mo	2.1	12
Shoeibi^25^/2013: Iran														
Treatment arm														
Monotherapy	41	17.10	60.4 ± 9.3	0.88 ± 0.32	414.6 ± 62.1	BEV	TA	Resistant	ME not responsive to previous laser treatment	Fixed	3	N/A	N/A	13
Combination therapy	37	18.90	59.1 ± 8.1	0.92 ± 0.30	417.7 ± 139.4	BEV	TA	Resistant	ME not responsive to previous laser treatment	Fixed	3	12	1	13
Eriş^35^/2019: Turkey														
Treatment arm														
Monotherapy	34	N/A	63.94 ± 8.47	0.47 ± 0.6	438.2 ± 90.99	RAN	TA	Resistant	CMT >350 μm w/ <3 lines of visual gain after at least 6 anti-VEGF injections	PRN	4.61 ± 0.88	N/A	N/A	6
Combination therapy	38	N/A	58.94 ± 7.79	0.51 ± 0.667	494 ± 118.32	RAN	TA	Resistant	CMT >350 μm w/ <3 lines of visual gain after at least 6 anti-VEGF injections	PRN	3.15 ± 0.88	12	1	6

Abbreviations: AFL, aflibercept; Anti-VEGF, antivascular endothelial growth factor; BCVA, best-corrected visual acuity; BEV, bevacizumab; CMT central macular thickness; CSME, clinically signficant macular edema; CST, central subfield thickness; DEX, dexamethasone; DME, diabetic macular edema; FU, follow-up; ME, macular edema; N/A, not available; PRN, pro re nata; RAN, ranibizumab; TA, triamcinolone acetonide.

a Entire cohort.

### Risk for Bias and GRADE Assessment

Supplemental Table 2 shows the outcomes of the Cochrane Risk of Bias Tool 2 evaluation. Some concerns about the risk for bias were found for the randomization process in 6 studies (37%), for deviation from intended interventions in 1 study (6%), for missing outcome data in 1 study (6%), for measurement of the outcomes in 5 studies (31%), and for selection of reported results in 2 studies (12%). All remaining domains were evaluated as having a low risk for bias.

Supplemental Table 3 shows the outcomes for certainty of evidence assessed using the GRADE tool. In the main analysis, 56% of outcomes compared were assigned a low certainty of evidence, 40% were assigned a medium certainty of evidence, and 4% were assigned a high certainty of evidence.

### Main Analysis: Anti-VEGF vs Combined Anti-VEGF and Steroid

#### Best Corrected Visual Acuity

**Figure 2.**
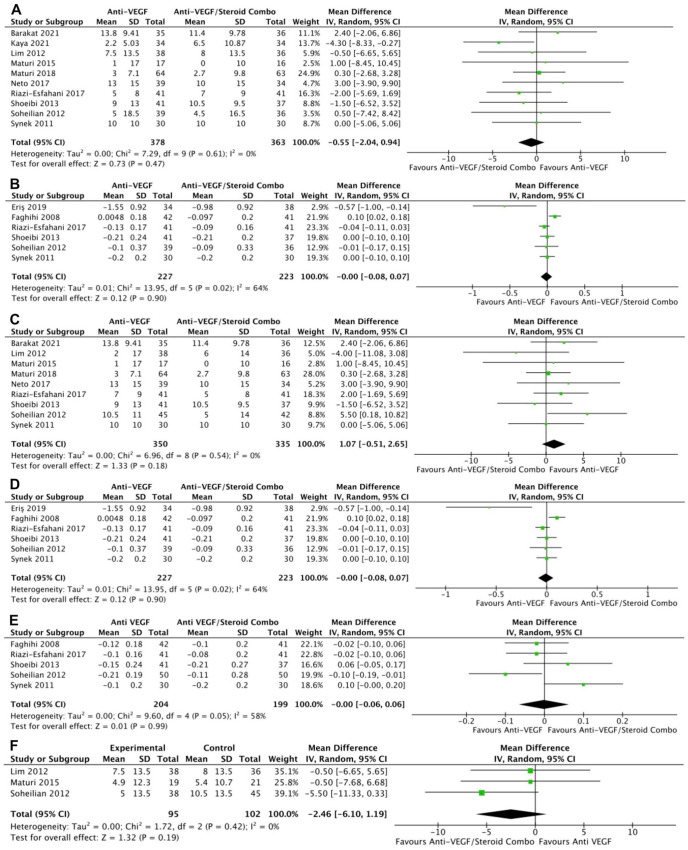
Change in BCVA over time. At last study observation (A) in letters and (B) in logMAR. At 4 to 6 months (C) in letters and (D) in logMAR. (E) At 0 to 3 months. (F) At 10 to 12 months. Abbreviations: Anti-VEGF, antivascular endothelial growth factor; BCVA, best-corrected visual acuity; IV, inverse variance.

**Figure 3.**
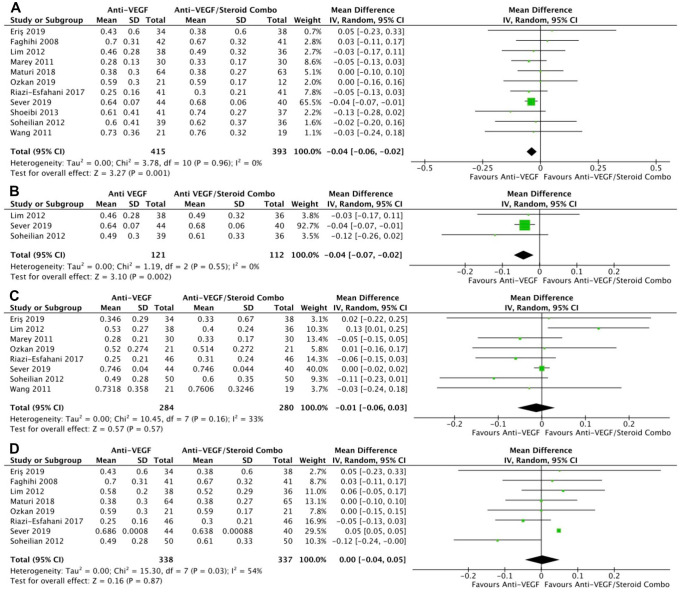
BCVA over time. (A) At last study observation. (B) At 10 to 12 months. (C) At 0 to 3 months. (D) At 4 to 6 months. Abbreviations: Anti-VEGF, antivascular endothelial growth factor; BCVA, best-corrected visual acuity; IV, inverse variance.

Monotherapy and combination therapy resulted in similar changes in BCVA at the final follow-up using ETDRS letters in 10 RCTs (*P* = .47) (Figure 2A) and using logMAR notation in 6 RCTs (*P* = .90) (Figure 2B). There were no significant differences between monotherapy and combination therapy in the change in BCVA at 4 to 6 months using ETDRS letters (*P* = .18) (Figure 2C) or logMAR notation (*P* = .90) (Figure 2D). In addition, the change in BCVA was similar for monotherapy therapy and combination therapy at 0 to 3 months (*P* = .99) (Figure 2E) and 10 to 12 months (*P* = .19) (Figure 2F). Monotherapy was associated with a significantly better BCVA than combination therapy at the final follow-up in 11 RCTs (WMD, −0.04 logMAR; 95% CI, −0.06 to −0.02; *P* = .001; *I*^2^ = 0%) (Figure 3A). Similarly, those receiving monotherapy had a significantly better BCVA at 10 to 12 months in 3 RCTs (WMD, −0.04 logMAR; 95% CI, −0.07 to −0.02; *P* = .002; *I*^2^ = 0%) (Figure 3B). In contrast, the BCVA was similar between groups at 0 to 3 months (*P* = .57) (Figure 3C) and at 4 to 6 months (*P* = .87) (Figure 3D).

##### Retinal Thickness

**Figure 4.**
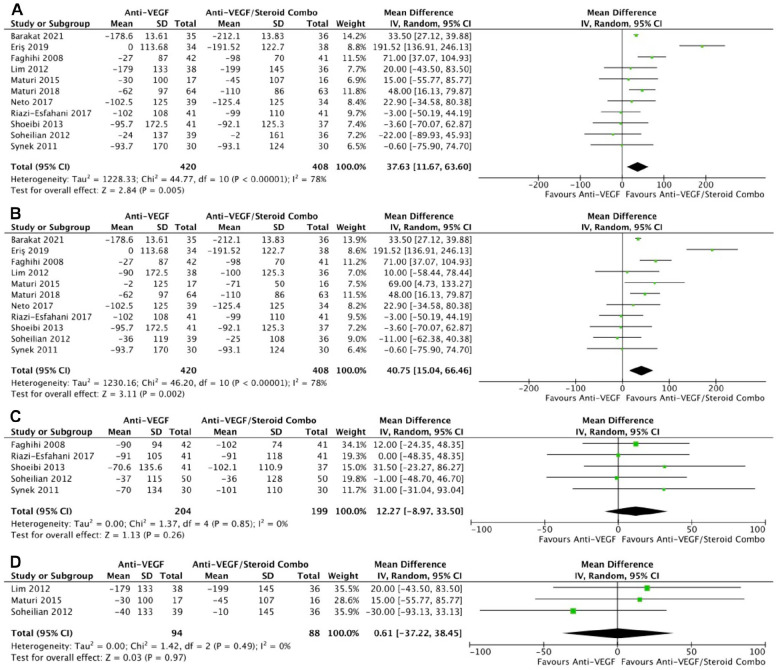
Change in retinal thickness over time. (A) At last study observation. (B) At 4 to 6 months. (C) At 1 to 3 months. (D) At 10 to 12 months. Abbreviations: Anti-VEGF, antivascular endothelial growth factor; IV, inverse variance.

**Figure 5.**
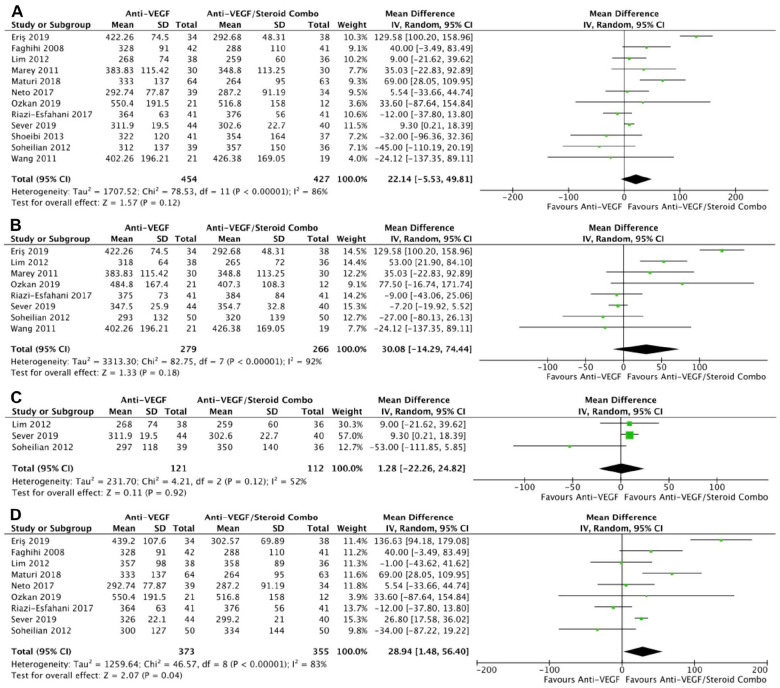
Retinal thickness over time. (A) At last study observation. (B) At 1 to 3 months. (C) At 10 to 12 months. (D) At 4 to 6 months. Abbreviations: Anti-VEGF, antivascular endothelial growth factor; IV, inverse variance.

In 11 RCTs, monotherapy was associated with a significantly smaller change in retinal thickness than combination therapy at the final follow-up (WMD, 37.63 μm; 95% CI, 11.67-63.60; *P* = .005; *I*^2^ = 78%) (Figure 4A) and at 4 to 6 months (WMD, 40.75 μm; 95% CI, 15.04-66.46; *P* = .001; *I*^2^ = 78%) (Figure 4B), with significant heterogeneity in both comparisons. However, the changes in retinal thickness were similar between the groups at 1 to 3 months (*P* = .26) (Figure 4C) and at 10 to 12 months (*P* = .97) (Figure 4D). The final retinal thickness was similar between the groups at the final follow-up (*P* = .12) (Figure 5A), at 1 to 3 months (*P* = .18) (Figure 5B), and at 10 to 12 months (*P* = .92) (Figure 5C). The retinal thickness was significantly greater with monotherapy at 4 to 6 months in 9 RCTs, although there was significant heterogeneity (WMD, 28.94 μm; 95% CI, 1.48-56.40; *P* = .04; *I*^2^ = 83%) (Figure 5D).

#### Adverse Events

**Figure 6.**
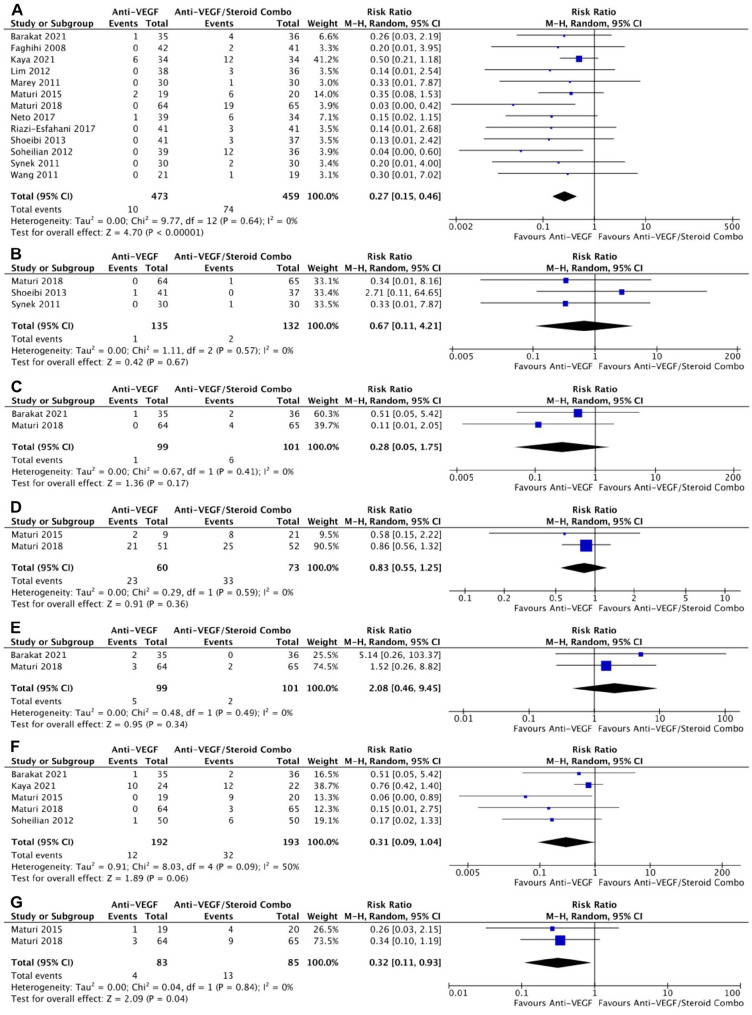
(A) Intraocular related-related AEs. (B) Intraocular inflammation AEs. (C) Conjunctival hemorrhage AEs. (D) Serious AEs. (E) Eye pain AEs. (F) Cataract-related AEs. (G) Vitreous floaters or hemorrhage. Abbreviations: AEs, adverse events; Anti-VEGF, antivascular endothelial growth factor; M-H, Mantel-Haenszel.

With respect to AEs, monotherapy was associated with a significantly lower risk for IOP-related AEs in 13 RCTs (risk ratio, 0.27; 95% CI, 0.15-0.46; *P* < .001; *I*^2^ = 0%) (Figure 6A). The risk for intraocular inflammation (*P* = .67) (Figure 6B), conjunctival hemorrhage (*P* = .17) (Figure 6C), serious AEs (*P* = .36) (Figure 6D), and eye pain (*P* = .34) (Figure 6E) were similar between groups. The risk for cataract-related AEs was not significantly different between groups in 5 RCTs; however, there were numerically fewer cataract-related events in the monotherapy group (*P* = .06) (Figure 6F). In 2 RCTs, monotherapy was associated with a significantly lower risk for vitreous floaters or hemorrhages than combination therapy (risk ratio, 0.32; 95% CI, 0.11-0.93; *P* = .04; *I*^2^ = 0%) (Figure 6G).

### Subgroup Analysis: Treatment Naive Anti-VEGF vs Combined Anti-VEGF and Steroid

Ten RCTs compared monotherapy vs combination therapy in treatment-naïve patients. Of the 20 outcomes analyzed, 17 were consistent with the results in the primary analysis (Supplemental Figure 1, a–t). The final BCVA was similar between monotherapy and combination therapy in treatment-naïve patients (*P* = .14) (Supplemental Figure 1g). The BCVA at 10 to 12 months was also similar between the 2 groups (*P* = .15) (Supplemental Figure 1h). There was no significant difference in retinal thickness between the groups at 4 to 6 months (*P* = 1.00) (Supplemental Figure 1r).

### Subgroup Analysis: Treatment-Resistant Anti-VEGF vs Combined Anti-VEGF and Steroid

Six RCTs compared monotherapy vs combination therapy in treatment-resistant patients, defined as having received any treatment for DME. Of the 29 outcomes analyzed, 15 were consistent with the results in the primary analysis (Supplemental Figure 2, a-t). In 2 RCTs, the change in BCVA at 0 to 3 months was significantly smaller with monotherapy than with combination therapy in treatment-resistant patients (WMD, 0.08 logMAR; 95% CI, 0.01-0.16; *P* = .03; I^2^ = 0%) (Supplemental Figure 2e). The BCVA at 4 to 6 months was significantly worse in the monotherapy group in 3 RCTs (WMD, 0.05 logMAR; 95% CI, 0.05-0.05; *P* ≤ .001; *I*^2^ = 0%) (Supplemental Figure 2h). The change in retinal thickness at the final follow-up (Supplemental Figure 2i) and at 4 to 6 months (Supplemental Figure 2j) was similar between the 2 groups (*P* = .14 and *P* = .06, respectively). The risk for cataract-related AEs was significantly lower in the monotherapy group in 2 RCTs (risk ratio, 0.09; 95% CI, 0.01-0.66; *P* = .02; *I*^2^ = 0%) (Supplemental Figure 2s).

### Subgroup Analysis: Bevacizumab vs Combined Bevacizumab and Steroid

Eleven RCTs compared bevacizumab vs combined bevacizumab and steroid. Of the 21 outcomes analyzed, 15 were consistent with the results in the primary analysis (Supplemental Figure 3, a–u). Although not statistically significant, the BCVA was numerically better at the final follow-up in the monotherapy group (*P* = .06) (Supplemental Figure 3g). The BCVA at 10 to 12 months was similar between the groups (*P* = .15) (Supplemental Figure 3h). The change in retinal thickness at the final follow-up (Supplemental Figure 3k) and at 4 to 6 months (Supplemental Figure 3i) was not significantly different between groups (*P* = .16 and *P* = .10, respectively. The retinal thickness at 4 to 6 months was similar between the 2 groups (*P* = 1.00) (Supplemental Figure 3r). In 2 RCTs, the risk for cataract-related AEs was significantly lower in the monotherapy group (risk ratio, 0.11; 95% CI, 0.02-0.59; *P* = .01; *I*^2^ = 0%) (Supplemental Figure 3u).

### Subgroup Analysis: Ranibizumab vs Combined Ranibizumab and Steroid

Four RCTs compared ranibizumab vs combined ranibizumab and steroid. Of the 11 outcomes analyzed, 6 were consistent with the results in the primary analysis (Supplemental Figure 4, a-k). In 3 RCTs, the BCVA at 4 to 6 months was significantly worse in the monotherapy group than in the combination therapy group (WMD, 0.05; 95% CI, 0.05-0.05; *P* < .001; *I*^2^ = 0%) (Supplemental Figure 4d). The change in retinal thickness at the final follow-up (Supplemental Figure 4e) and at 4 to 6 months (Supplemental Figure 4f) was not significantly different between the 2 groups (both *P* = .10). A risk for IOP-related AEs (Supplemental Figure 4j) and cataract-related AEs (Supplemental Figure 4k) was similar between groups (*P* = .28 and *P* = .41, respectively).

### Subgroup Analysis: Anti-VEGF vs Combined Anti-VEGF and Dexamethasone

Four RCTs compared anti-VEGF vs combined anti-VEGF and an intravitreal (IVT) dexamethasone implant. Of the 12 outcomes analyzed, 10 were consistent with the results in the primary analysis (Supplemental Figure 5, a–l). In 2 RCTSs, monotherapy was associated with significantly worse BCVA at 4 to 6 months (WMD, 0.05; 95% CI, 0.05-0.05; *P* ≤ .001; *I*^2^ = 0%) (Supplemental Figure 5d). The risk for IOP-related AEs was not significantly different between groups; however, the monotherapy group had fewer IOP-related AEs (*P* = .07) (Supplemental Figure 5i).

### Subgroup Analysis: Anti-VEGF vs Combined Anti-VEGF and Triamcinolone Acetonide

Twelve 12 RCTs compared anti-VEGF vs combined anti-VEGF and triamcinolone acetonide. Of the 21 outcomes analyzed, 18 were consistent with the results in the primary analysis (Supplemental Figure 6, a-u). Although not significant, the BCVA was better at the final follow-up in the monotherapy group (*P* = .07) (Supplemental Figure 6g). The BCVA at 10 to 12 months was not significantly different between the 2 groups (*P* = .15) (Supplemental Figure 6h). The retinal thickness was similar between the groups at 4 to 6 months (*P* = .29) (Supplemental Figure 6r).

## Conclusions

This meta-analysis analyzed the safety and efficacy of anti-VEGF agents compared with anti-VEGF combined with steroids for the treatment of DME. The main analysis found no significant differences in the change in BCVA from baseline to the final follow-up. The changes in retinal thickness were significantly greater with combination therapy than with monotherapy at the final follow-up, which ranged from 3 to 24 months. In the subgroup analysis of studies that used dexamethasone, the BCVA at 4 to 6 months was better in the combination group without a significantly increased risk for IOP-related AEs. This highlights the potential value of an IVT dexamethasone implant as an adjunct to anti-VEGF monotherapy.

Anti-VEGF agents primarily work to reduce the amount of binding between VEGF and retinal endothelial cells because VEGF increases vascular permeability and angiogenesis.^36^ Ranibizumab and bevacizumab are monoclonal antibodies that bind to the receptor binding site on VEGF, preventing the interaction of VEGF and receptors on endothelial cells.^37,38^ Aflibercept functions as a decoy protein receptor for VEGF as a result of its much higher affinity than the receptors on endothelial cells.^39^ Steroids are effective in reducing DME by inhibiting the production of various inflammatory cytokines, including P-selectin and ICAM-1, which increase leukocyte–endothelium interactions and contribute to the breakdown of the blood–retinal barrier.^40^ Furthermore, steroids have been shown to lower VEGF concentrations when administered individually, although less so than an anti-VEGF agent alone.^41^ It is possible that the improvement in retinal thickness observed with combination therapy is the result of the combined mechanism of actions providing a greater reduction in VEGF and other inflammatory cytokine concentrations.

Although there is a correlation between retinal thickness and BCVA in patients with DME, it is reported to be in the range of 0.2 to 0.3 after treatment.^42,43^ This weak correlation may explain the significant change in retinal thickness observed without a corresponding significant change in BCVA in our analysis. The aforementioned outcomes continue to be used in clinical settings since phase II and III clinical trials of anti-VEGF for DME used these outcome measures.^44^ Although optical coherence tomography measures have value in the clinical evaluation of DME, they are cannot be reliably used as a surrogate for VA outcomes.^45^

IOP-related AEs occurred significantly more often with combination therapy, and there was a trend toward an increased risk for cataract formation with this therapy. A significant difference was observed between treatment groups in the BCVA at the final follow-up. BCVA values measured at final observation timepoints are more prone to bias and may underestimate or overestimate the effects of treatment because of a variety of factors, such as baseline differences between groups across studies.^46^ In our review, the results of the subgroup analysis of treatment-naïve patients were similar to those in the primary analysis. The findings in our analysis of treatment-resistant patients were similar to those in the primary analysis except for a similar change in retinal thickness and an increased risk for cataract formation with combination therapy.

A 2018 Cochrane review by Mehta et al^47^ examined the efficacy and safety of anti-VEGF monotherapy compared with anti-VEGF and steroid combination therapy. The study had lower power comparisons, warranting further exploration into the topic given that more research has since been published. Our current analysis had 8 more RCTs and 349 more eyes than the review by Mehta et al.^[Bibr bibr47-24741264241280597]^ Similar to our findings, no significant differences were observed in the change in BCVA and combination therapy carried a greater a risk for IOP-related AEs. Although the in the analysis by Mehta et al cataract formation was significantly more frequent in combination therapy, our analysis did not find a significant difference between groups. However, our analysis found a significantly smaller change in retinal thickness with monotherapy than with combination therapy at the final follow-up, whereas Mehta et al did not find a significant difference between the 2 groups. In addition, Mehta et al did not perform a subgroup analysis comparing treatment-naïve patients and treatment-resistant patients with DME.

It is well documented that the administration of steroids is associated with increased IOP and cataract formation.^48,49^ Our main analysis found a significant difference in IOP-related AEs only; however, there was an increased trend toward cataract formation in the combination therapy group (*P* = .06). Even though the weighted mean follow-up was 12.0 ± 6.9 months, the follow-up in the studies included in our review might not have been long enough because most cataracts that develop after steroid injections are not appreciable until 1 to 2 years later, if not longer.^50^ Steroids alter the trabecular meshwork structure by causing formation of crosslinked actin and increasing extracellular matrix deposition. Both changes increase aqueous outflow resistance and directly lead to increased pressure.^51,52^ The pathophysiology of steroid-induced cataracts is still not well understood, although it is believed to be a result of steroids altering gene transcription in the epithelial cells of the lens.^53^

Our study has limitations that are relevant when interpreting the findings. First, because of the differences in methodologies among studies, we pooled different follow-up times to obtain higher powered comparisons. However, this approach also increased the amount of statistical indirectness between comparators. Similarily, the length of follow-up varied substantially across studies, ranging from 3 months to 24 months, which limited the certainity of the evidence in the analysis.

In addition, the anti-VEGF agents used in the studies were primarily bevacizumab^20-25,27,29,30,32,34^ and ranibizumab,^26,28,33,35^ with only a single study using aflibercept.^31^ We were unable to perform a subgroup analysis of aflibercept because of the paucity of studies in the literature. Aflibercept appears to be associated with slightly better visual outcomes for DME than bevacizumab or ranibizumab.^54,55^ It is possible that the results in our analysis would be slightly different, with greater changes in BCVA, if more of the included studies had used aflibercept. In addition, newer anti-VEGF treatments have shown similar BCVA outcomes and require fewer injections than current anti-VEGF agents, making them an appealing option to many patients.^56,57^ However, we were unable to include these newer agents; thus, the conclusions of this study are not necessarily applicable to all anti-VEGF agents.

Because the AE analysis was underpowered, we placed ocular hypertension and IOP progression in the IOP-related AE category. Likewise, cataract development, cataract progression, and cataract surgery were grouped into the cataract-related AEs category. Although we were able to perform a subgroup analysis of treatment-naïve patients and treatment-resistant patients in our main group analysis, we were unable to make comparisons between these 2 groups for each drug pairing given limitations in the literature.

Another limitation of our study is there is not a consensus in the existing literature regarding the definition treatment resistant. We assigned 6 RCTs as treatment resistant because they explicitly provided information on treatment resistance, ranging from determining resistance after 3 loading anti-VEGF doses,^26^ unresponsive to previous macular laser photocoagulation,^24,25^ incomplete response to multiple anti-VEGF injections and a central subfield thickness of 250 µm,^34^ persistent DME after the 12-week run-in phase despite receiving 3 additional anti-VEGF injections,^33^ and a central macular thickness greater than 350 μm with less than 3 lines of visual gain after at least 6 anti-VEGF injections.^35^ The remaining RCTs were assigned as treatment naïve in our analysis. Therefore, clinicians should use caution when assigning naïve or refractory criteria to patients with DME as potential candidates that would benefit from combination therapy.

An important limitation of our meta-analysis is the variability of the analyzed studies, such as treatment schedules, type of anti-VEGF and steroid agents used, definitions of treatment resistance, the number of phakic eyes, and the length of follow-up. Although this diversity reflects real-world clinical scenarios, it may influence the generalizability of our findings. Our analysis attempted to mitigate these differences through rigorous statistical methods and subgroup analyses; however, the results should be interpreted with caution given the inherent variabilities among the included studies.

In conclusion, our meta-analysis of 16 studies comparing monotherapy with combination therapy for the treatment of DME found that combination therapy had a similar effect on visual outcomes and provided a greater reduction in retinal thickness. However, it was associated with an increased risk for IOP-related AEs and possibly cataract-related AEs. Similar findings were observed in treatment-naïve patients. The findings for treatment-resistant patients were similar other than a similar reduction in retinal thickness between groups and a significantly increased risk for cataract formation with combination therapy. Future research should develop standard criteria to define treatment resistance, and re-evaluation of this topic is warranted to provide a better understanding of the comparative efficacy and safety of these treatment modalities as new studies continue to be published.

## Supplemental Material

sj-zip-1-vrd-10.1177_24741264241280597 – Supplemental material for Anti-VEGF Monotherapy vs Anti-VEGF and Steroid Combination Therapy for Diabetic Macular Edema: A Meta-analysis Supplemental material, sj-zip-1-vrd-10.1177_24741264241280597 for Anti-VEGF Monotherapy vs Anti-VEGF and Steroid Combination Therapy for Diabetic Macular Edema: A Meta-analysis by Justin Grad, Amin Hatamnejad, Rohan Dadak, Simrat Sodhi, Niveditha Pattathil and Netan Choudhry in Journal of VitreoRetinal Diseases
