# Supplementary figures and images for: Anti-VEGF Monotherapy vs Anti-VEGF and Steroid Combination Therapy for Diabetic Macular Edema: A Meta-analysis (part 2 of 2)
Source: J Vitreoretin Dis. 2024 Oct 10;9(1):70–83. doi: 10.1177/24741264241280597 (PMC11556321; doi:10.1177/24741264241280597)

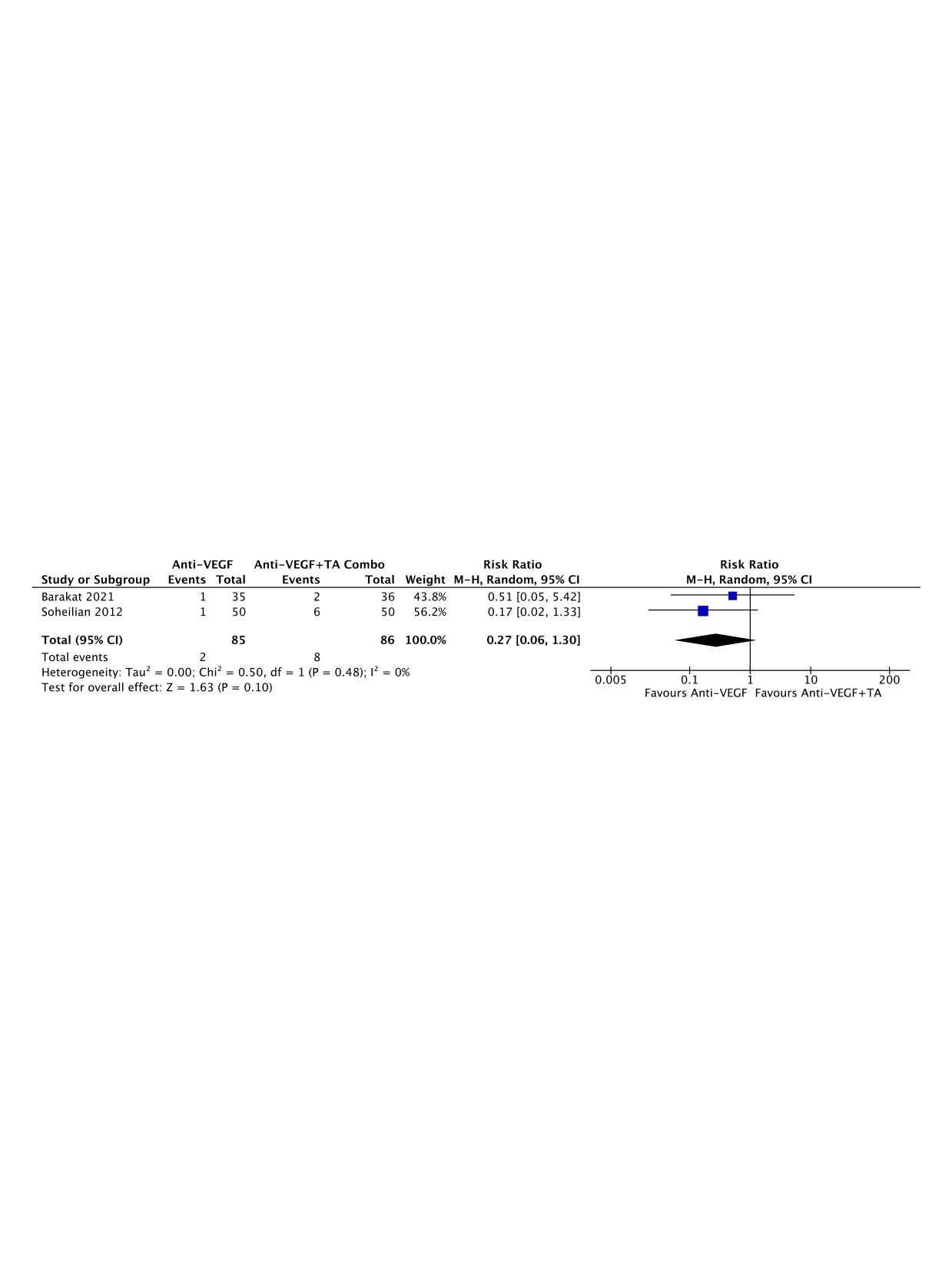

Supplement: sj-zip-1-vrd-10.1177_24741264241280597 – Supplemental material for Anti-VEGF Monotherapy vs Anti-VEGF and Steroid Combination Therapy for Diabetic Macular Edema: A Meta-analysis [file sj-zip-1-vrd-10.1177_24741264241280597.zip › Supplemental Figure 6. u.jpg]

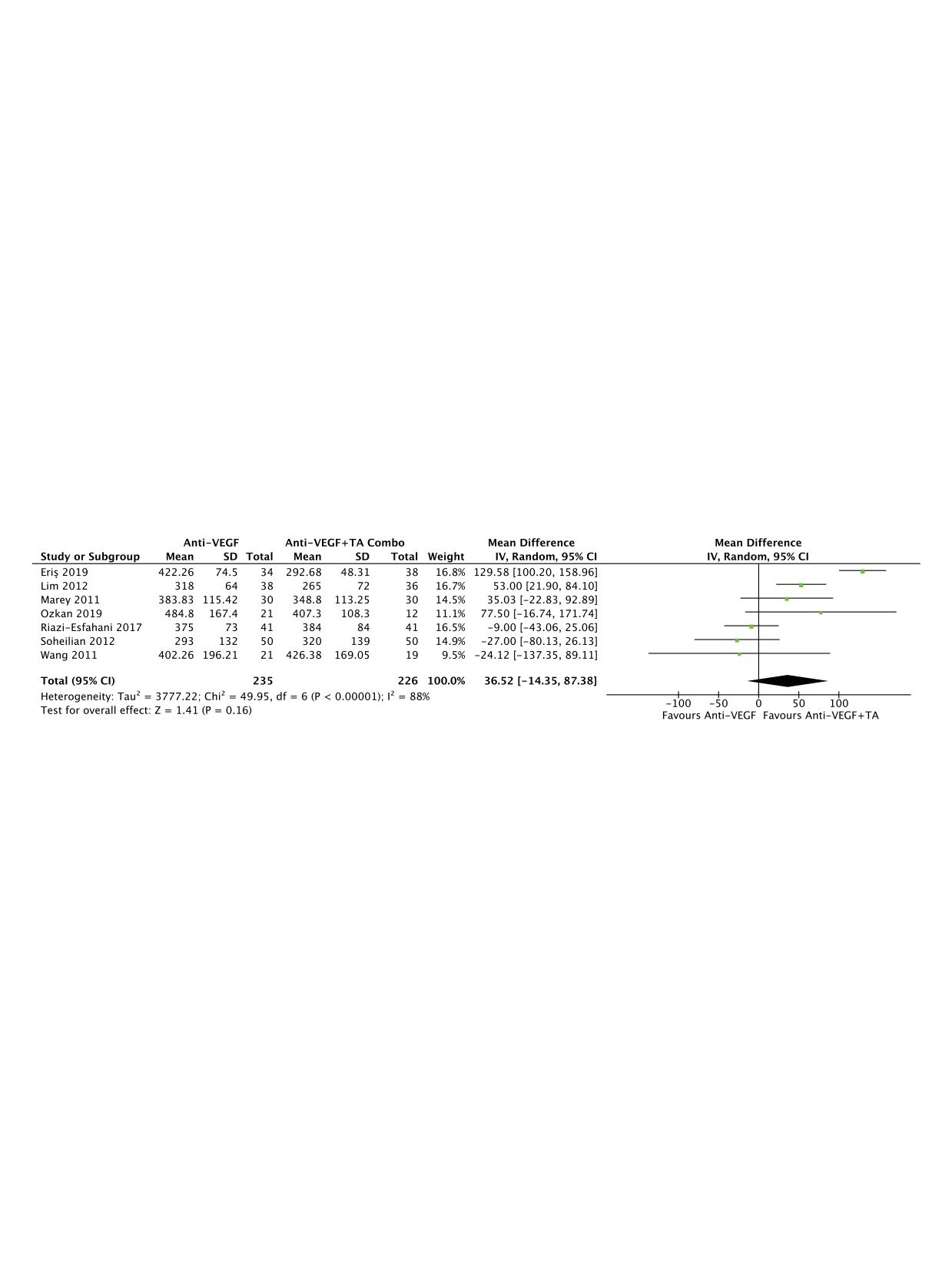

Supplement: sj-zip-1-vrd-10.1177_24741264241280597 – Supplemental material for Anti-VEGF Monotherapy vs Anti-VEGF and Steroid Combination Therapy for Diabetic Macular Edema: A Meta-analysis [file sj-zip-1-vrd-10.1177_24741264241280597.zip › Supplemental Figure 6. p.jpg]

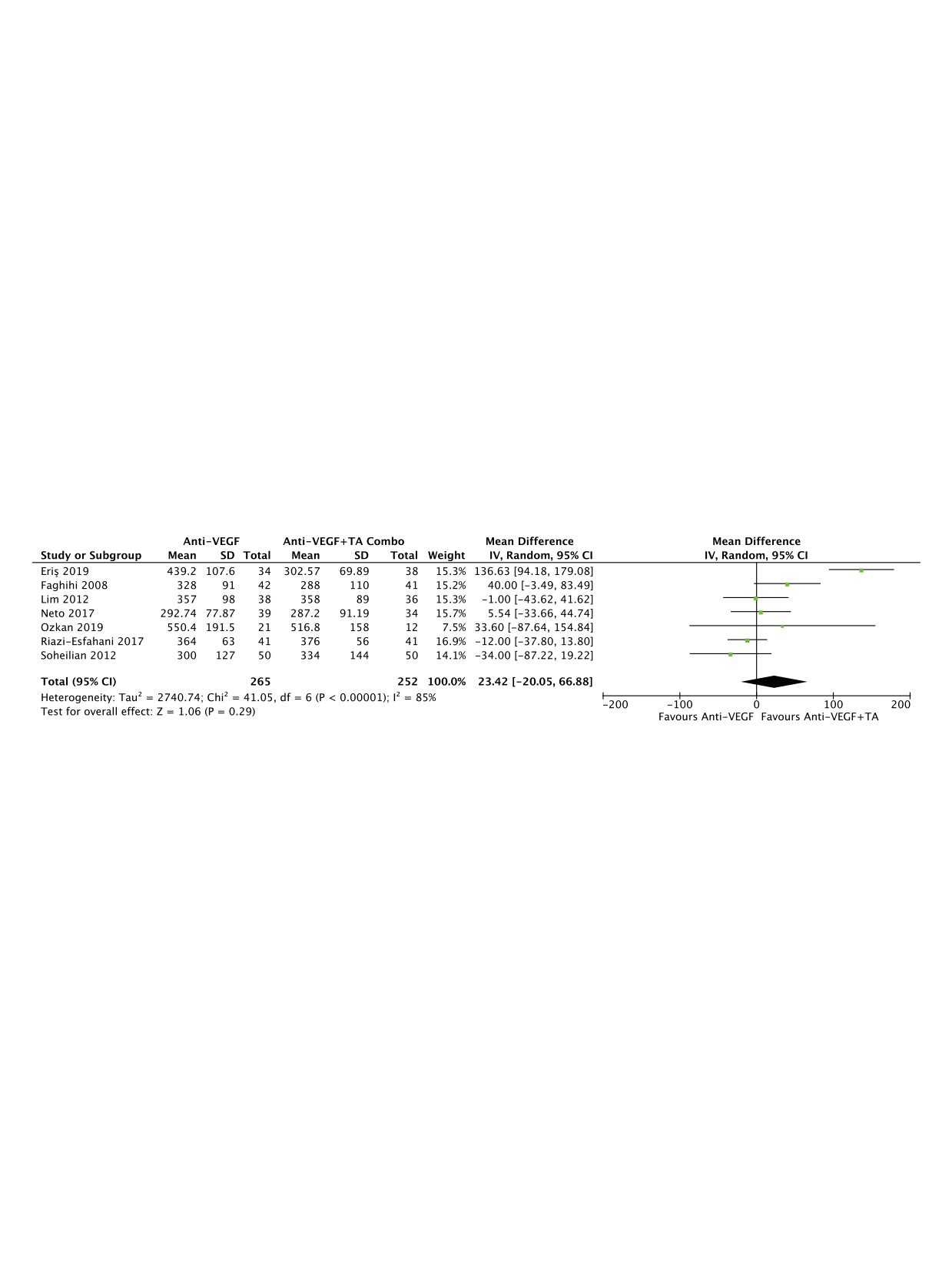

Supplement: sj-zip-1-vrd-10.1177_24741264241280597 – Supplemental material for Anti-VEGF Monotherapy vs Anti-VEGF and Steroid Combination Therapy for Diabetic Macular Edema: A Meta-analysis [file sj-zip-1-vrd-10.1177_24741264241280597.zip › Supplemental Figure 6. r.jpg]

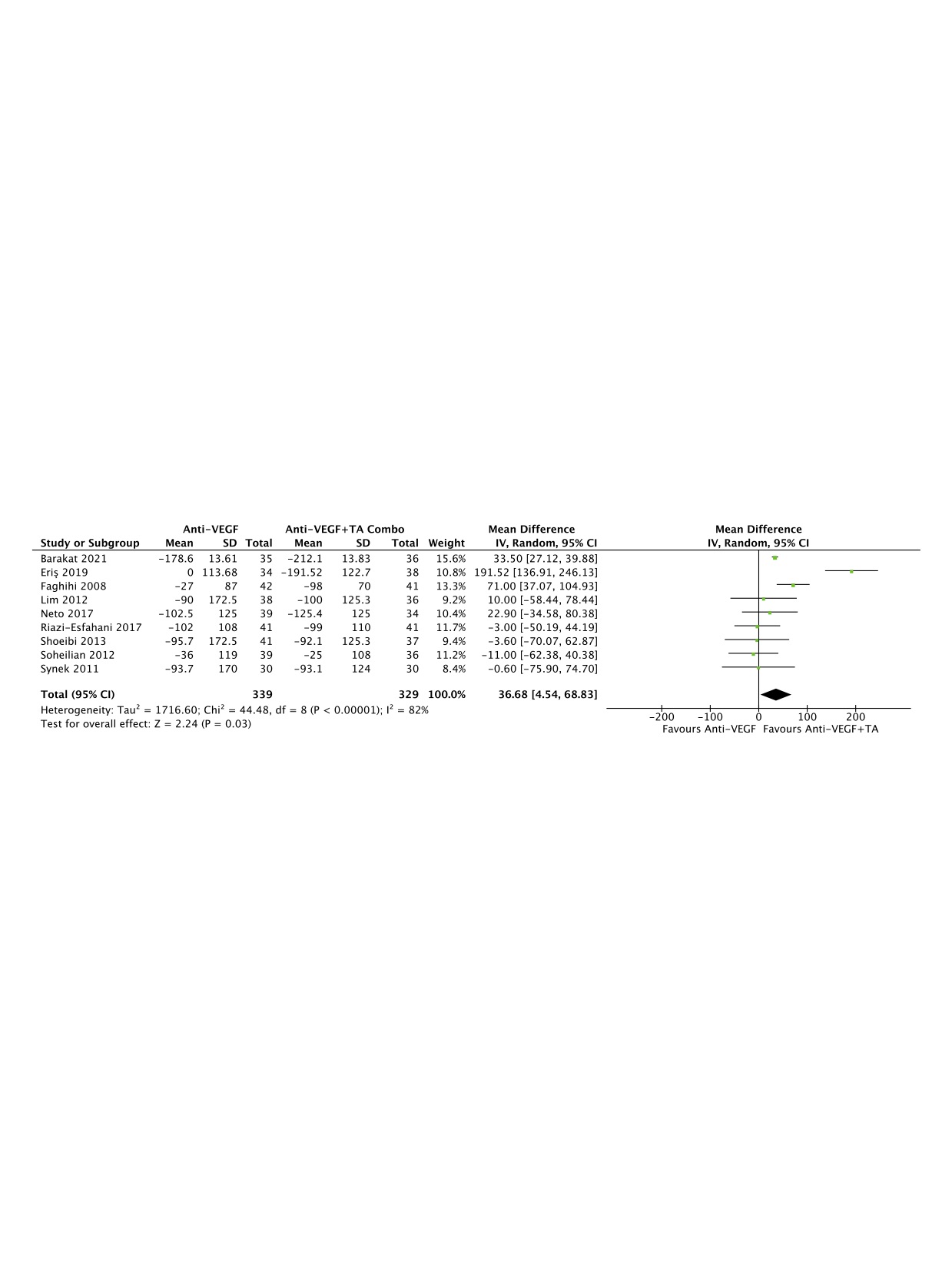

Supplement: sj-zip-1-vrd-10.1177_24741264241280597 – Supplemental material for Anti-VEGF Monotherapy vs Anti-VEGF and Steroid Combination Therapy for Diabetic Macular Edema: A Meta-analysis [file sj-zip-1-vrd-10.1177_24741264241280597.zip › Supplemental Figure 6. l.jpg]

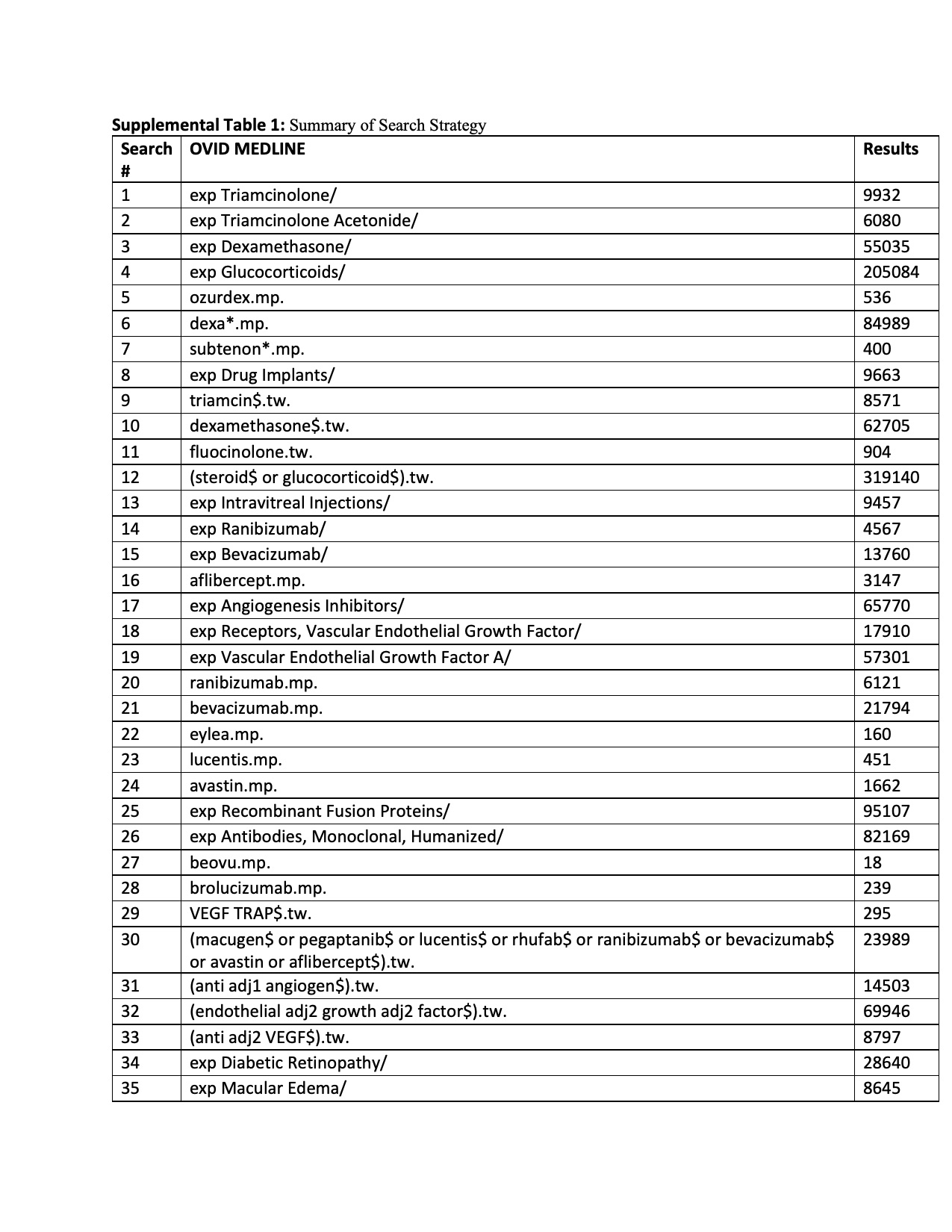

Supplement: sj-zip-1-vrd-10.1177_24741264241280597 – Supplemental material for Anti-VEGF Monotherapy vs Anti-VEGF and Steroid Combination Therapy for Diabetic Macular Edema: A Meta-analysis [file sj-zip-1-vrd-10.1177_24741264241280597.zip › Supplemental Table 1.jpg]

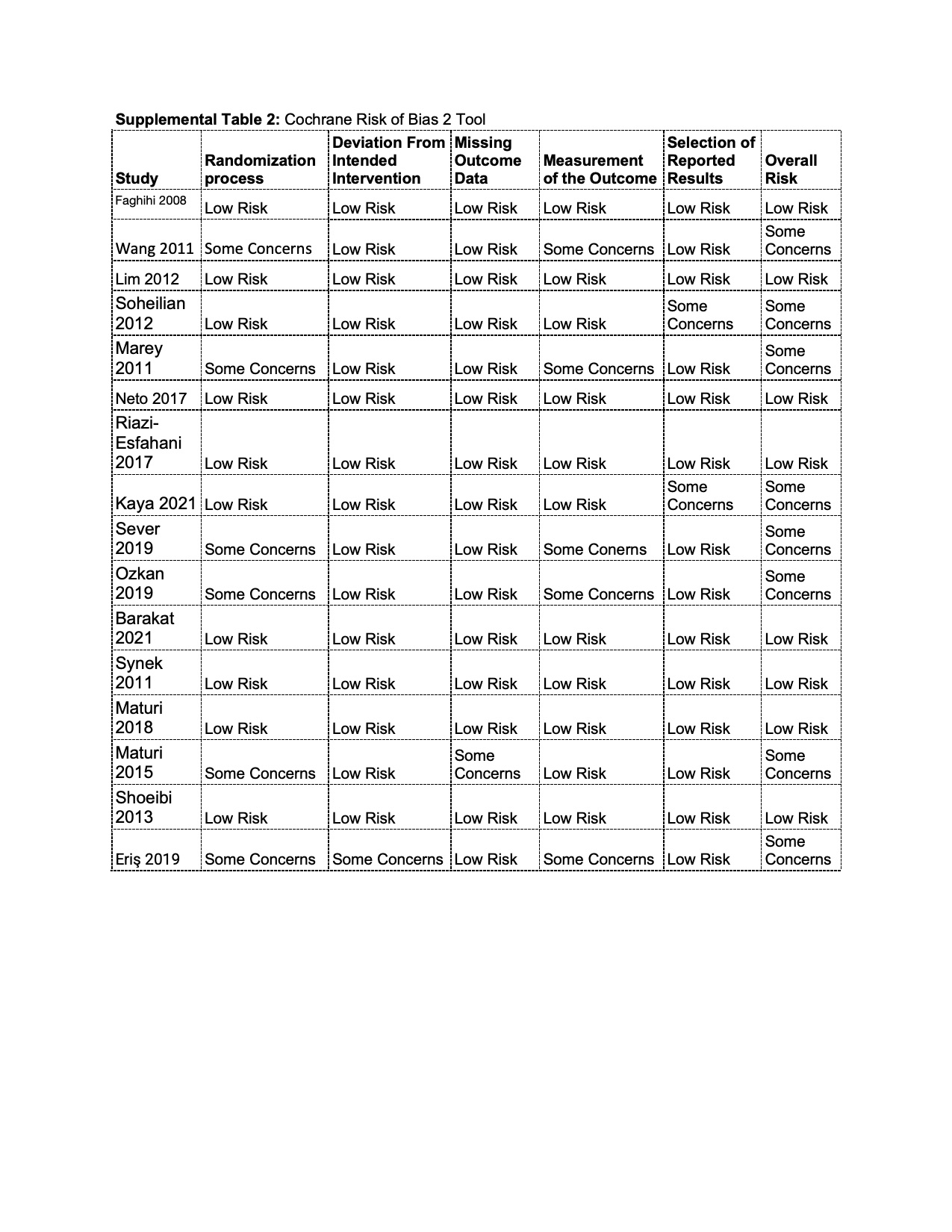

Supplement: sj-zip-1-vrd-10.1177_24741264241280597 – Supplemental material for Anti-VEGF Monotherapy vs Anti-VEGF and Steroid Combination Therapy for Diabetic Macular Edema: A Meta-analysis [file sj-zip-1-vrd-10.1177_24741264241280597.zip › Supplemental Table 2.jpg]

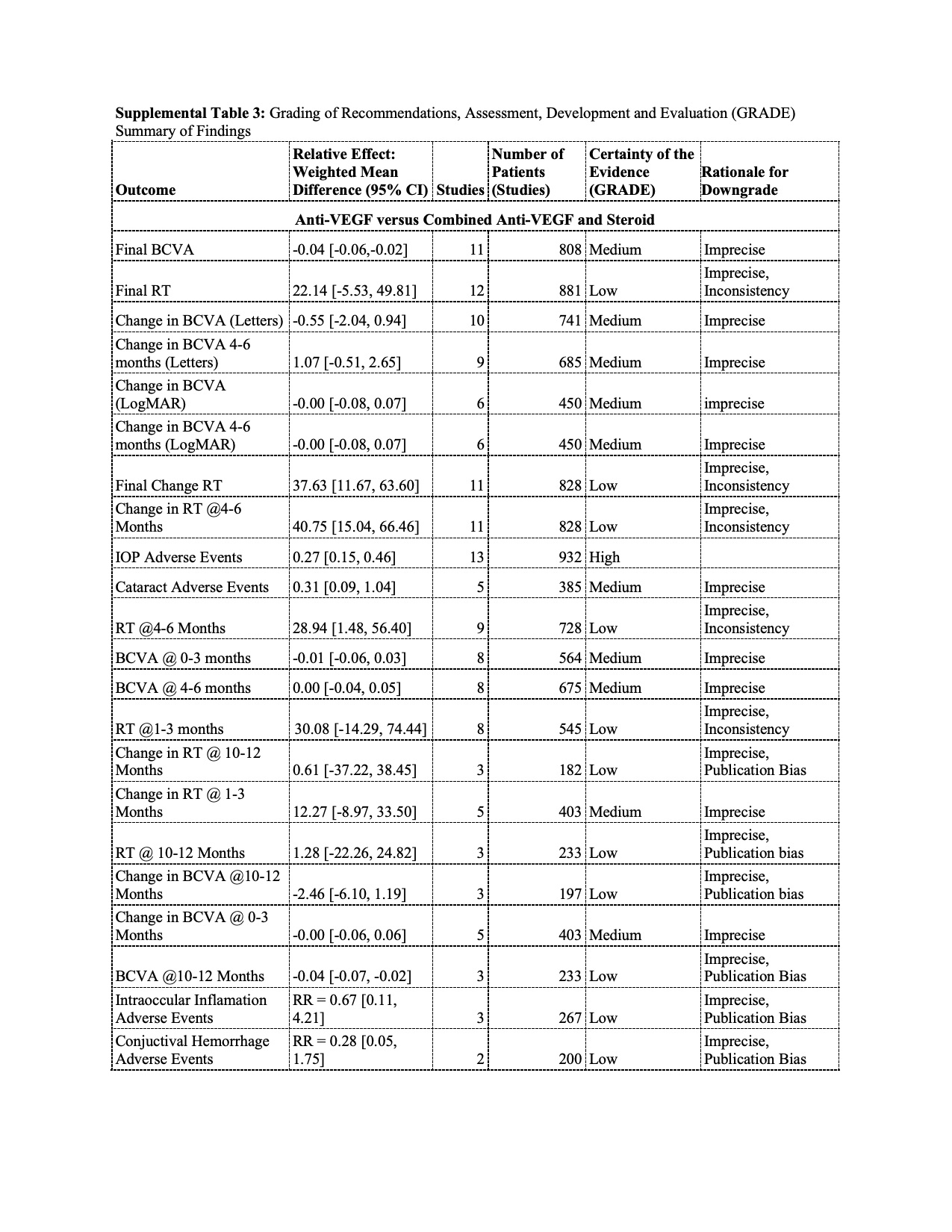

Supplement: sj-zip-1-vrd-10.1177_24741264241280597 – Supplemental material for Anti-VEGF Monotherapy vs Anti-VEGF and Steroid Combination Therapy for Diabetic Macular Edema: A Meta-analysis [file sj-zip-1-vrd-10.1177_24741264241280597.zip › Supplemental Table 3.jpg]
