# Supplementary figures and images for: Anti-VEGF Monotherapy vs Anti-VEGF and Steroid Combination Therapy for Diabetic Macular Edema: A Meta-analysis (part 1 of 2)
Source: J Vitreoretin Dis. 2024 Oct 10;9(1):70–83. doi: 10.1177/24741264241280597 (PMC11556321; doi:10.1177/24741264241280597)

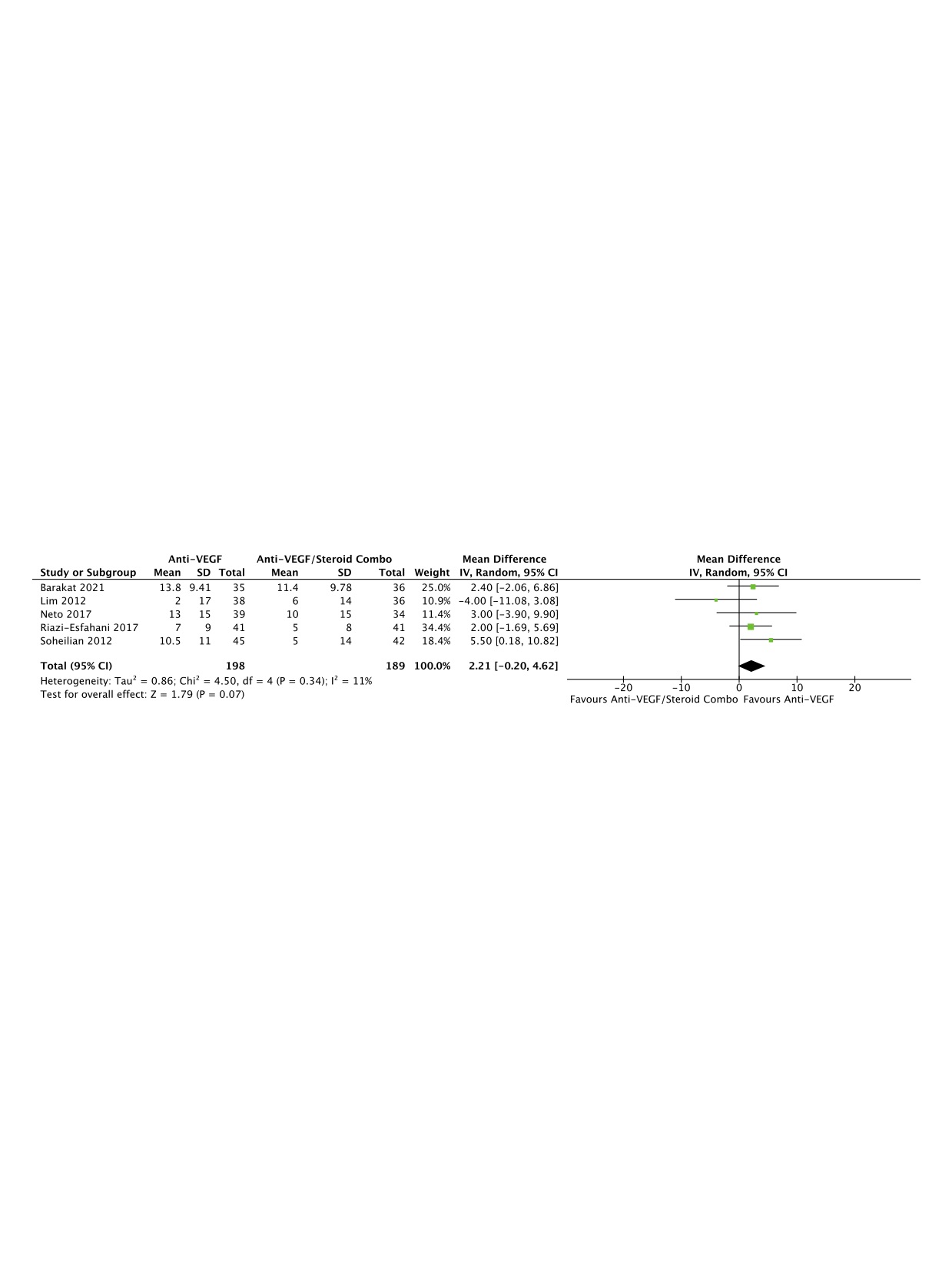

Supplement: sj-zip-1-vrd-10.1177_24741264241280597 – Supplemental material for Anti-VEGF Monotherapy vs Anti-VEGF and Steroid Combination Therapy for Diabetic Macular Edema: A Meta-analysis [file sj-zip-1-vrd-10.1177_24741264241280597.zip › Supplemental Figure 1. c.jpg]

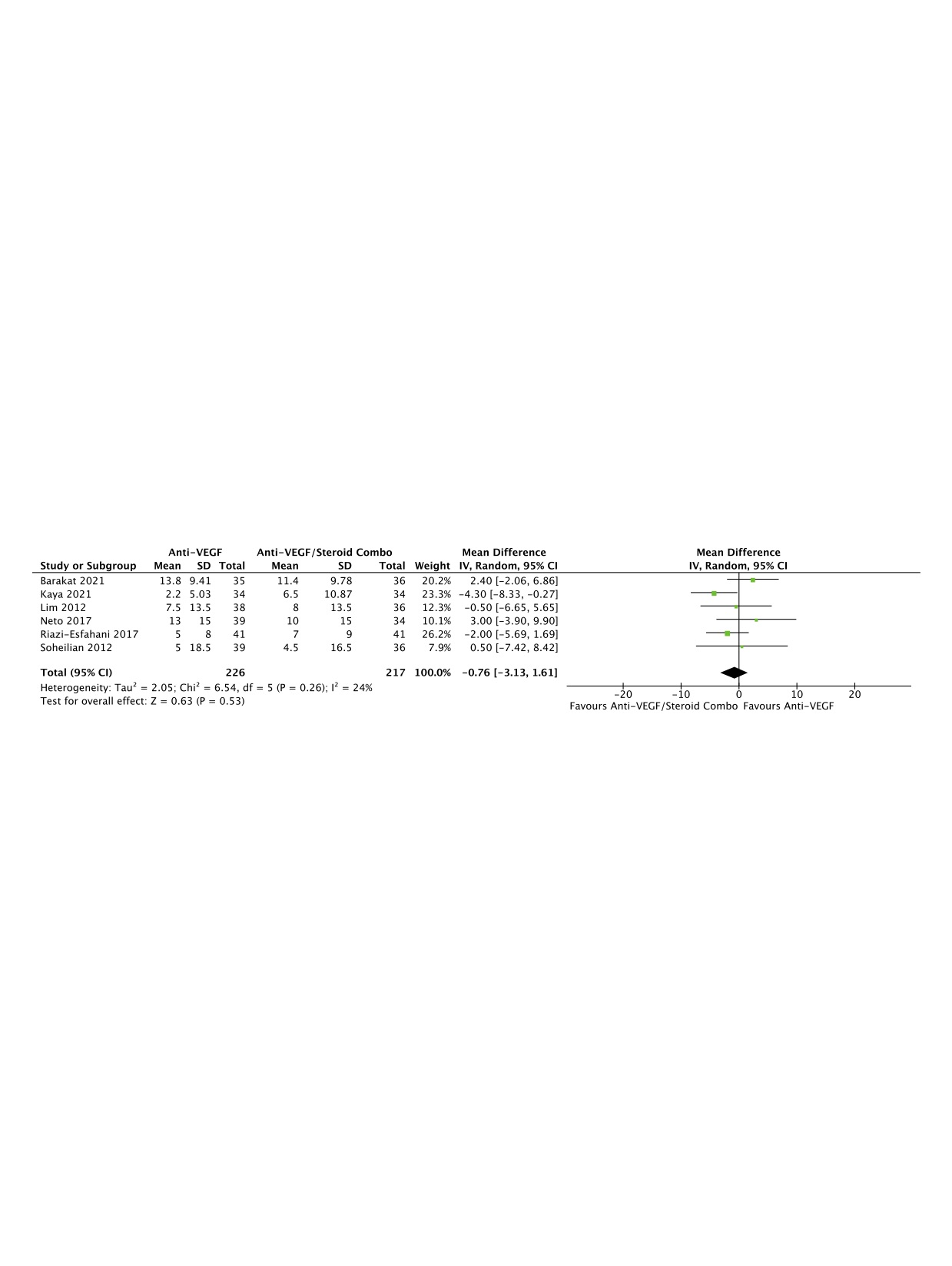

Supplement: sj-zip-1-vrd-10.1177_24741264241280597 – Supplemental material for Anti-VEGF Monotherapy vs Anti-VEGF and Steroid Combination Therapy for Diabetic Macular Edema: A Meta-analysis [file sj-zip-1-vrd-10.1177_24741264241280597.zip › Supplemental Figure 1. a.jpg]

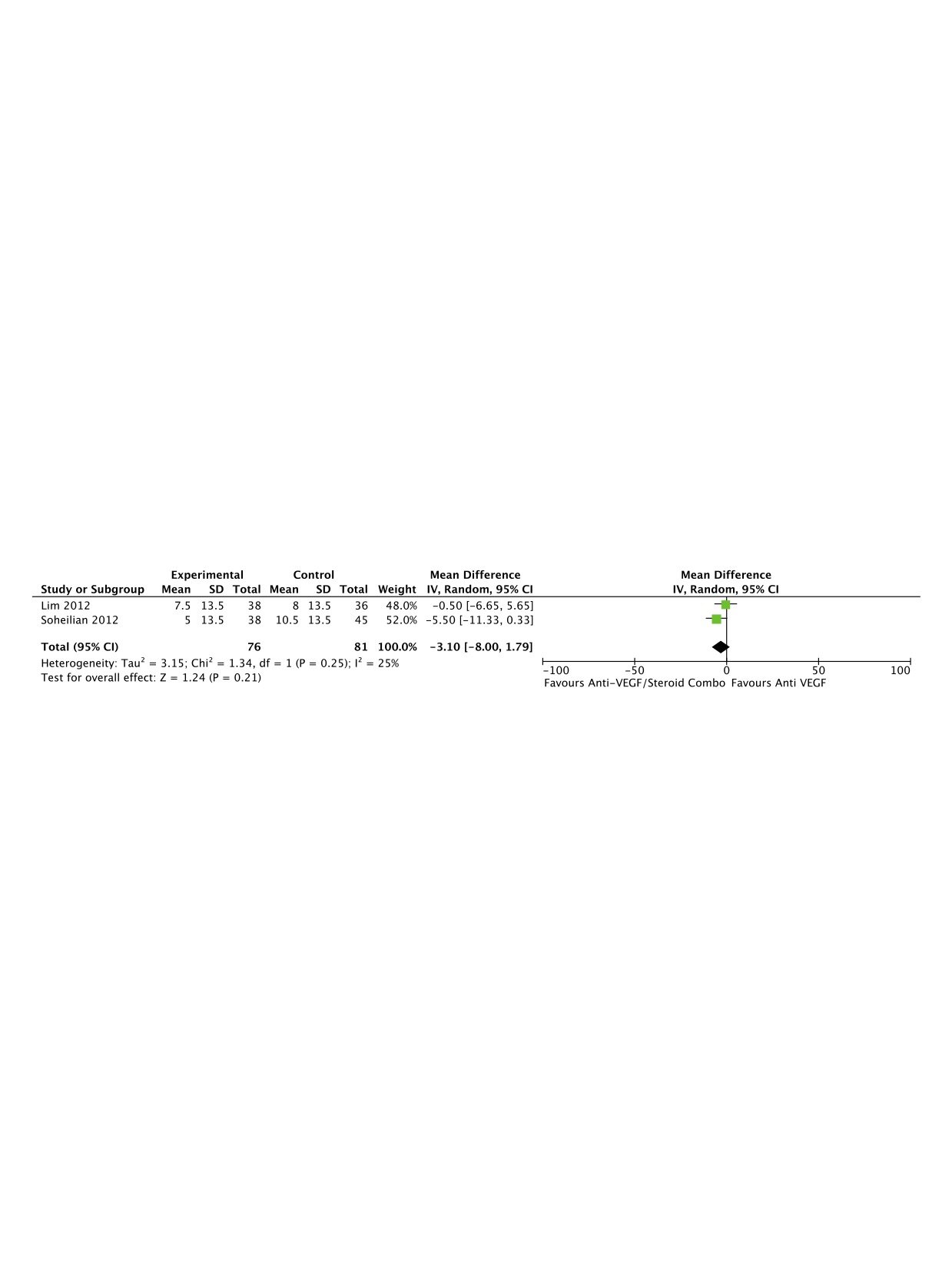

Supplement: sj-zip-1-vrd-10.1177_24741264241280597 – Supplemental material for Anti-VEGF Monotherapy vs Anti-VEGF and Steroid Combination Therapy for Diabetic Macular Edema: A Meta-analysis [file sj-zip-1-vrd-10.1177_24741264241280597.zip › Supplemental Figure 1. f.jpg]

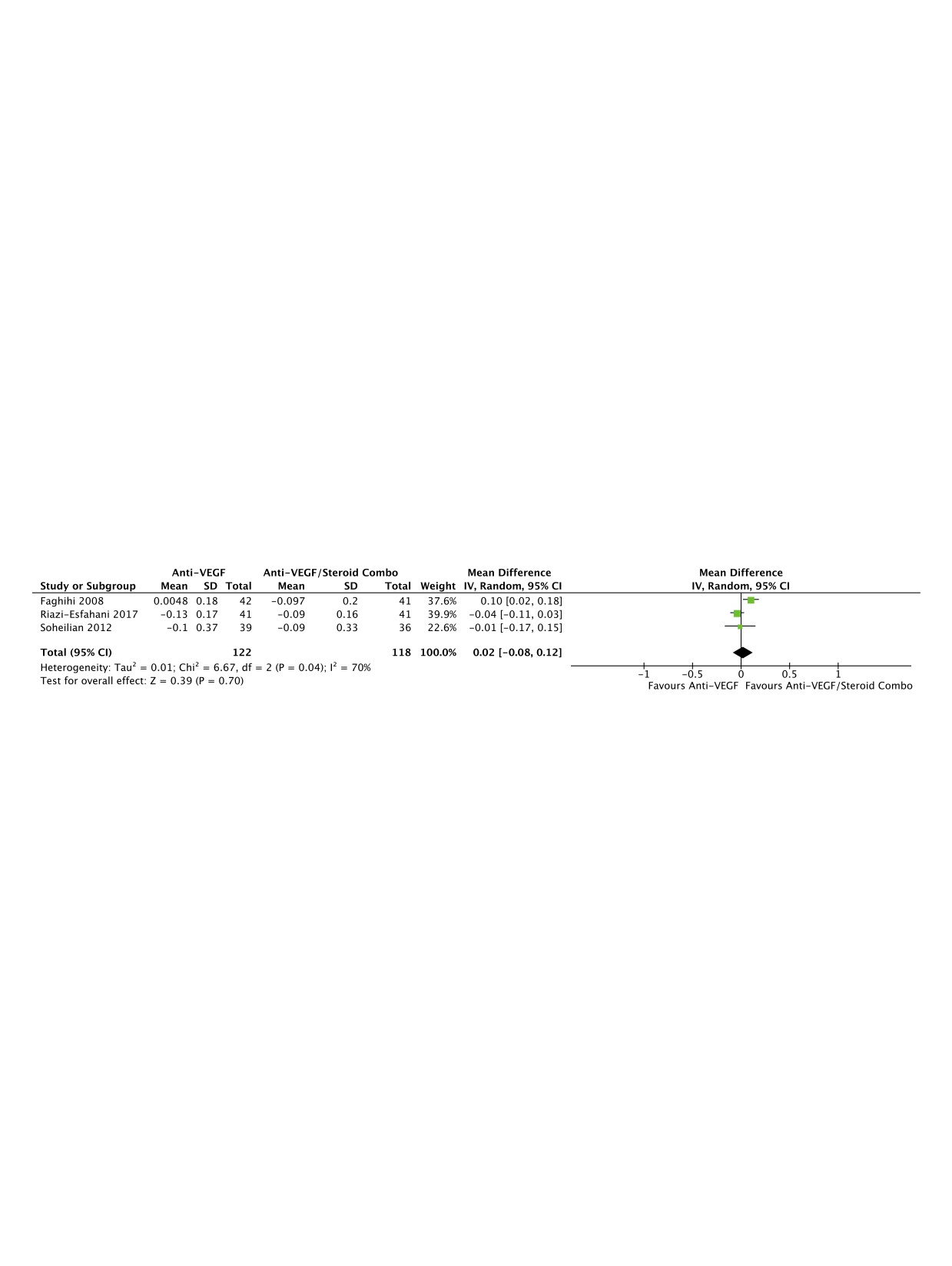

Supplement: sj-zip-1-vrd-10.1177_24741264241280597 – Supplemental material for Anti-VEGF Monotherapy vs Anti-VEGF and Steroid Combination Therapy for Diabetic Macular Edema: A Meta-analysis [file sj-zip-1-vrd-10.1177_24741264241280597.zip › Supplemental Figure 1. b.jpg]

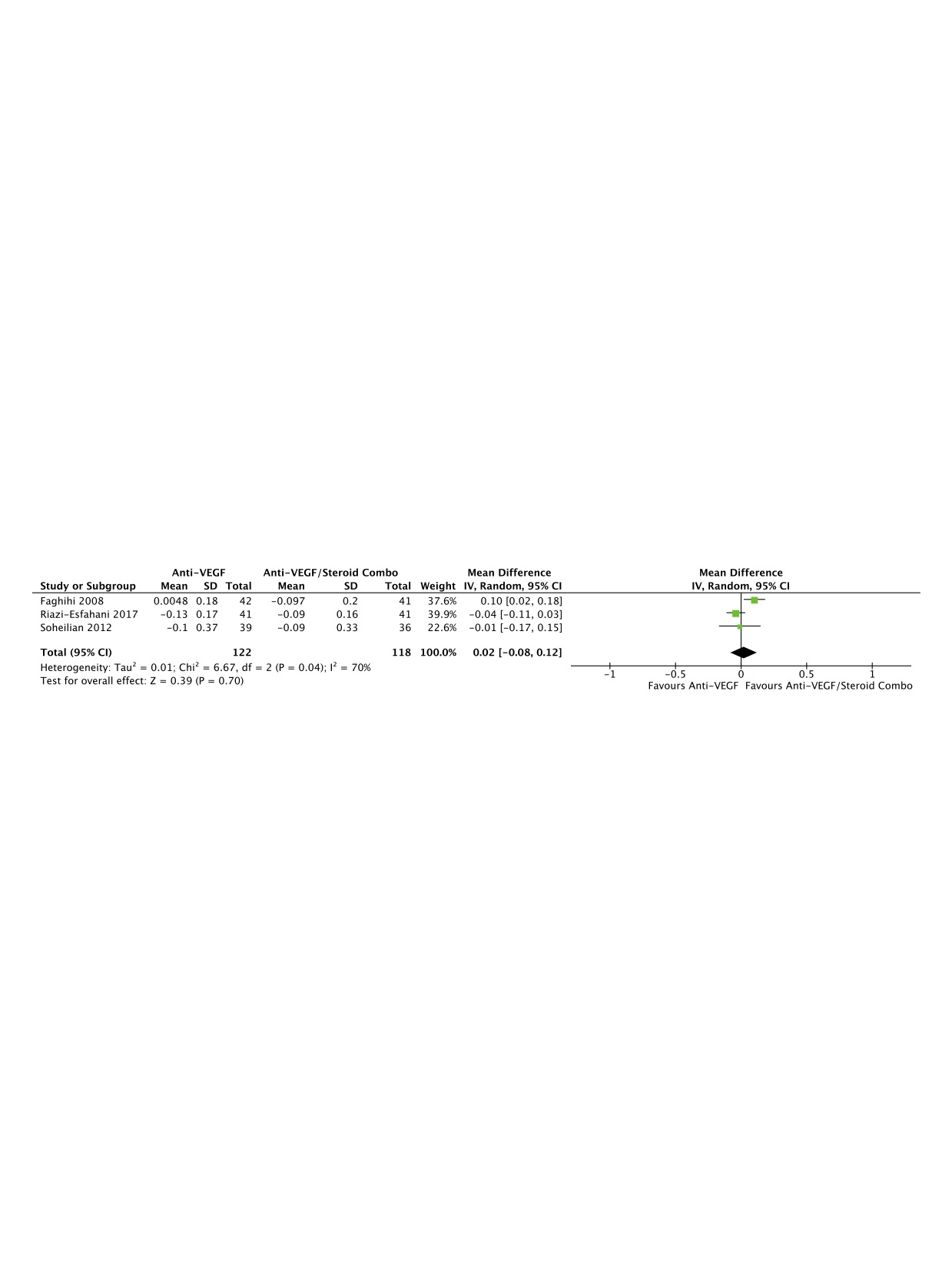

Supplement: sj-zip-1-vrd-10.1177_24741264241280597 – Supplemental material for Anti-VEGF Monotherapy vs Anti-VEGF and Steroid Combination Therapy for Diabetic Macular Edema: A Meta-analysis [file sj-zip-1-vrd-10.1177_24741264241280597.zip › Supplemental Figure 1. d.jpg]

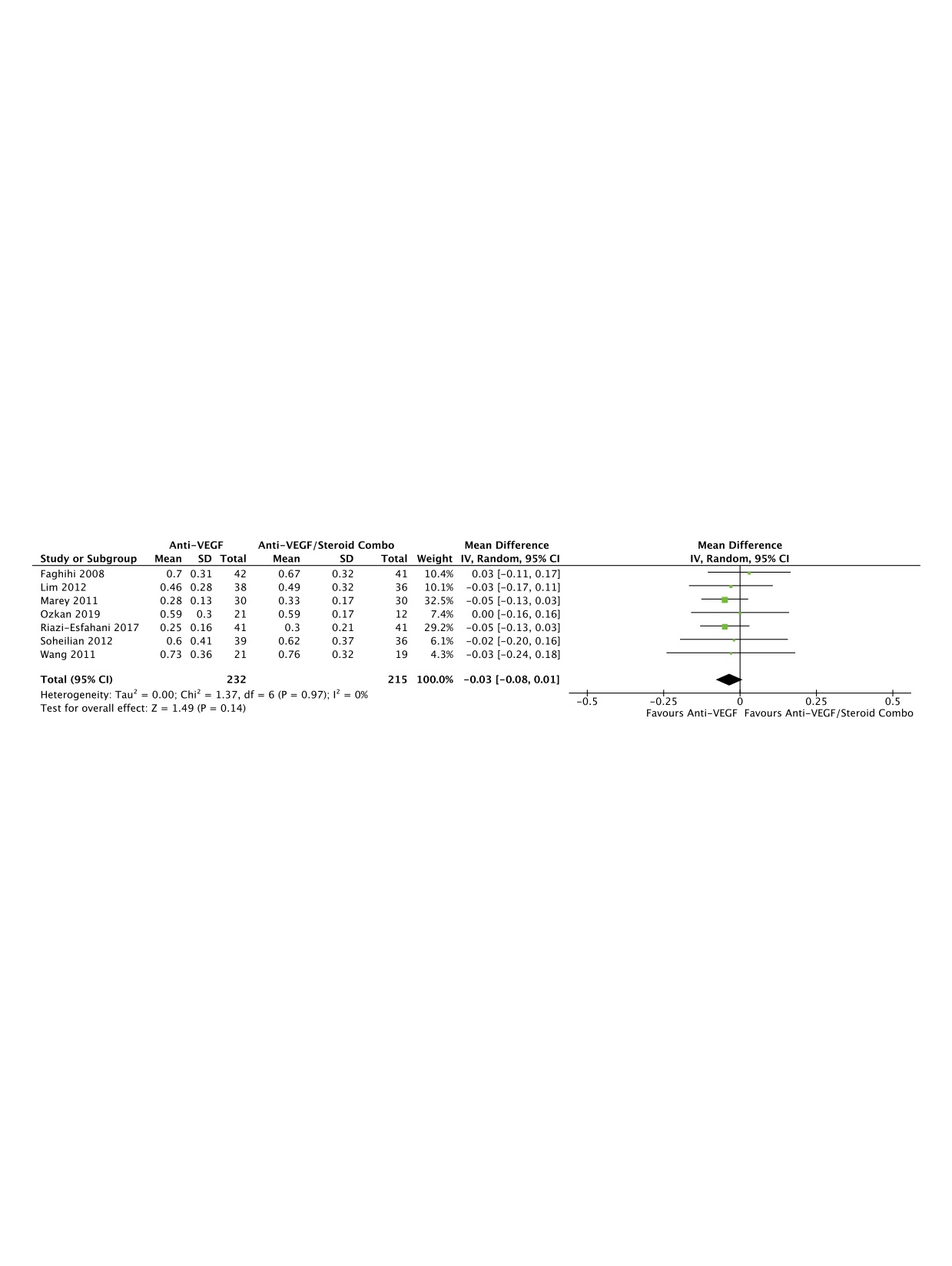

Supplement: sj-zip-1-vrd-10.1177_24741264241280597 – Supplemental material for Anti-VEGF Monotherapy vs Anti-VEGF and Steroid Combination Therapy for Diabetic Macular Edema: A Meta-analysis [file sj-zip-1-vrd-10.1177_24741264241280597.zip › Supplemental Figure 1. g.jpg]

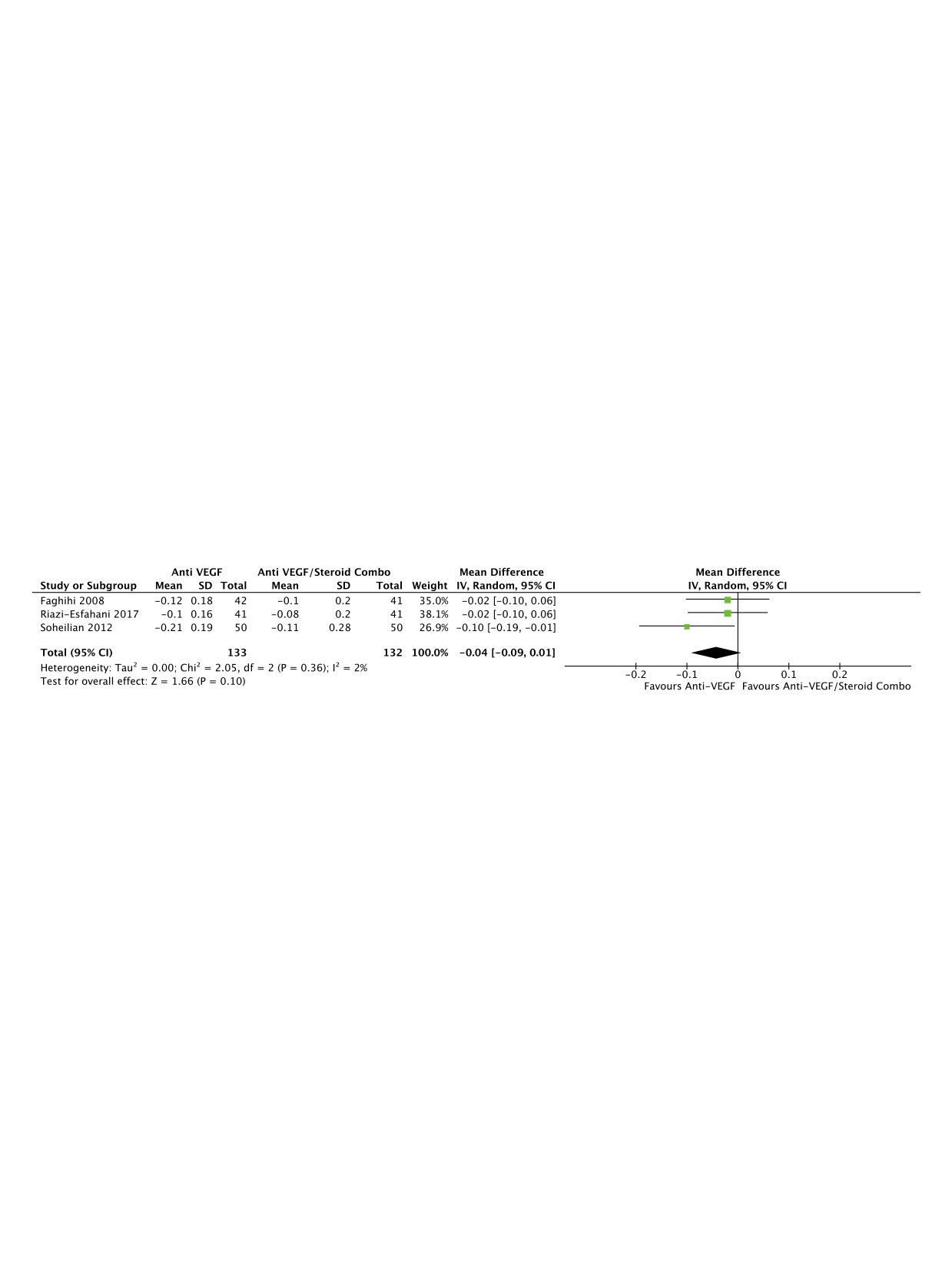

Supplement: sj-zip-1-vrd-10.1177_24741264241280597 – Supplemental material for Anti-VEGF Monotherapy vs Anti-VEGF and Steroid Combination Therapy for Diabetic Macular Edema: A Meta-analysis [file sj-zip-1-vrd-10.1177_24741264241280597.zip › Supplemental Figure 1. e.jpg]

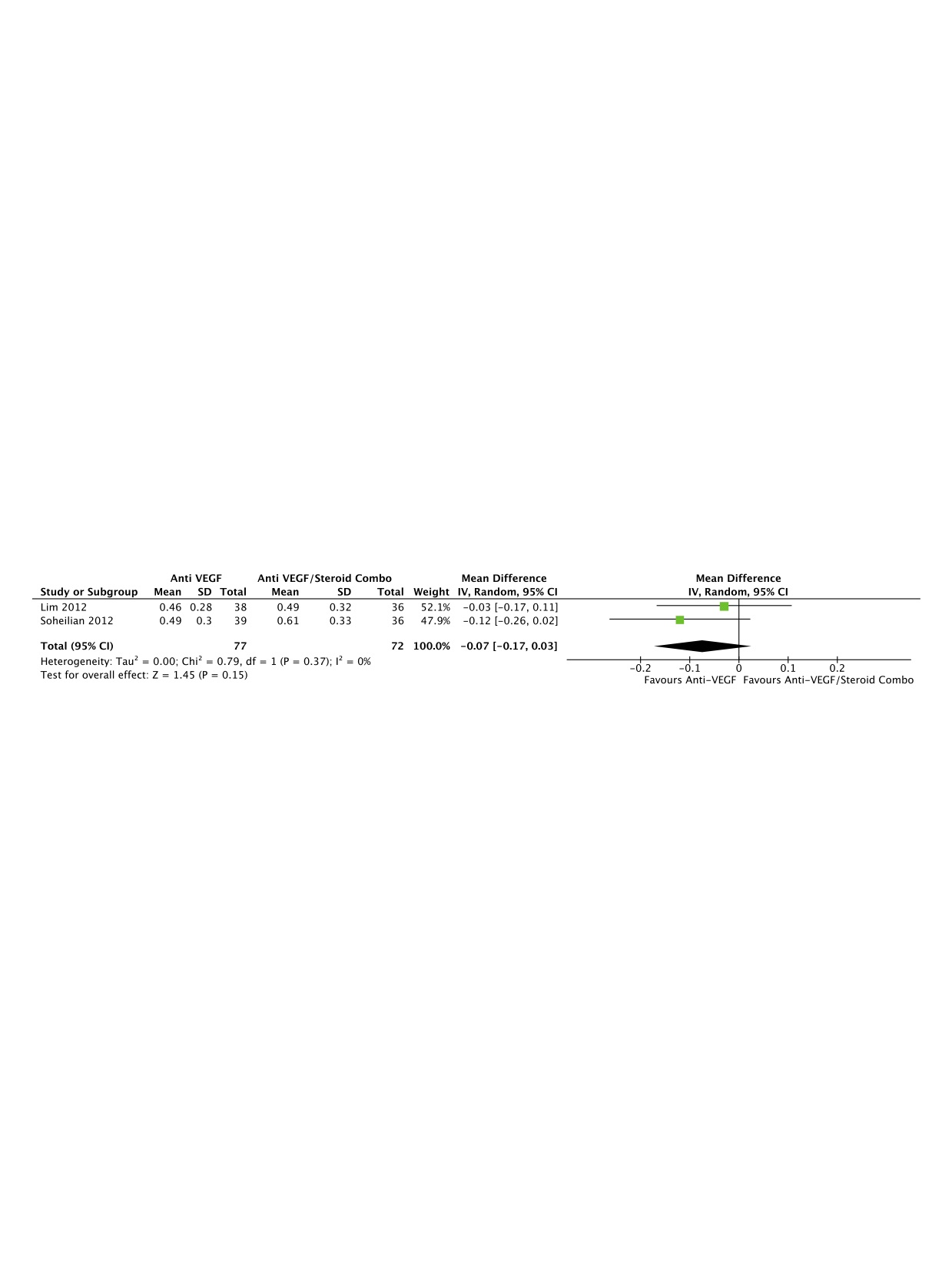

Supplement: sj-zip-1-vrd-10.1177_24741264241280597 – Supplemental material for Anti-VEGF Monotherapy vs Anti-VEGF and Steroid Combination Therapy for Diabetic Macular Edema: A Meta-analysis [file sj-zip-1-vrd-10.1177_24741264241280597.zip › Supplemental Figure 1. h.jpg]

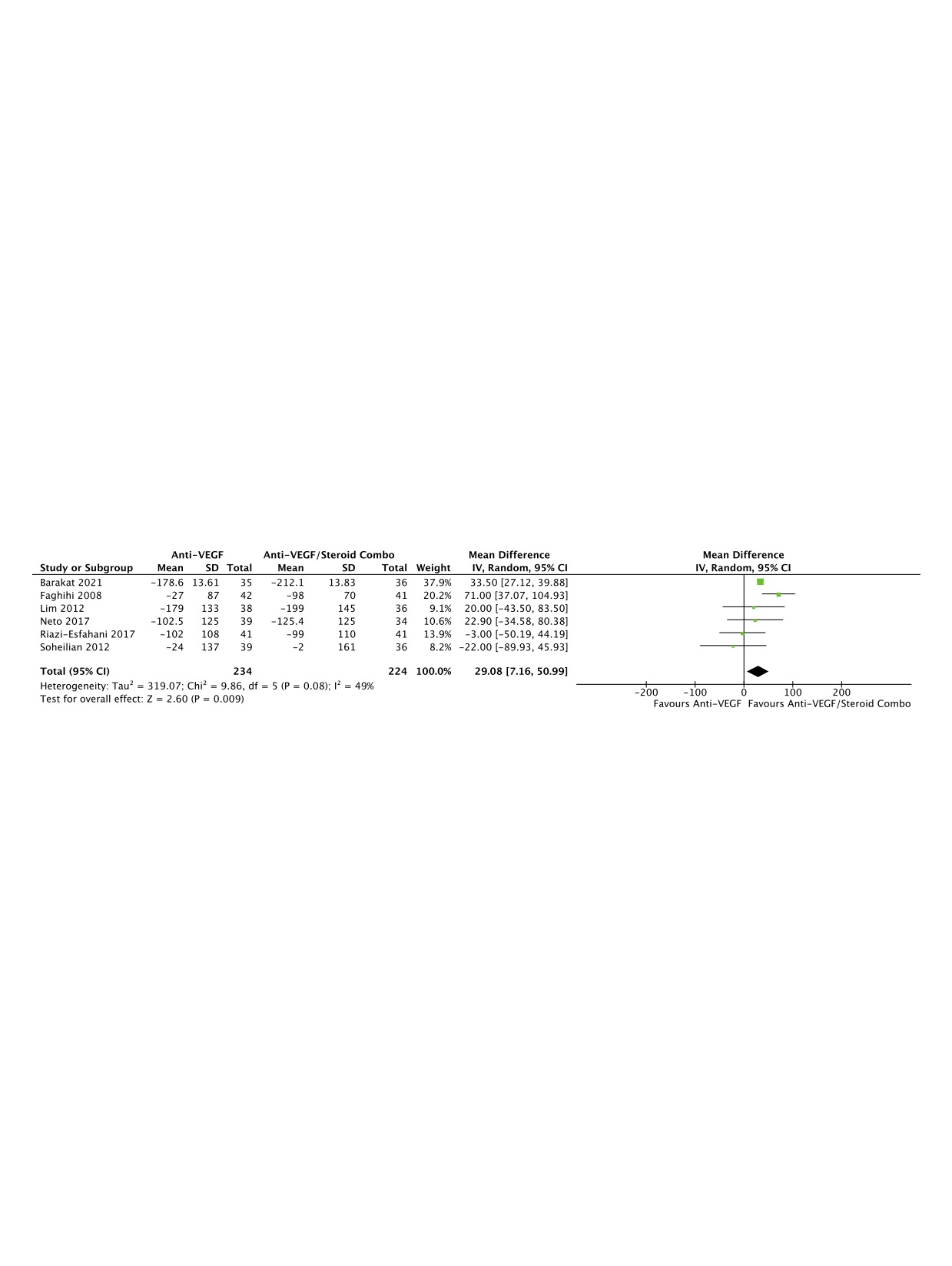

Supplement: sj-zip-1-vrd-10.1177_24741264241280597 – Supplemental material for Anti-VEGF Monotherapy vs Anti-VEGF and Steroid Combination Therapy for Diabetic Macular Edema: A Meta-analysis [file sj-zip-1-vrd-10.1177_24741264241280597.zip › Supplemental Figure 1. k.jpg]

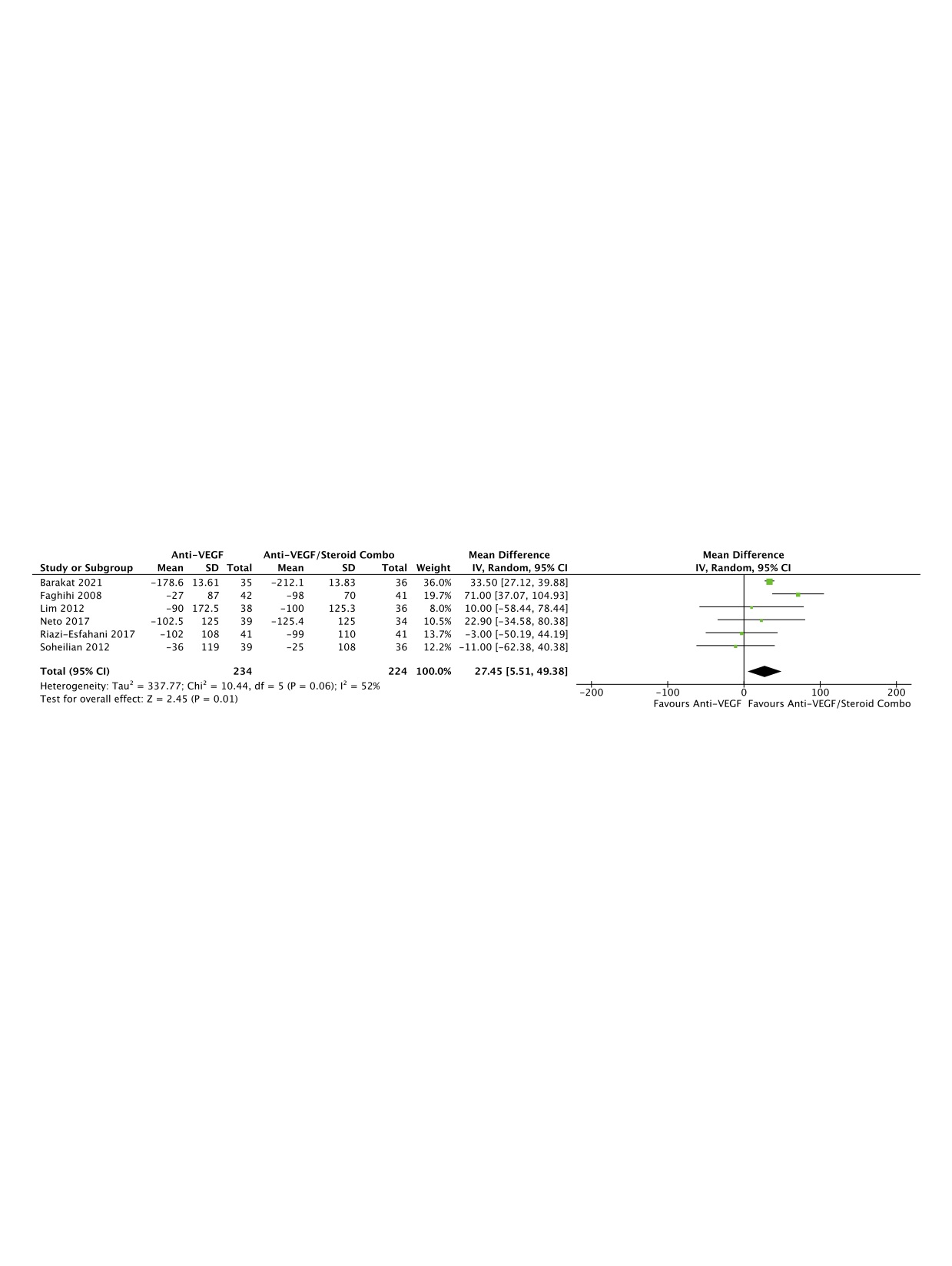

Supplement: sj-zip-1-vrd-10.1177_24741264241280597 – Supplemental material for Anti-VEGF Monotherapy vs Anti-VEGF and Steroid Combination Therapy for Diabetic Macular Edema: A Meta-analysis [file sj-zip-1-vrd-10.1177_24741264241280597.zip › Supplemental Figure 1. l.jpg]

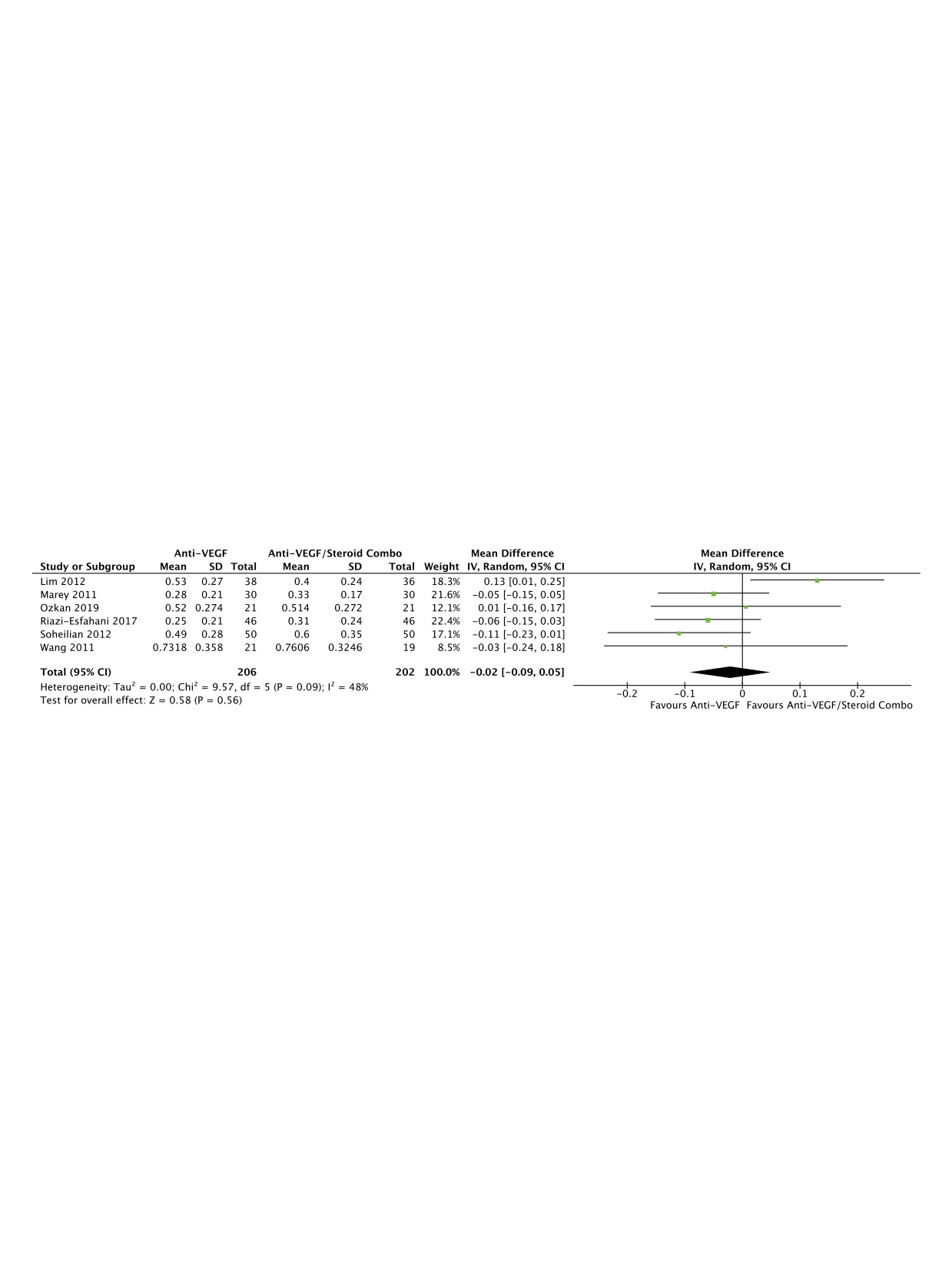

Supplement: sj-zip-1-vrd-10.1177_24741264241280597 – Supplemental material for Anti-VEGF Monotherapy vs Anti-VEGF and Steroid Combination Therapy for Diabetic Macular Edema: A Meta-analysis [file sj-zip-1-vrd-10.1177_24741264241280597.zip › Supplemental Figure 1. i.jpg]

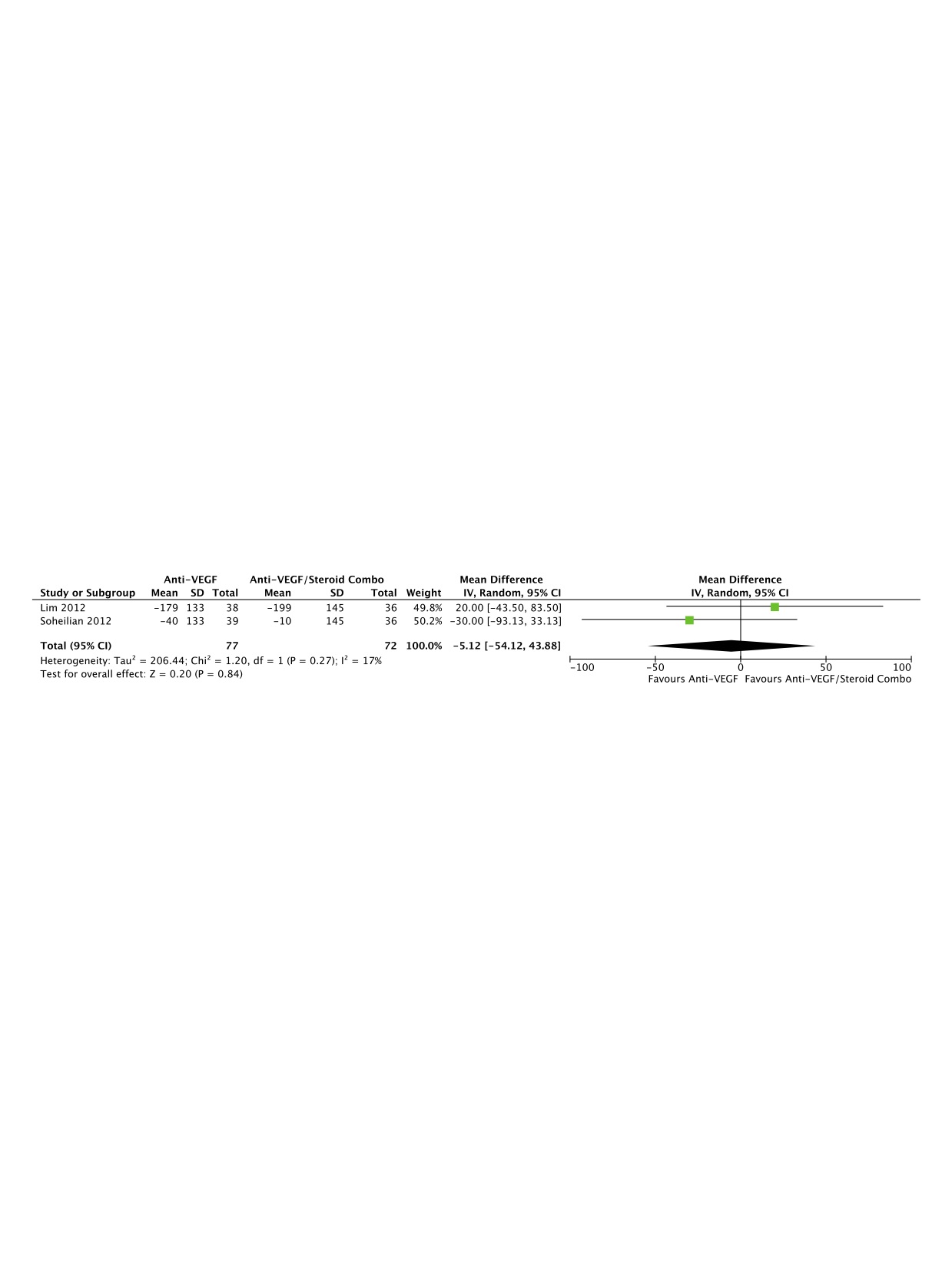

Supplement: sj-zip-1-vrd-10.1177_24741264241280597 – Supplemental material for Anti-VEGF Monotherapy vs Anti-VEGF and Steroid Combination Therapy for Diabetic Macular Edema: A Meta-analysis [file sj-zip-1-vrd-10.1177_24741264241280597.zip › Supplemental Figure 1. n.jpg]

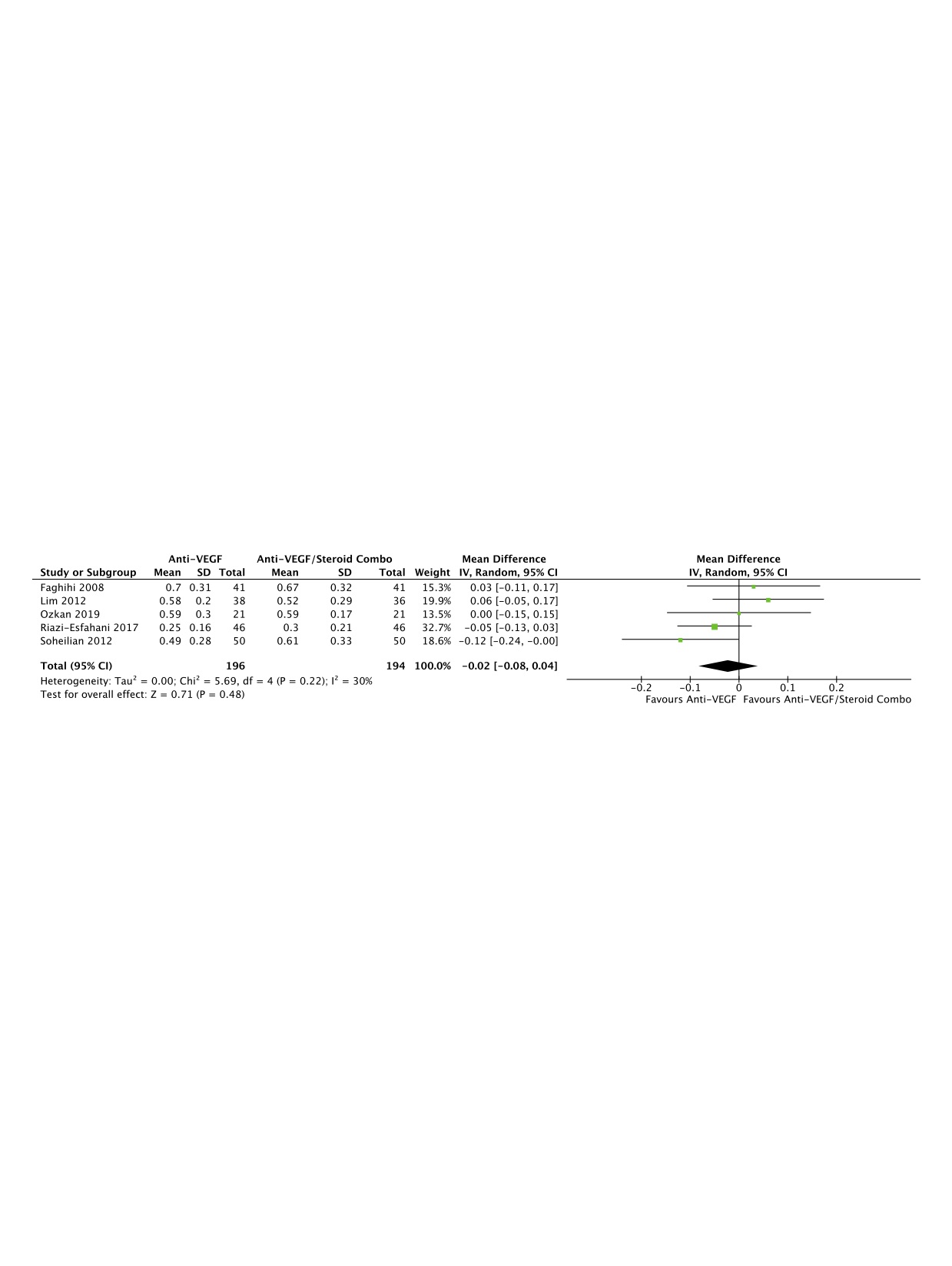

Supplement: sj-zip-1-vrd-10.1177_24741264241280597 – Supplemental material for Anti-VEGF Monotherapy vs Anti-VEGF and Steroid Combination Therapy for Diabetic Macular Edema: A Meta-analysis [file sj-zip-1-vrd-10.1177_24741264241280597.zip › Supplemental Figure 1. j.jpg]

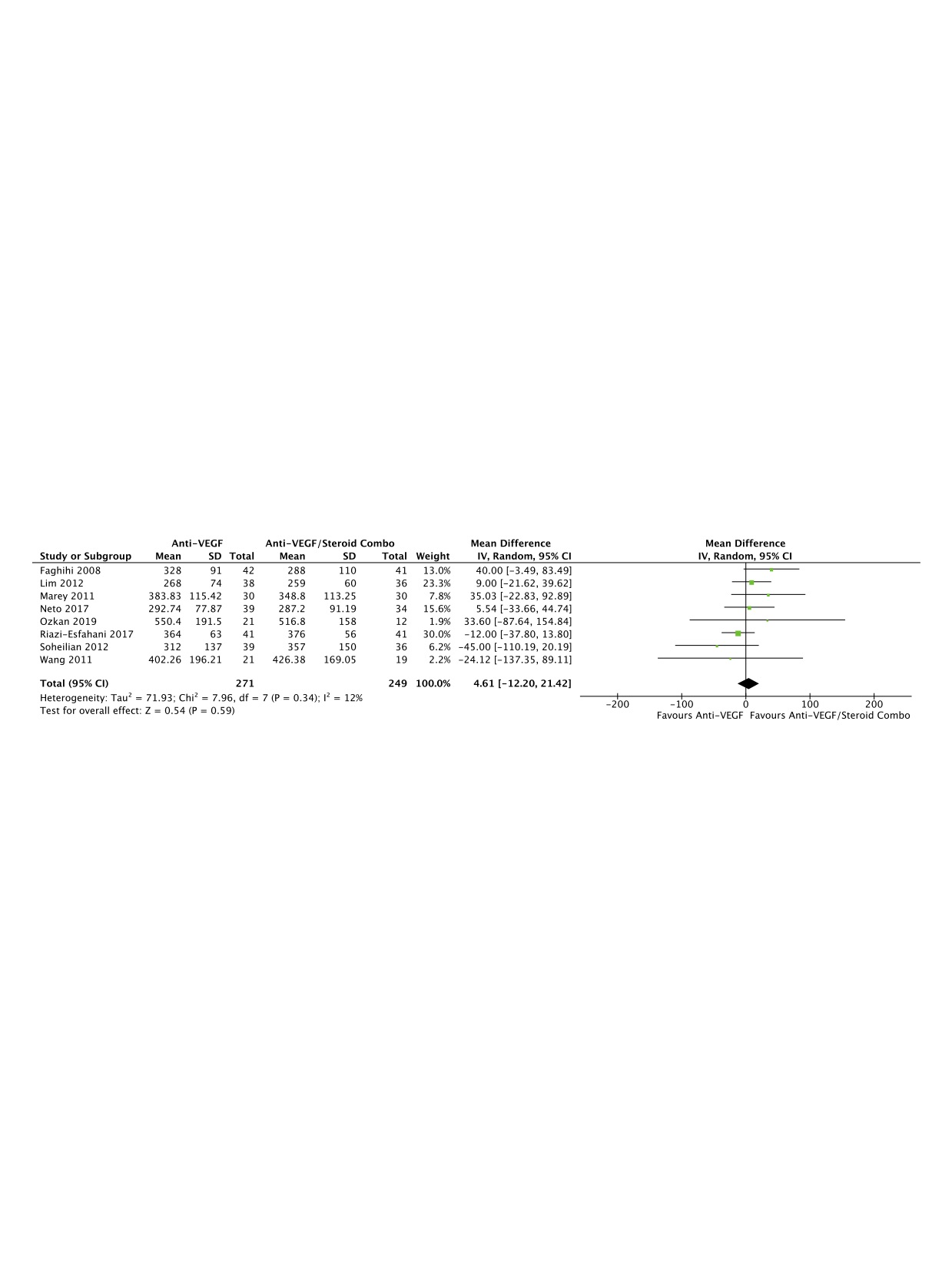

Supplement: sj-zip-1-vrd-10.1177_24741264241280597 – Supplemental material for Anti-VEGF Monotherapy vs Anti-VEGF and Steroid Combination Therapy for Diabetic Macular Edema: A Meta-analysis [file sj-zip-1-vrd-10.1177_24741264241280597.zip › Supplemental Figure 1. o.jpg]

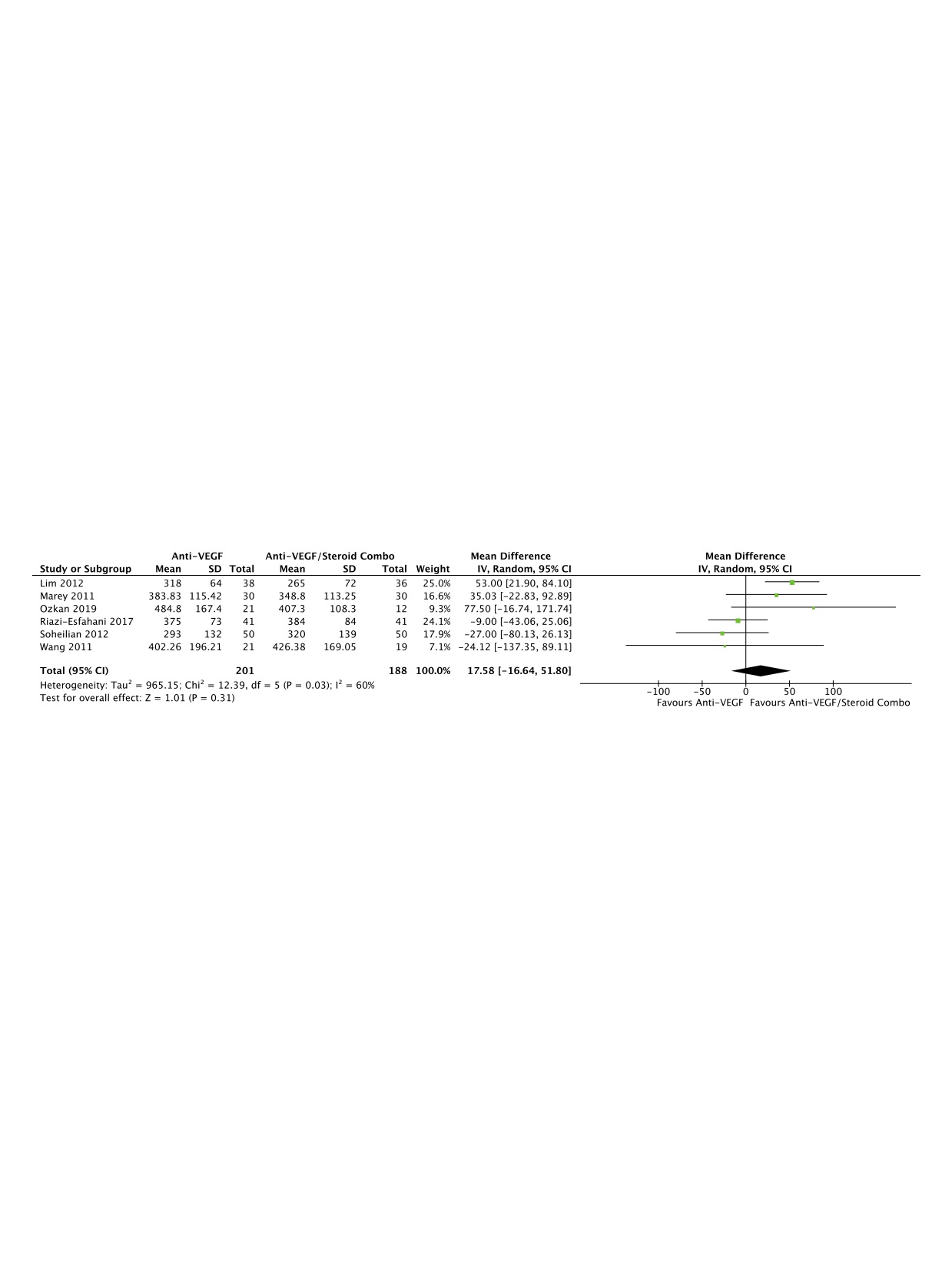

Supplement: sj-zip-1-vrd-10.1177_24741264241280597 – Supplemental material for Anti-VEGF Monotherapy vs Anti-VEGF and Steroid Combination Therapy for Diabetic Macular Edema: A Meta-analysis [file sj-zip-1-vrd-10.1177_24741264241280597.zip › Supplemental Figure 1. p.jpg]

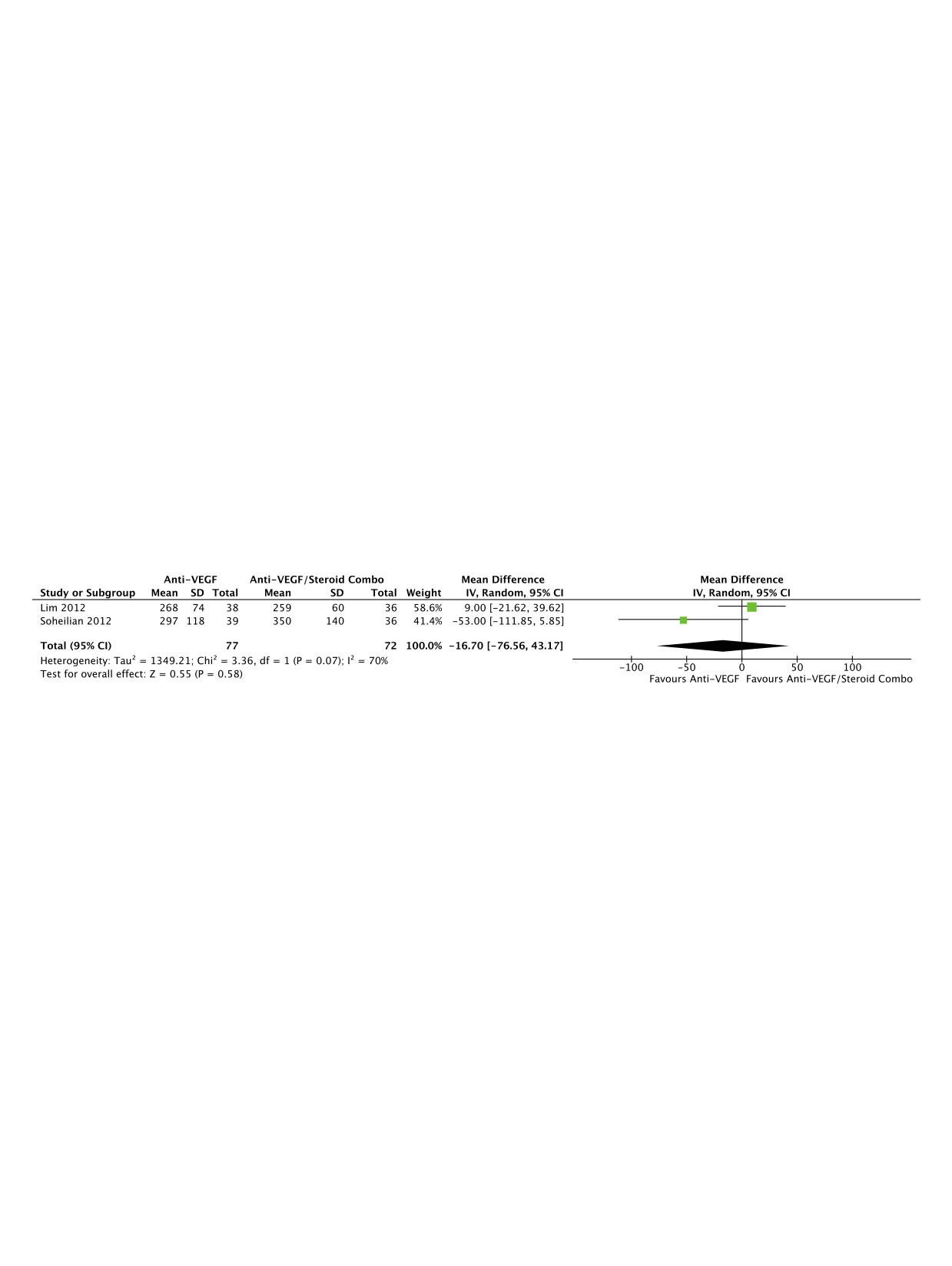

Supplement: sj-zip-1-vrd-10.1177_24741264241280597 – Supplemental material for Anti-VEGF Monotherapy vs Anti-VEGF and Steroid Combination Therapy for Diabetic Macular Edema: A Meta-analysis [file sj-zip-1-vrd-10.1177_24741264241280597.zip › Supplemental Figure 1. q.jpg]

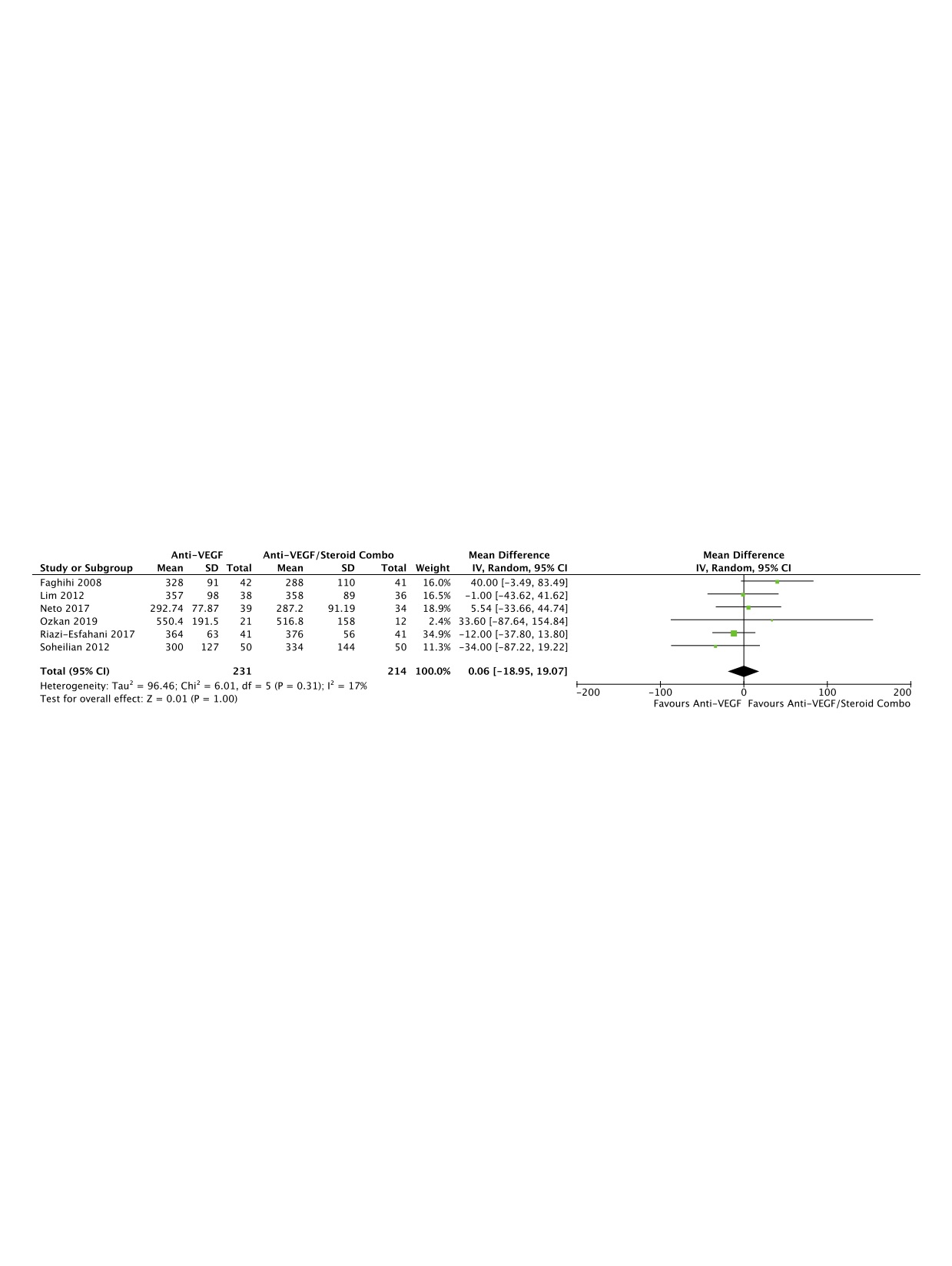

Supplement: sj-zip-1-vrd-10.1177_24741264241280597 – Supplemental material for Anti-VEGF Monotherapy vs Anti-VEGF and Steroid Combination Therapy for Diabetic Macular Edema: A Meta-analysis [file sj-zip-1-vrd-10.1177_24741264241280597.zip › Supplemental Figure 1. r.jpg]

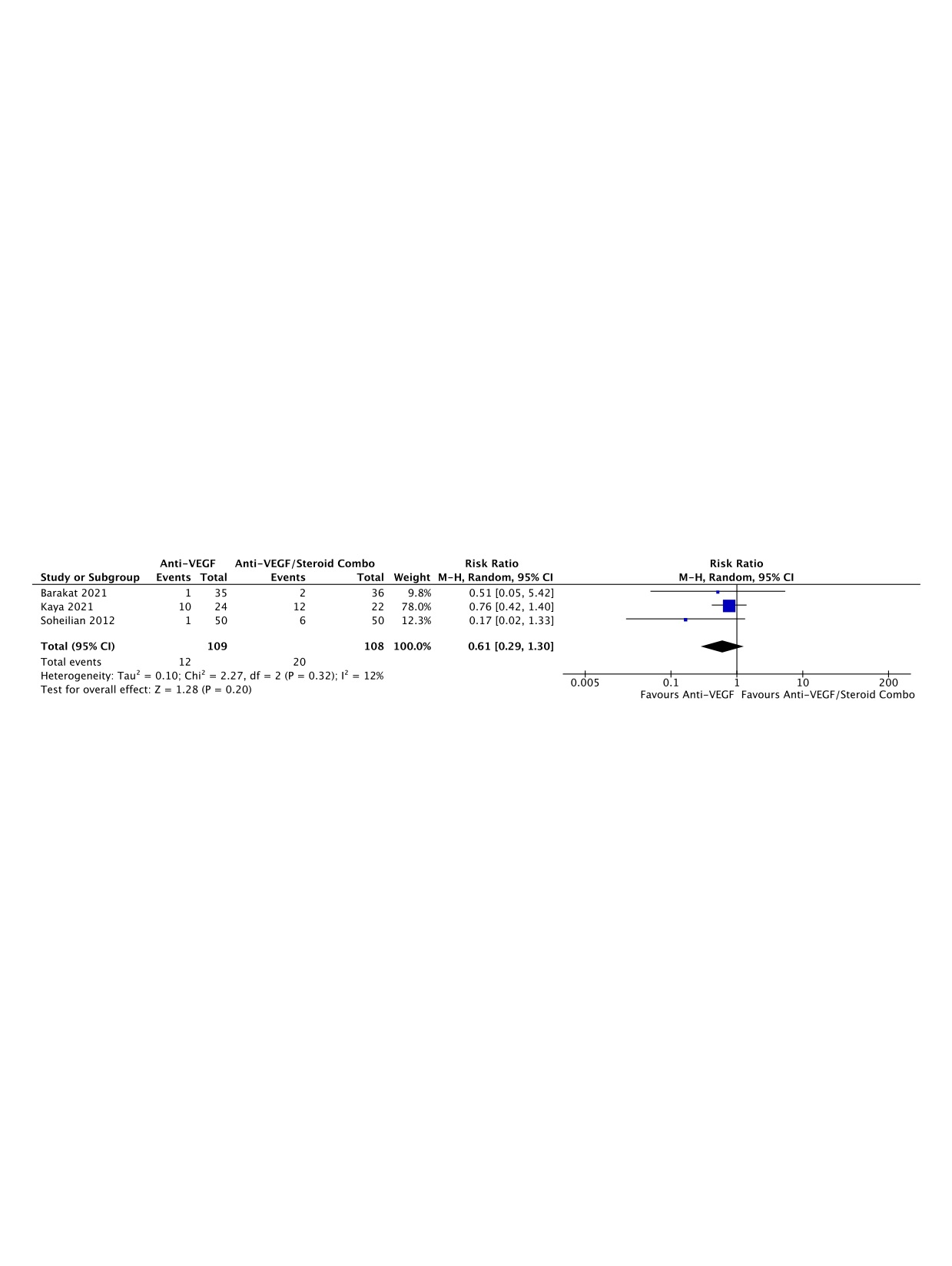

Supplement: sj-zip-1-vrd-10.1177_24741264241280597 – Supplemental material for Anti-VEGF Monotherapy vs Anti-VEGF and Steroid Combination Therapy for Diabetic Macular Edema: A Meta-analysis [file sj-zip-1-vrd-10.1177_24741264241280597.zip › Supplemental Figure 1. t.jpg]

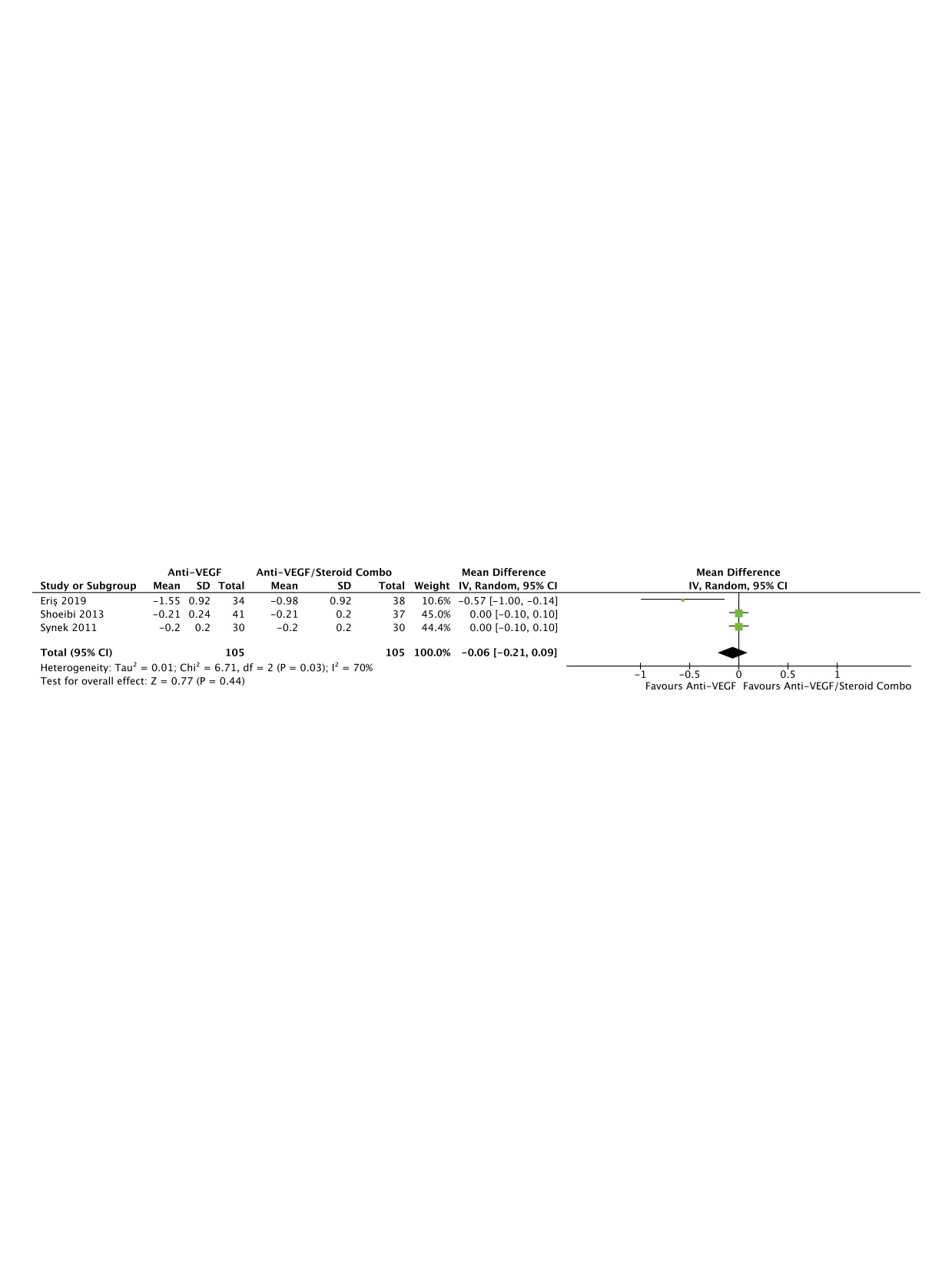

Supplement: sj-zip-1-vrd-10.1177_24741264241280597 – Supplemental material for Anti-VEGF Monotherapy vs Anti-VEGF and Steroid Combination Therapy for Diabetic Macular Edema: A Meta-analysis [file sj-zip-1-vrd-10.1177_24741264241280597.zip › Supplemental Figure 2. b.jpg]

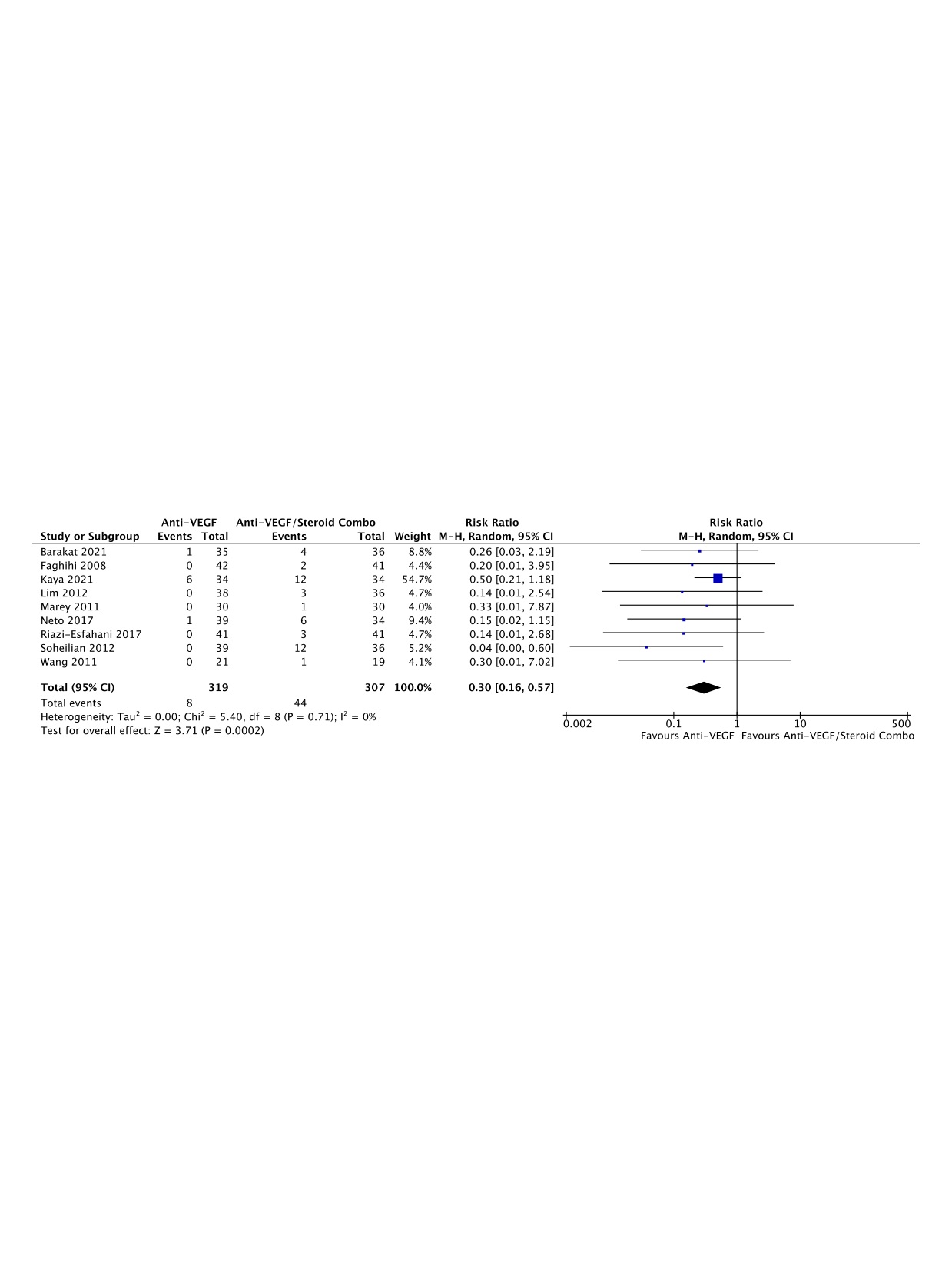

Supplement: sj-zip-1-vrd-10.1177_24741264241280597 – Supplemental material for Anti-VEGF Monotherapy vs Anti-VEGF and Steroid Combination Therapy for Diabetic Macular Edema: A Meta-analysis [file sj-zip-1-vrd-10.1177_24741264241280597.zip › Supplemental Figure 1. s.jpg]

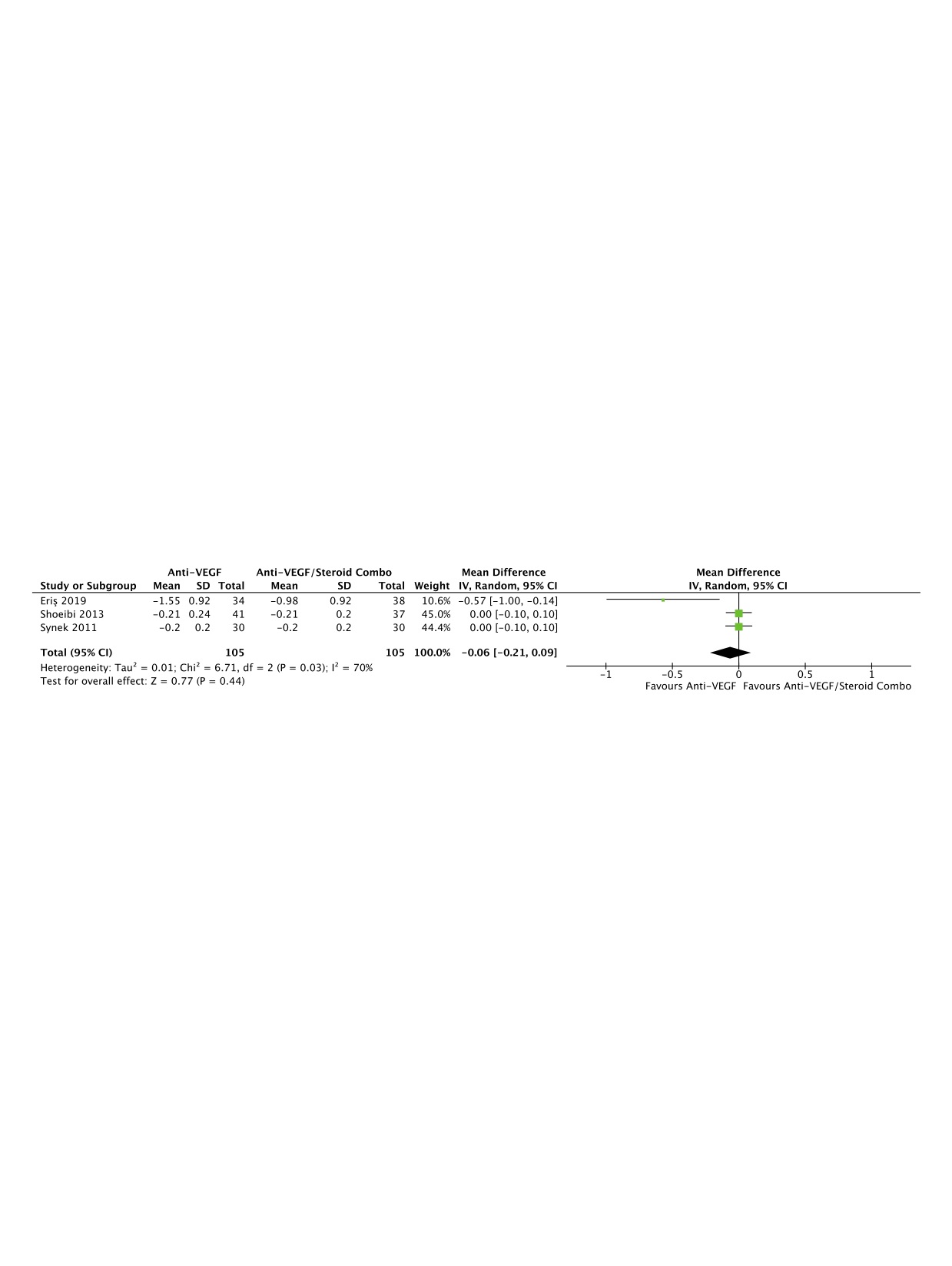

Supplement: sj-zip-1-vrd-10.1177_24741264241280597 – Supplemental material for Anti-VEGF Monotherapy vs Anti-VEGF and Steroid Combination Therapy for Diabetic Macular Edema: A Meta-analysis [file sj-zip-1-vrd-10.1177_24741264241280597.zip › Supplemental Figure 2. d.jpg]

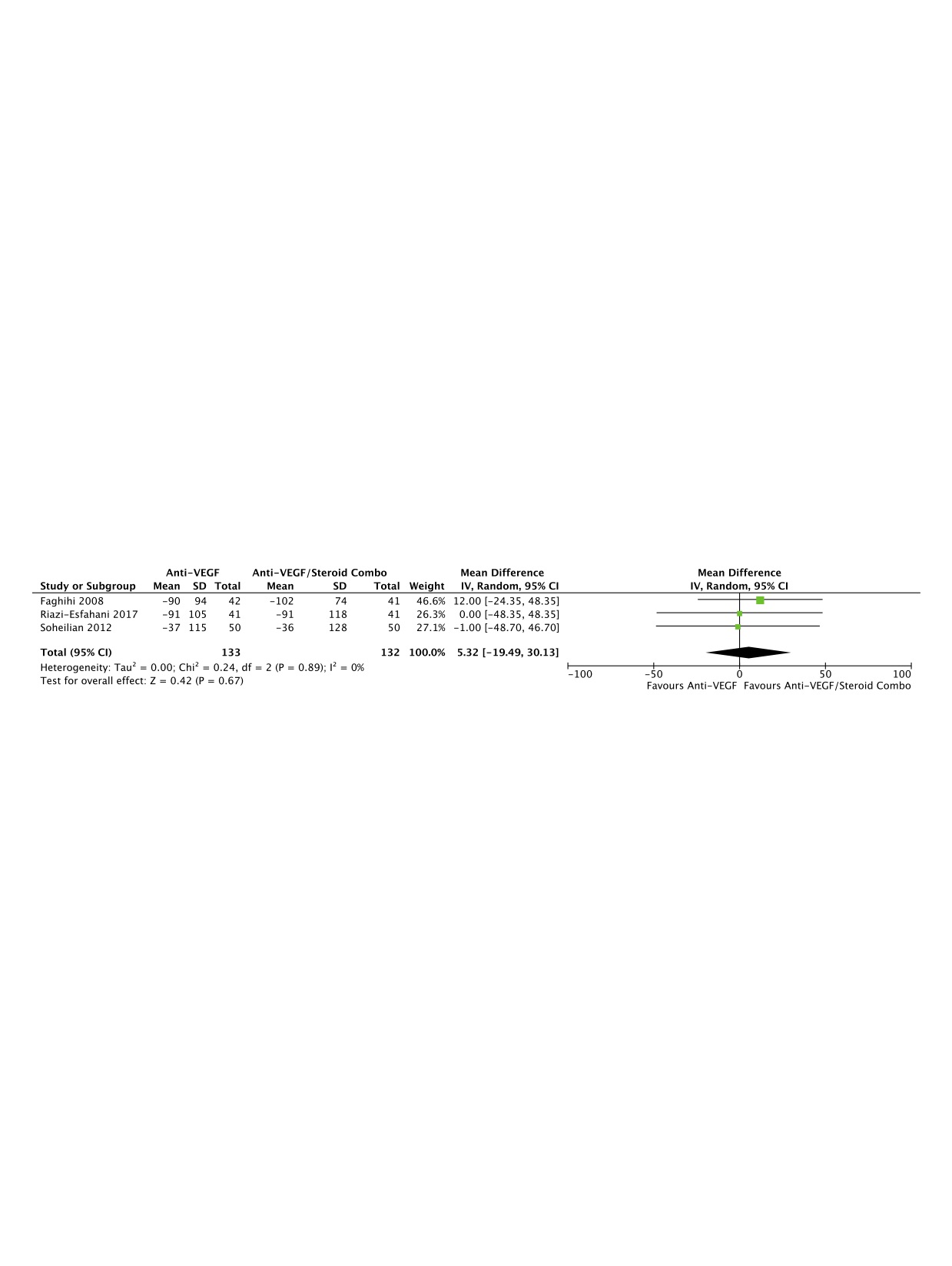

Supplement: sj-zip-1-vrd-10.1177_24741264241280597 – Supplemental material for Anti-VEGF Monotherapy vs Anti-VEGF and Steroid Combination Therapy for Diabetic Macular Edema: A Meta-analysis [file sj-zip-1-vrd-10.1177_24741264241280597.zip › Supplemental Figure 1. m.jpg]

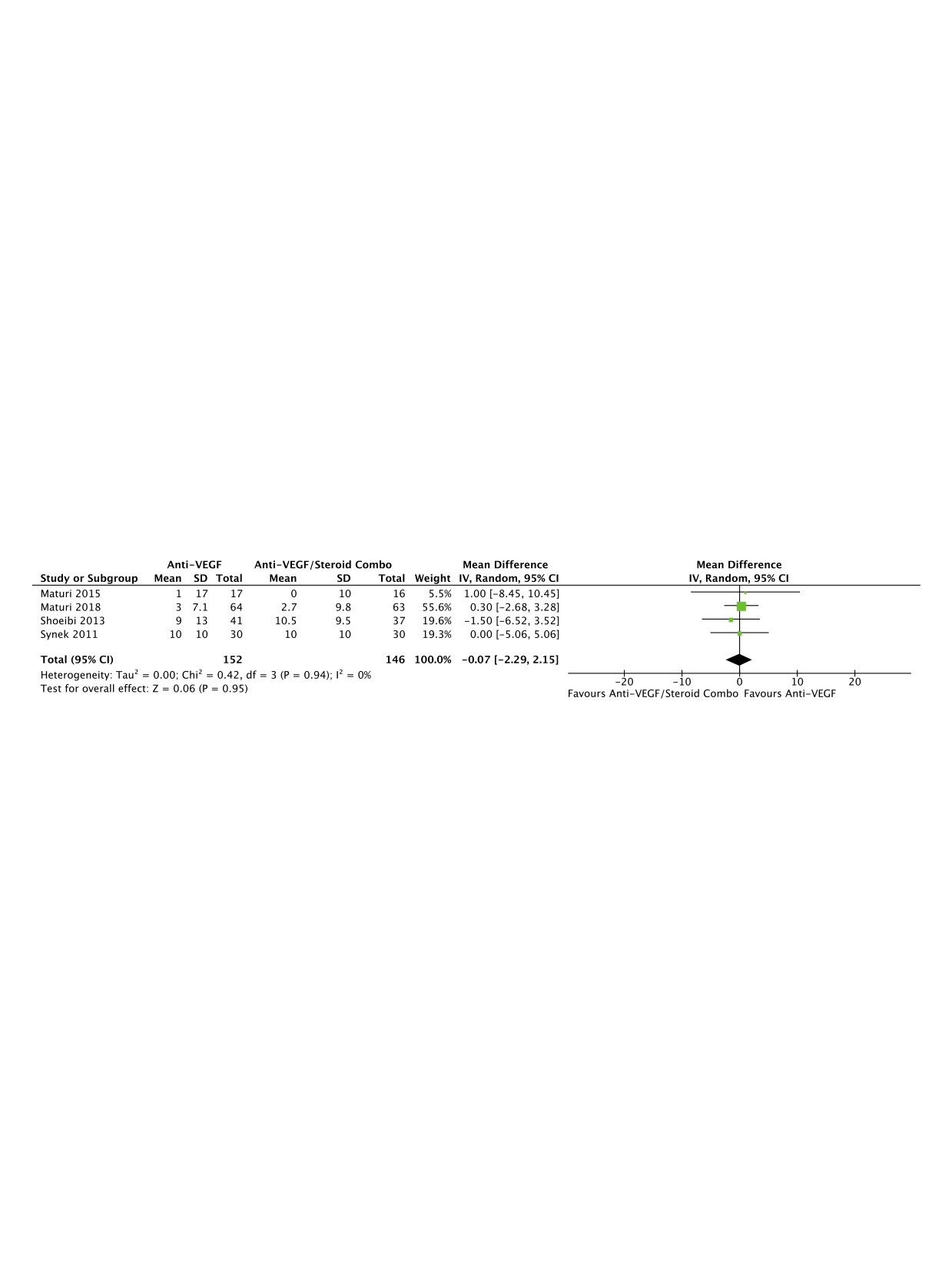

Supplement: sj-zip-1-vrd-10.1177_24741264241280597 – Supplemental material for Anti-VEGF Monotherapy vs Anti-VEGF and Steroid Combination Therapy for Diabetic Macular Edema: A Meta-analysis [file sj-zip-1-vrd-10.1177_24741264241280597.zip › Supplemental Figure 2. a.jpg]

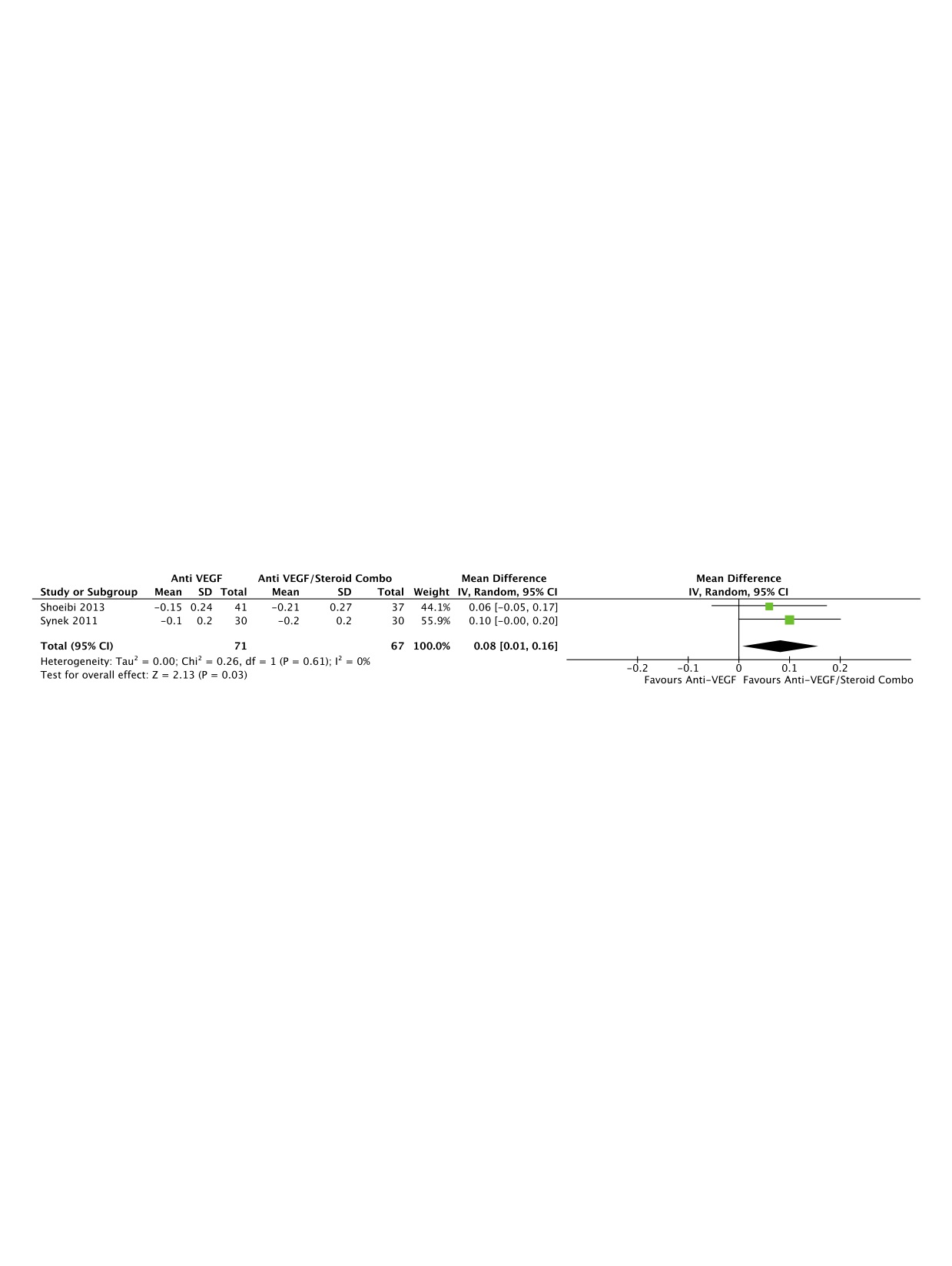

Supplement: sj-zip-1-vrd-10.1177_24741264241280597 – Supplemental material for Anti-VEGF Monotherapy vs Anti-VEGF and Steroid Combination Therapy for Diabetic Macular Edema: A Meta-analysis [file sj-zip-1-vrd-10.1177_24741264241280597.zip › Supplemental Figure 2. e.jpg]

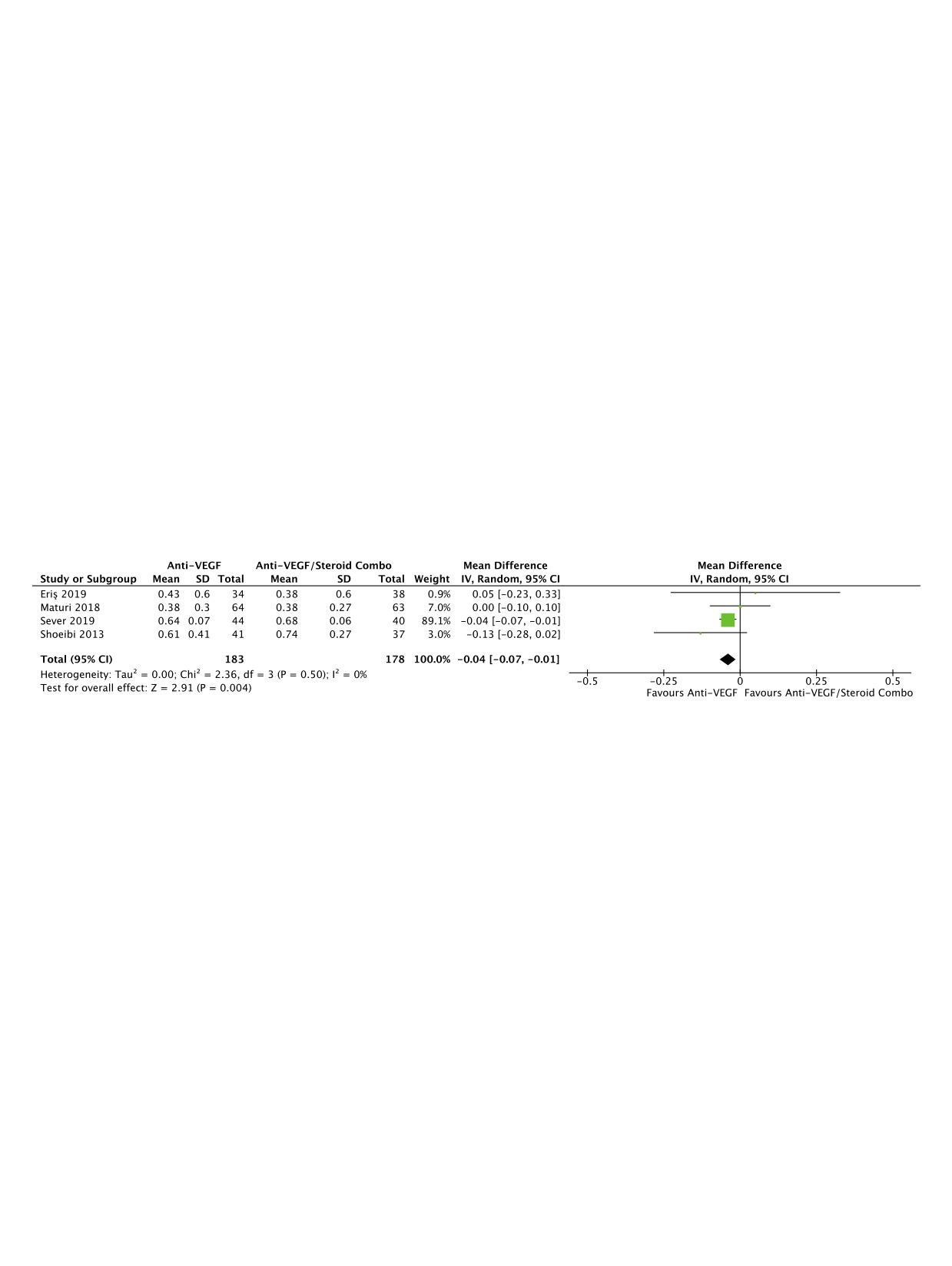

Supplement: sj-zip-1-vrd-10.1177_24741264241280597 – Supplemental material for Anti-VEGF Monotherapy vs Anti-VEGF and Steroid Combination Therapy for Diabetic Macular Edema: A Meta-analysis [file sj-zip-1-vrd-10.1177_24741264241280597.zip › Supplemental Figure 2. f.jpg]

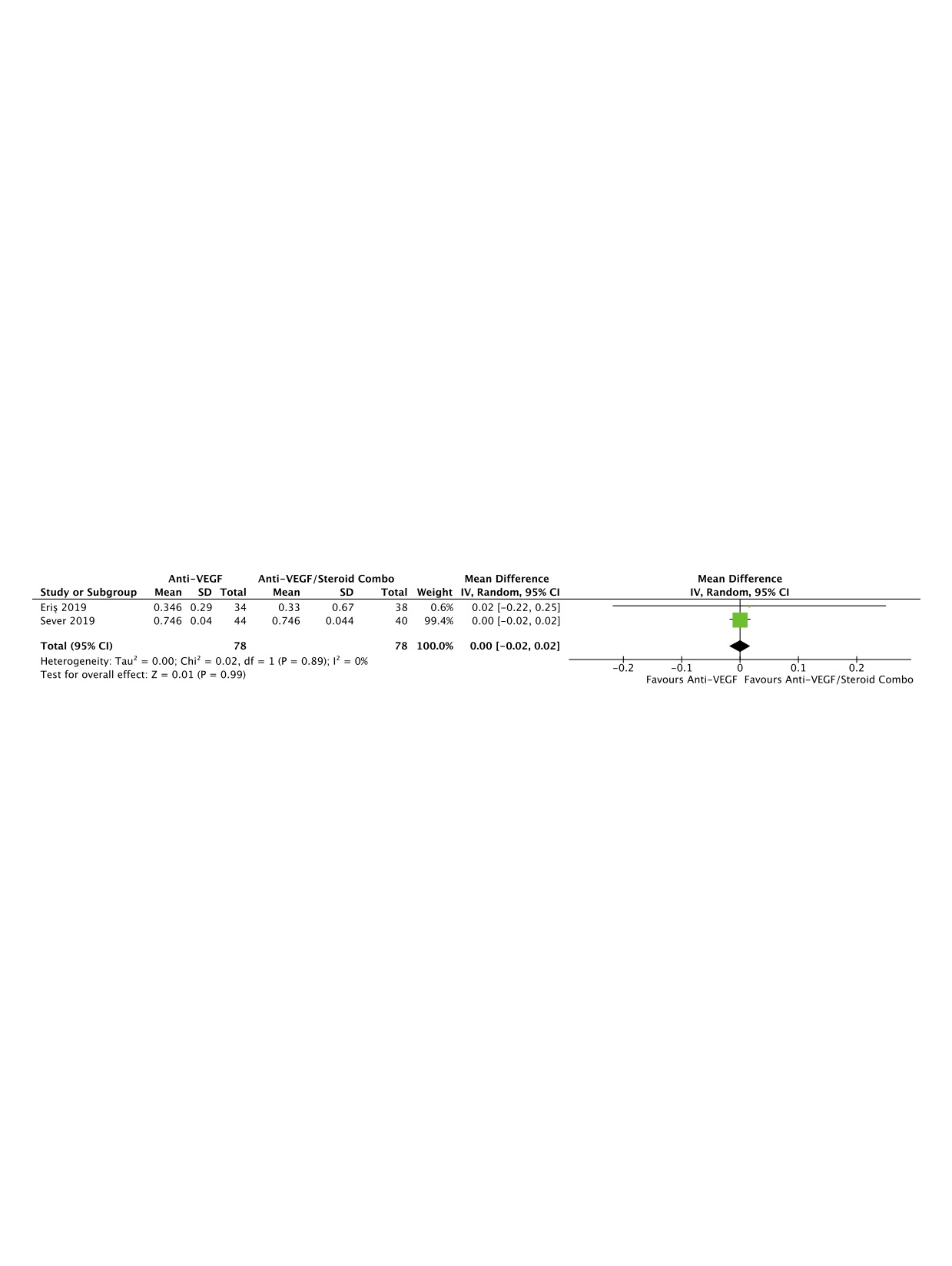

Supplement: sj-zip-1-vrd-10.1177_24741264241280597 – Supplemental material for Anti-VEGF Monotherapy vs Anti-VEGF and Steroid Combination Therapy for Diabetic Macular Edema: A Meta-analysis [file sj-zip-1-vrd-10.1177_24741264241280597.zip › Supplemental Figure 2. g.jpg]

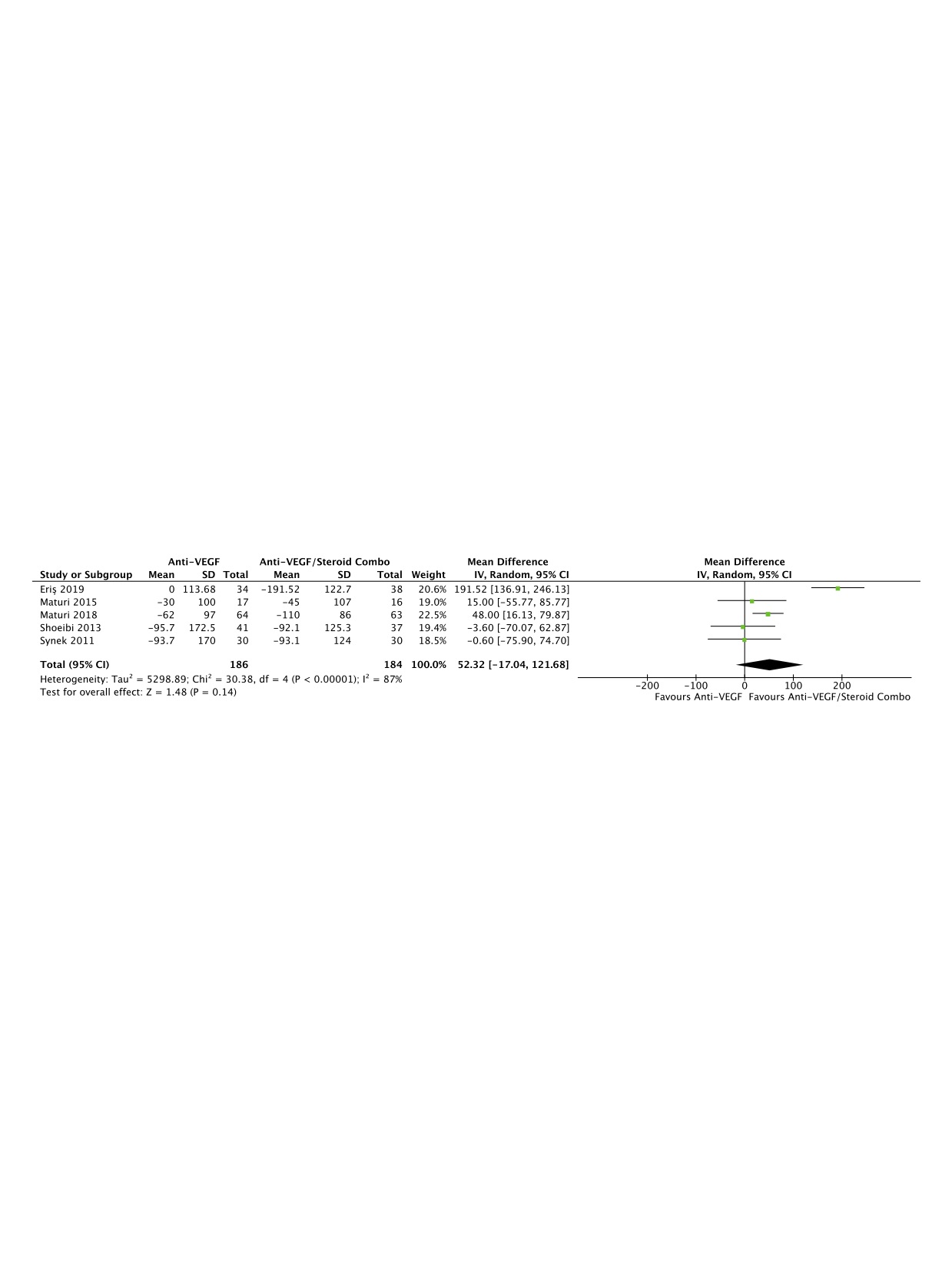

Supplement: sj-zip-1-vrd-10.1177_24741264241280597 – Supplemental material for Anti-VEGF Monotherapy vs Anti-VEGF and Steroid Combination Therapy for Diabetic Macular Edema: A Meta-analysis [file sj-zip-1-vrd-10.1177_24741264241280597.zip › Supplemental Figure 2. i.jpg]

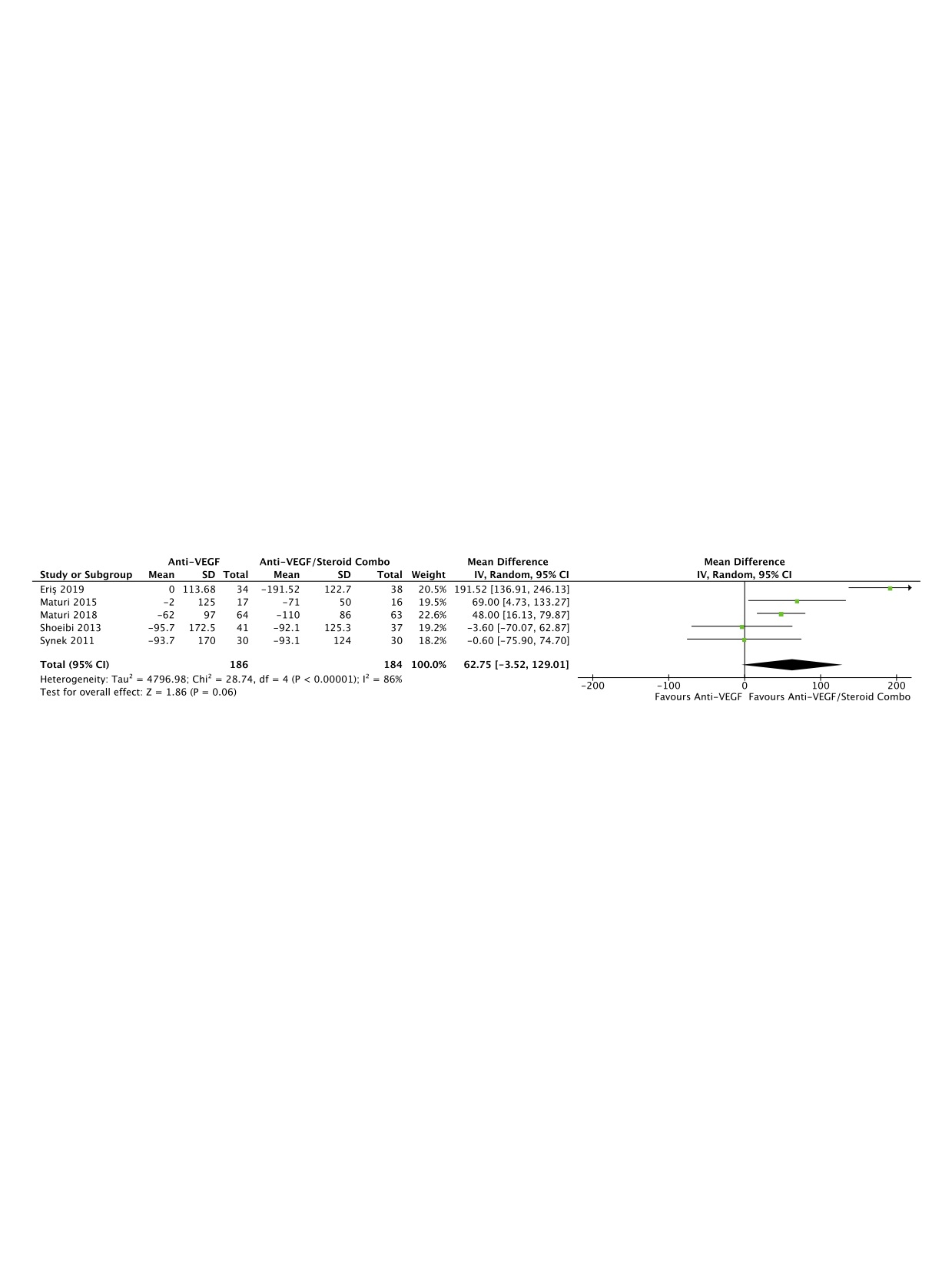

Supplement: sj-zip-1-vrd-10.1177_24741264241280597 – Supplemental material for Anti-VEGF Monotherapy vs Anti-VEGF and Steroid Combination Therapy for Diabetic Macular Edema: A Meta-analysis [file sj-zip-1-vrd-10.1177_24741264241280597.zip › Supplemental Figure 2. j.jpg]

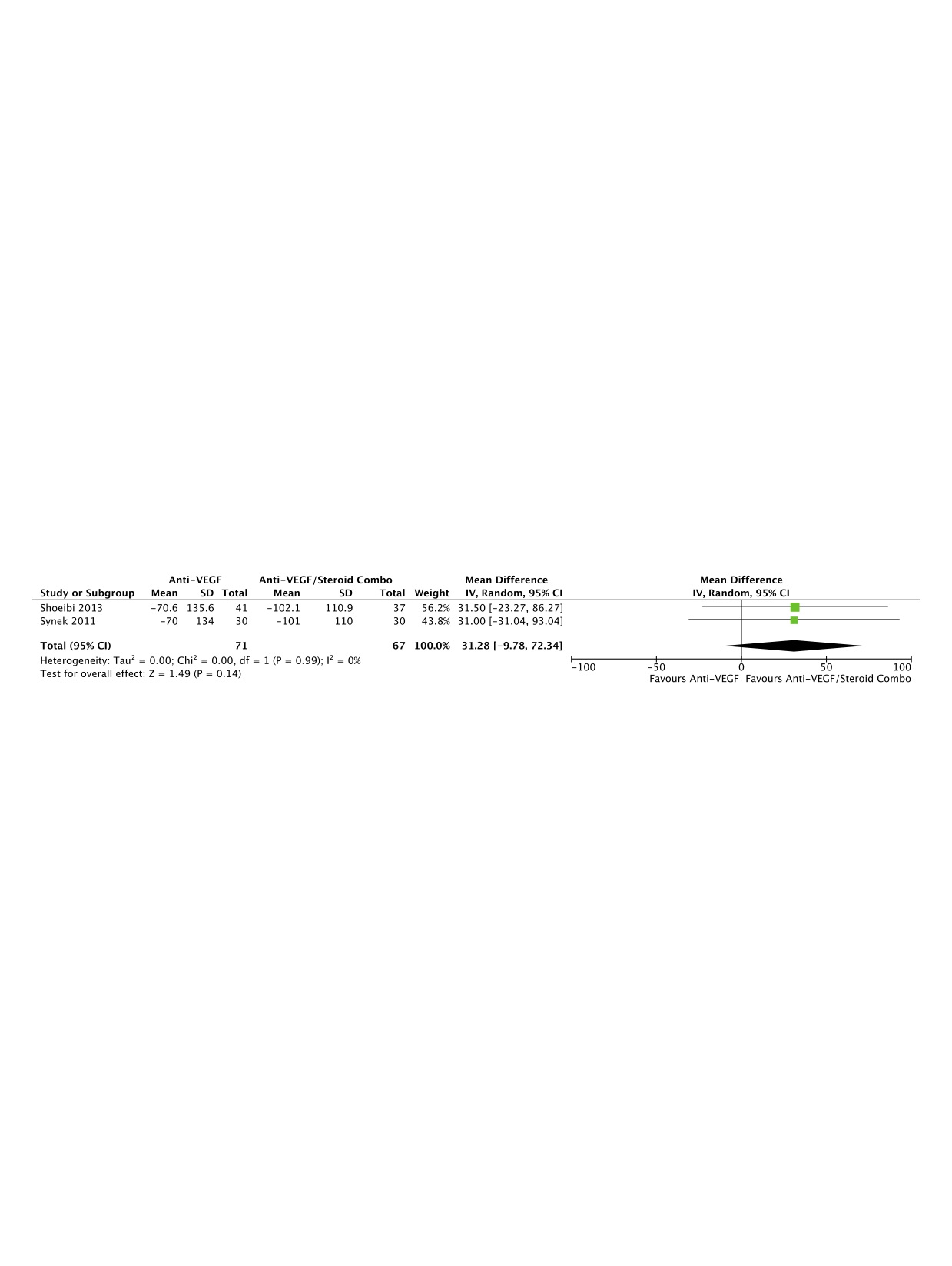

Supplement: sj-zip-1-vrd-10.1177_24741264241280597 – Supplemental material for Anti-VEGF Monotherapy vs Anti-VEGF and Steroid Combination Therapy for Diabetic Macular Edema: A Meta-analysis [file sj-zip-1-vrd-10.1177_24741264241280597.zip › Supplemental Figure 2. k.jpg]

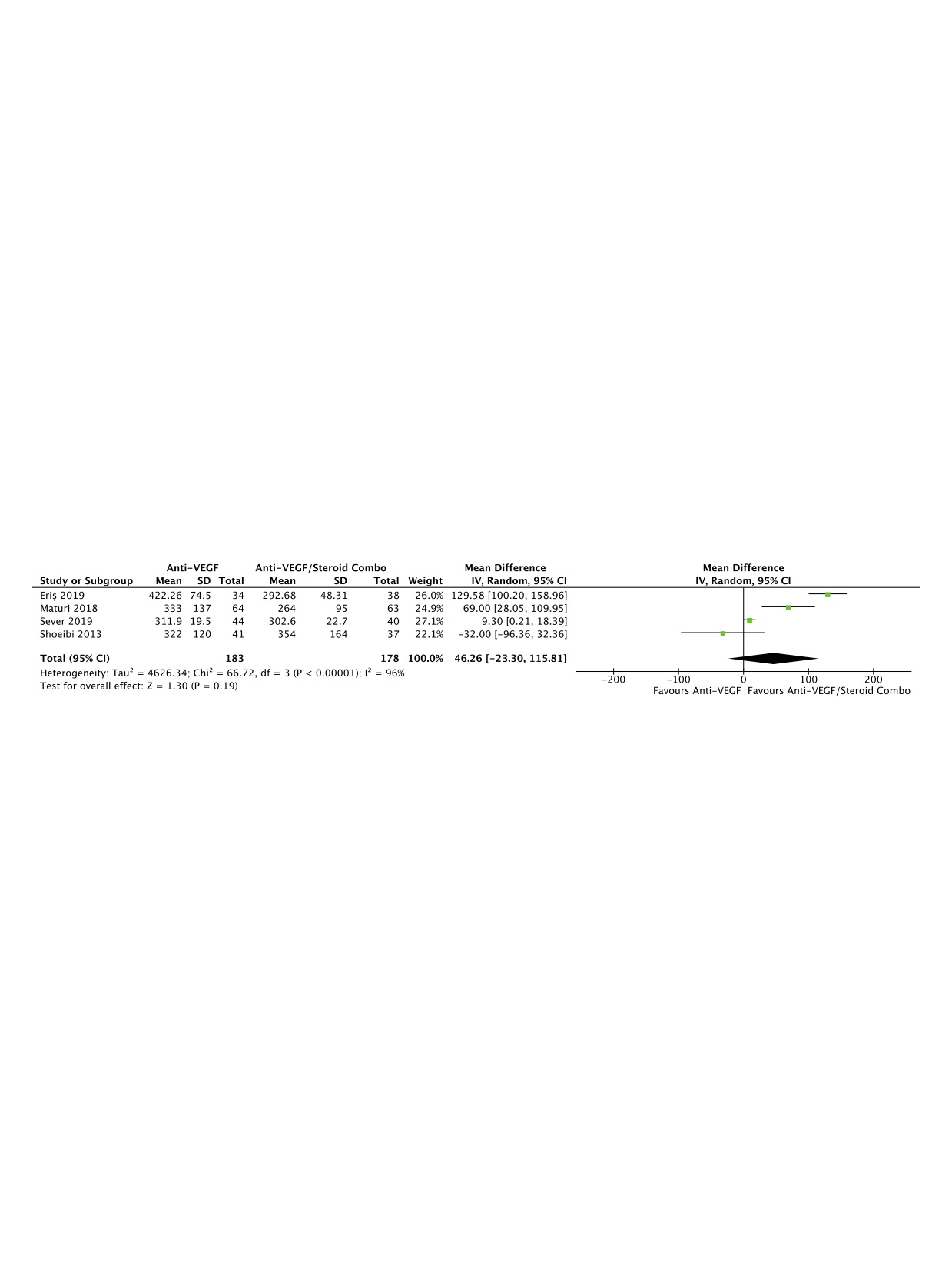

Supplement: sj-zip-1-vrd-10.1177_24741264241280597 – Supplemental material for Anti-VEGF Monotherapy vs Anti-VEGF and Steroid Combination Therapy for Diabetic Macular Edema: A Meta-analysis [file sj-zip-1-vrd-10.1177_24741264241280597.zip › Supplemental Figure 2. l.jpg]

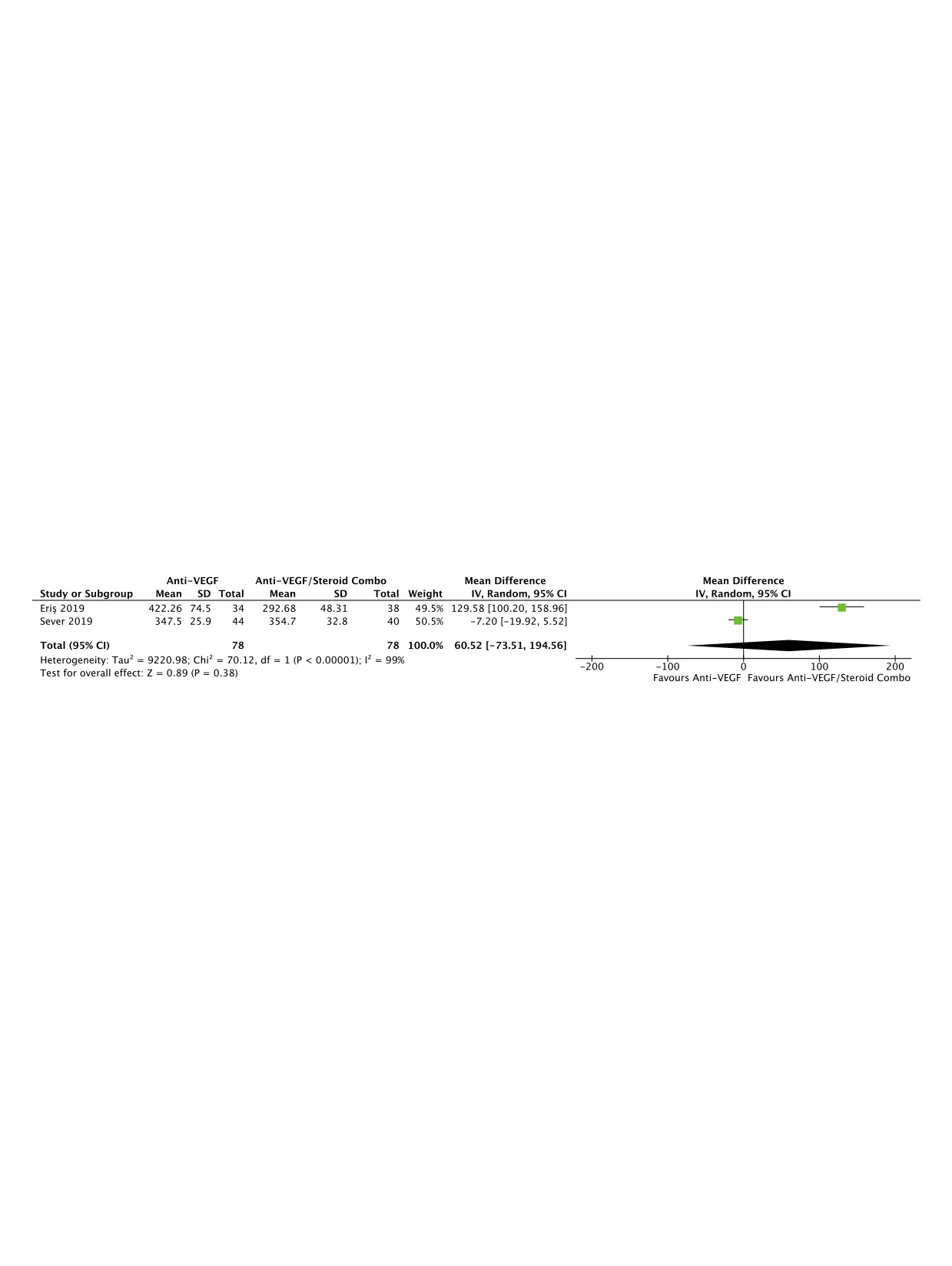

Supplement: sj-zip-1-vrd-10.1177_24741264241280597 – Supplemental material for Anti-VEGF Monotherapy vs Anti-VEGF and Steroid Combination Therapy for Diabetic Macular Edema: A Meta-analysis [file sj-zip-1-vrd-10.1177_24741264241280597.zip › Supplemental Figure 2. m.jpg]

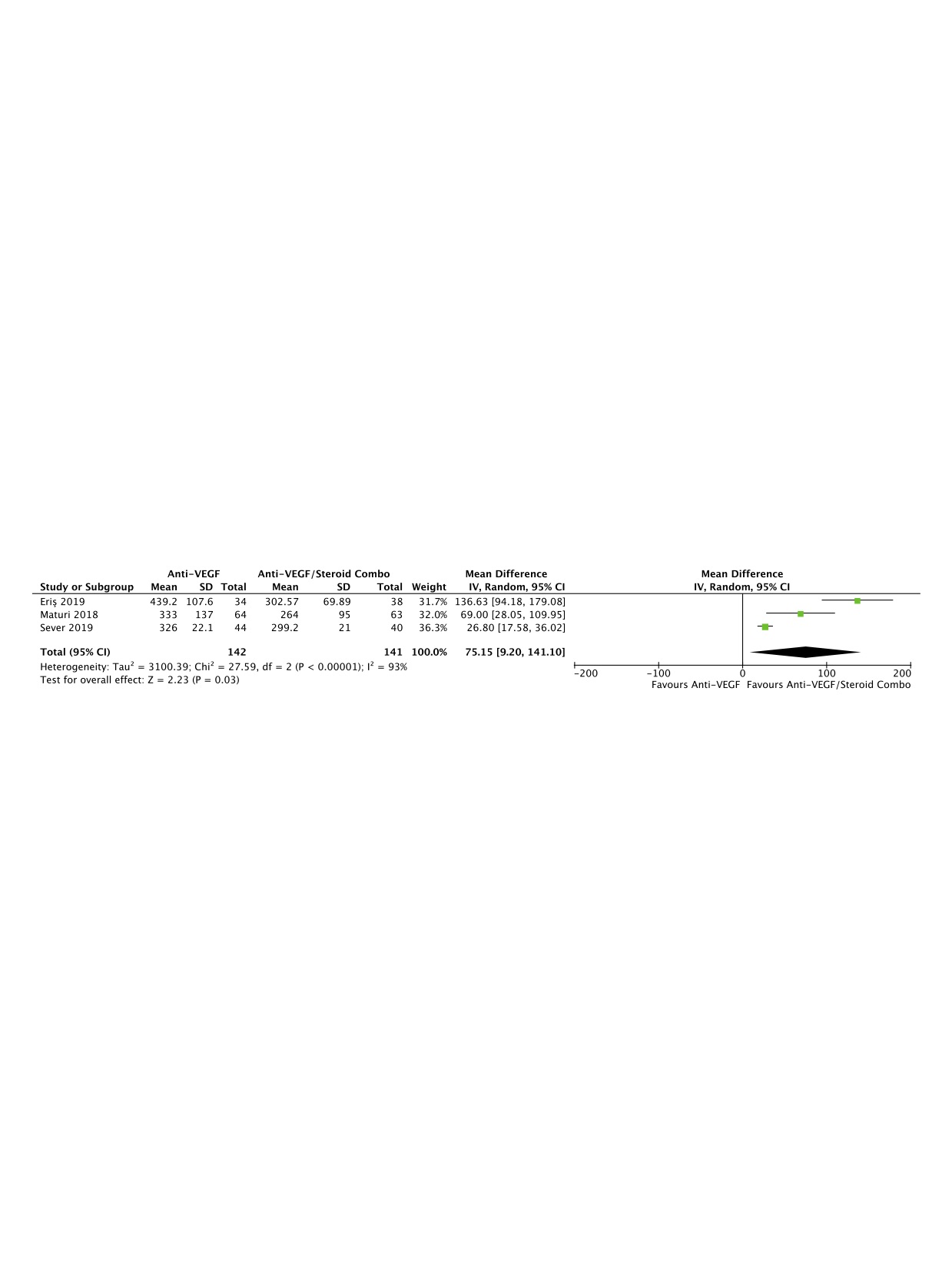

Supplement: sj-zip-1-vrd-10.1177_24741264241280597 – Supplemental material for Anti-VEGF Monotherapy vs Anti-VEGF and Steroid Combination Therapy for Diabetic Macular Edema: A Meta-analysis [file sj-zip-1-vrd-10.1177_24741264241280597.zip › Supplemental Figure 2. n.jpg]

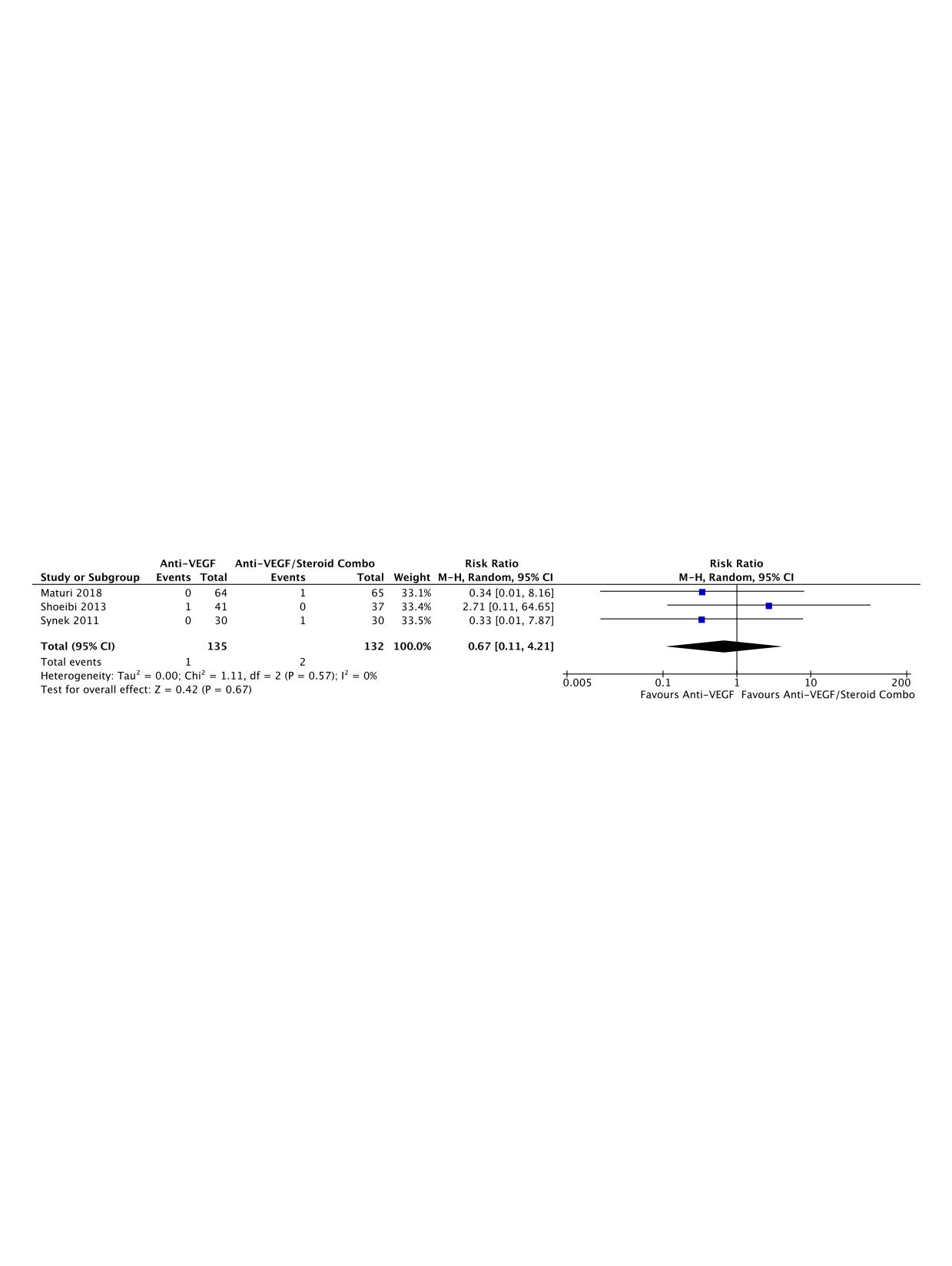

Supplement: sj-zip-1-vrd-10.1177_24741264241280597 – Supplemental material for Anti-VEGF Monotherapy vs Anti-VEGF and Steroid Combination Therapy for Diabetic Macular Edema: A Meta-analysis [file sj-zip-1-vrd-10.1177_24741264241280597.zip › Supplemental Figure 2. p.jpg]

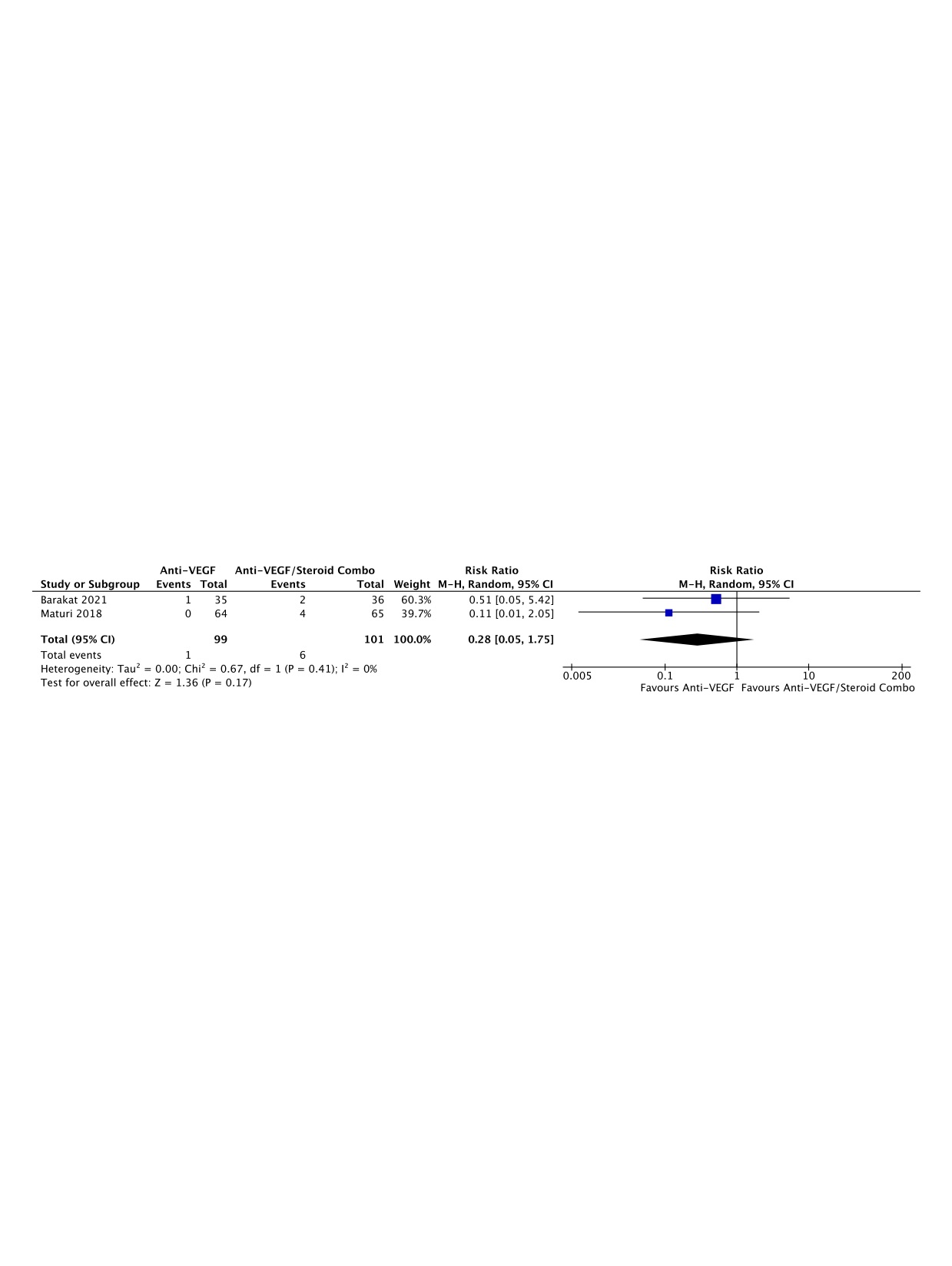

Supplement: sj-zip-1-vrd-10.1177_24741264241280597 – Supplemental material for Anti-VEGF Monotherapy vs Anti-VEGF and Steroid Combination Therapy for Diabetic Macular Edema: A Meta-analysis [file sj-zip-1-vrd-10.1177_24741264241280597.zip › Supplemental Figure 2. q.jpg]

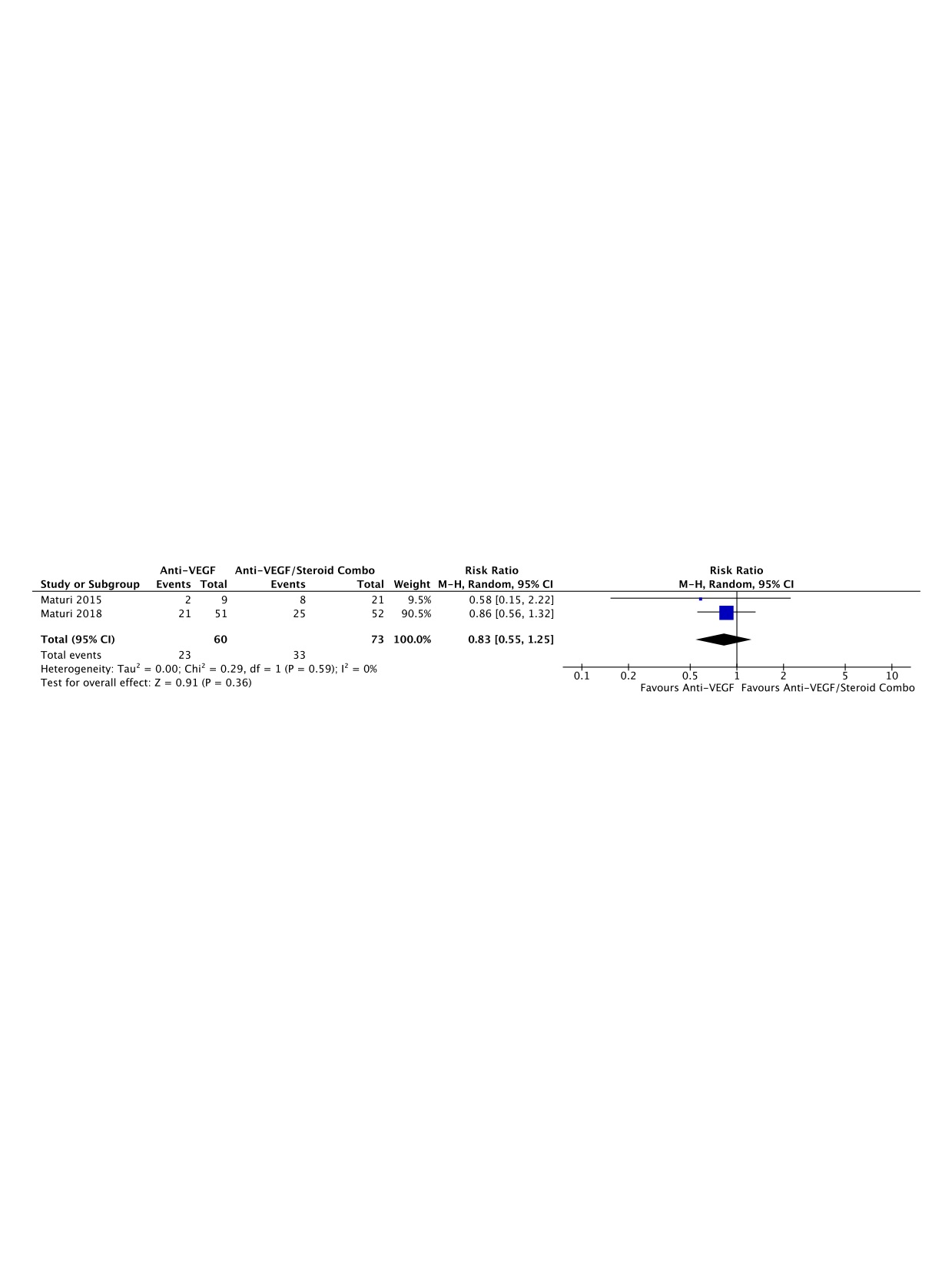

Supplement: sj-zip-1-vrd-10.1177_24741264241280597 – Supplemental material for Anti-VEGF Monotherapy vs Anti-VEGF and Steroid Combination Therapy for Diabetic Macular Edema: A Meta-analysis [file sj-zip-1-vrd-10.1177_24741264241280597.zip › Supplemental Figure 2. r.jpg]

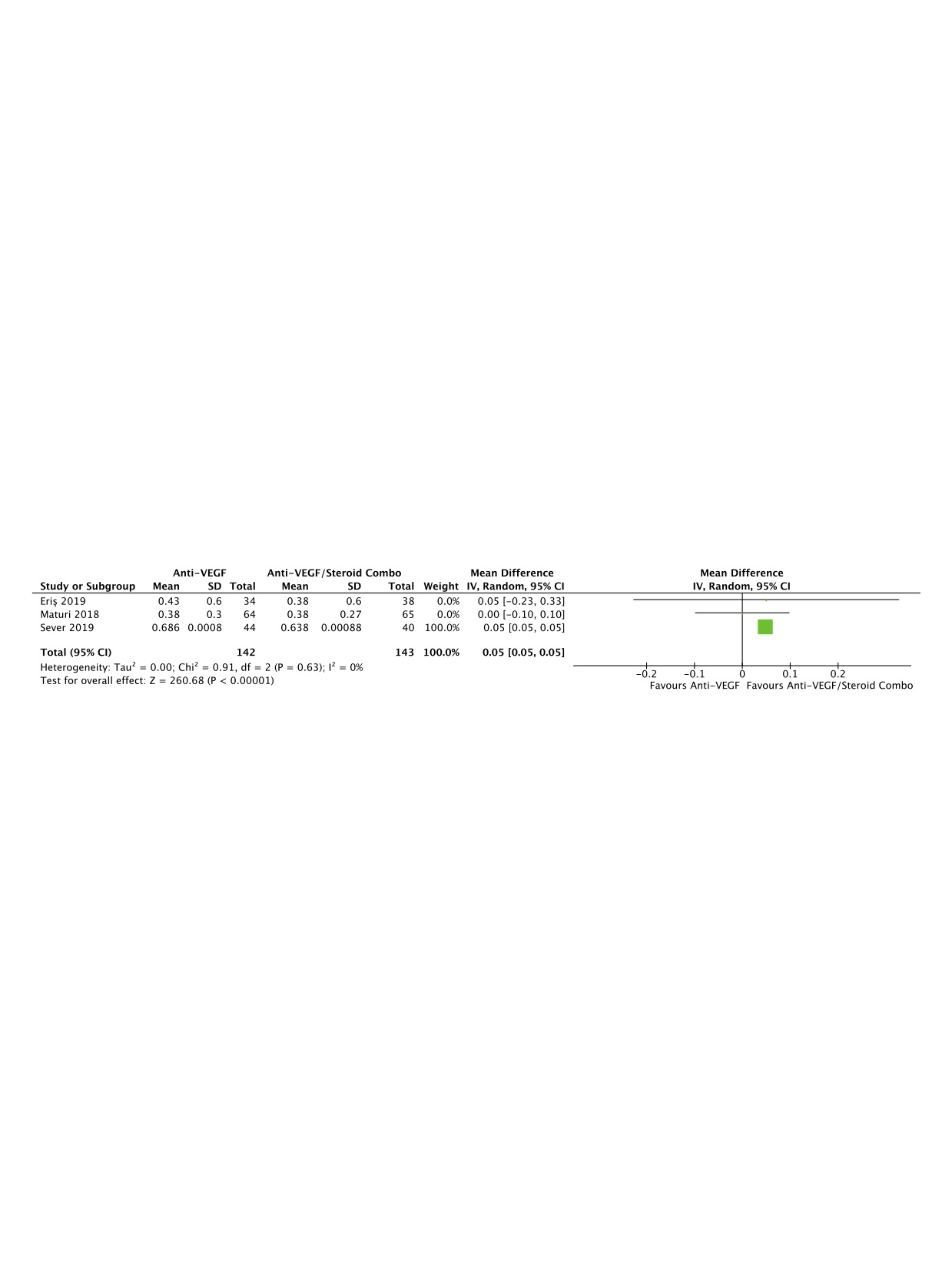

Supplement: sj-zip-1-vrd-10.1177_24741264241280597 – Supplemental material for Anti-VEGF Monotherapy vs Anti-VEGF and Steroid Combination Therapy for Diabetic Macular Edema: A Meta-analysis [file sj-zip-1-vrd-10.1177_24741264241280597.zip › Supplemental Figure 2. h.jpg]

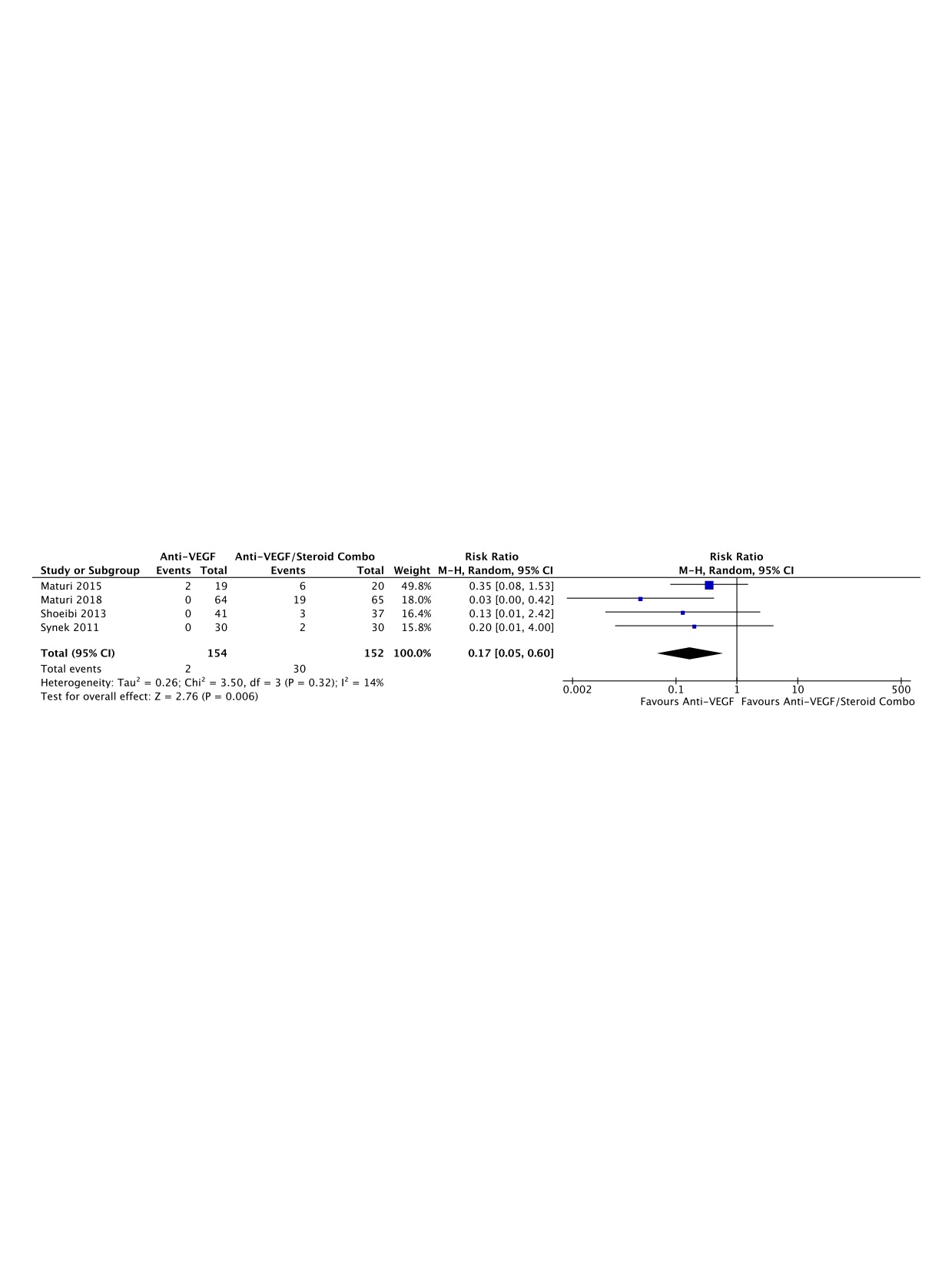

Supplement: sj-zip-1-vrd-10.1177_24741264241280597 – Supplemental material for Anti-VEGF Monotherapy vs Anti-VEGF and Steroid Combination Therapy for Diabetic Macular Edema: A Meta-analysis [file sj-zip-1-vrd-10.1177_24741264241280597.zip › Supplemental Figure 2. o.jpg]

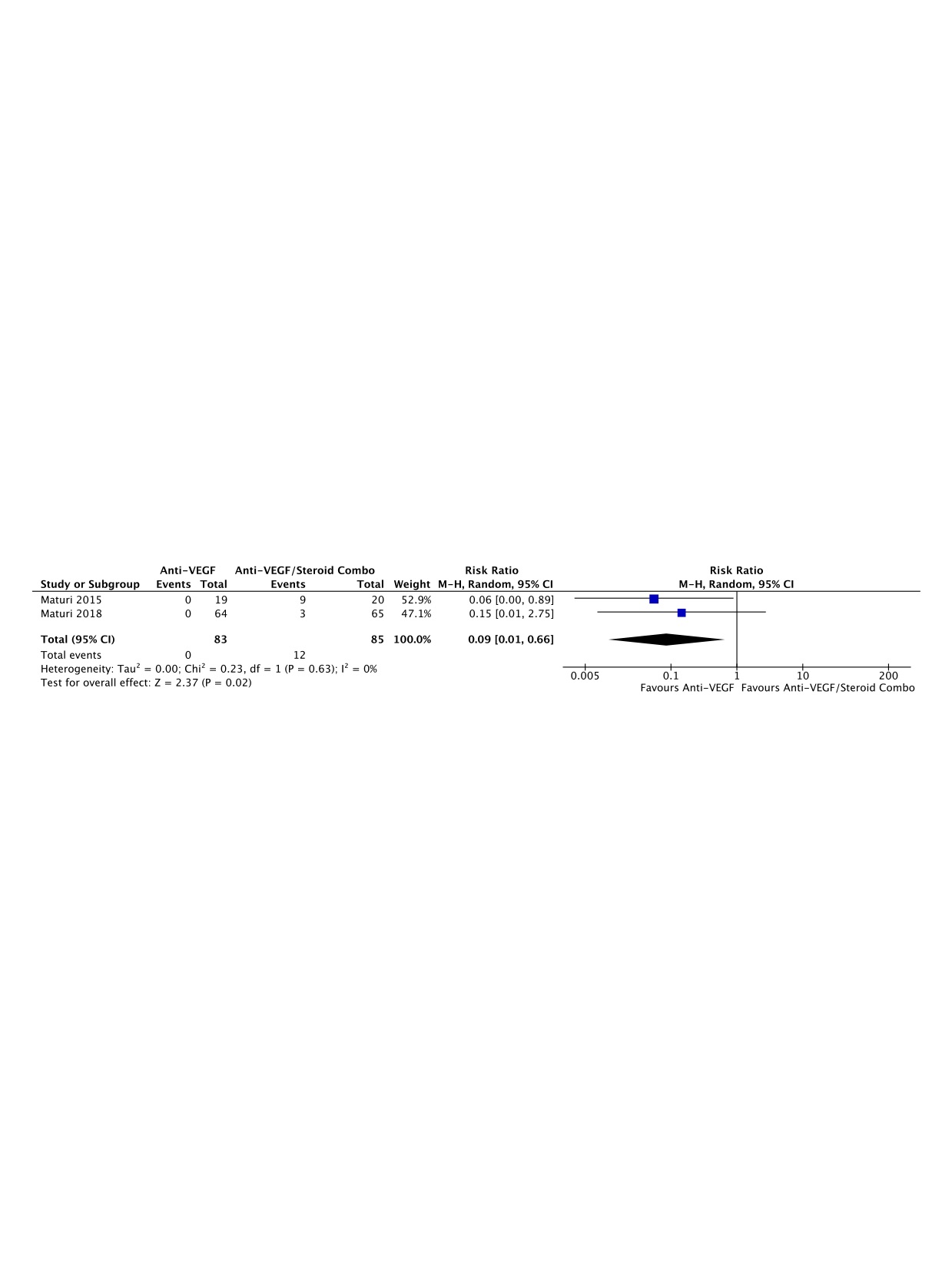

Supplement: sj-zip-1-vrd-10.1177_24741264241280597 – Supplemental material for Anti-VEGF Monotherapy vs Anti-VEGF and Steroid Combination Therapy for Diabetic Macular Edema: A Meta-analysis [file sj-zip-1-vrd-10.1177_24741264241280597.zip › Supplemental Figure 2. s.jpg]

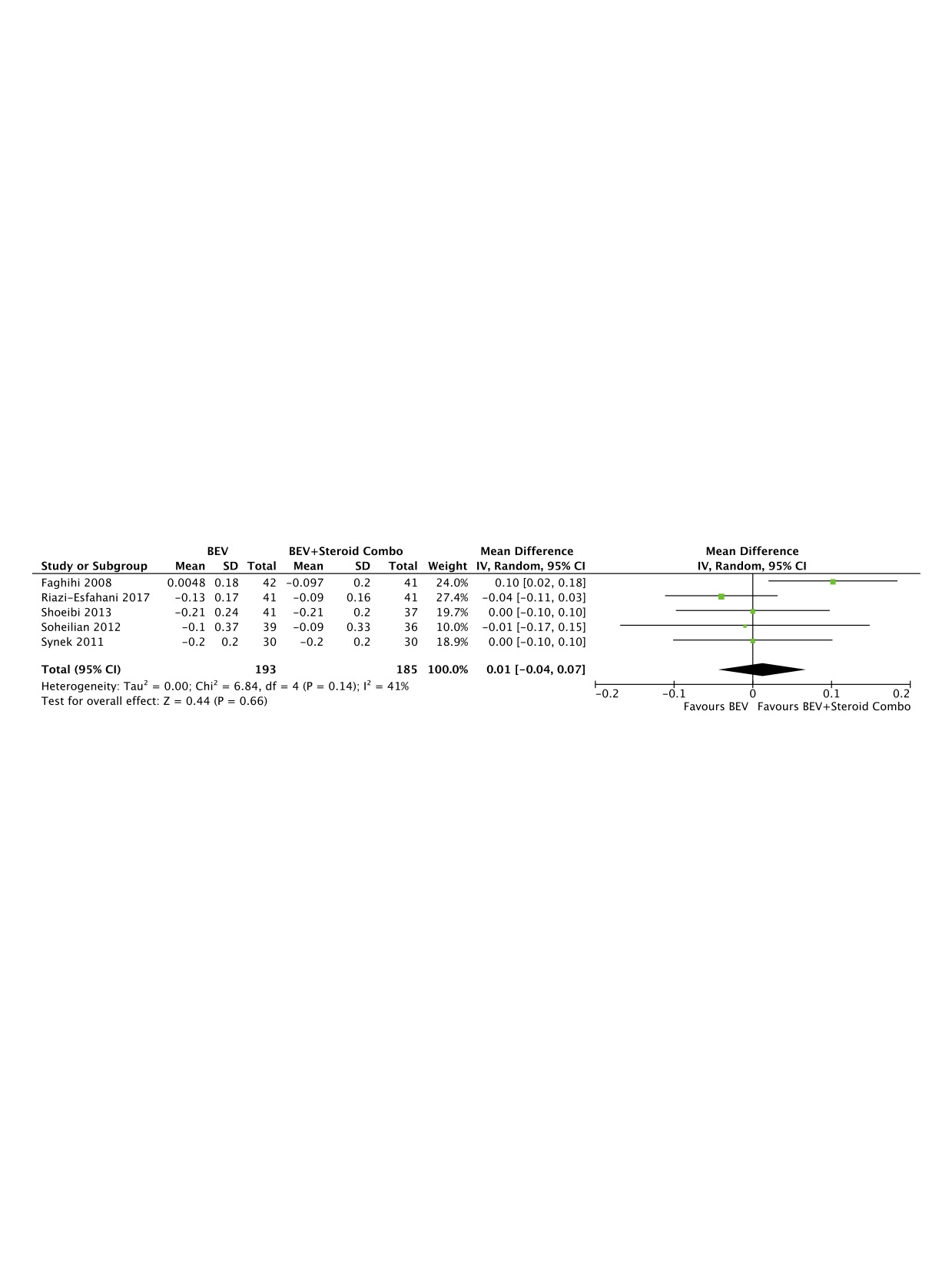

Supplement: sj-zip-1-vrd-10.1177_24741264241280597 – Supplemental material for Anti-VEGF Monotherapy vs Anti-VEGF and Steroid Combination Therapy for Diabetic Macular Edema: A Meta-analysis [file sj-zip-1-vrd-10.1177_24741264241280597.zip › Supplemental Figure 3. b.jpg]

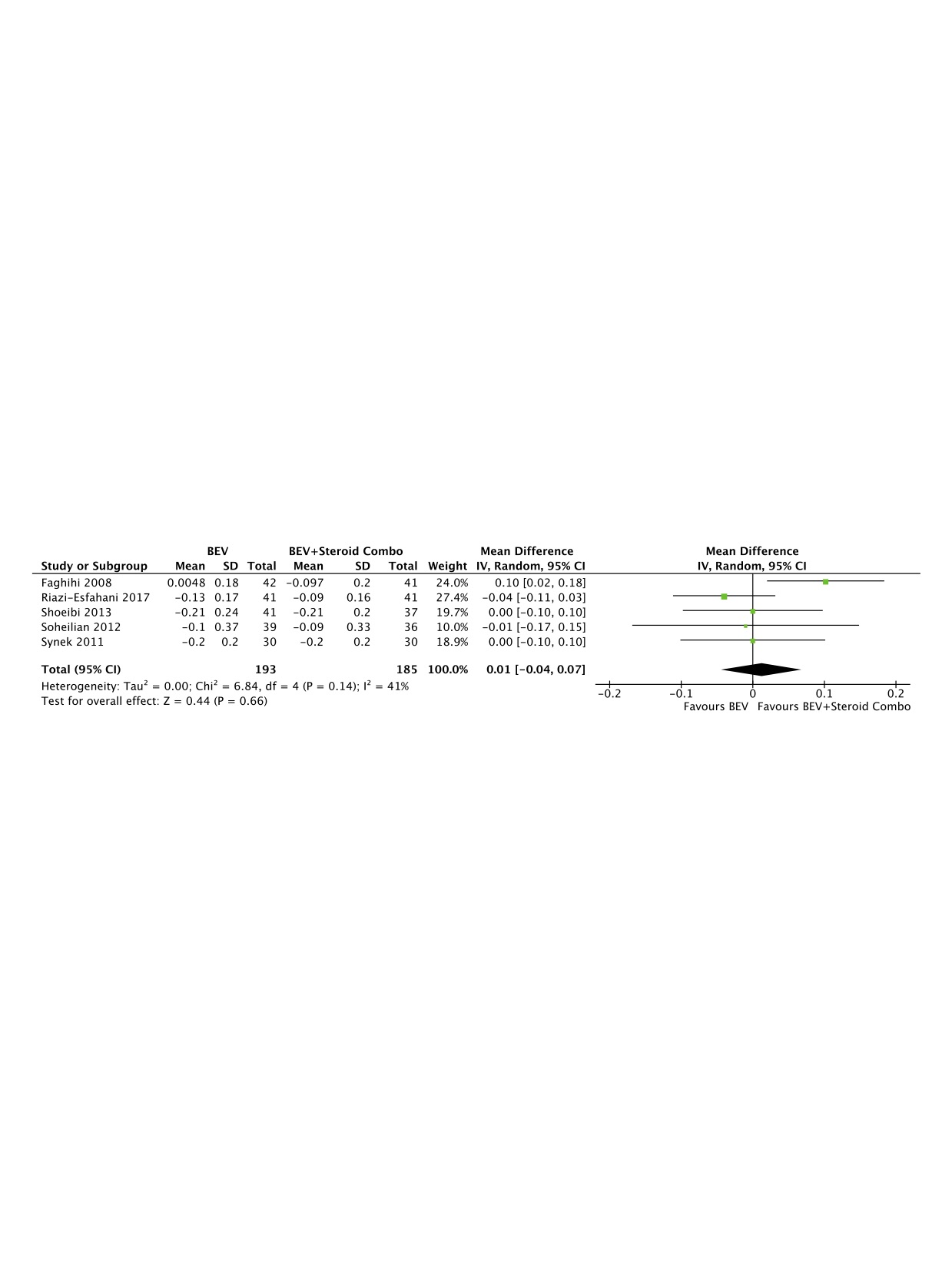

Supplement: sj-zip-1-vrd-10.1177_24741264241280597 – Supplemental material for Anti-VEGF Monotherapy vs Anti-VEGF and Steroid Combination Therapy for Diabetic Macular Edema: A Meta-analysis [file sj-zip-1-vrd-10.1177_24741264241280597.zip › Supplemental Figure 3. d.jpg]

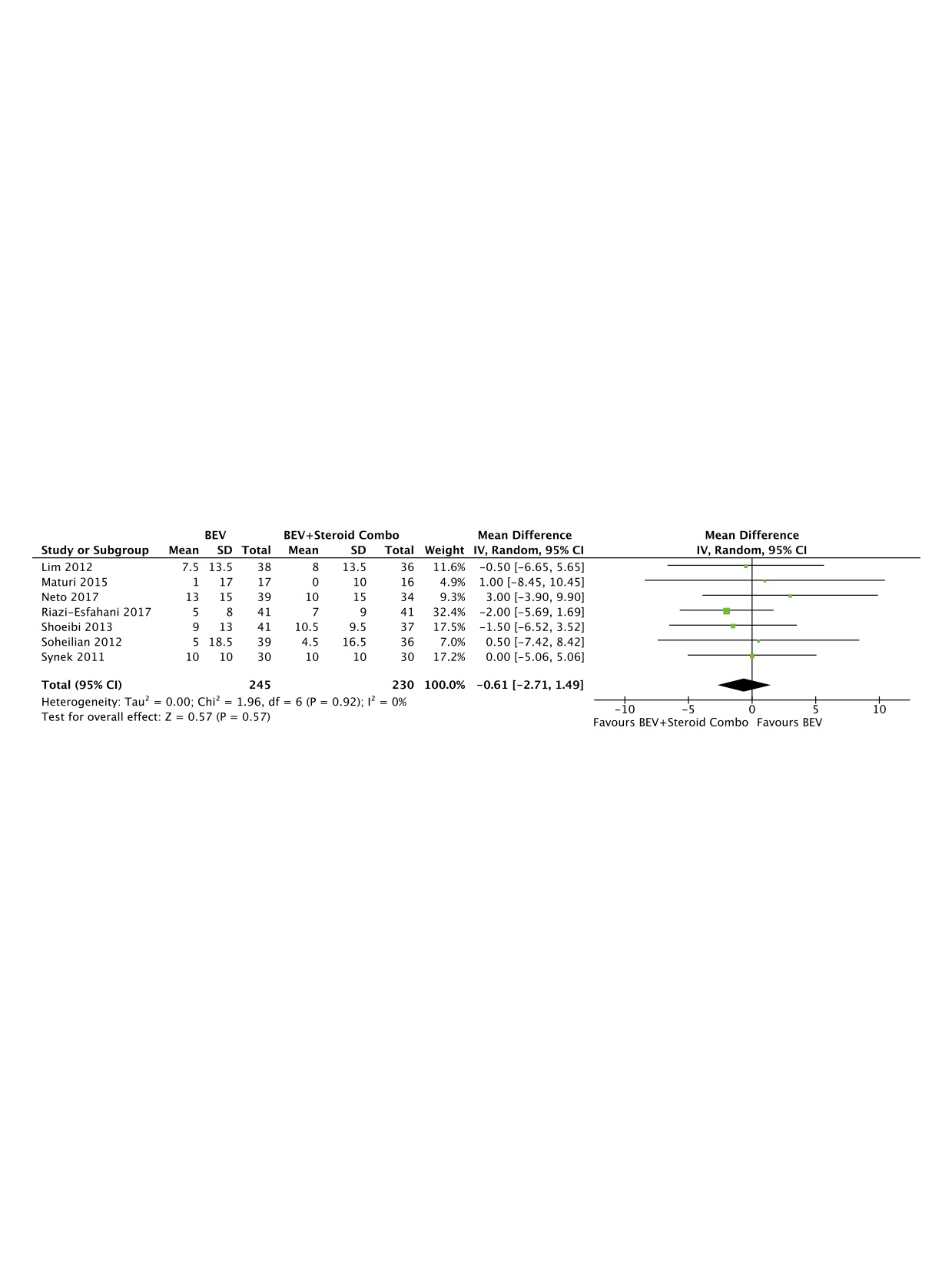

Supplement: sj-zip-1-vrd-10.1177_24741264241280597 – Supplemental material for Anti-VEGF Monotherapy vs Anti-VEGF and Steroid Combination Therapy for Diabetic Macular Edema: A Meta-analysis [file sj-zip-1-vrd-10.1177_24741264241280597.zip › Supplemental Figure 3. a.jpg]

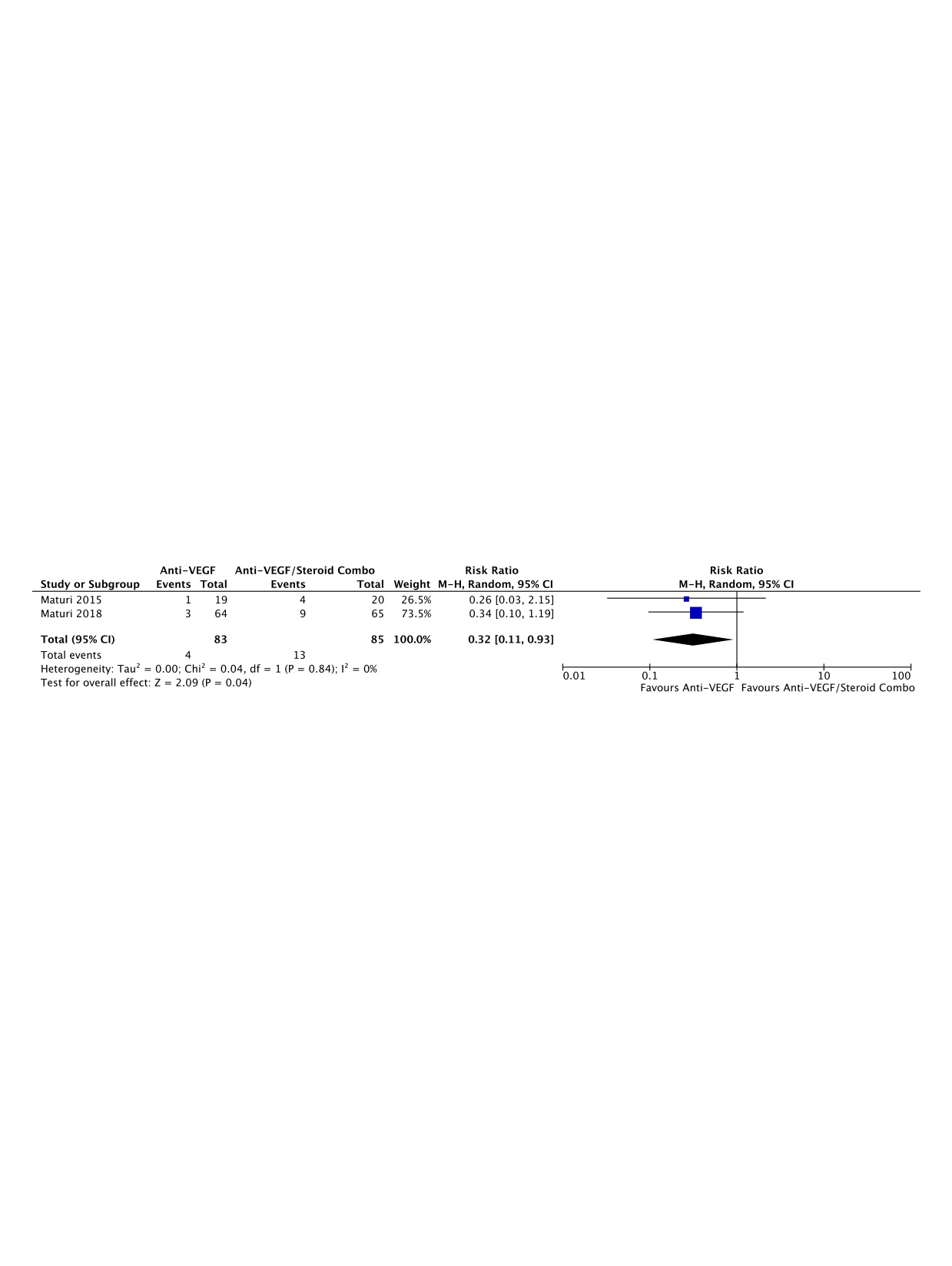

Supplement: sj-zip-1-vrd-10.1177_24741264241280597 – Supplemental material for Anti-VEGF Monotherapy vs Anti-VEGF and Steroid Combination Therapy for Diabetic Macular Edema: A Meta-analysis [file sj-zip-1-vrd-10.1177_24741264241280597.zip › Supplemental Figure 2. t.jpg]

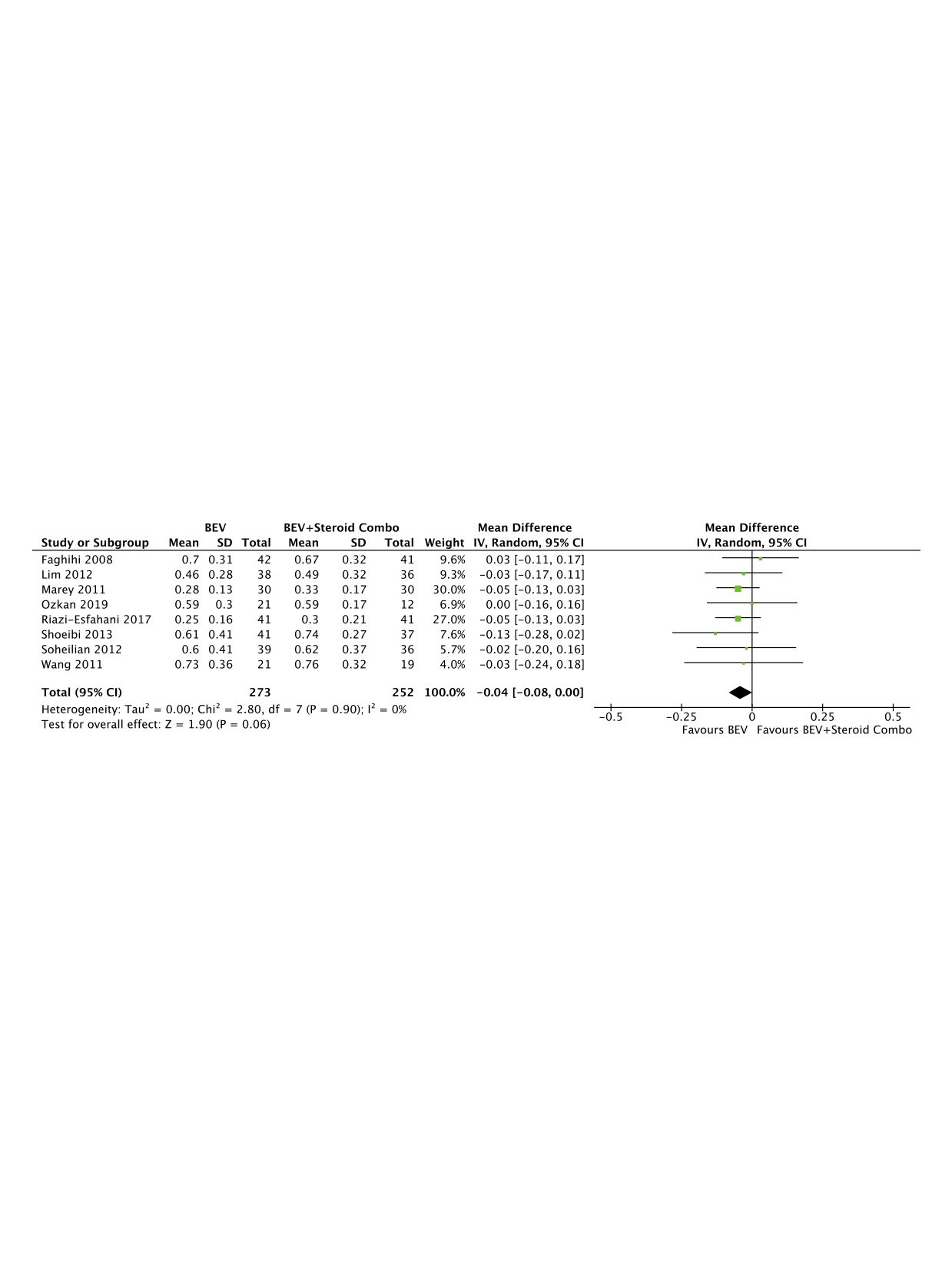

Supplement: sj-zip-1-vrd-10.1177_24741264241280597 – Supplemental material for Anti-VEGF Monotherapy vs Anti-VEGF and Steroid Combination Therapy for Diabetic Macular Edema: A Meta-analysis [file sj-zip-1-vrd-10.1177_24741264241280597.zip › Supplemental Figure 3. g.jpg]

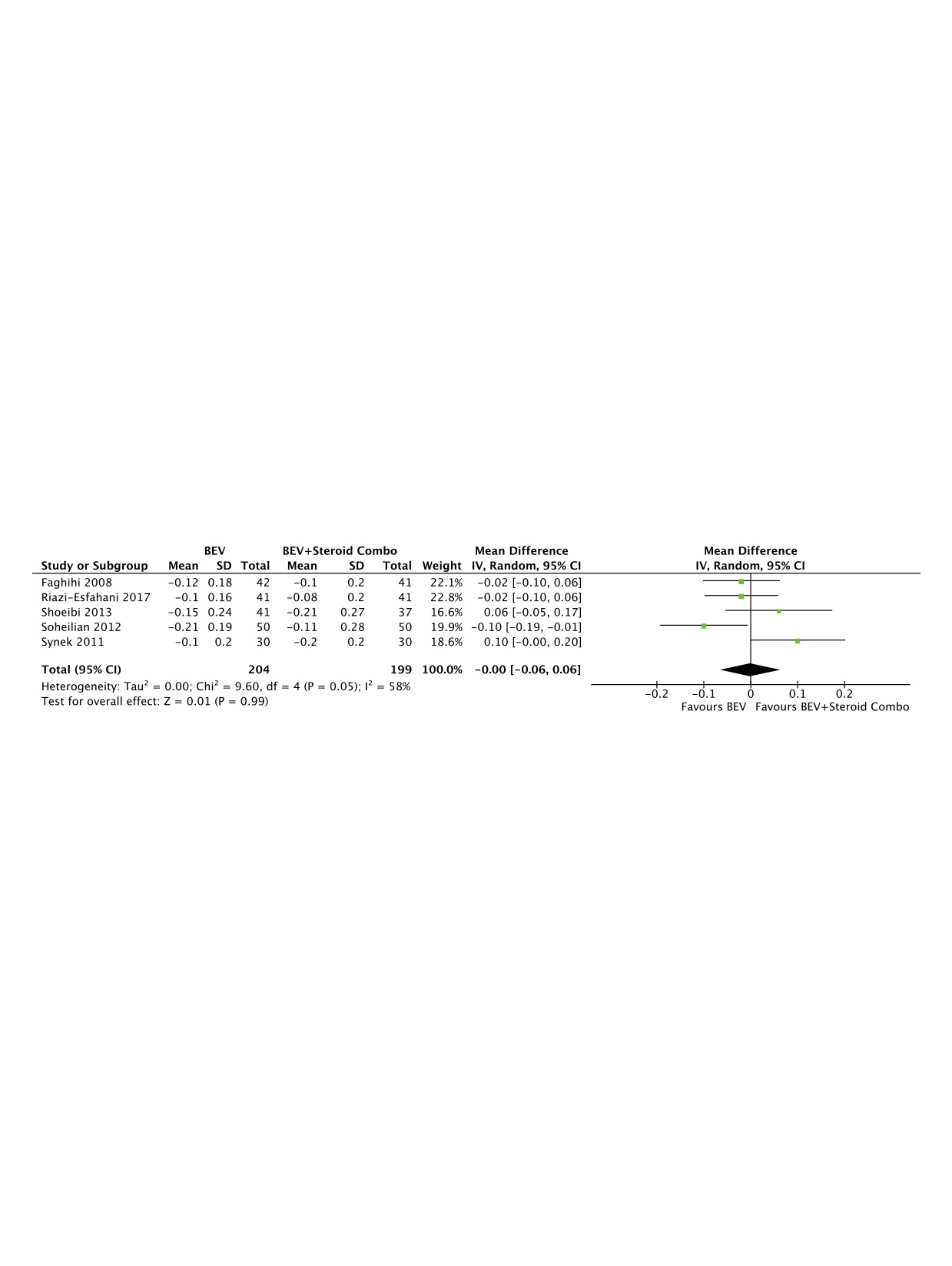

Supplement: sj-zip-1-vrd-10.1177_24741264241280597 – Supplemental material for Anti-VEGF Monotherapy vs Anti-VEGF and Steroid Combination Therapy for Diabetic Macular Edema: A Meta-analysis [file sj-zip-1-vrd-10.1177_24741264241280597.zip › Supplemental Figure 3. e.jpg]

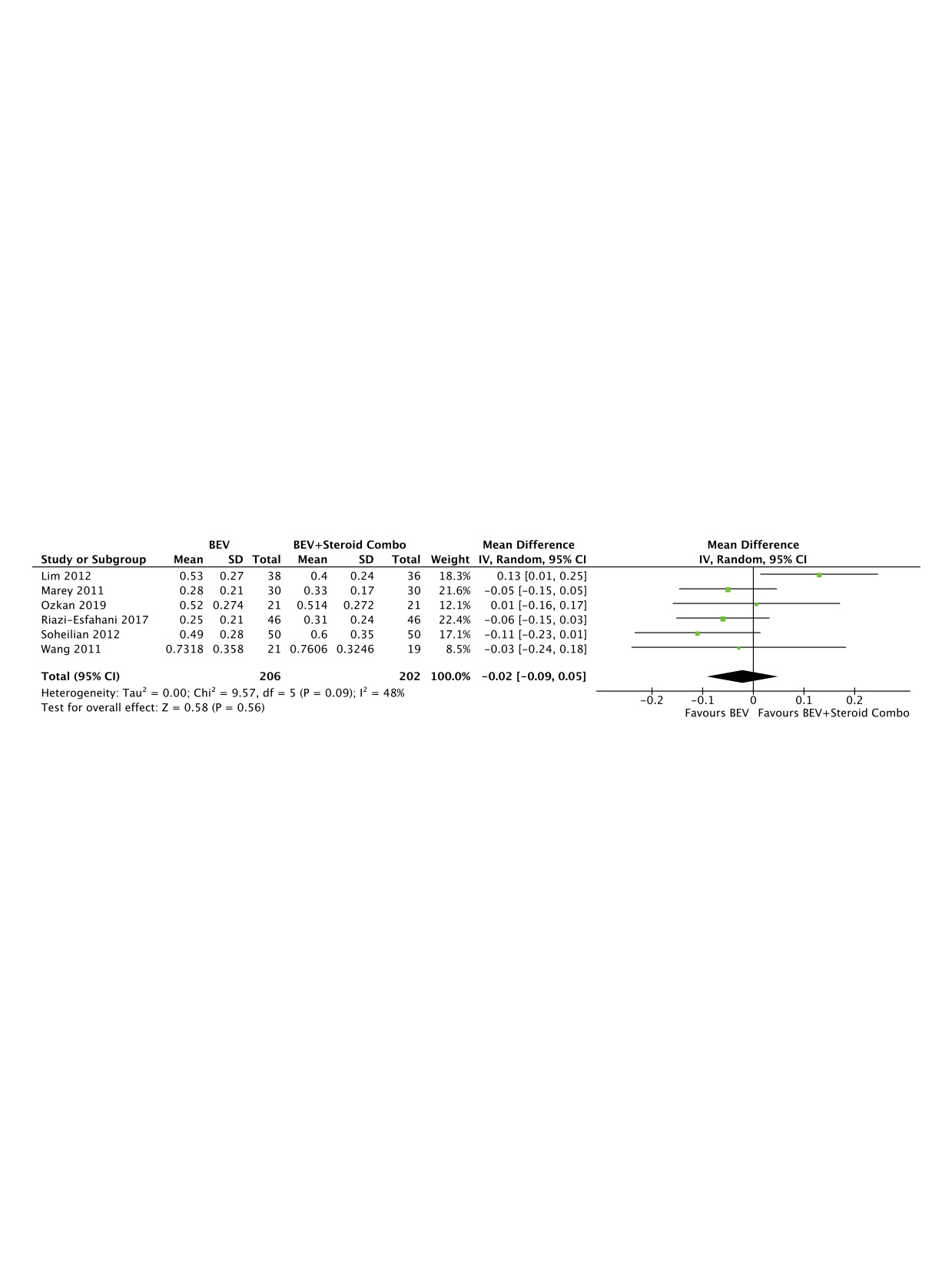

Supplement: sj-zip-1-vrd-10.1177_24741264241280597 – Supplemental material for Anti-VEGF Monotherapy vs Anti-VEGF and Steroid Combination Therapy for Diabetic Macular Edema: A Meta-analysis [file sj-zip-1-vrd-10.1177_24741264241280597.zip › Supplemental Figure 3. i.jpg]

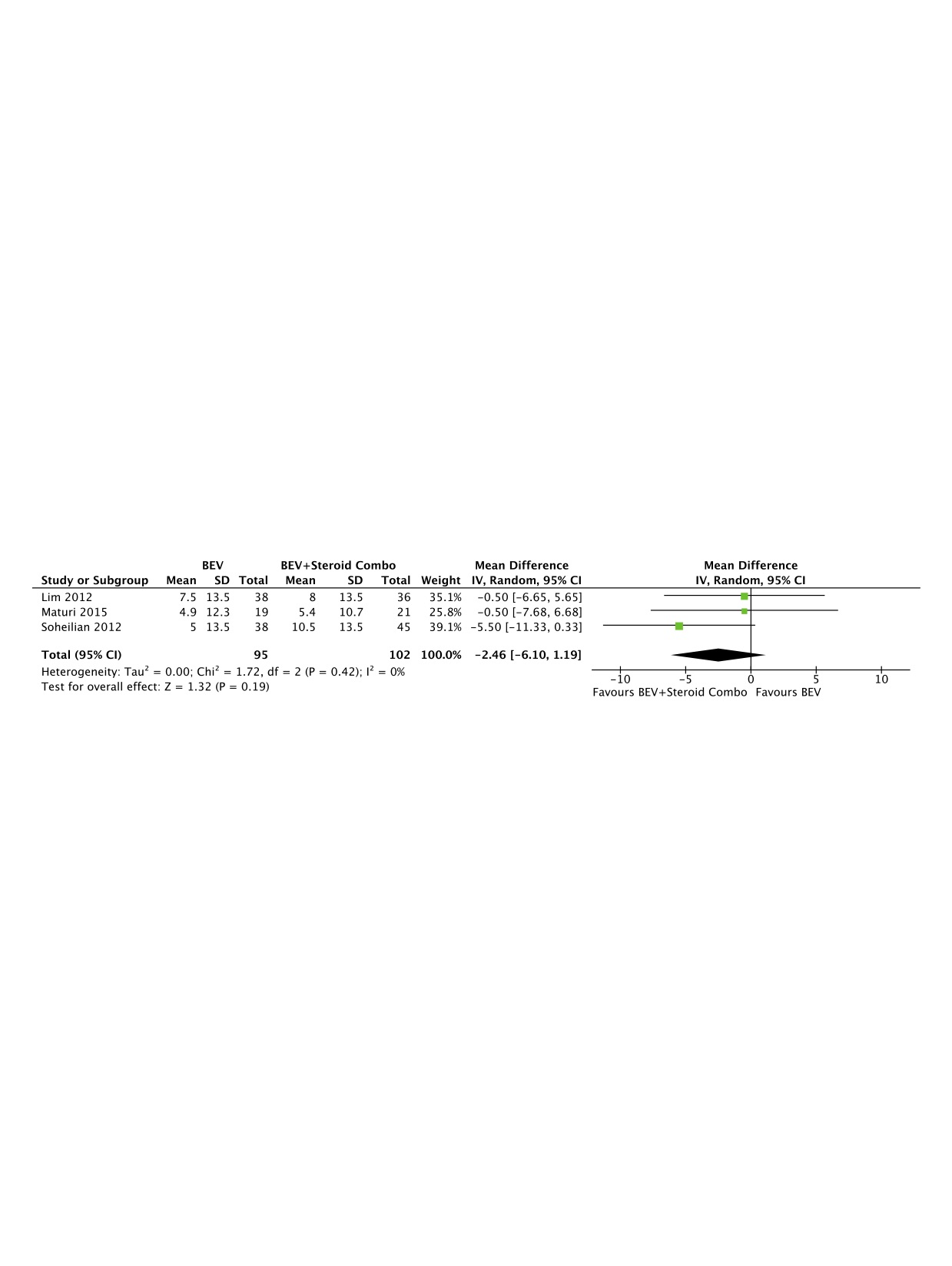

Supplement: sj-zip-1-vrd-10.1177_24741264241280597 – Supplemental material for Anti-VEGF Monotherapy vs Anti-VEGF and Steroid Combination Therapy for Diabetic Macular Edema: A Meta-analysis [file sj-zip-1-vrd-10.1177_24741264241280597.zip › Supplemental Figure 3. f.jpg]

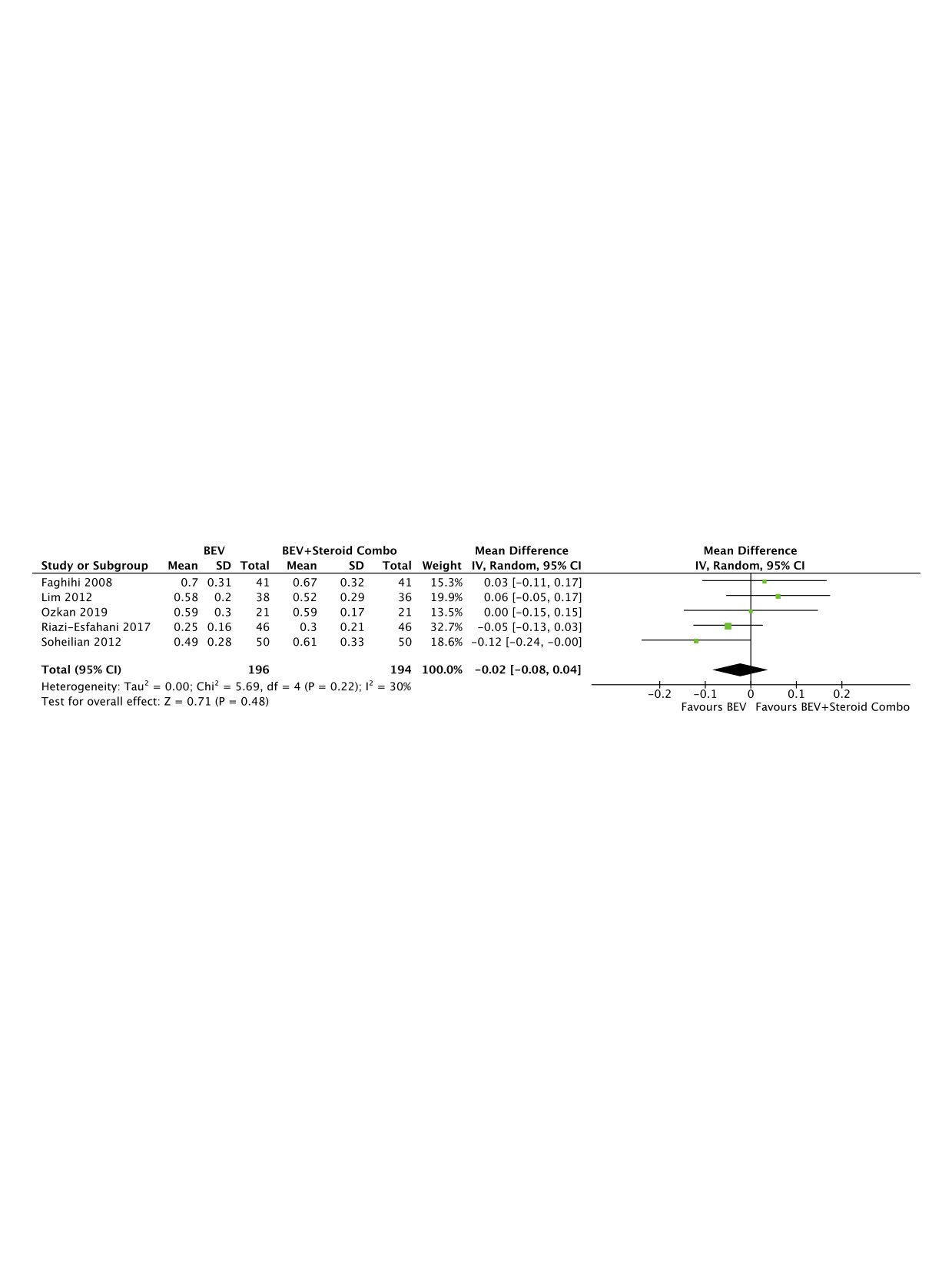

Supplement: sj-zip-1-vrd-10.1177_24741264241280597 – Supplemental material for Anti-VEGF Monotherapy vs Anti-VEGF and Steroid Combination Therapy for Diabetic Macular Edema: A Meta-analysis [file sj-zip-1-vrd-10.1177_24741264241280597.zip › Supplemental Figure 3. j.jpg]

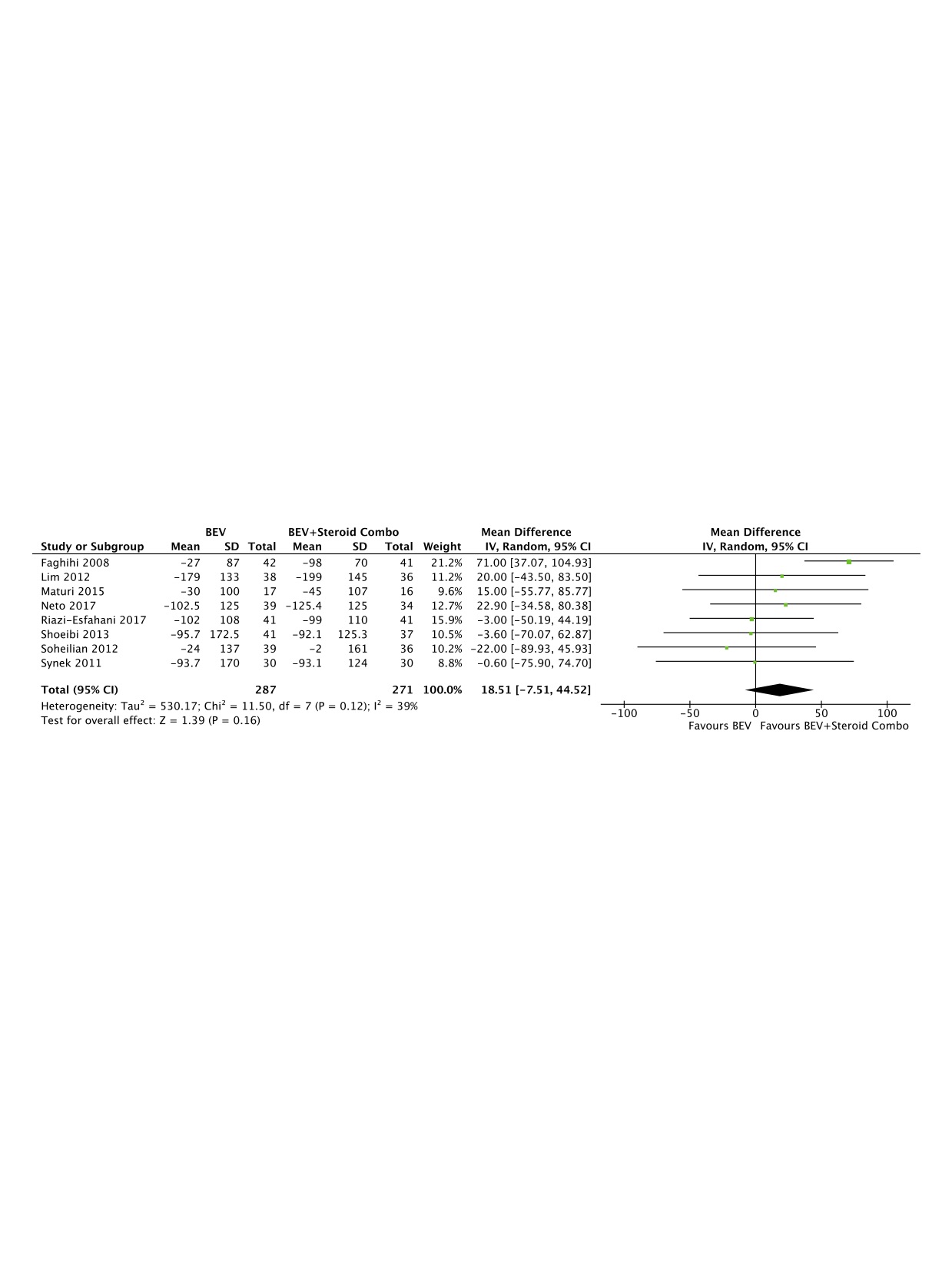

Supplement: sj-zip-1-vrd-10.1177_24741264241280597 – Supplemental material for Anti-VEGF Monotherapy vs Anti-VEGF and Steroid Combination Therapy for Diabetic Macular Edema: A Meta-analysis [file sj-zip-1-vrd-10.1177_24741264241280597.zip › Supplemental Figure 3. k.jpg]

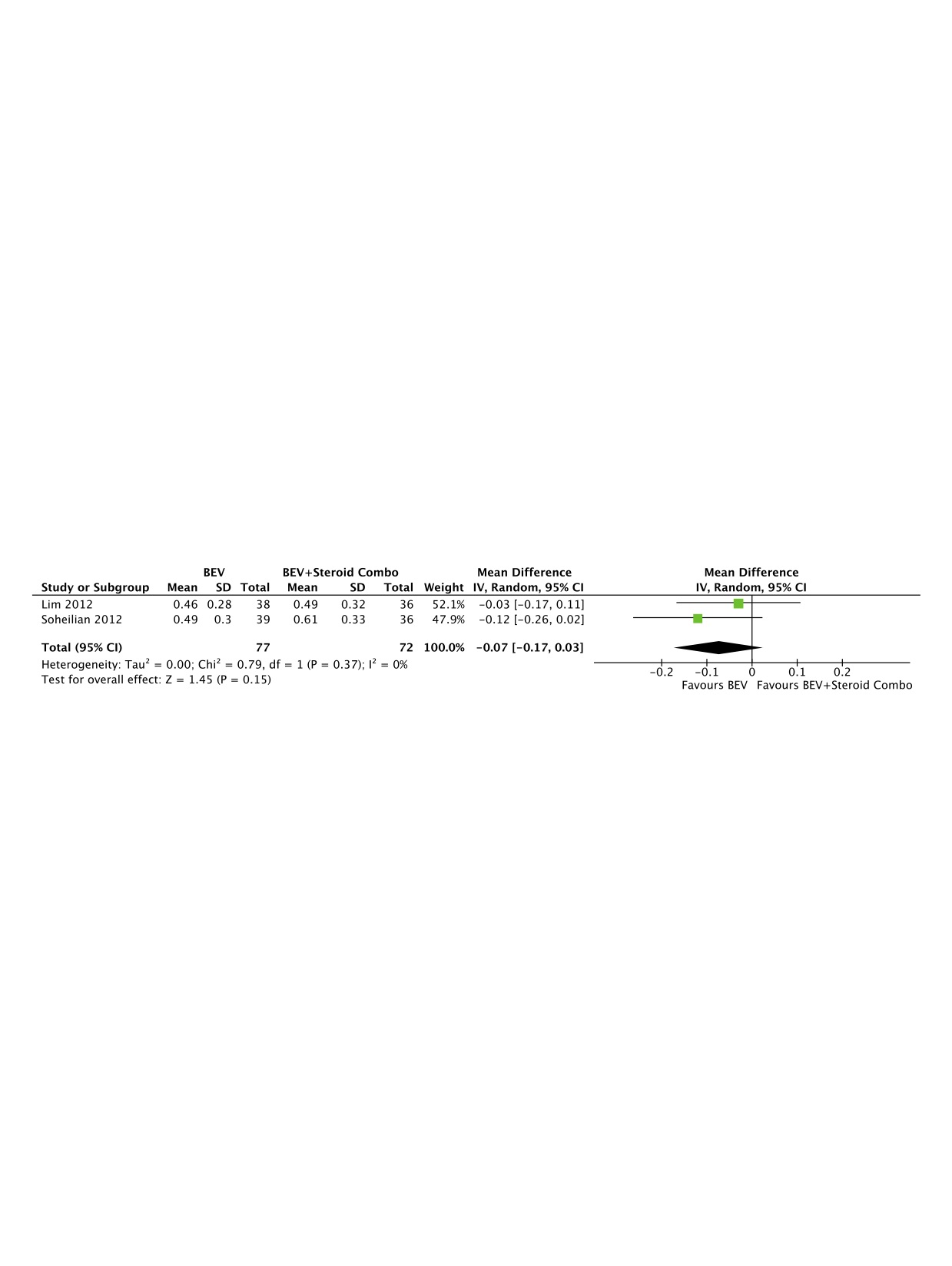

Supplement: sj-zip-1-vrd-10.1177_24741264241280597 – Supplemental material for Anti-VEGF Monotherapy vs Anti-VEGF and Steroid Combination Therapy for Diabetic Macular Edema: A Meta-analysis [file sj-zip-1-vrd-10.1177_24741264241280597.zip › Supplemental Figure 3. h.jpg]

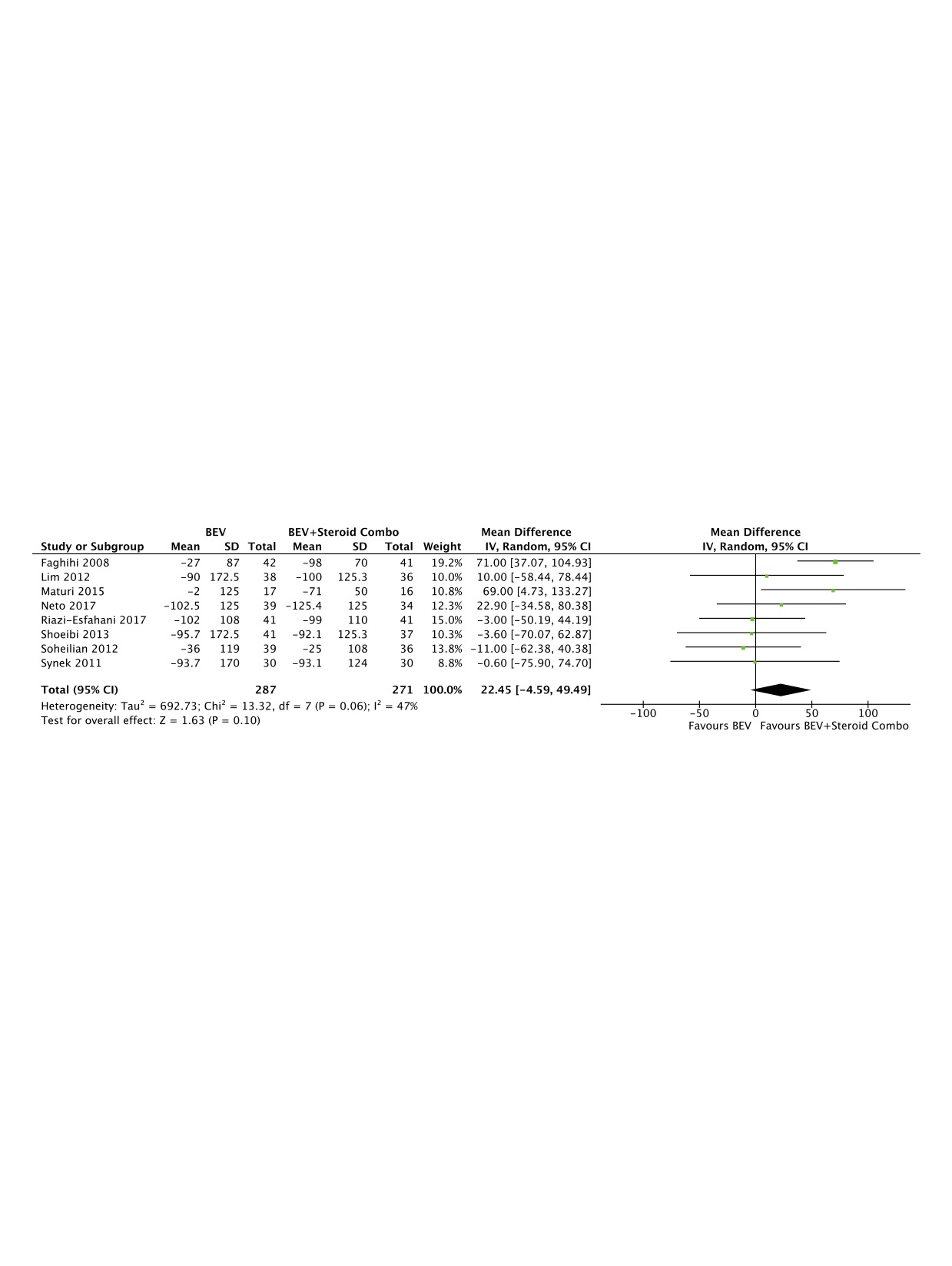

Supplement: sj-zip-1-vrd-10.1177_24741264241280597 – Supplemental material for Anti-VEGF Monotherapy vs Anti-VEGF and Steroid Combination Therapy for Diabetic Macular Edema: A Meta-analysis [file sj-zip-1-vrd-10.1177_24741264241280597.zip › Supplemental Figure 3. l.jpg]

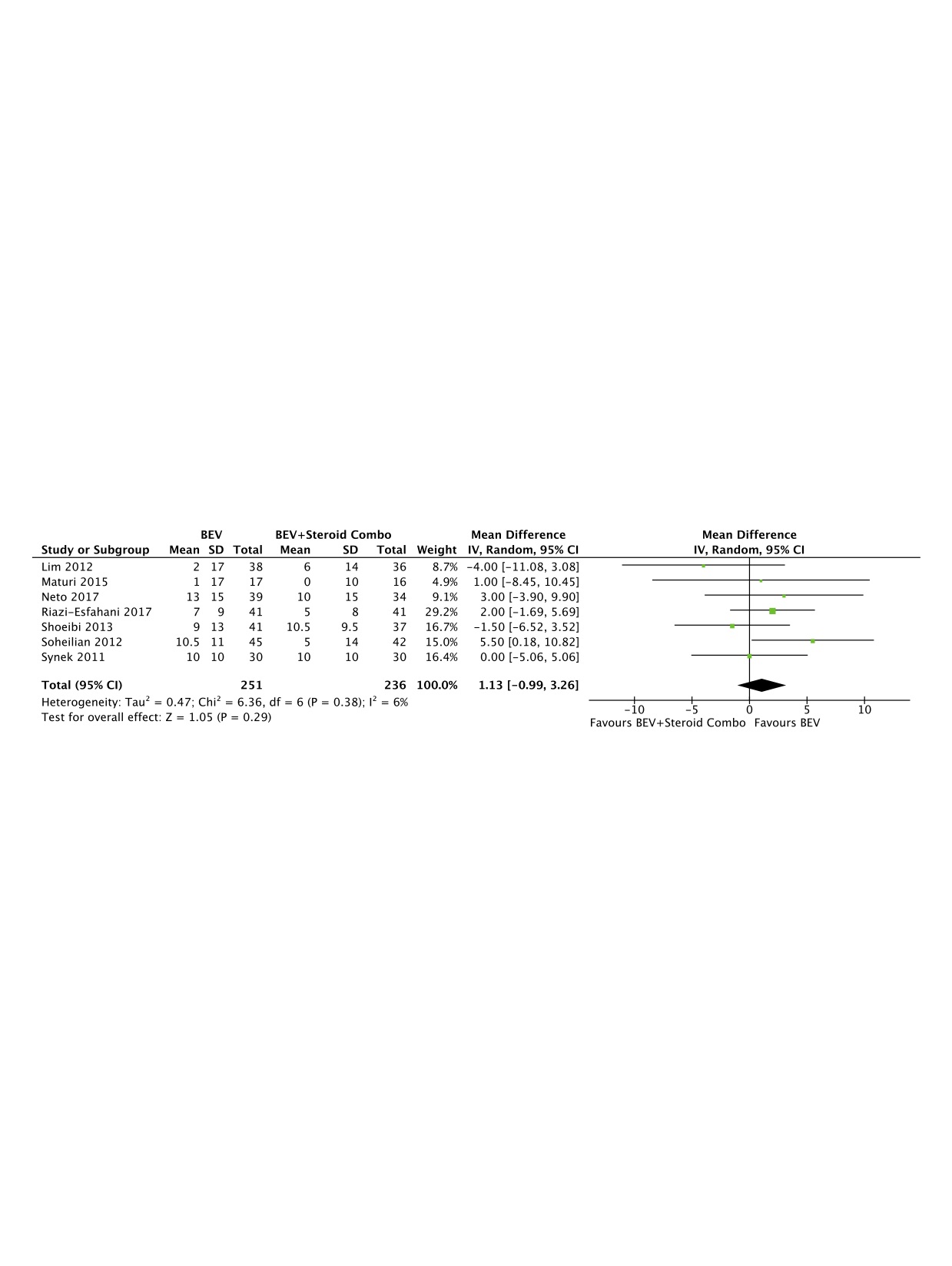

Supplement: sj-zip-1-vrd-10.1177_24741264241280597 – Supplemental material for Anti-VEGF Monotherapy vs Anti-VEGF and Steroid Combination Therapy for Diabetic Macular Edema: A Meta-analysis [file sj-zip-1-vrd-10.1177_24741264241280597.zip › Supplemental Figure 3. c.jpg]

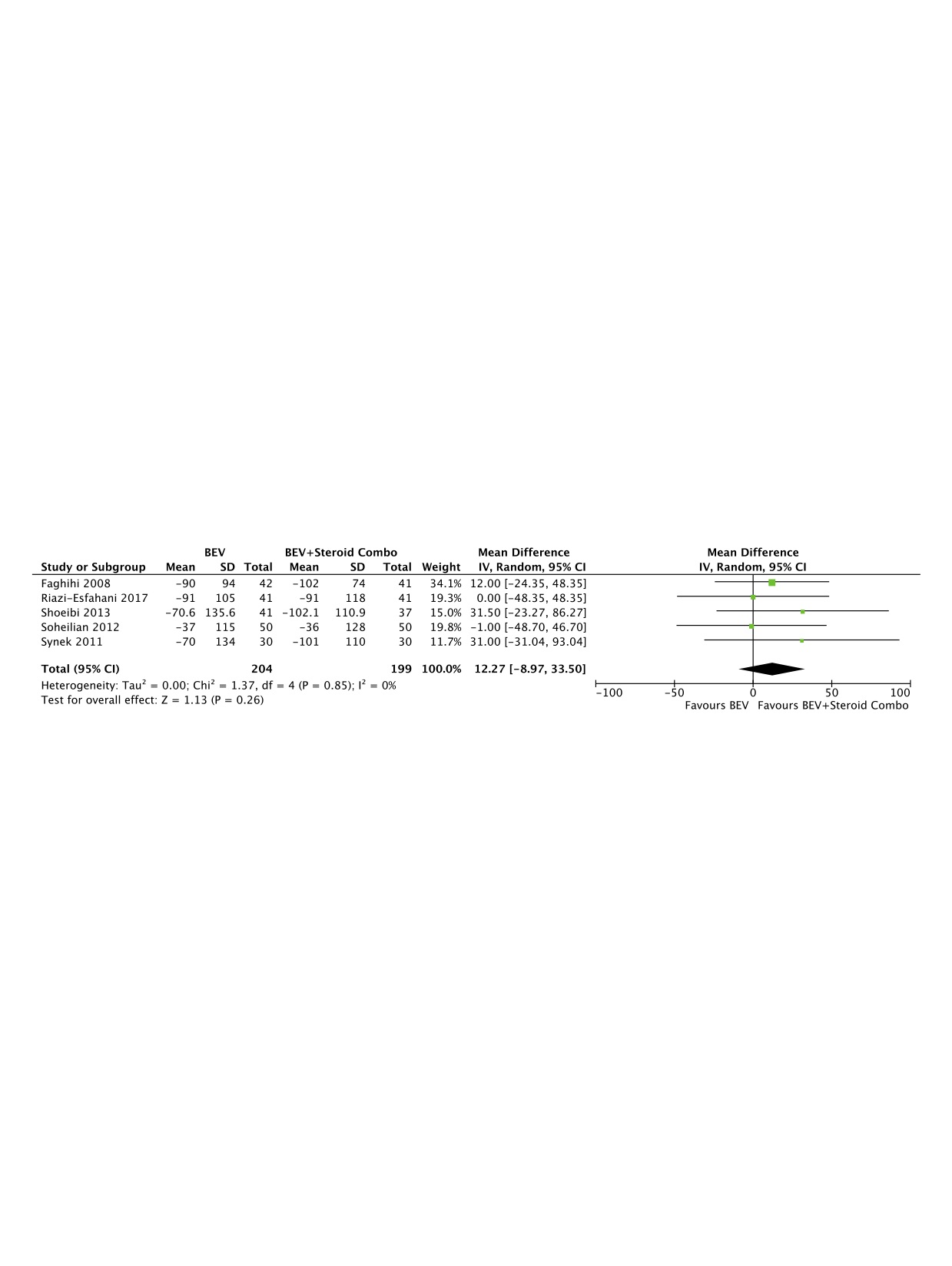

Supplement: sj-zip-1-vrd-10.1177_24741264241280597 – Supplemental material for Anti-VEGF Monotherapy vs Anti-VEGF and Steroid Combination Therapy for Diabetic Macular Edema: A Meta-analysis [file sj-zip-1-vrd-10.1177_24741264241280597.zip › Supplemental Figure 3. m.jpg]

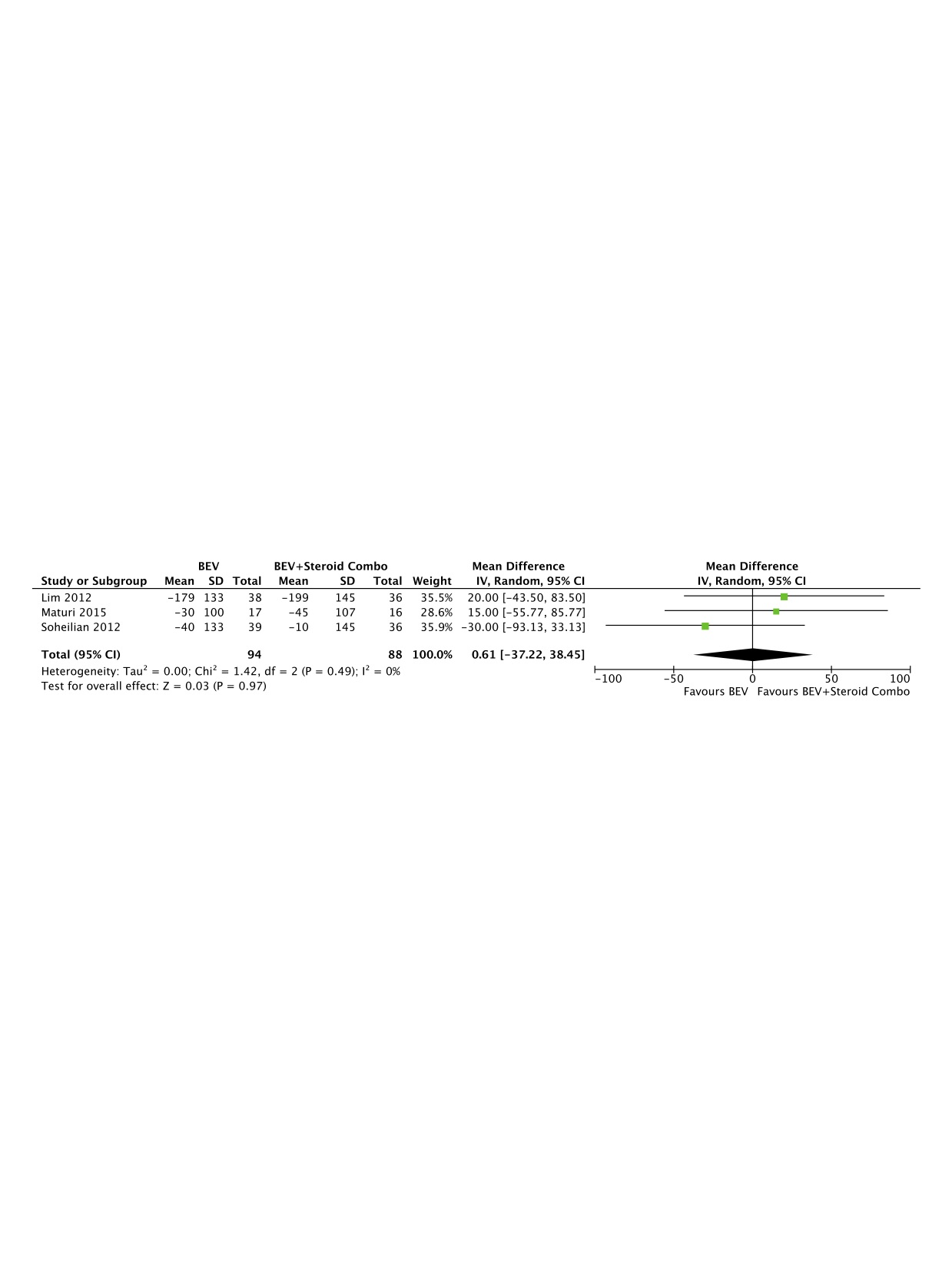

Supplement: sj-zip-1-vrd-10.1177_24741264241280597 – Supplemental material for Anti-VEGF Monotherapy vs Anti-VEGF and Steroid Combination Therapy for Diabetic Macular Edema: A Meta-analysis [file sj-zip-1-vrd-10.1177_24741264241280597.zip › Supplemental Figure 3. n.jpg]

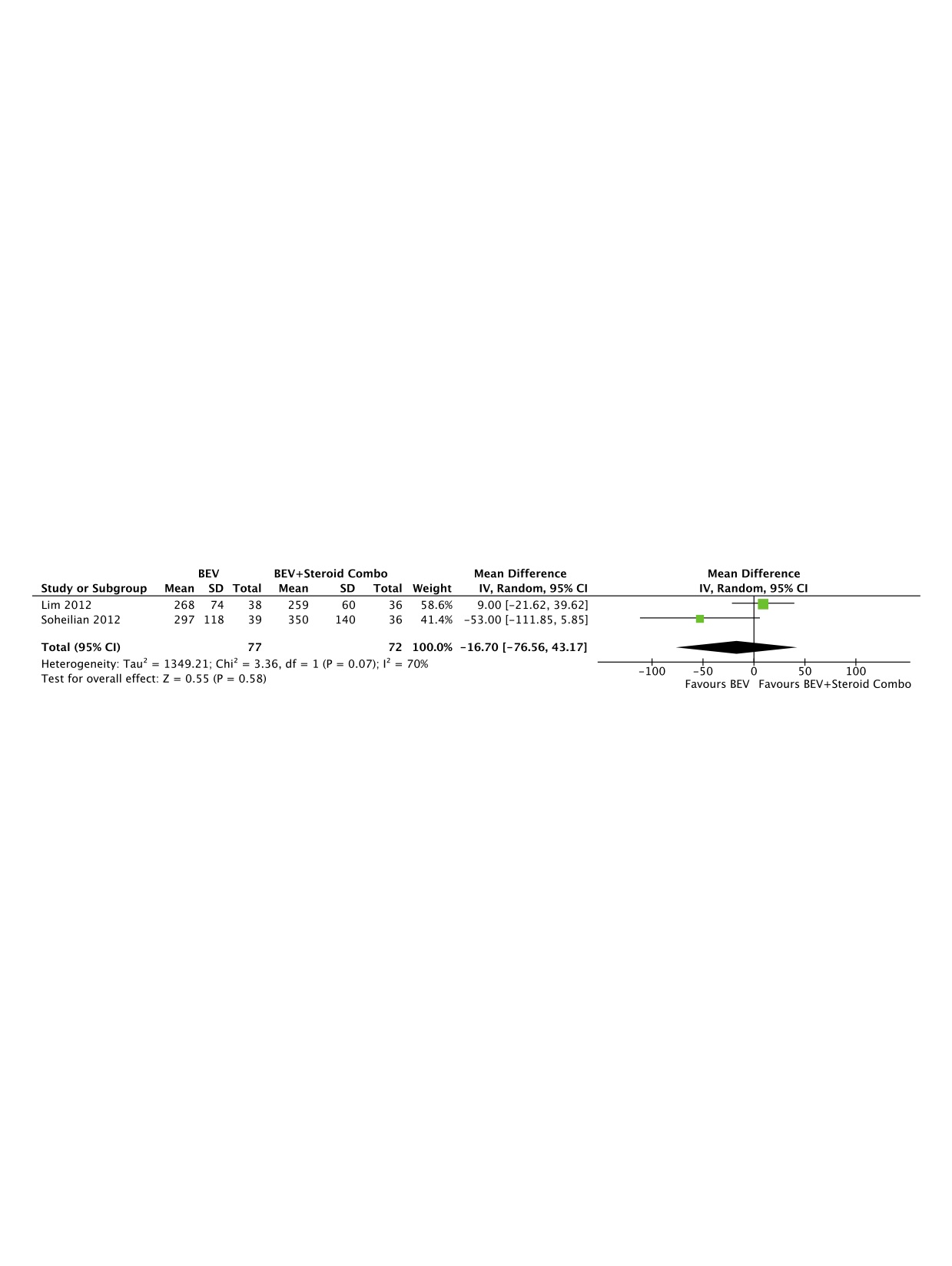

Supplement: sj-zip-1-vrd-10.1177_24741264241280597 – Supplemental material for Anti-VEGF Monotherapy vs Anti-VEGF and Steroid Combination Therapy for Diabetic Macular Edema: A Meta-analysis [file sj-zip-1-vrd-10.1177_24741264241280597.zip › Supplemental Figure 3. q.jpg]

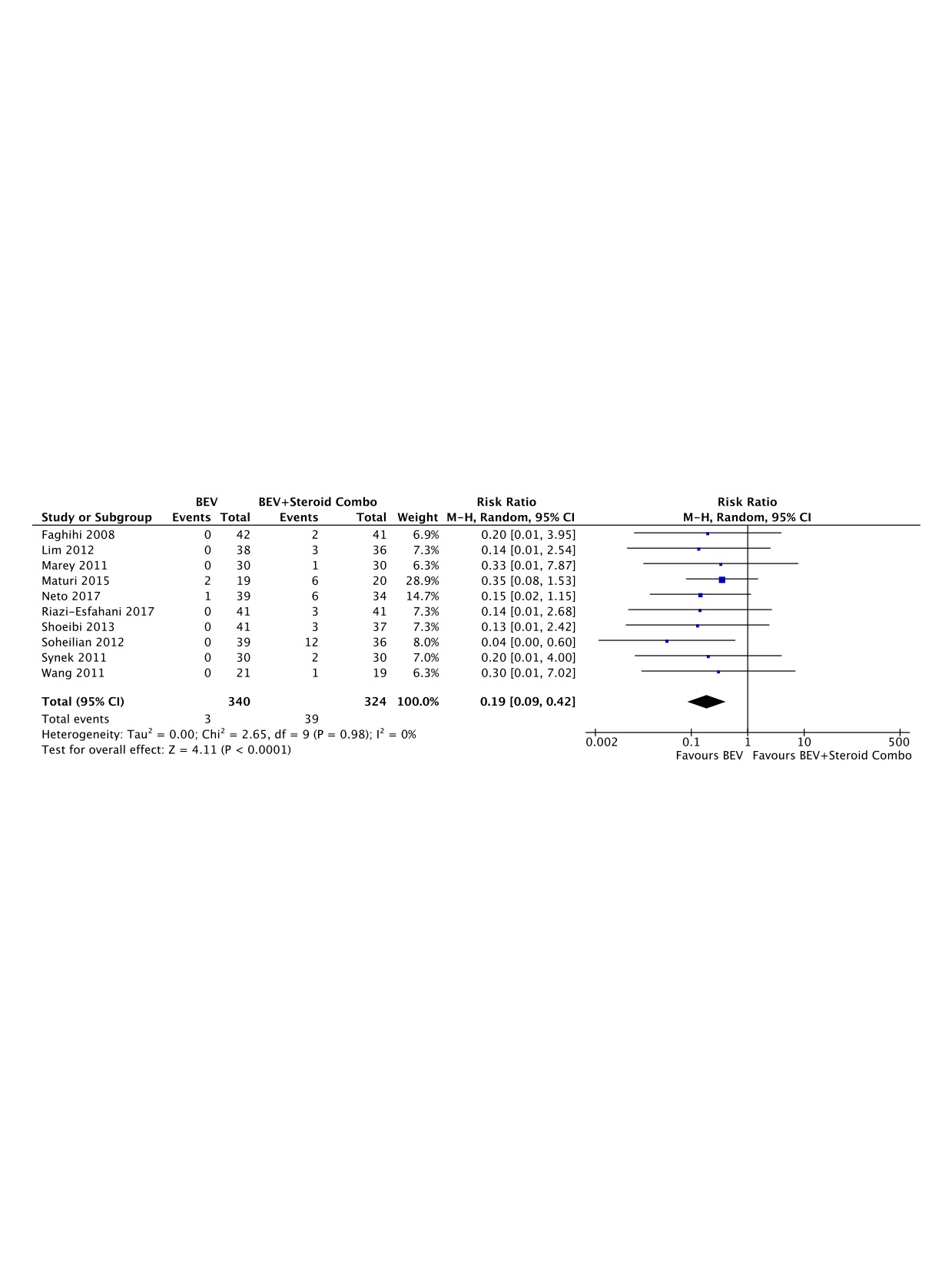

Supplement: sj-zip-1-vrd-10.1177_24741264241280597 – Supplemental material for Anti-VEGF Monotherapy vs Anti-VEGF and Steroid Combination Therapy for Diabetic Macular Edema: A Meta-analysis [file sj-zip-1-vrd-10.1177_24741264241280597.zip › Supplemental Figure 3. s.jpg]

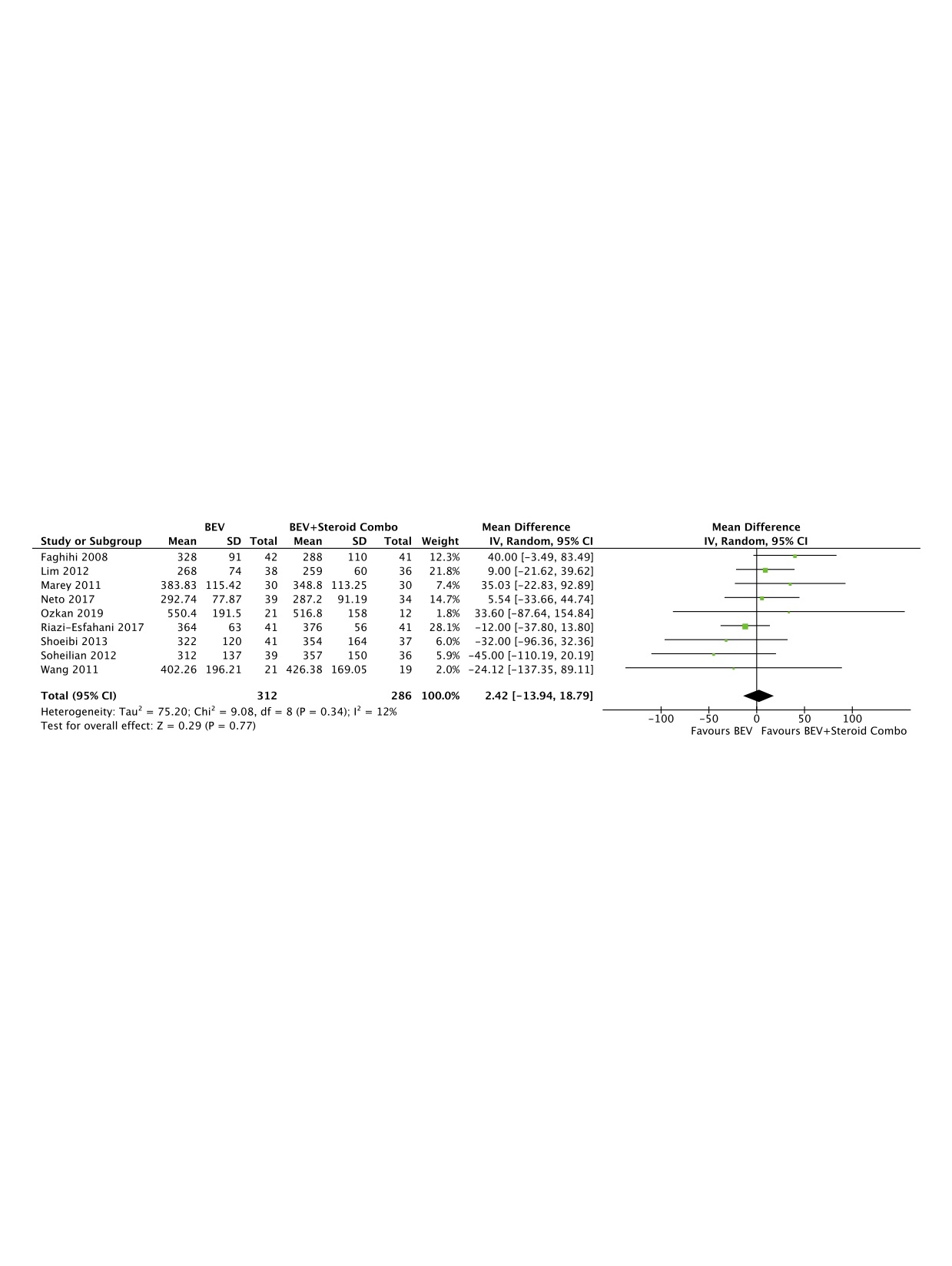

Supplement: sj-zip-1-vrd-10.1177_24741264241280597 – Supplemental material for Anti-VEGF Monotherapy vs Anti-VEGF and Steroid Combination Therapy for Diabetic Macular Edema: A Meta-analysis [file sj-zip-1-vrd-10.1177_24741264241280597.zip › Supplemental Figure 3. o.jpg]

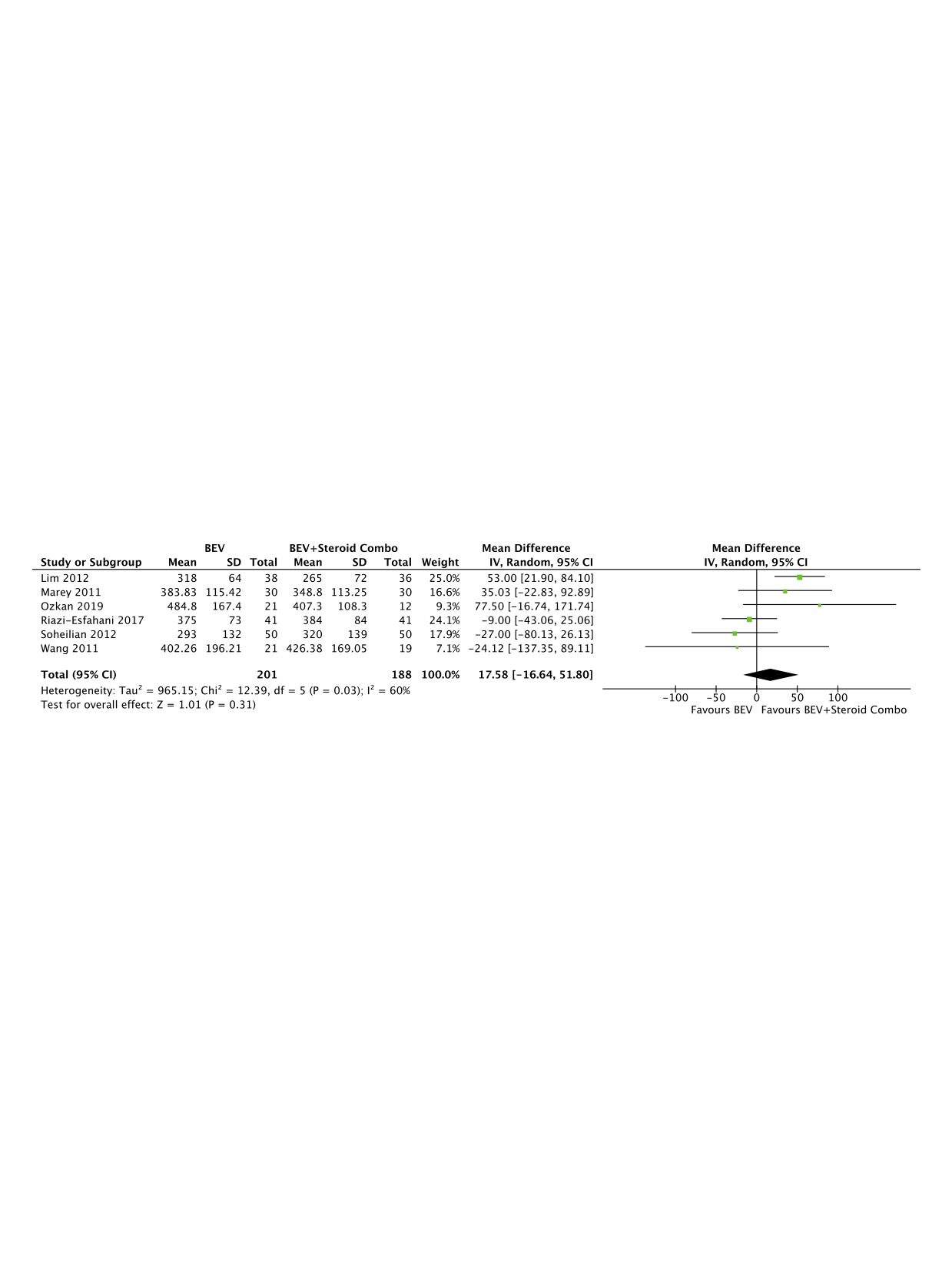

Supplement: sj-zip-1-vrd-10.1177_24741264241280597 – Supplemental material for Anti-VEGF Monotherapy vs Anti-VEGF and Steroid Combination Therapy for Diabetic Macular Edema: A Meta-analysis [file sj-zip-1-vrd-10.1177_24741264241280597.zip › Supplemental Figure 3. p.jpg]

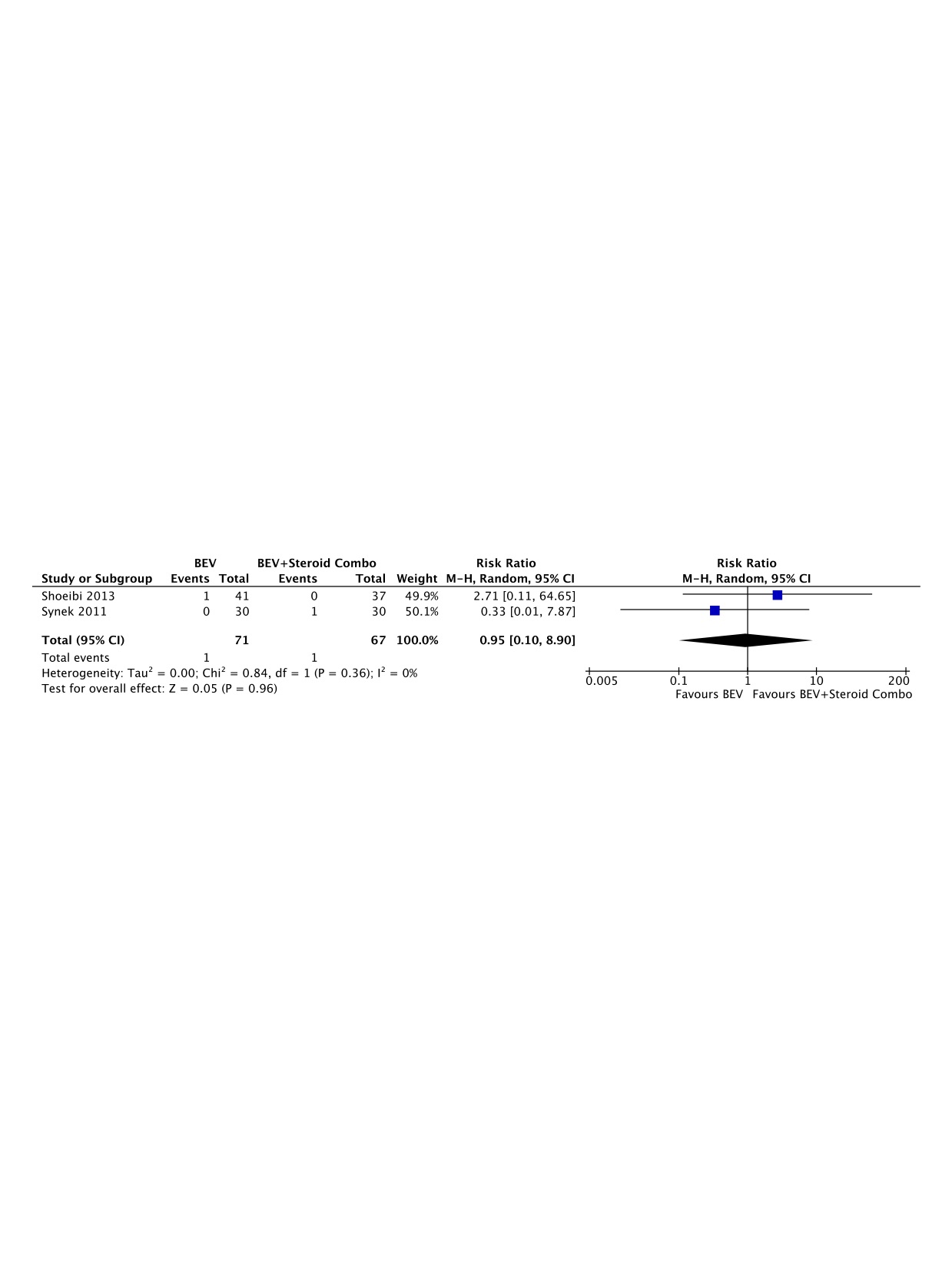

Supplement: sj-zip-1-vrd-10.1177_24741264241280597 – Supplemental material for Anti-VEGF Monotherapy vs Anti-VEGF and Steroid Combination Therapy for Diabetic Macular Edema: A Meta-analysis [file sj-zip-1-vrd-10.1177_24741264241280597.zip › Supplemental Figure 3. t.jpg]

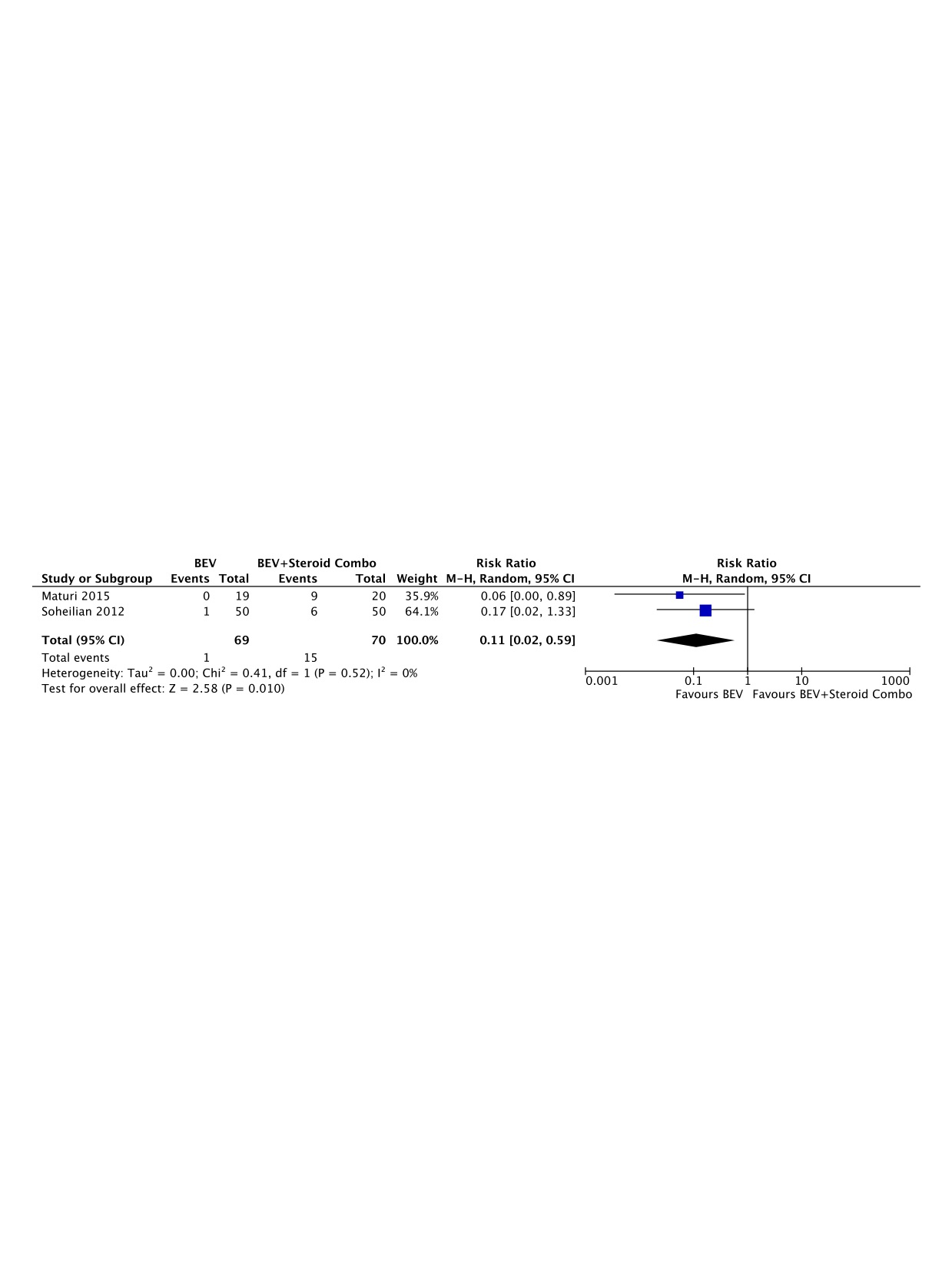

Supplement: sj-zip-1-vrd-10.1177_24741264241280597 – Supplemental material for Anti-VEGF Monotherapy vs Anti-VEGF and Steroid Combination Therapy for Diabetic Macular Edema: A Meta-analysis [file sj-zip-1-vrd-10.1177_24741264241280597.zip › Supplemental Figure 3. u.jpg]

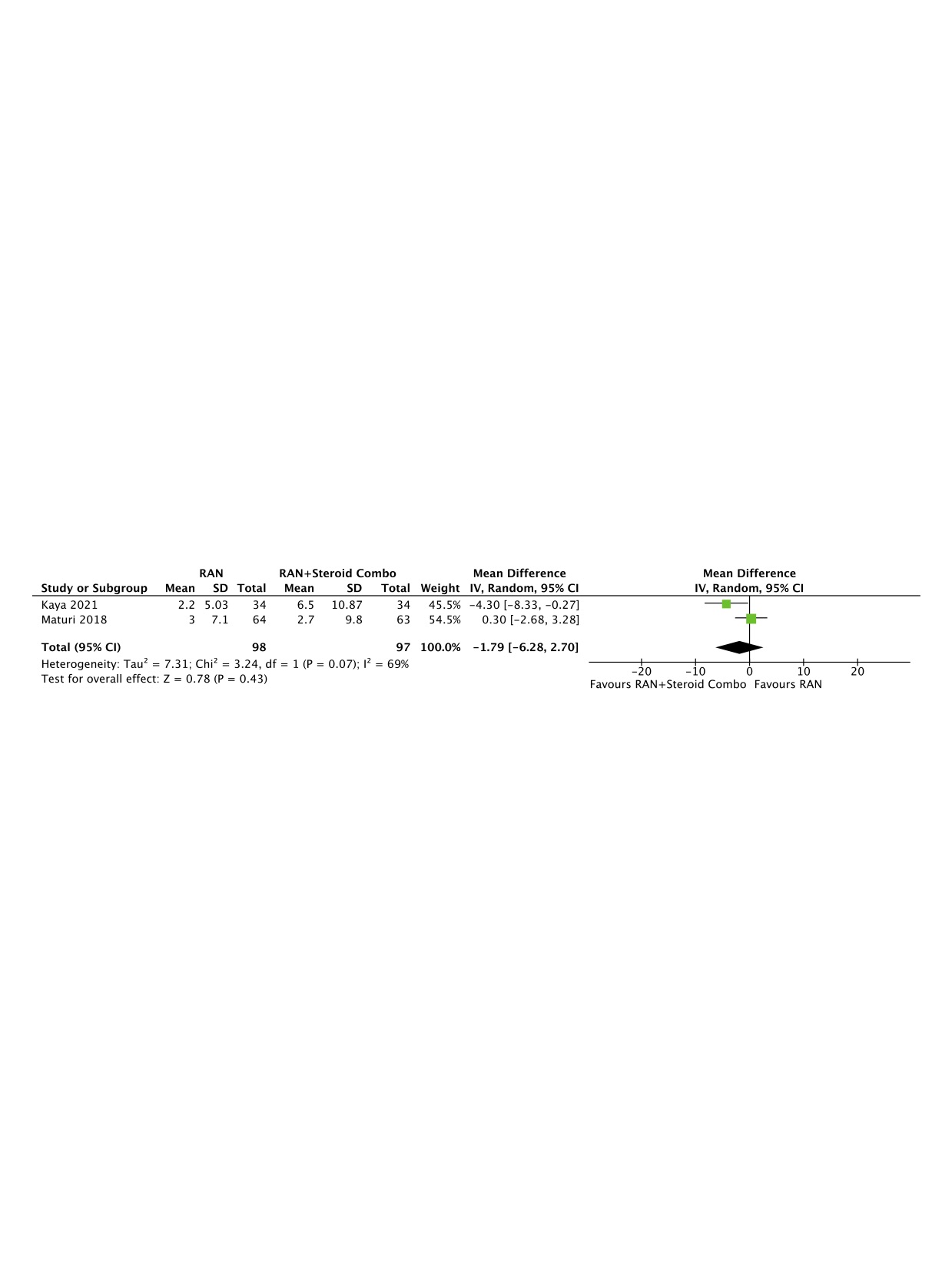

Supplement: sj-zip-1-vrd-10.1177_24741264241280597 – Supplemental material for Anti-VEGF Monotherapy vs Anti-VEGF and Steroid Combination Therapy for Diabetic Macular Edema: A Meta-analysis [file sj-zip-1-vrd-10.1177_24741264241280597.zip › Supplemental Figure 4. a.jpg]

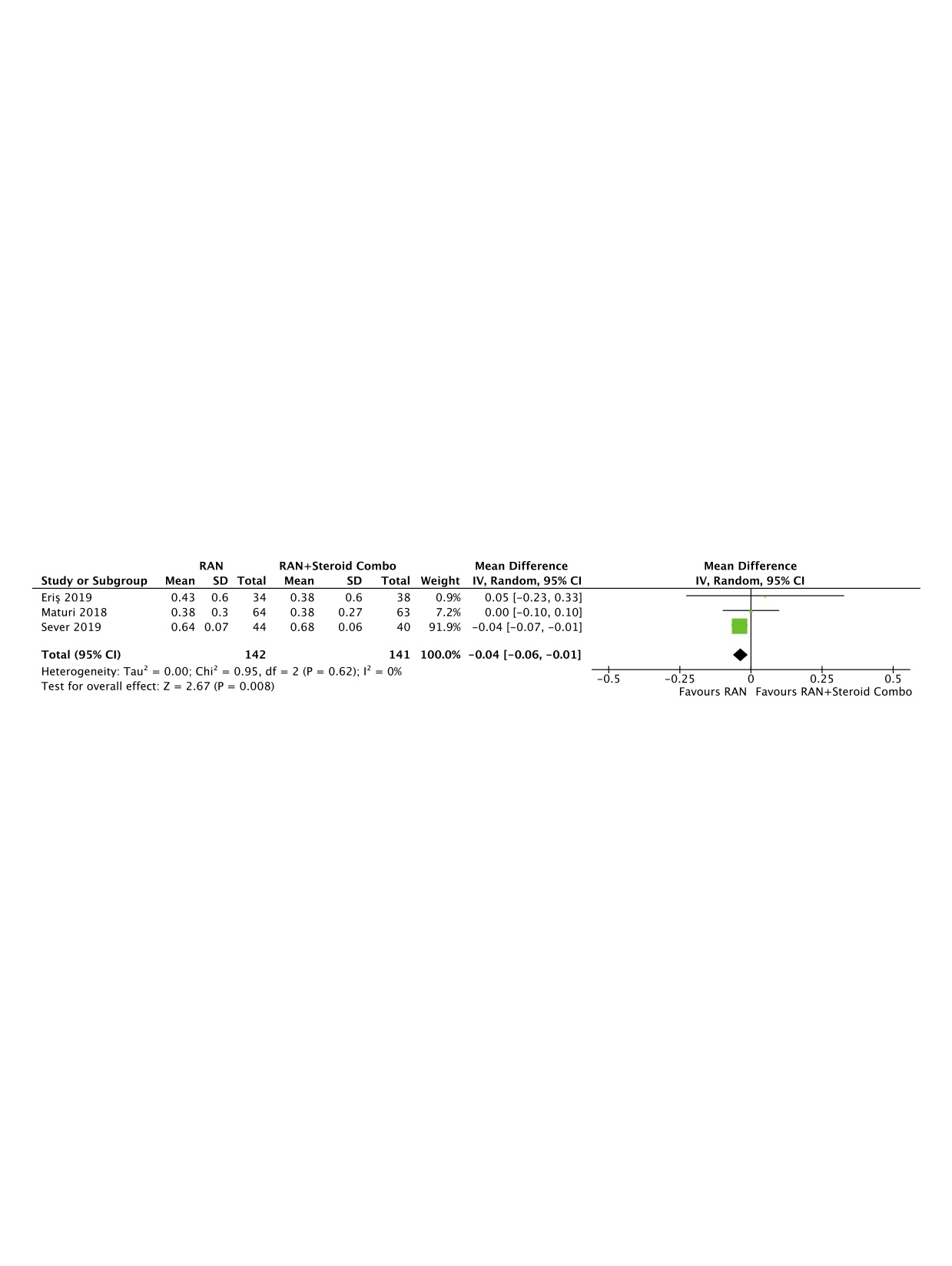

Supplement: sj-zip-1-vrd-10.1177_24741264241280597 – Supplemental material for Anti-VEGF Monotherapy vs Anti-VEGF and Steroid Combination Therapy for Diabetic Macular Edema: A Meta-analysis [file sj-zip-1-vrd-10.1177_24741264241280597.zip › Supplemental Figure 4. b.jpg]

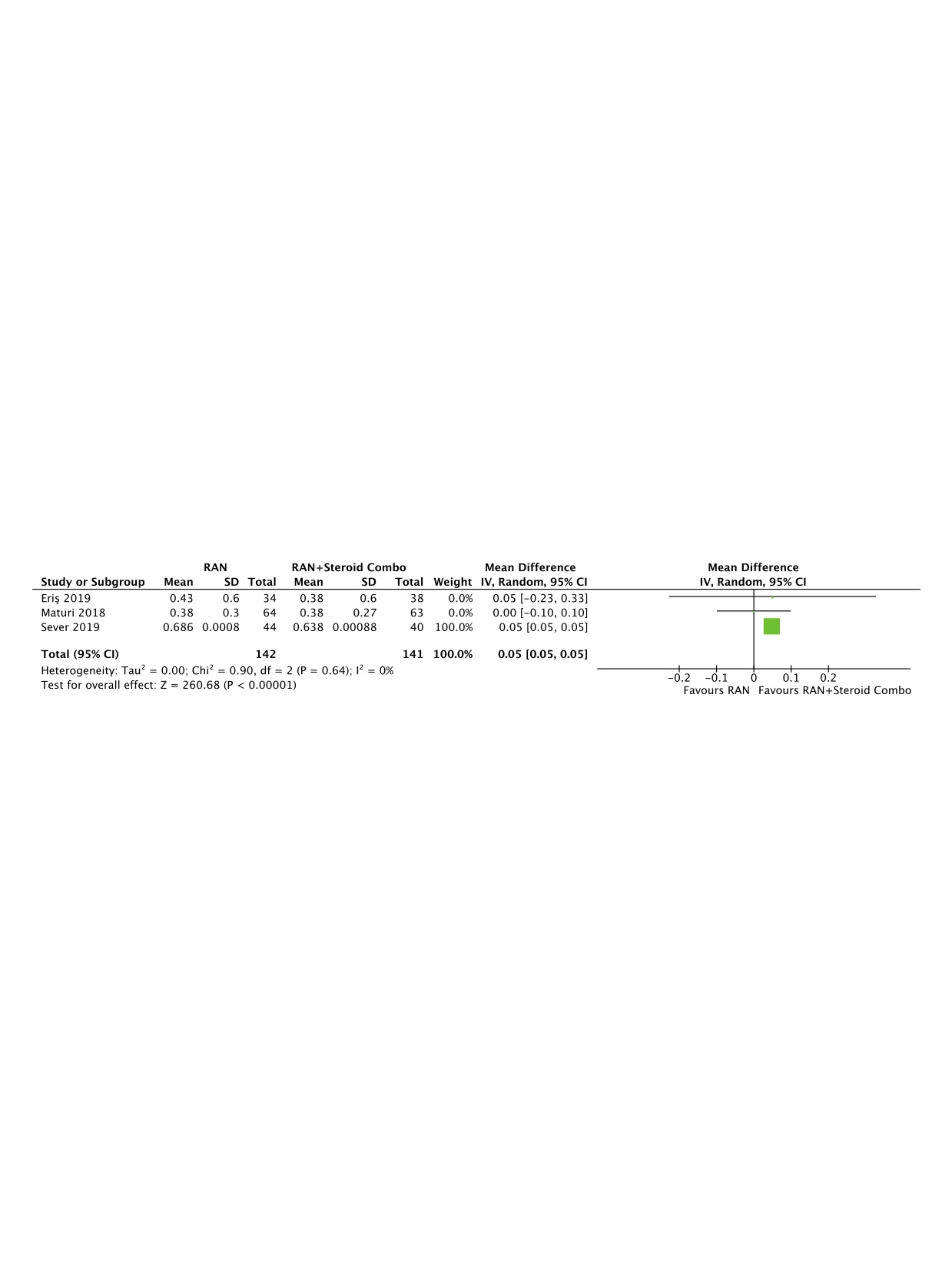

Supplement: sj-zip-1-vrd-10.1177_24741264241280597 – Supplemental material for Anti-VEGF Monotherapy vs Anti-VEGF and Steroid Combination Therapy for Diabetic Macular Edema: A Meta-analysis [file sj-zip-1-vrd-10.1177_24741264241280597.zip › Supplemental Figure 4. d.jpg]

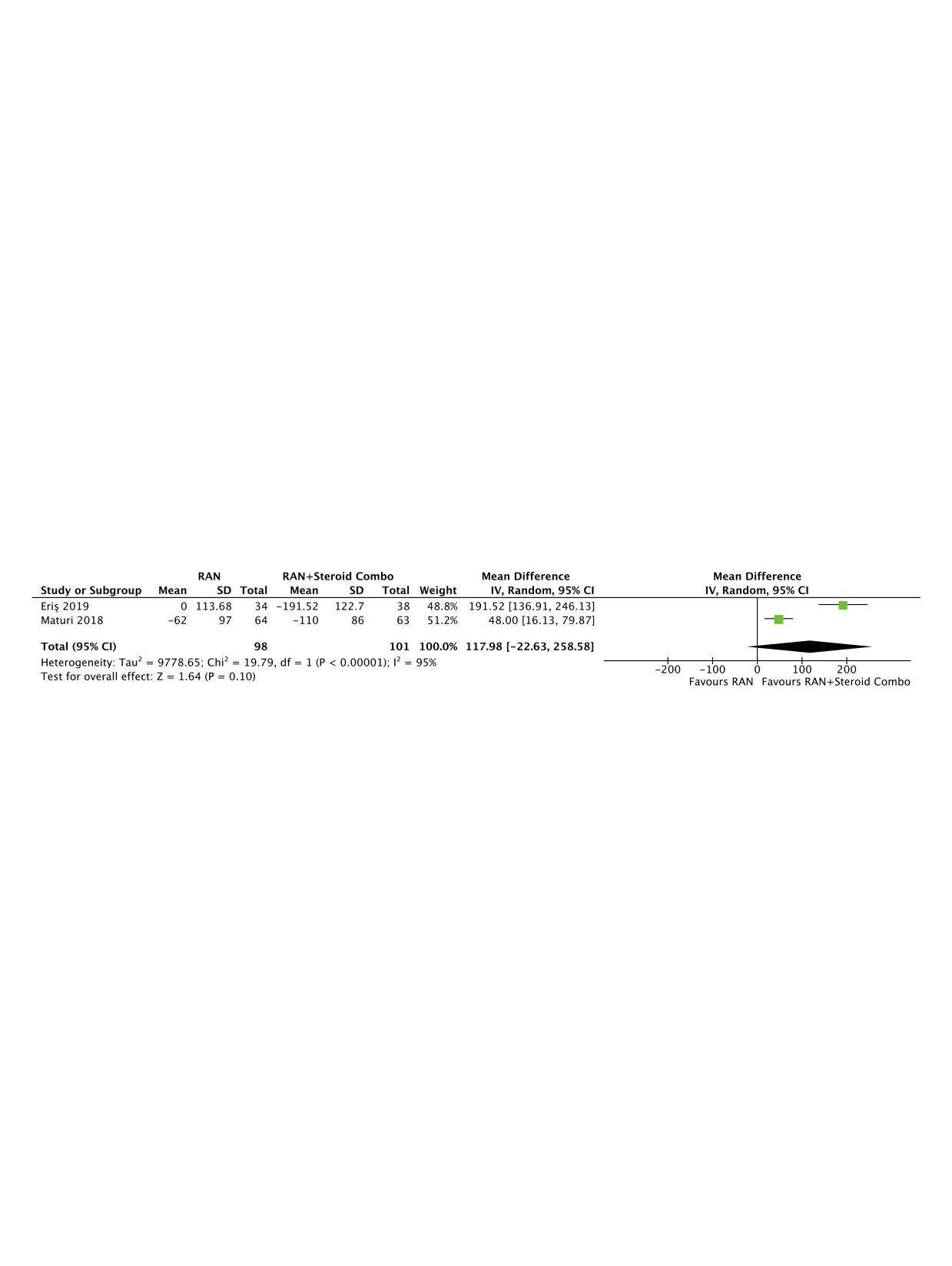

Supplement: sj-zip-1-vrd-10.1177_24741264241280597 – Supplemental material for Anti-VEGF Monotherapy vs Anti-VEGF and Steroid Combination Therapy for Diabetic Macular Edema: A Meta-analysis [file sj-zip-1-vrd-10.1177_24741264241280597.zip › Supplemental Figure 4. e.jpg]

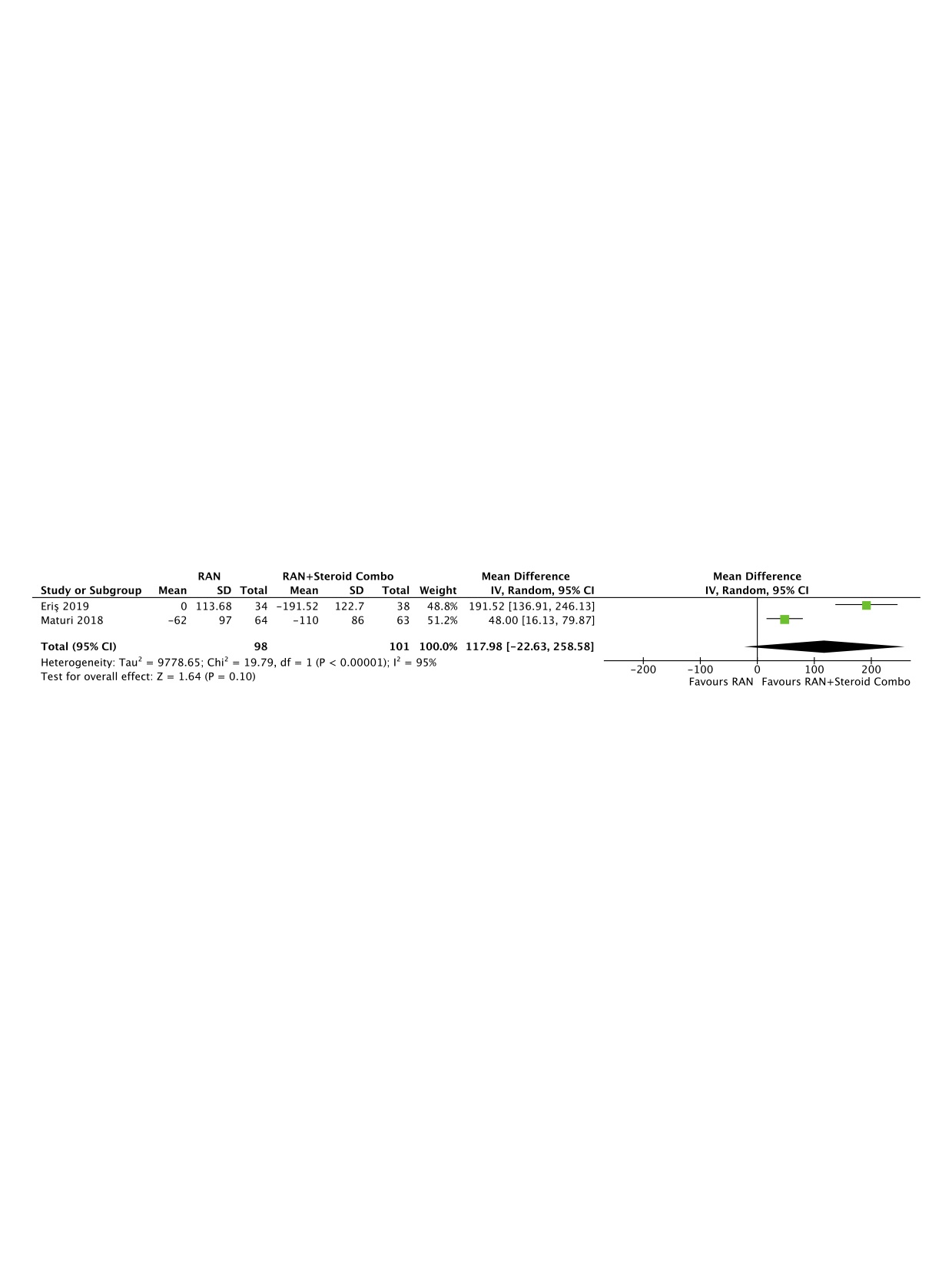

Supplement: sj-zip-1-vrd-10.1177_24741264241280597 – Supplemental material for Anti-VEGF Monotherapy vs Anti-VEGF and Steroid Combination Therapy for Diabetic Macular Edema: A Meta-analysis [file sj-zip-1-vrd-10.1177_24741264241280597.zip › Supplemental Figure 4. f.jpg]

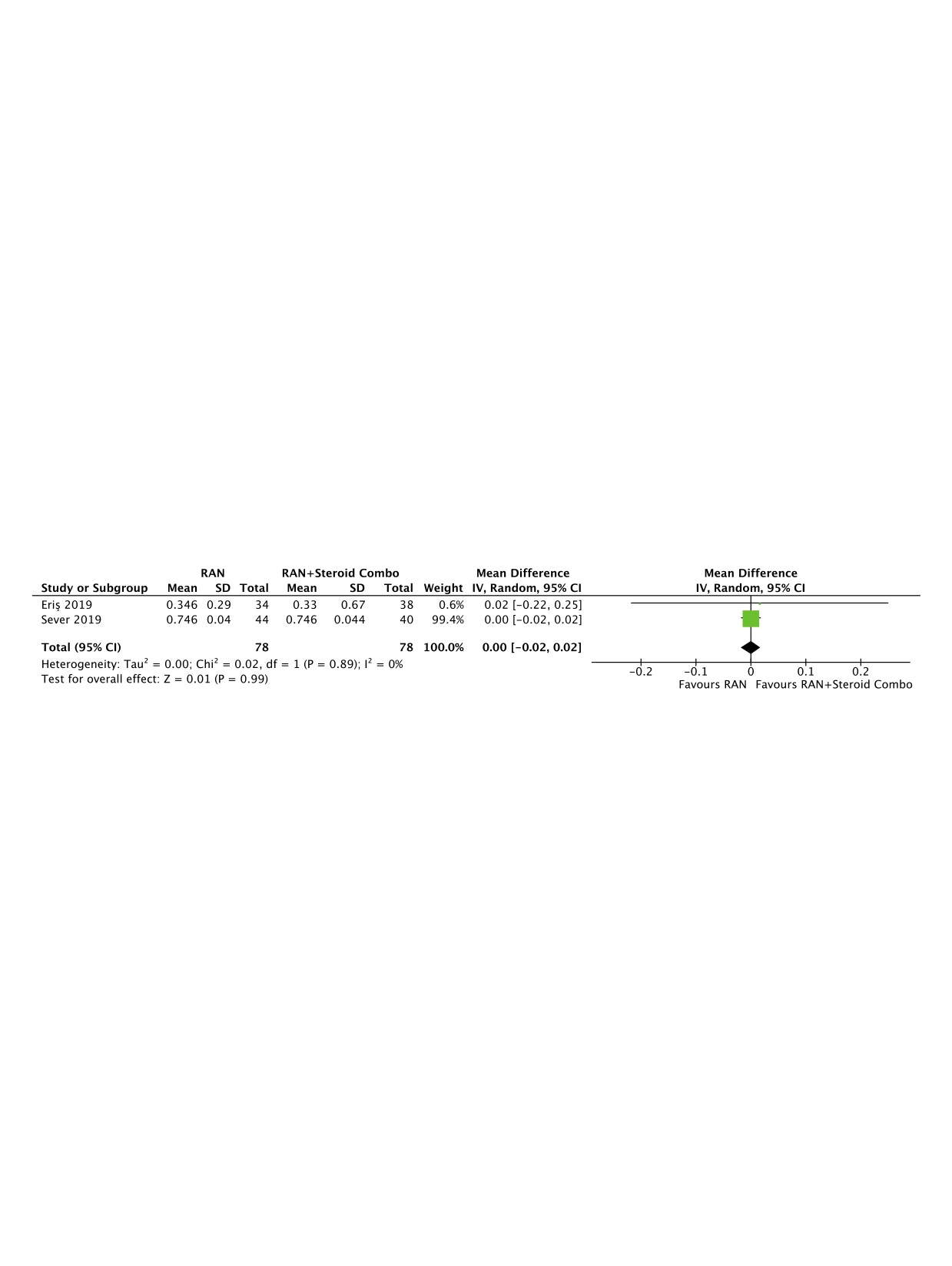

Supplement: sj-zip-1-vrd-10.1177_24741264241280597 – Supplemental material for Anti-VEGF Monotherapy vs Anti-VEGF and Steroid Combination Therapy for Diabetic Macular Edema: A Meta-analysis [file sj-zip-1-vrd-10.1177_24741264241280597.zip › Supplemental Figure 4. c.jpg]

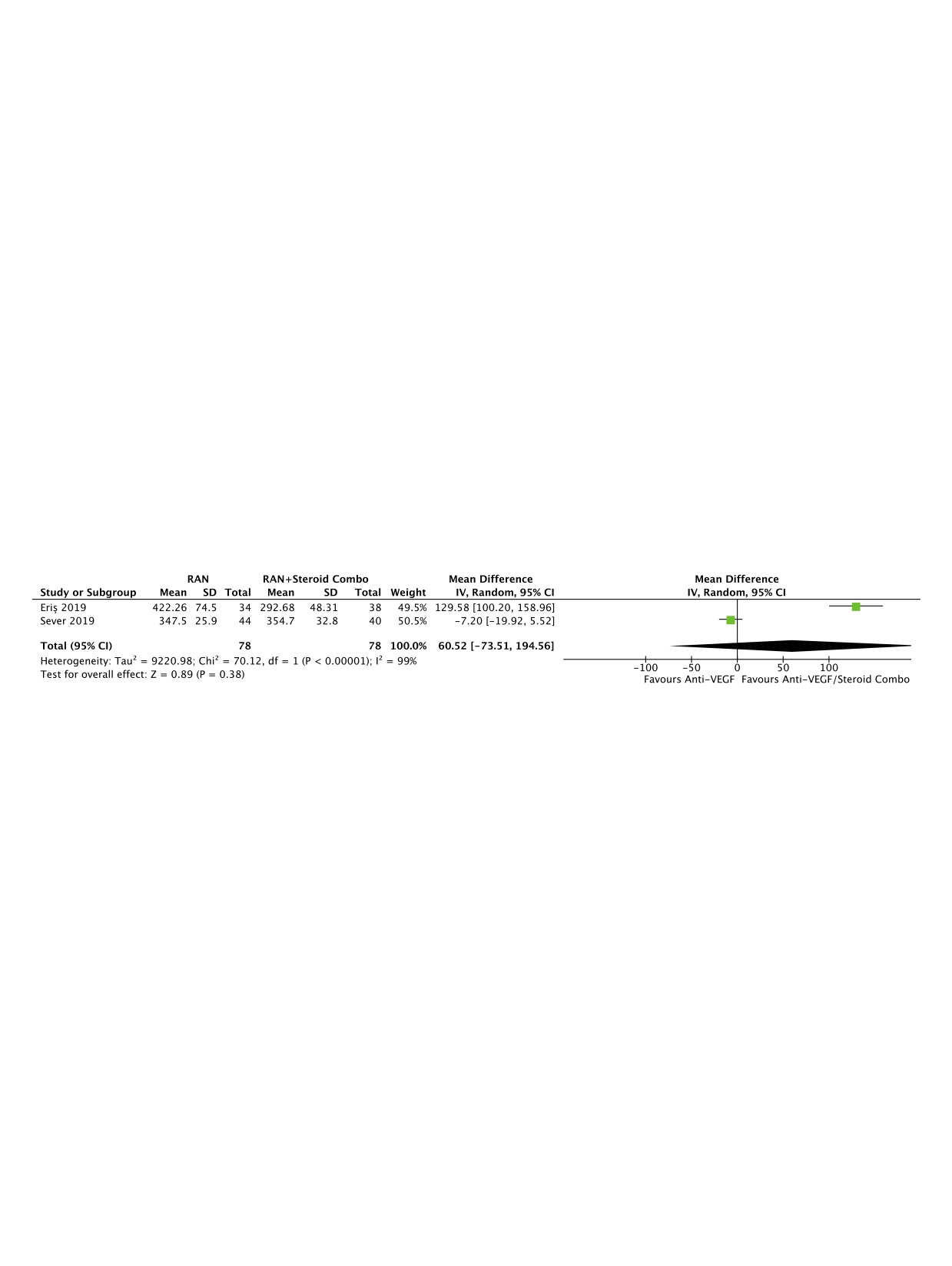

Supplement: sj-zip-1-vrd-10.1177_24741264241280597 – Supplemental material for Anti-VEGF Monotherapy vs Anti-VEGF and Steroid Combination Therapy for Diabetic Macular Edema: A Meta-analysis [file sj-zip-1-vrd-10.1177_24741264241280597.zip › Supplemental Figure 4. h.jpg]

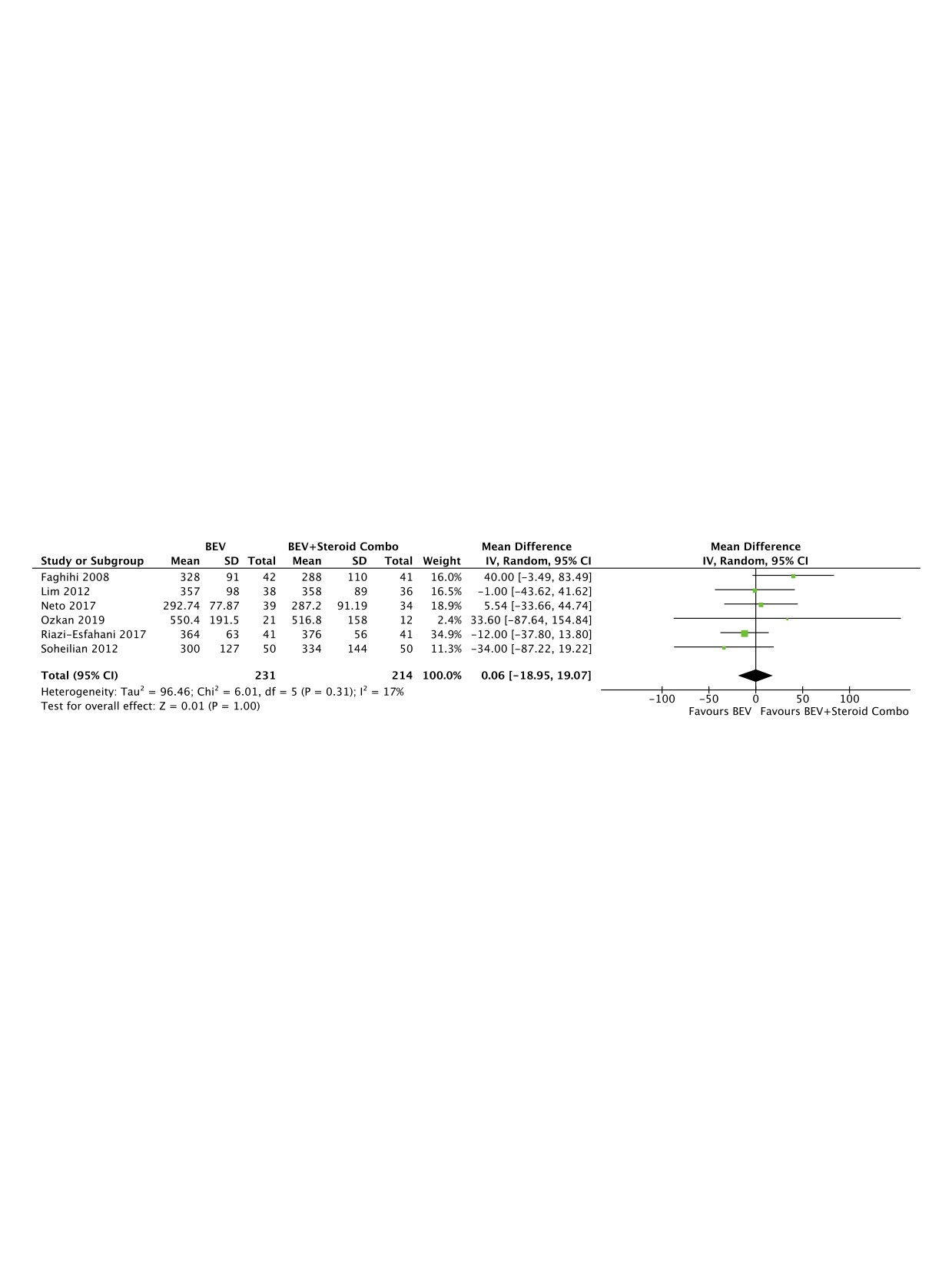

Supplement: sj-zip-1-vrd-10.1177_24741264241280597 – Supplemental material for Anti-VEGF Monotherapy vs Anti-VEGF and Steroid Combination Therapy for Diabetic Macular Edema: A Meta-analysis [file sj-zip-1-vrd-10.1177_24741264241280597.zip › Supplemental Figure 3. r.jpg]

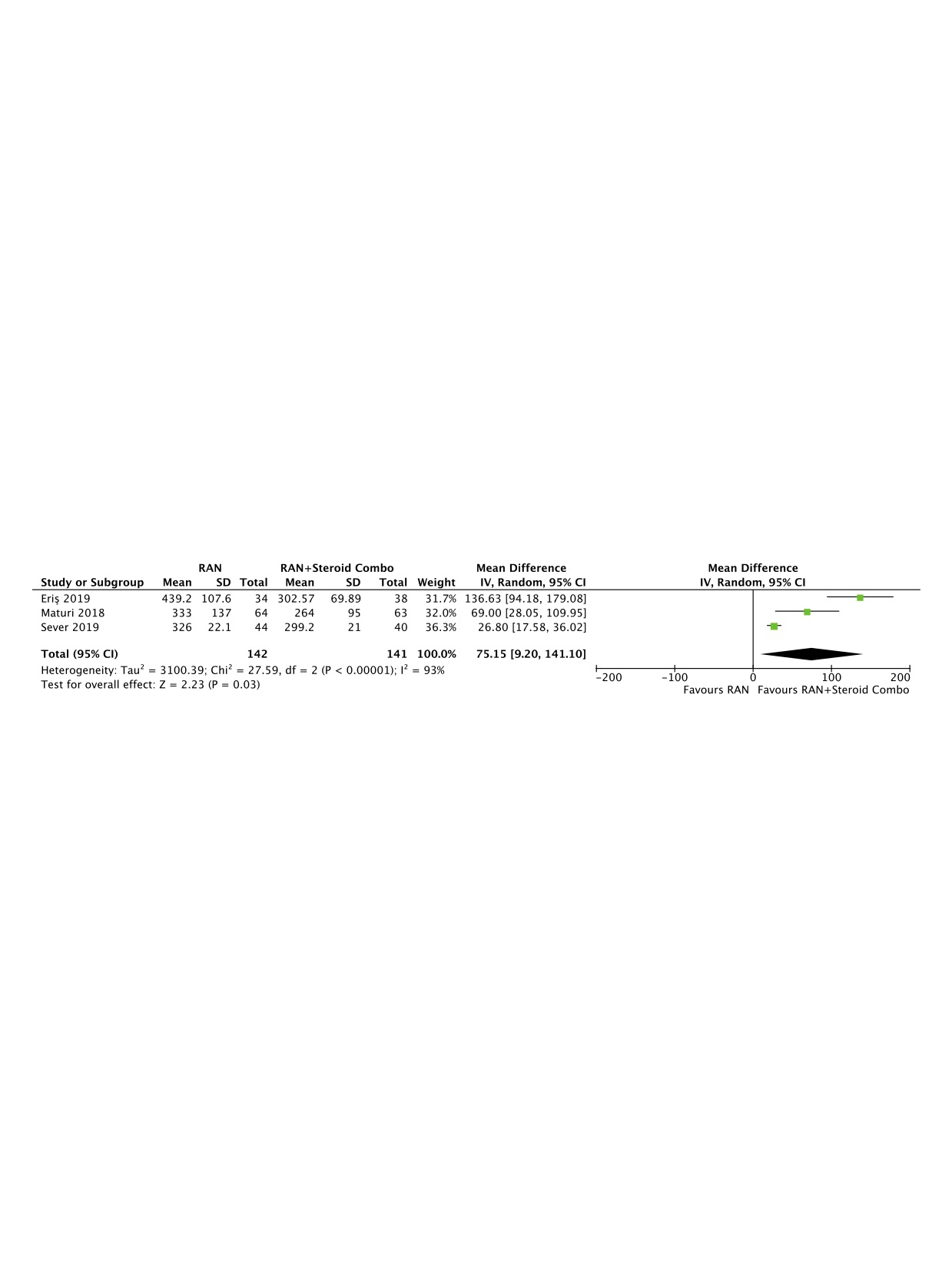

Supplement: sj-zip-1-vrd-10.1177_24741264241280597 – Supplemental material for Anti-VEGF Monotherapy vs Anti-VEGF and Steroid Combination Therapy for Diabetic Macular Edema: A Meta-analysis [file sj-zip-1-vrd-10.1177_24741264241280597.zip › Supplemental Figure 4. i.jpg]

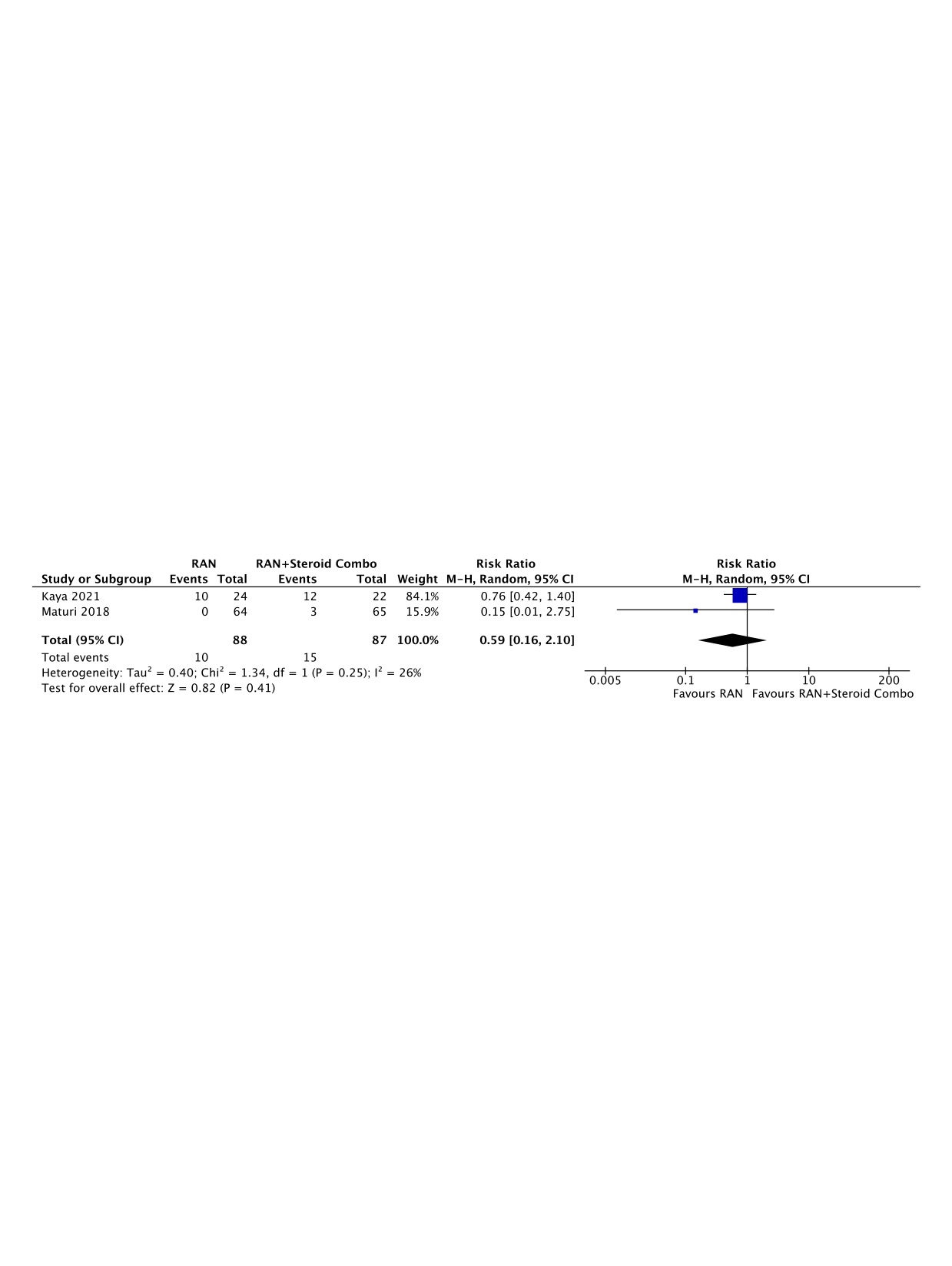

Supplement: sj-zip-1-vrd-10.1177_24741264241280597 – Supplemental material for Anti-VEGF Monotherapy vs Anti-VEGF and Steroid Combination Therapy for Diabetic Macular Edema: A Meta-analysis [file sj-zip-1-vrd-10.1177_24741264241280597.zip › Supplemental Figure 4. k.jpg]

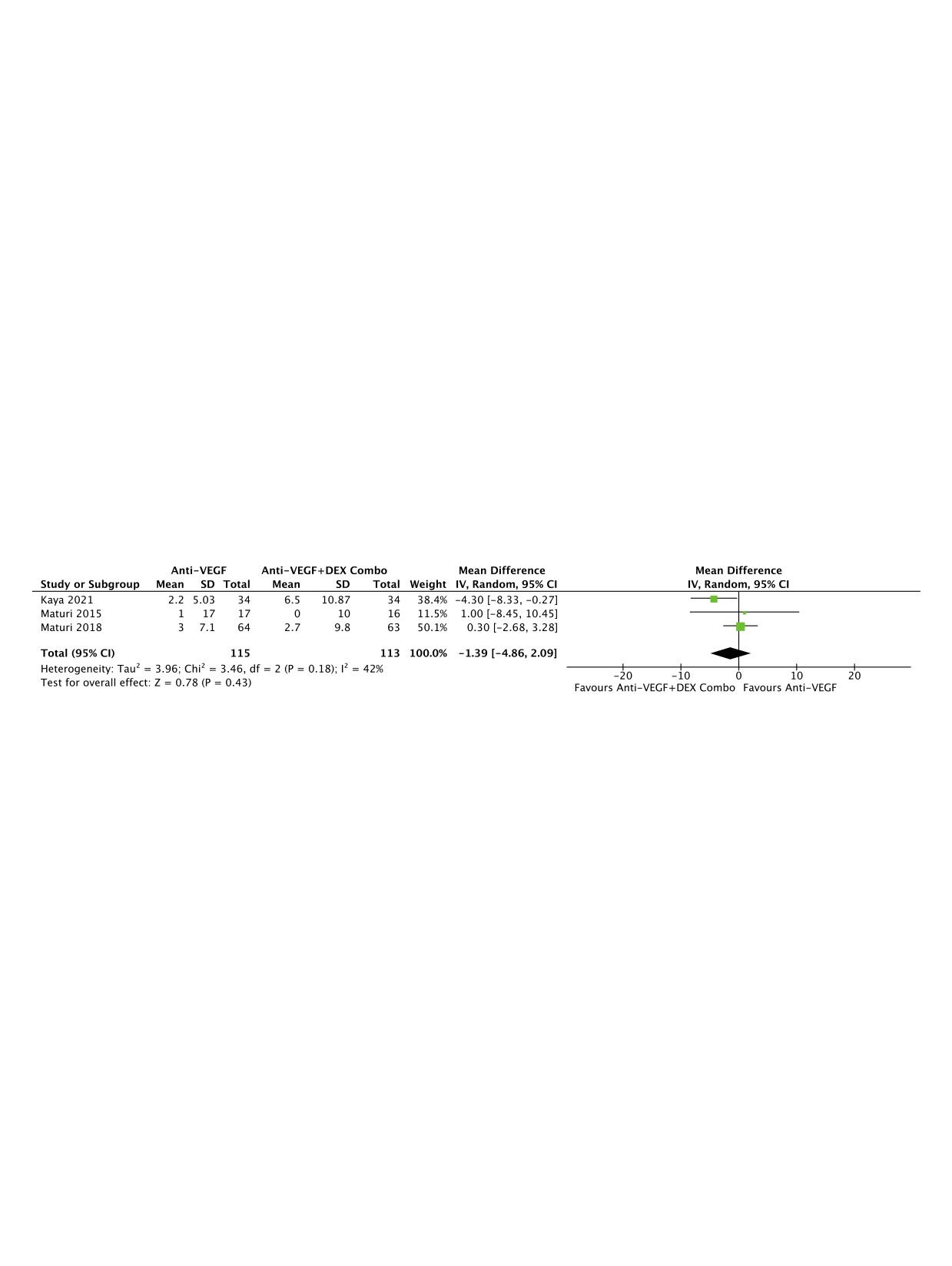

Supplement: sj-zip-1-vrd-10.1177_24741264241280597 – Supplemental material for Anti-VEGF Monotherapy vs Anti-VEGF and Steroid Combination Therapy for Diabetic Macular Edema: A Meta-analysis [file sj-zip-1-vrd-10.1177_24741264241280597.zip › Supplemental Figure 5. a.jpg]

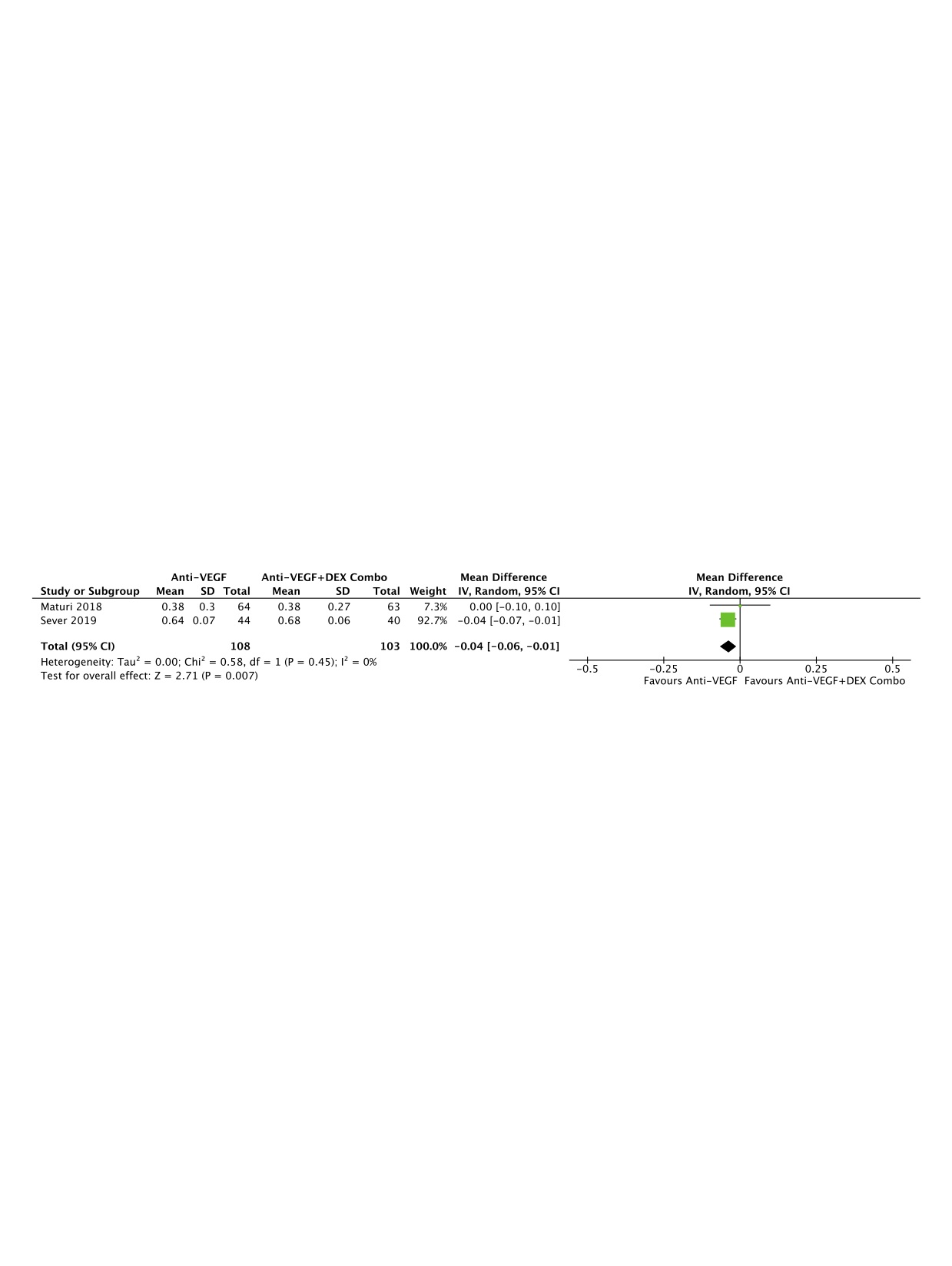

Supplement: sj-zip-1-vrd-10.1177_24741264241280597 – Supplemental material for Anti-VEGF Monotherapy vs Anti-VEGF and Steroid Combination Therapy for Diabetic Macular Edema: A Meta-analysis [file sj-zip-1-vrd-10.1177_24741264241280597.zip › Supplemental Figure 5. c.jpg]

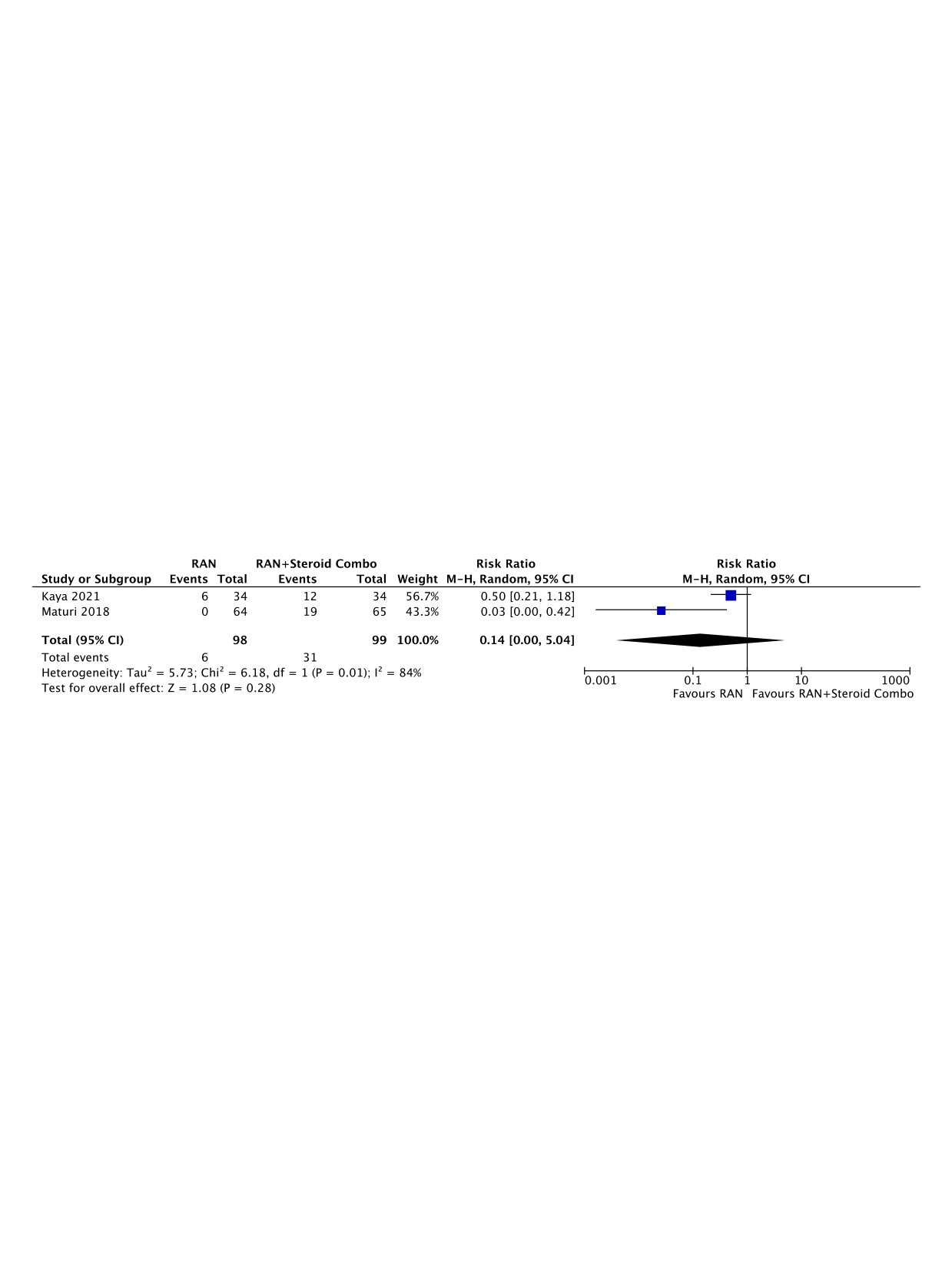

Supplement: sj-zip-1-vrd-10.1177_24741264241280597 – Supplemental material for Anti-VEGF Monotherapy vs Anti-VEGF and Steroid Combination Therapy for Diabetic Macular Edema: A Meta-analysis [file sj-zip-1-vrd-10.1177_24741264241280597.zip › Supplemental Figure 4. j.jpg]

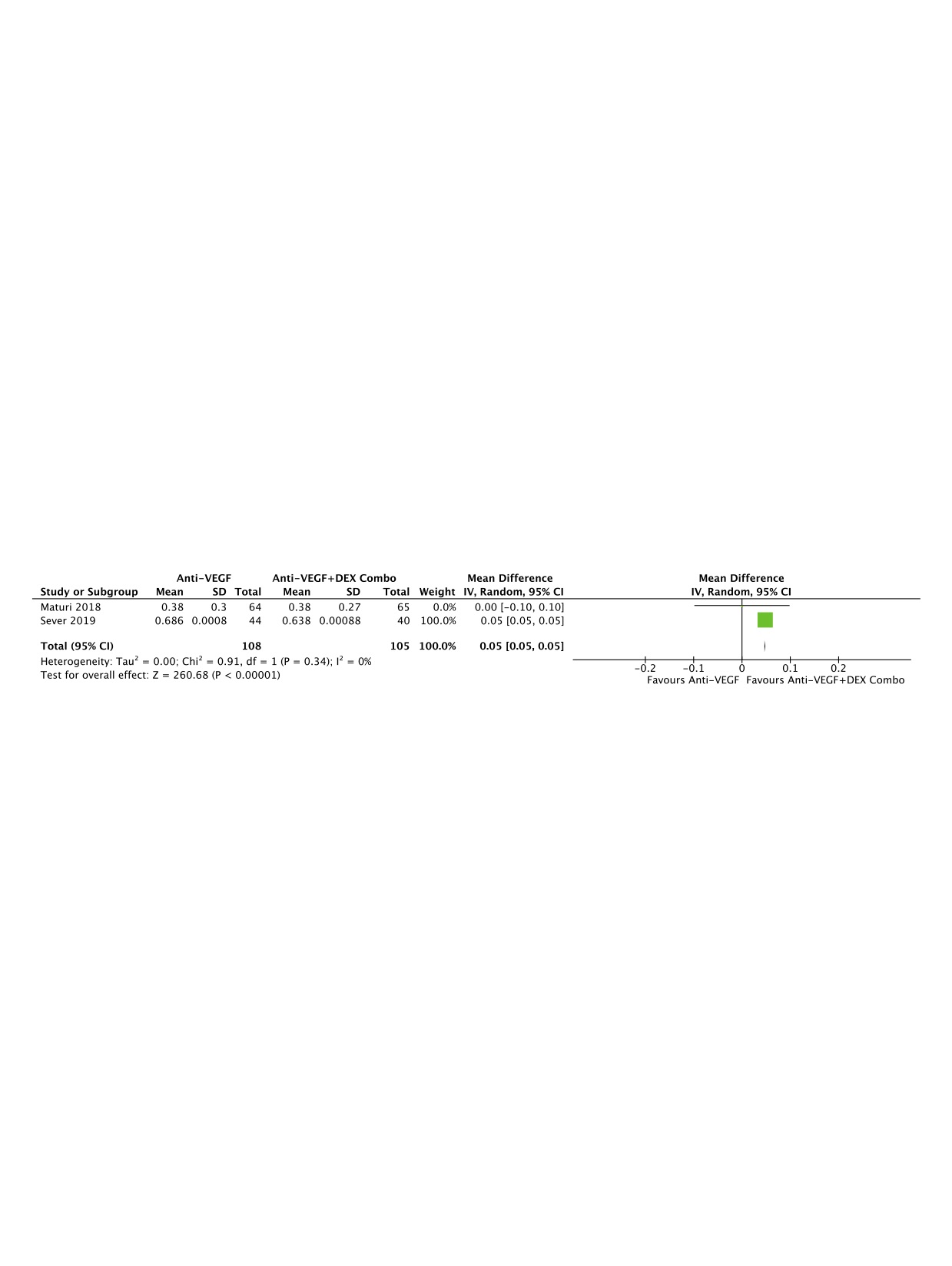

Supplement: sj-zip-1-vrd-10.1177_24741264241280597 – Supplemental material for Anti-VEGF Monotherapy vs Anti-VEGF and Steroid Combination Therapy for Diabetic Macular Edema: A Meta-analysis [file sj-zip-1-vrd-10.1177_24741264241280597.zip › Supplemental Figure 5. d.jpg]

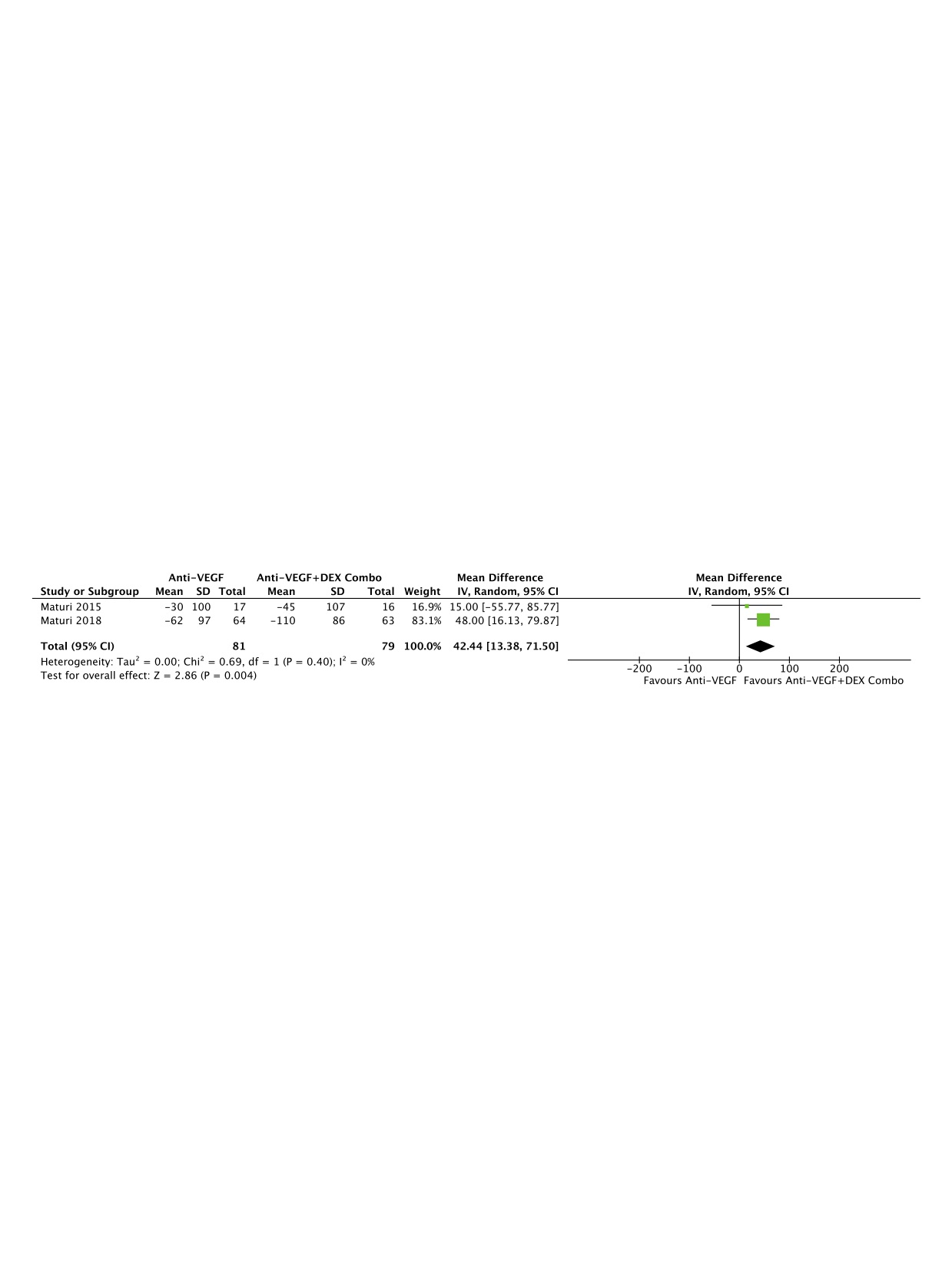

Supplement: sj-zip-1-vrd-10.1177_24741264241280597 – Supplemental material for Anti-VEGF Monotherapy vs Anti-VEGF and Steroid Combination Therapy for Diabetic Macular Edema: A Meta-analysis [file sj-zip-1-vrd-10.1177_24741264241280597.zip › Supplemental Figure 5. e.jpg]

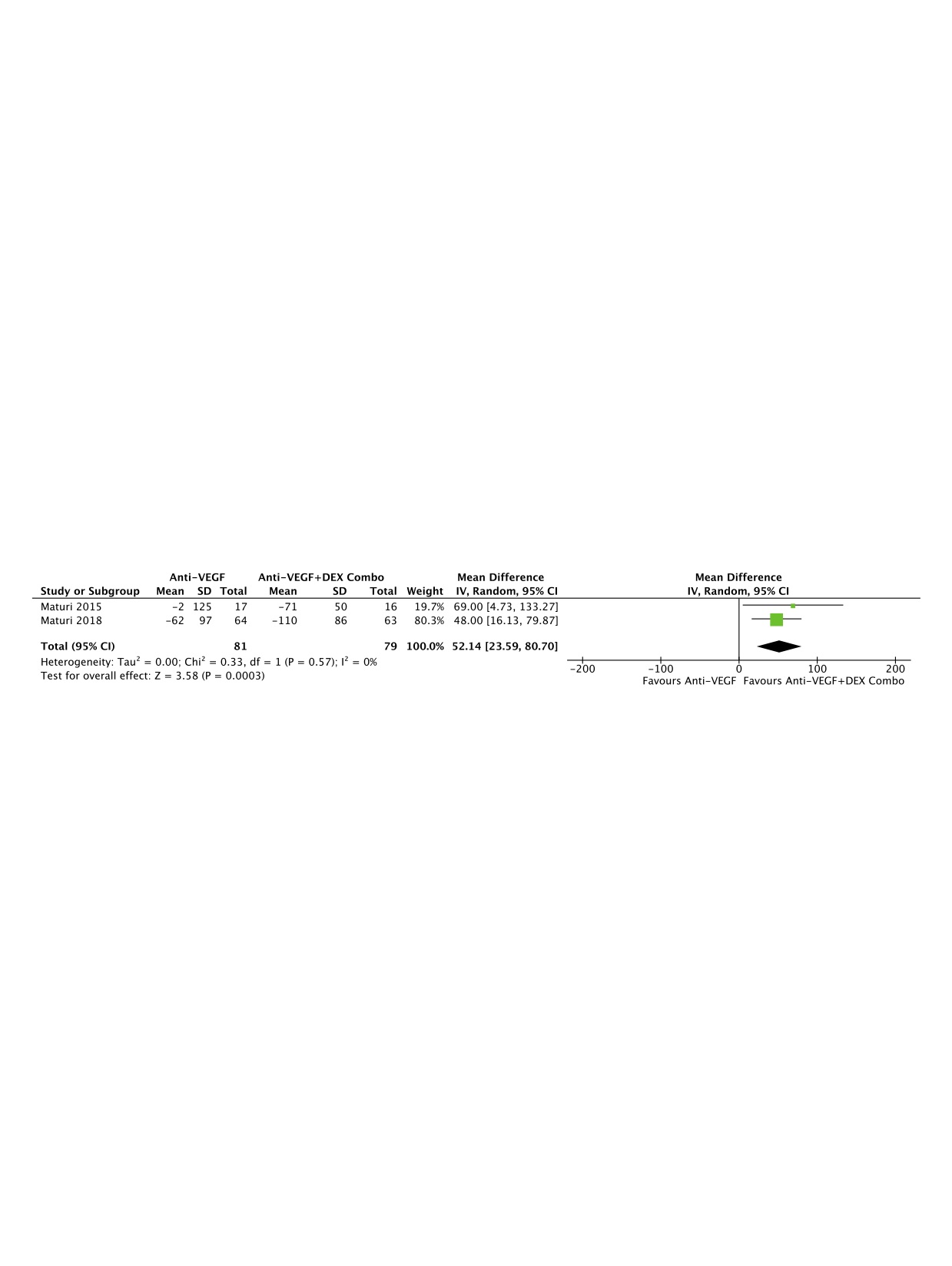

Supplement: sj-zip-1-vrd-10.1177_24741264241280597 – Supplemental material for Anti-VEGF Monotherapy vs Anti-VEGF and Steroid Combination Therapy for Diabetic Macular Edema: A Meta-analysis [file sj-zip-1-vrd-10.1177_24741264241280597.zip › Supplemental Figure 5. f.jpg]

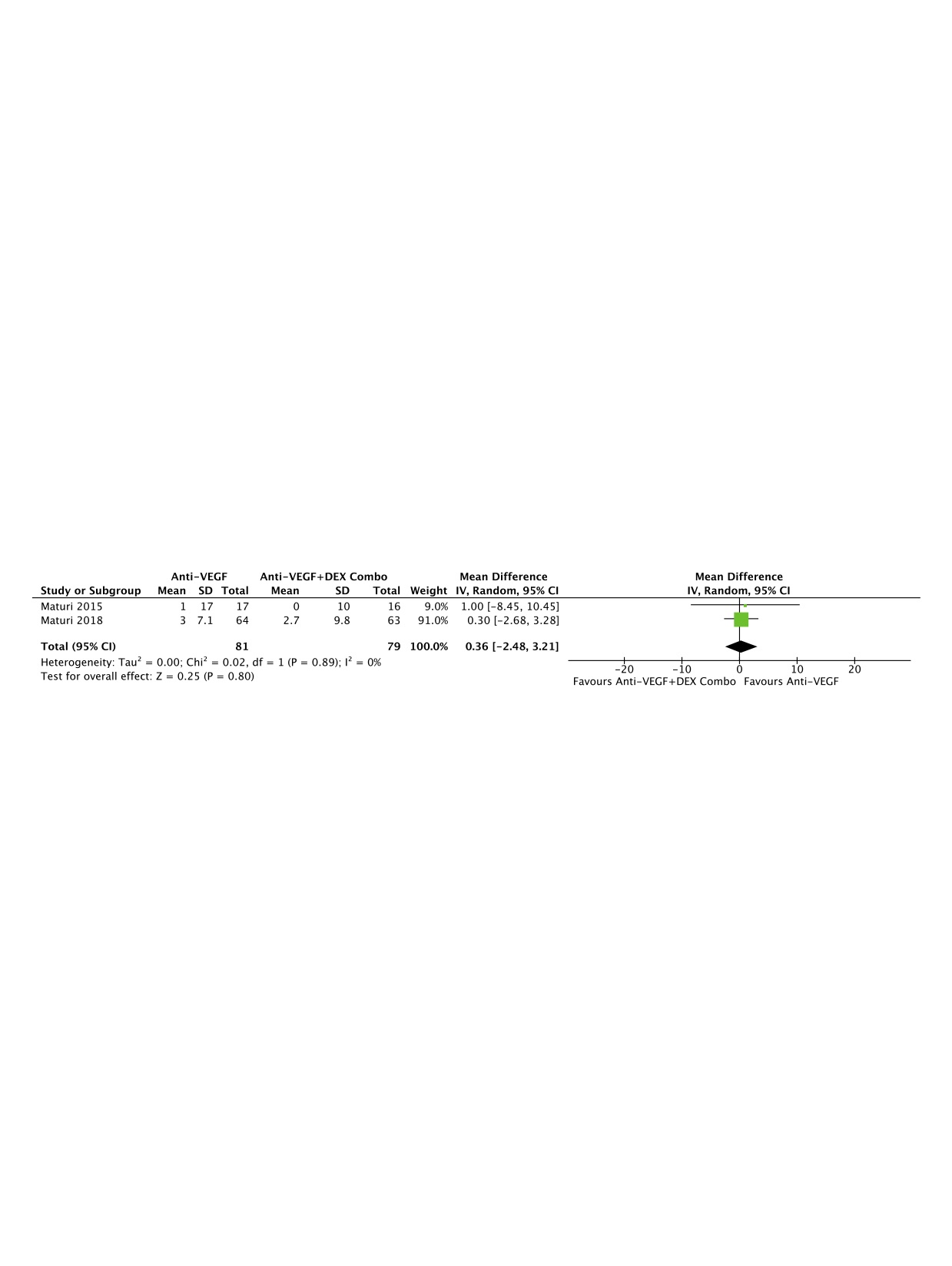

Supplement: sj-zip-1-vrd-10.1177_24741264241280597 – Supplemental material for Anti-VEGF Monotherapy vs Anti-VEGF and Steroid Combination Therapy for Diabetic Macular Edema: A Meta-analysis [file sj-zip-1-vrd-10.1177_24741264241280597.zip › Supplemental Figure 5. b.jpg]

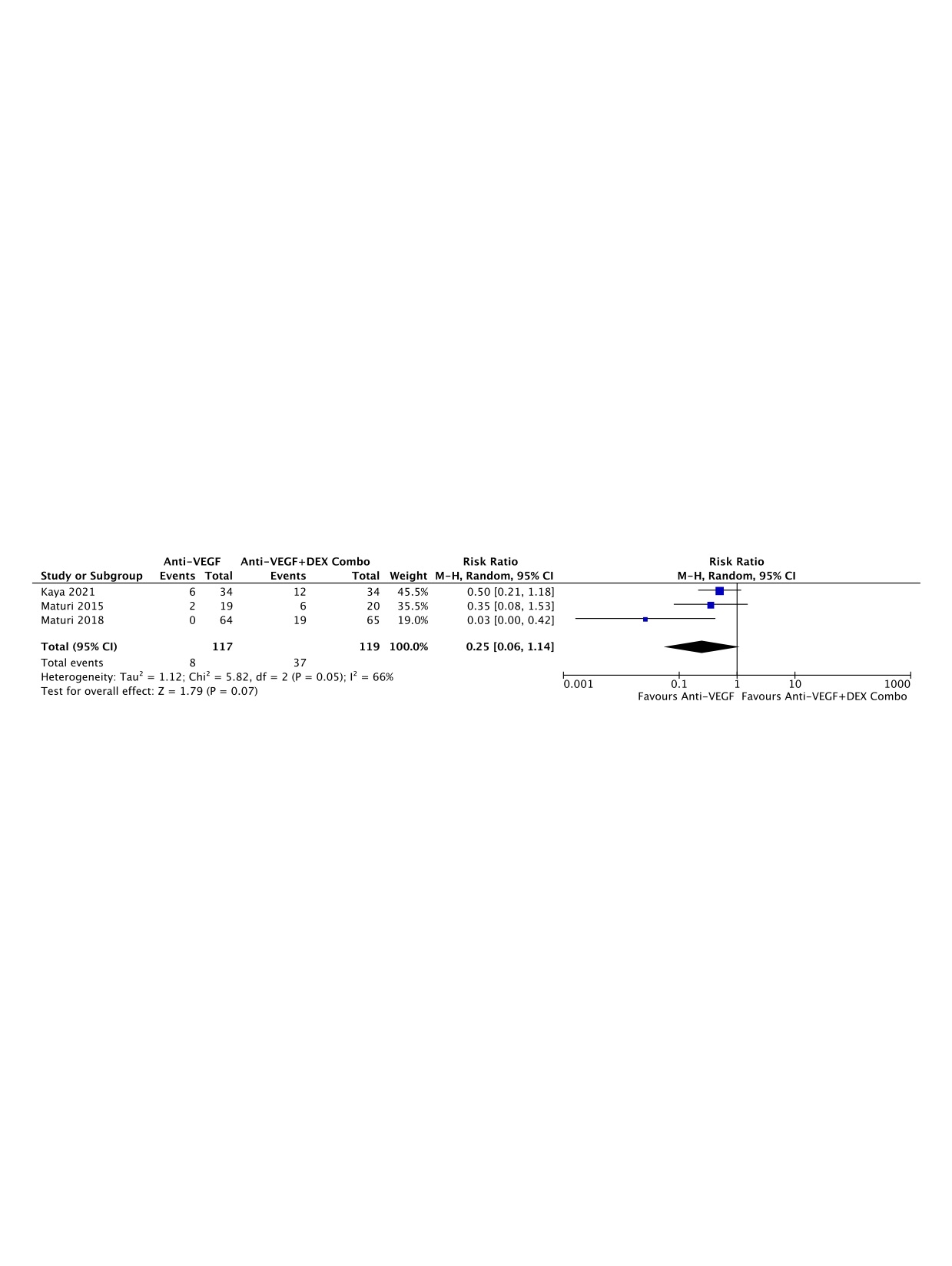

Supplement: sj-zip-1-vrd-10.1177_24741264241280597 – Supplemental material for Anti-VEGF Monotherapy vs Anti-VEGF and Steroid Combination Therapy for Diabetic Macular Edema: A Meta-analysis [file sj-zip-1-vrd-10.1177_24741264241280597.zip › Supplemental Figure 5. i.jpg]

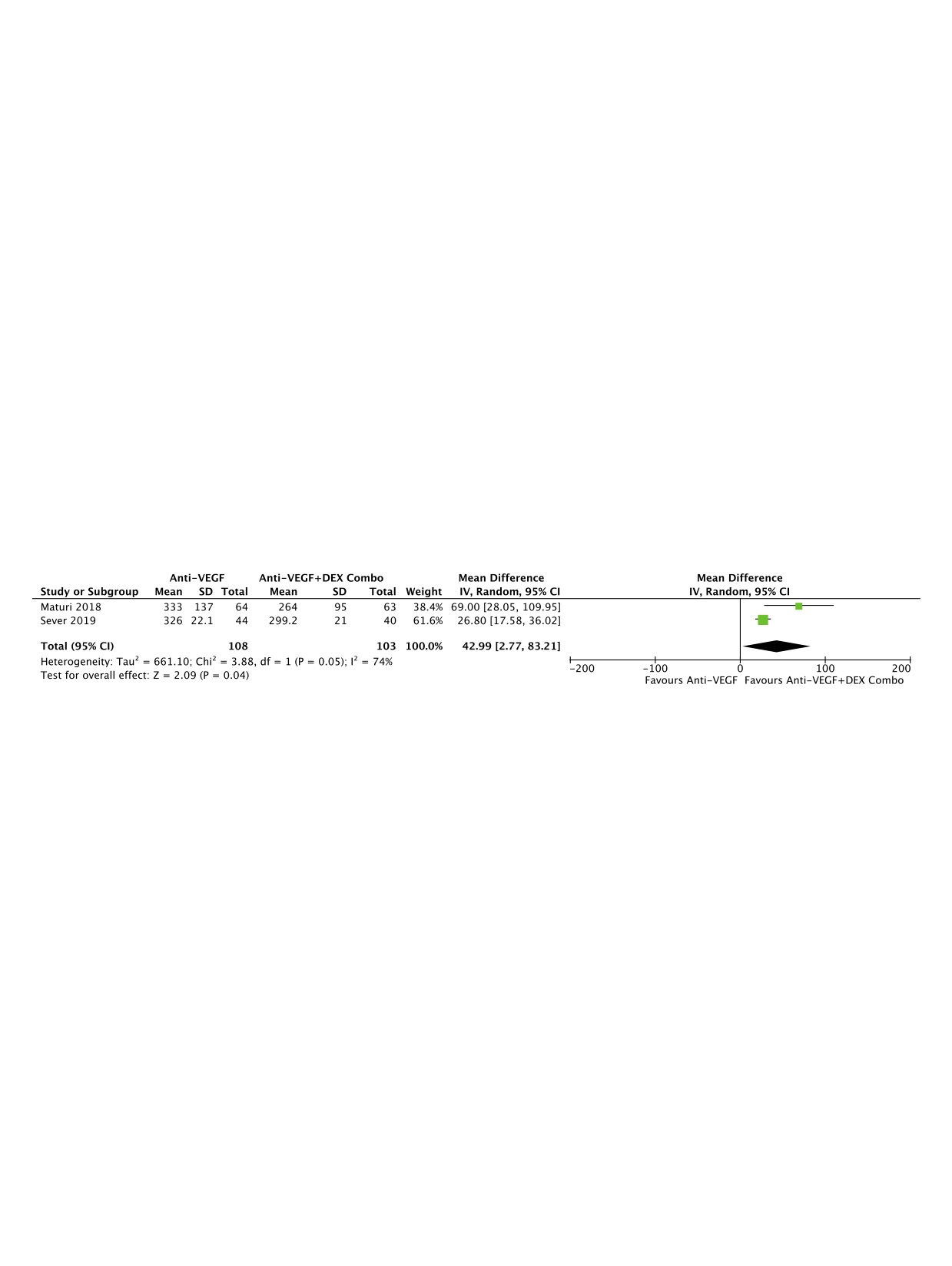

Supplement: sj-zip-1-vrd-10.1177_24741264241280597 – Supplemental material for Anti-VEGF Monotherapy vs Anti-VEGF and Steroid Combination Therapy for Diabetic Macular Edema: A Meta-analysis [file sj-zip-1-vrd-10.1177_24741264241280597.zip › Supplemental Figure 5. h.jpg]

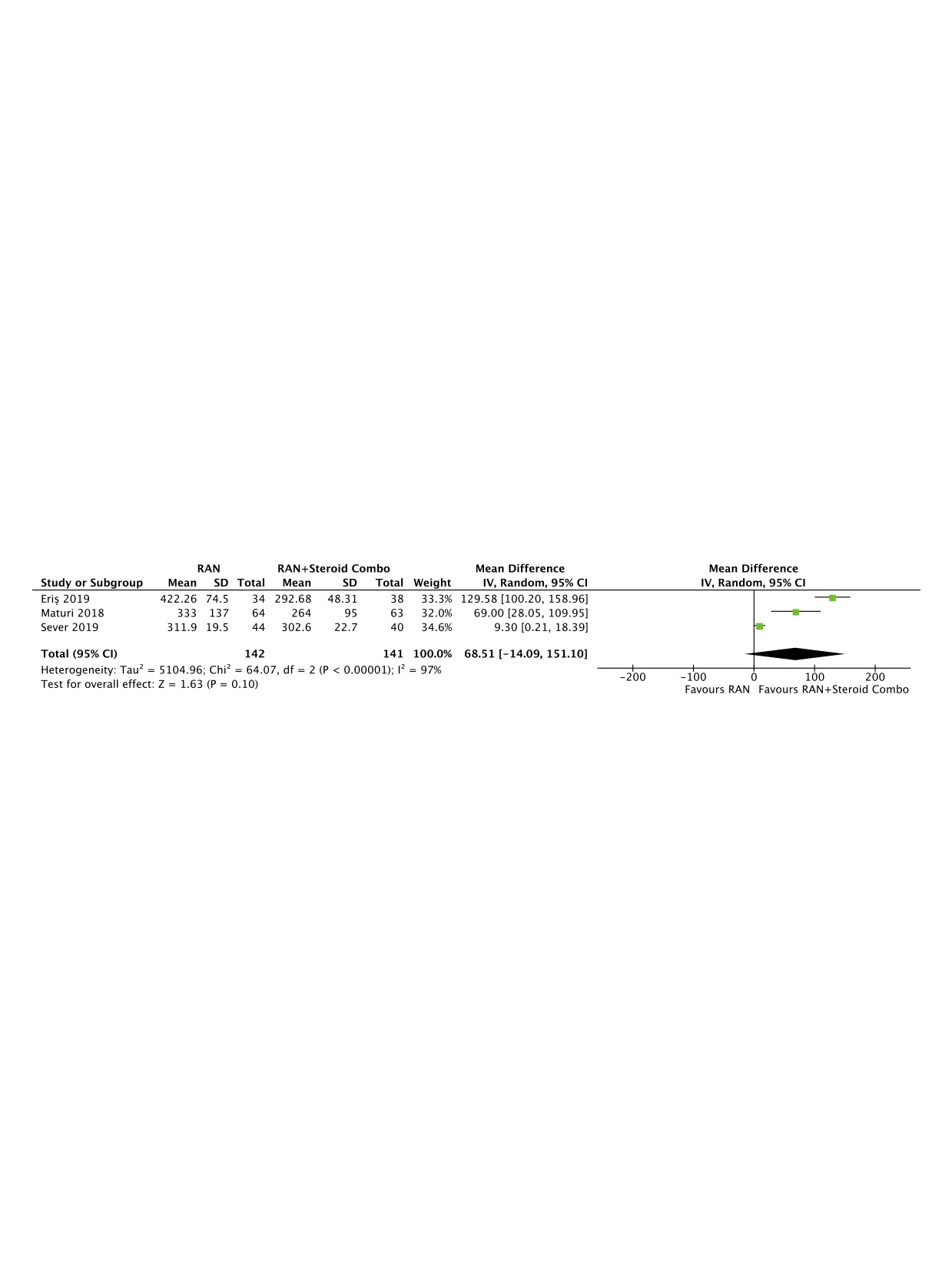

Supplement: sj-zip-1-vrd-10.1177_24741264241280597 – Supplemental material for Anti-VEGF Monotherapy vs Anti-VEGF and Steroid Combination Therapy for Diabetic Macular Edema: A Meta-analysis [file sj-zip-1-vrd-10.1177_24741264241280597.zip › Supplemental Figure 4. g.jpg]

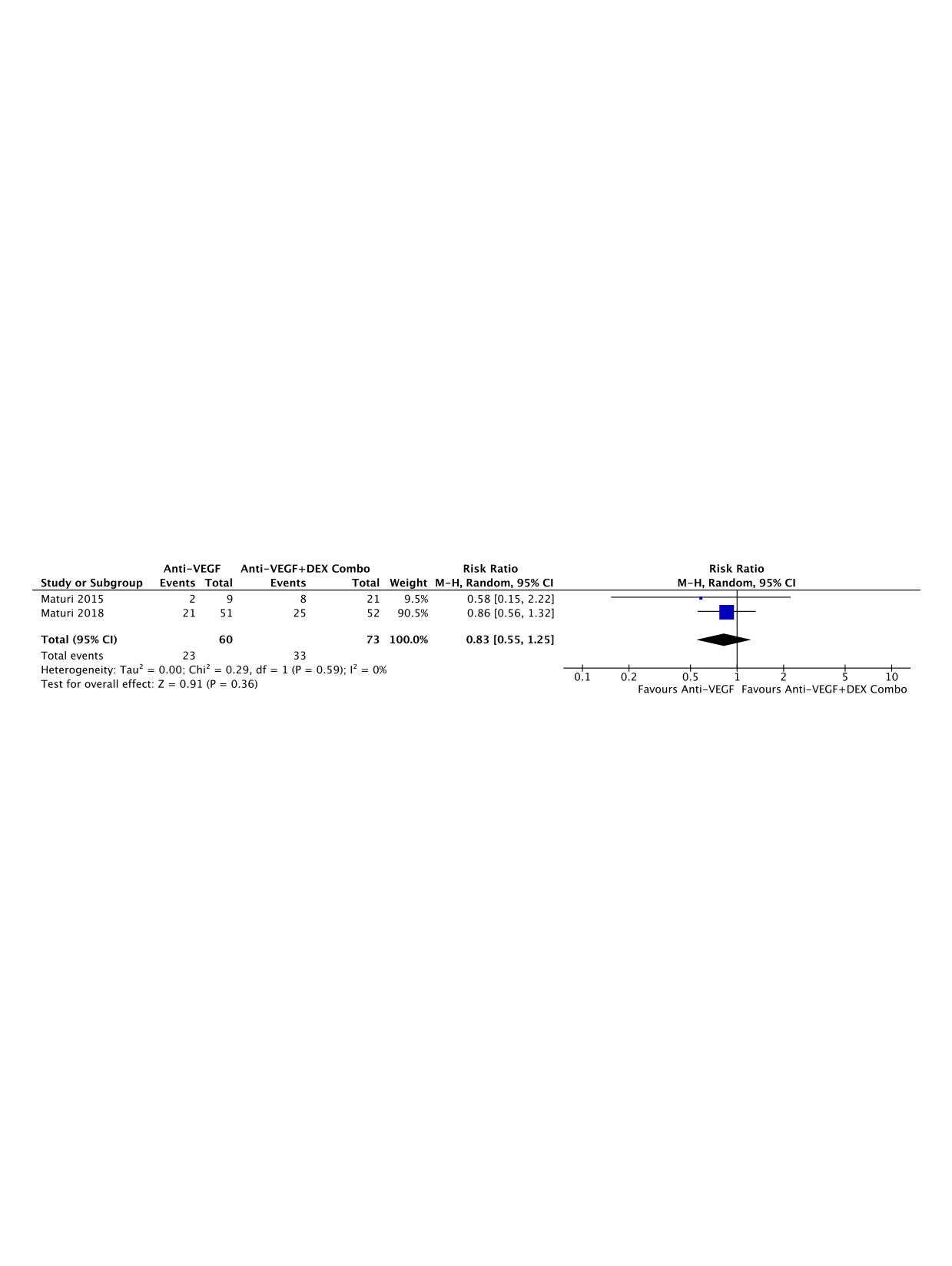

Supplement: sj-zip-1-vrd-10.1177_24741264241280597 – Supplemental material for Anti-VEGF Monotherapy vs Anti-VEGF and Steroid Combination Therapy for Diabetic Macular Edema: A Meta-analysis [file sj-zip-1-vrd-10.1177_24741264241280597.zip › Supplemental Figure 5. j.jpg]

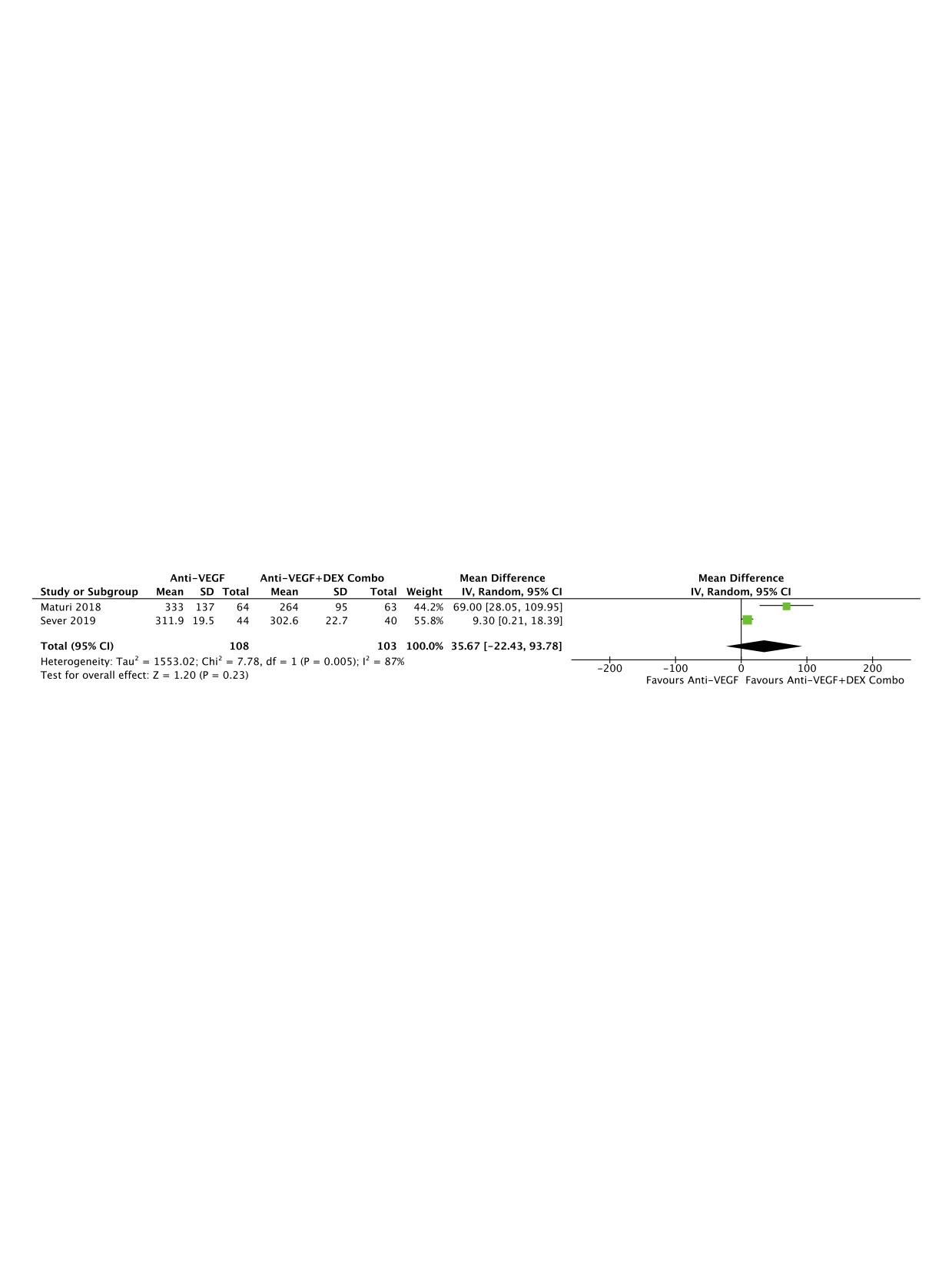

Supplement: sj-zip-1-vrd-10.1177_24741264241280597 – Supplemental material for Anti-VEGF Monotherapy vs Anti-VEGF and Steroid Combination Therapy for Diabetic Macular Edema: A Meta-analysis [file sj-zip-1-vrd-10.1177_24741264241280597.zip › Supplemental Figure 5. g.jpg]

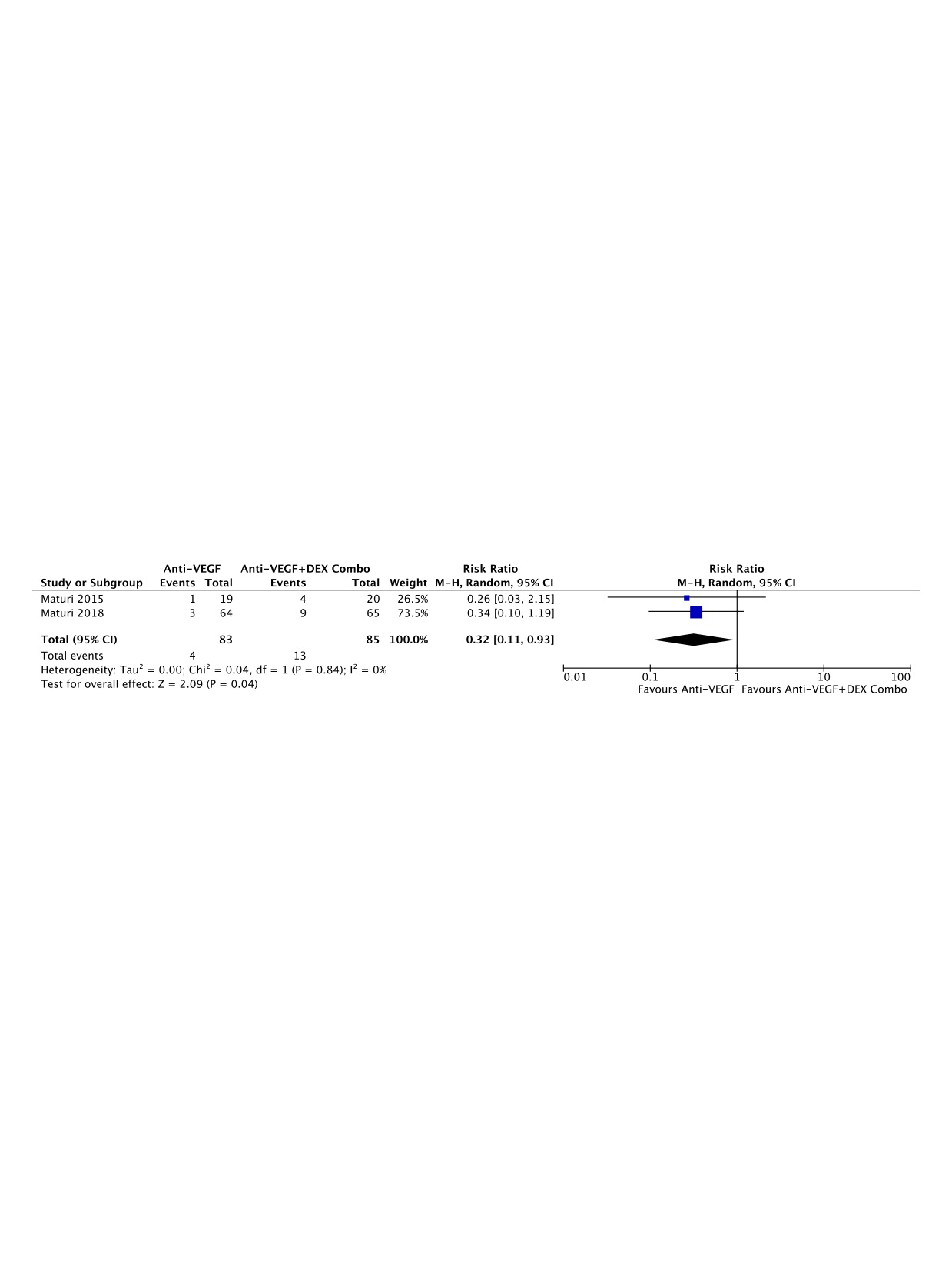

Supplement: sj-zip-1-vrd-10.1177_24741264241280597 – Supplemental material for Anti-VEGF Monotherapy vs Anti-VEGF and Steroid Combination Therapy for Diabetic Macular Edema: A Meta-analysis [file sj-zip-1-vrd-10.1177_24741264241280597.zip › Supplemental Figure 5. l.jpg]

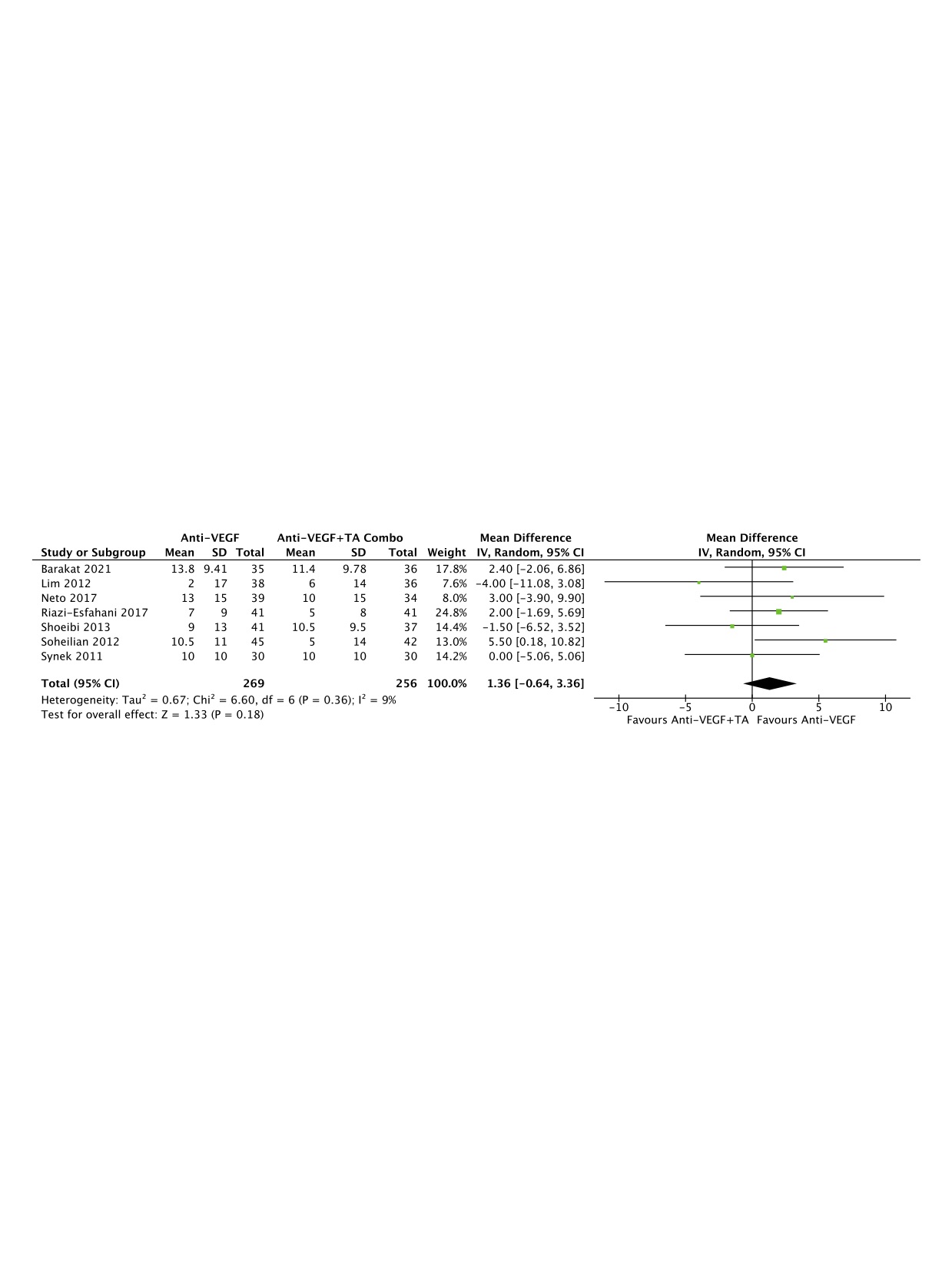

Supplement: sj-zip-1-vrd-10.1177_24741264241280597 – Supplemental material for Anti-VEGF Monotherapy vs Anti-VEGF and Steroid Combination Therapy for Diabetic Macular Edema: A Meta-analysis [file sj-zip-1-vrd-10.1177_24741264241280597.zip › Supplemental Figure 6. c.jpg]

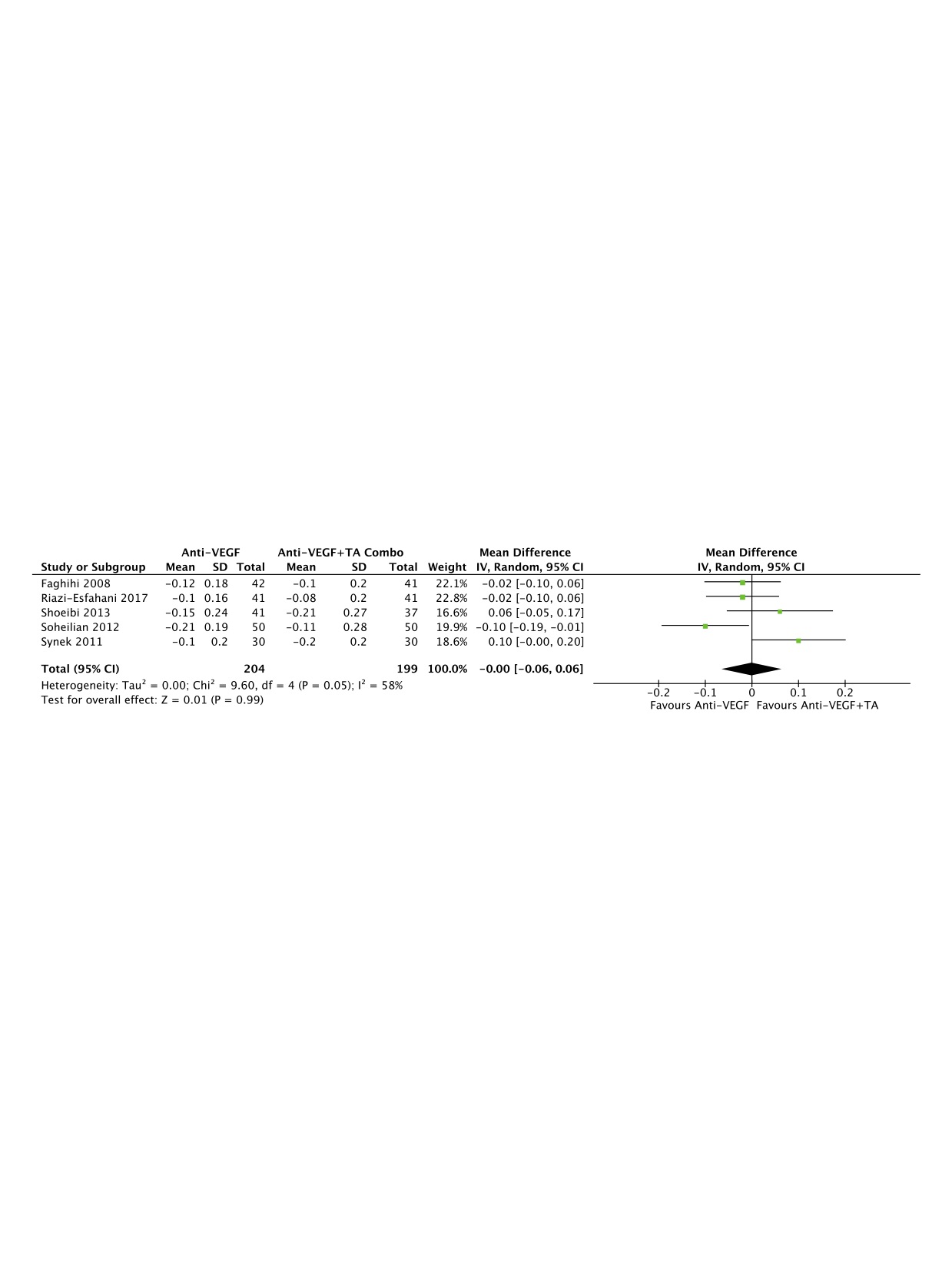

Supplement: sj-zip-1-vrd-10.1177_24741264241280597 – Supplemental material for Anti-VEGF Monotherapy vs Anti-VEGF and Steroid Combination Therapy for Diabetic Macular Edema: A Meta-analysis [file sj-zip-1-vrd-10.1177_24741264241280597.zip › Supplemental Figure 6. e.jpg]

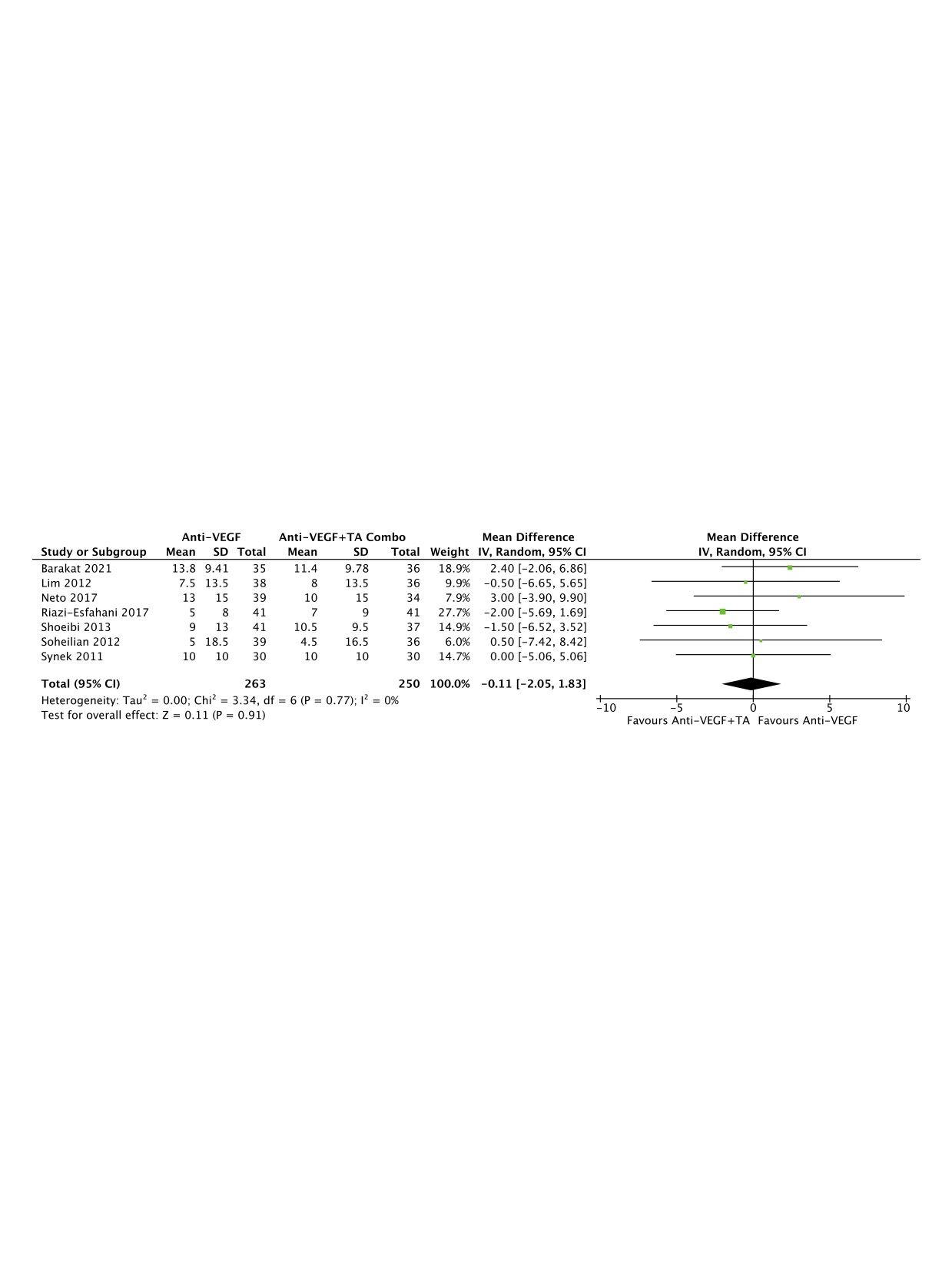

Supplement: sj-zip-1-vrd-10.1177_24741264241280597 – Supplemental material for Anti-VEGF Monotherapy vs Anti-VEGF and Steroid Combination Therapy for Diabetic Macular Edema: A Meta-analysis [file sj-zip-1-vrd-10.1177_24741264241280597.zip › Supplemental Figure 6. a.jpg]

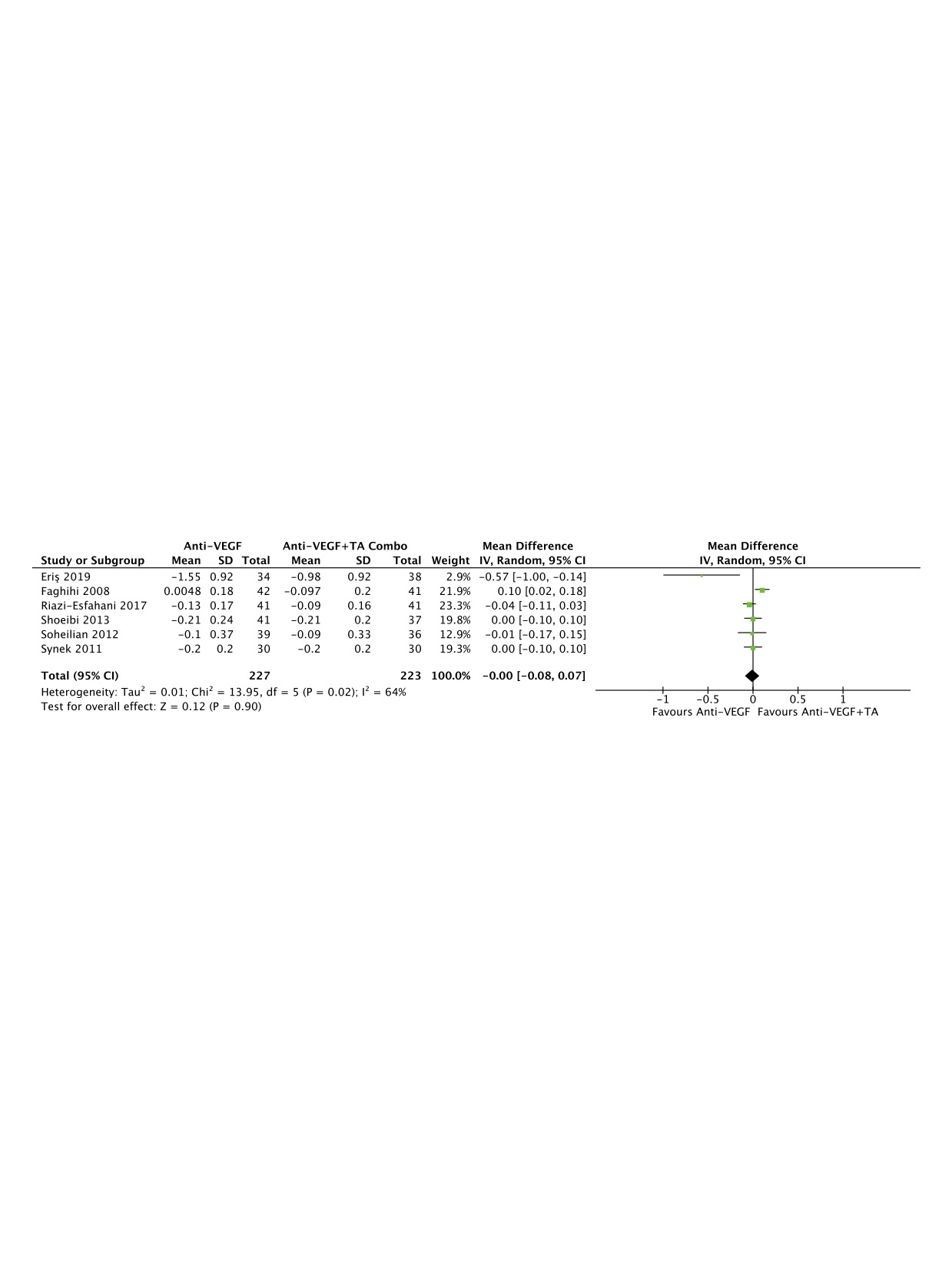

Supplement: sj-zip-1-vrd-10.1177_24741264241280597 – Supplemental material for Anti-VEGF Monotherapy vs Anti-VEGF and Steroid Combination Therapy for Diabetic Macular Edema: A Meta-analysis [file sj-zip-1-vrd-10.1177_24741264241280597.zip › Supplemental Figure 6. b.jpg]

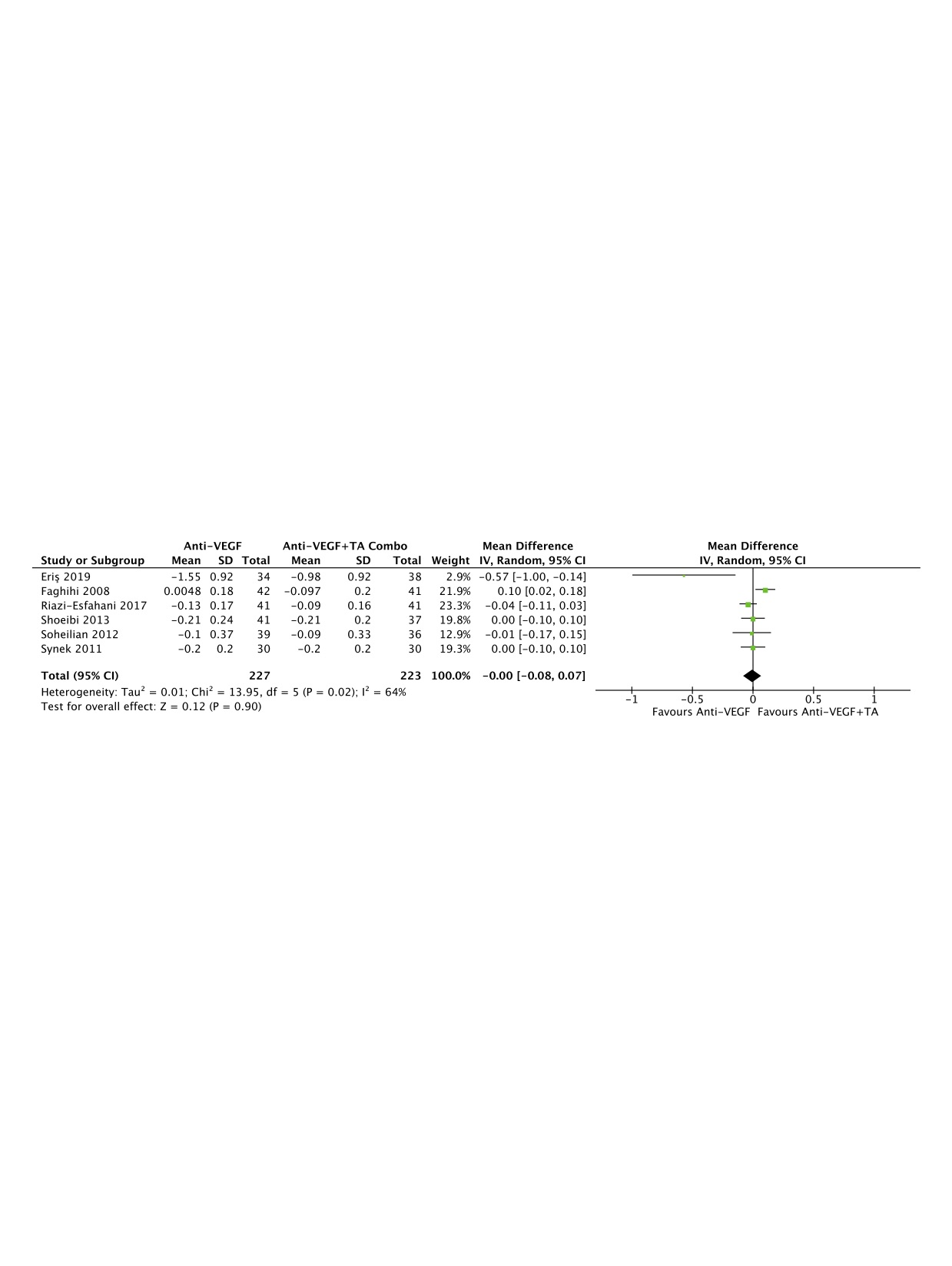

Supplement: sj-zip-1-vrd-10.1177_24741264241280597 – Supplemental material for Anti-VEGF Monotherapy vs Anti-VEGF and Steroid Combination Therapy for Diabetic Macular Edema: A Meta-analysis [file sj-zip-1-vrd-10.1177_24741264241280597.zip › Supplemental Figure 6. d.jpg]

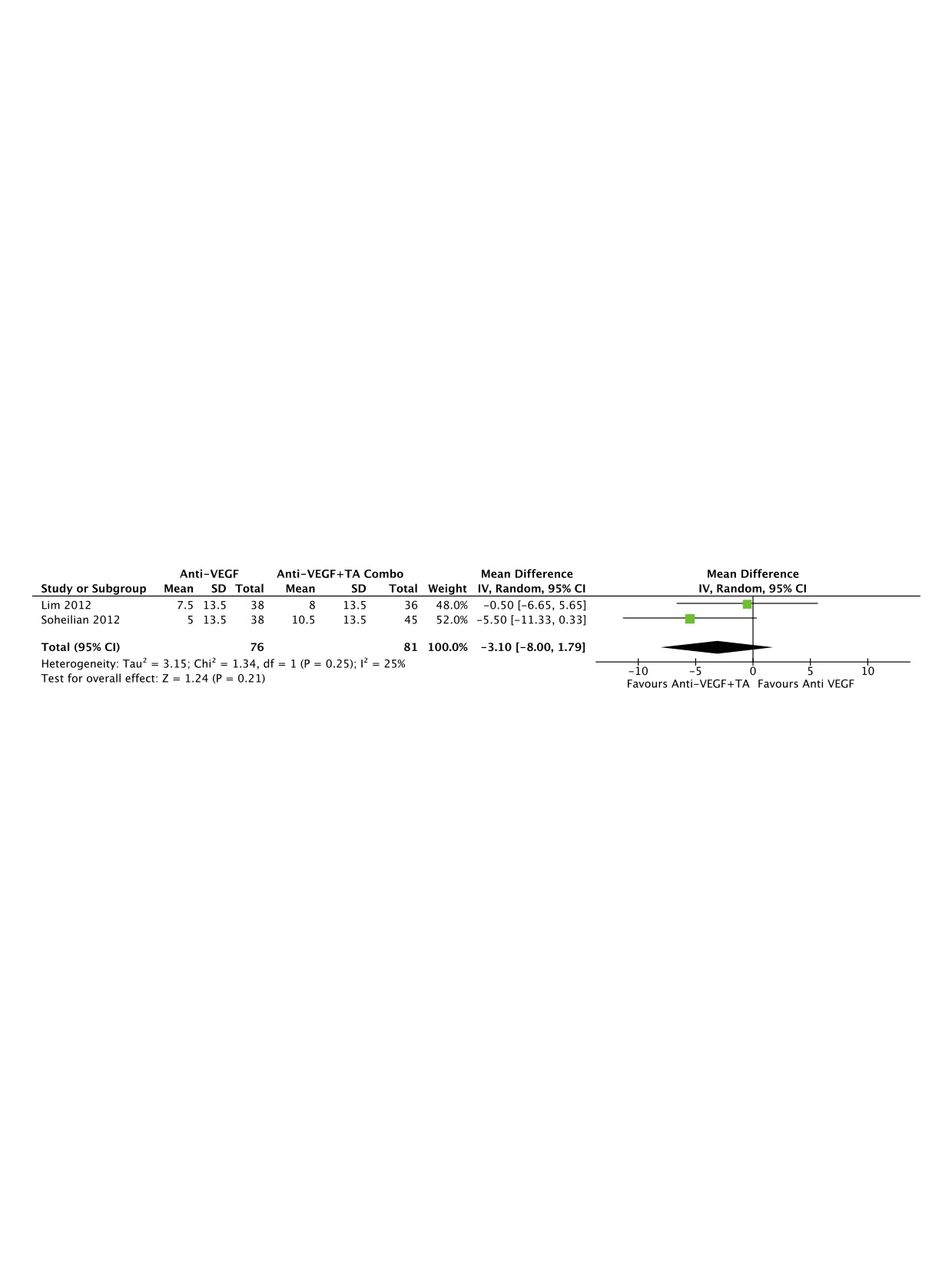

Supplement: sj-zip-1-vrd-10.1177_24741264241280597 – Supplemental material for Anti-VEGF Monotherapy vs Anti-VEGF and Steroid Combination Therapy for Diabetic Macular Edema: A Meta-analysis [file sj-zip-1-vrd-10.1177_24741264241280597.zip › Supplemental Figure 6. f.jpg]

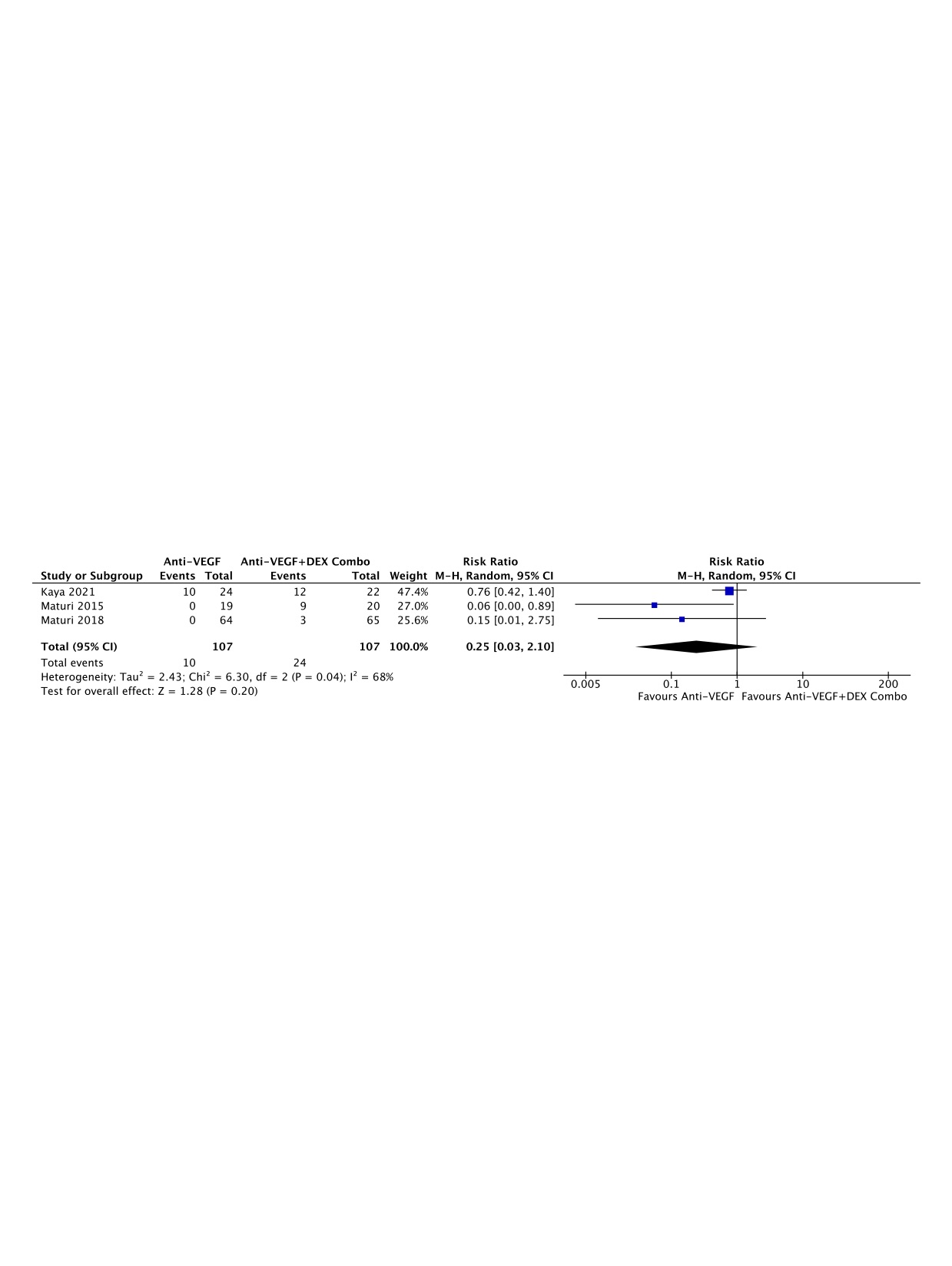

Supplement: sj-zip-1-vrd-10.1177_24741264241280597 – Supplemental material for Anti-VEGF Monotherapy vs Anti-VEGF and Steroid Combination Therapy for Diabetic Macular Edema: A Meta-analysis [file sj-zip-1-vrd-10.1177_24741264241280597.zip › Supplemental Figure 5. k.jpg]

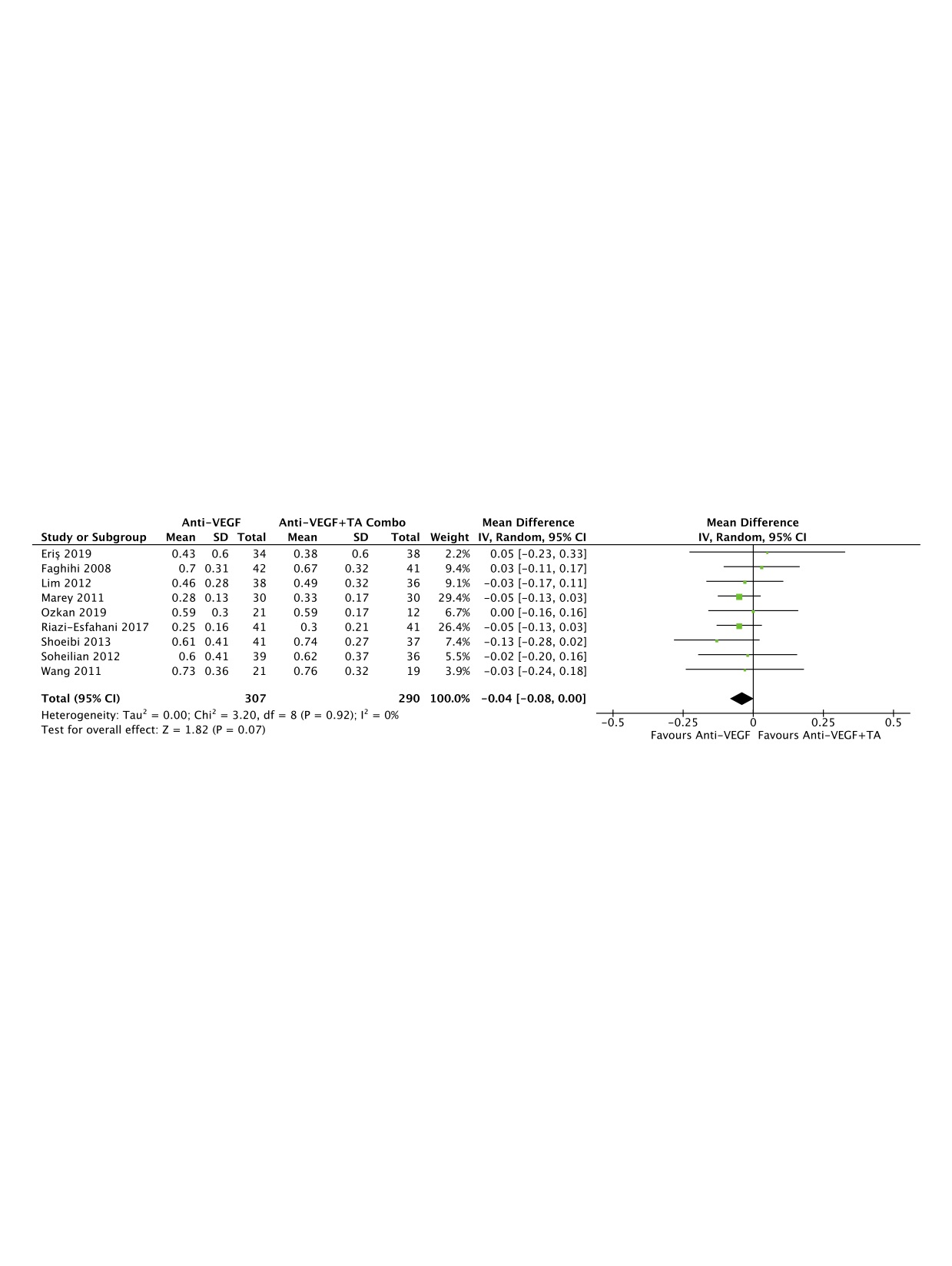

Supplement: sj-zip-1-vrd-10.1177_24741264241280597 – Supplemental material for Anti-VEGF Monotherapy vs Anti-VEGF and Steroid Combination Therapy for Diabetic Macular Edema: A Meta-analysis [file sj-zip-1-vrd-10.1177_24741264241280597.zip › Supplemental Figure 6. g.jpg]

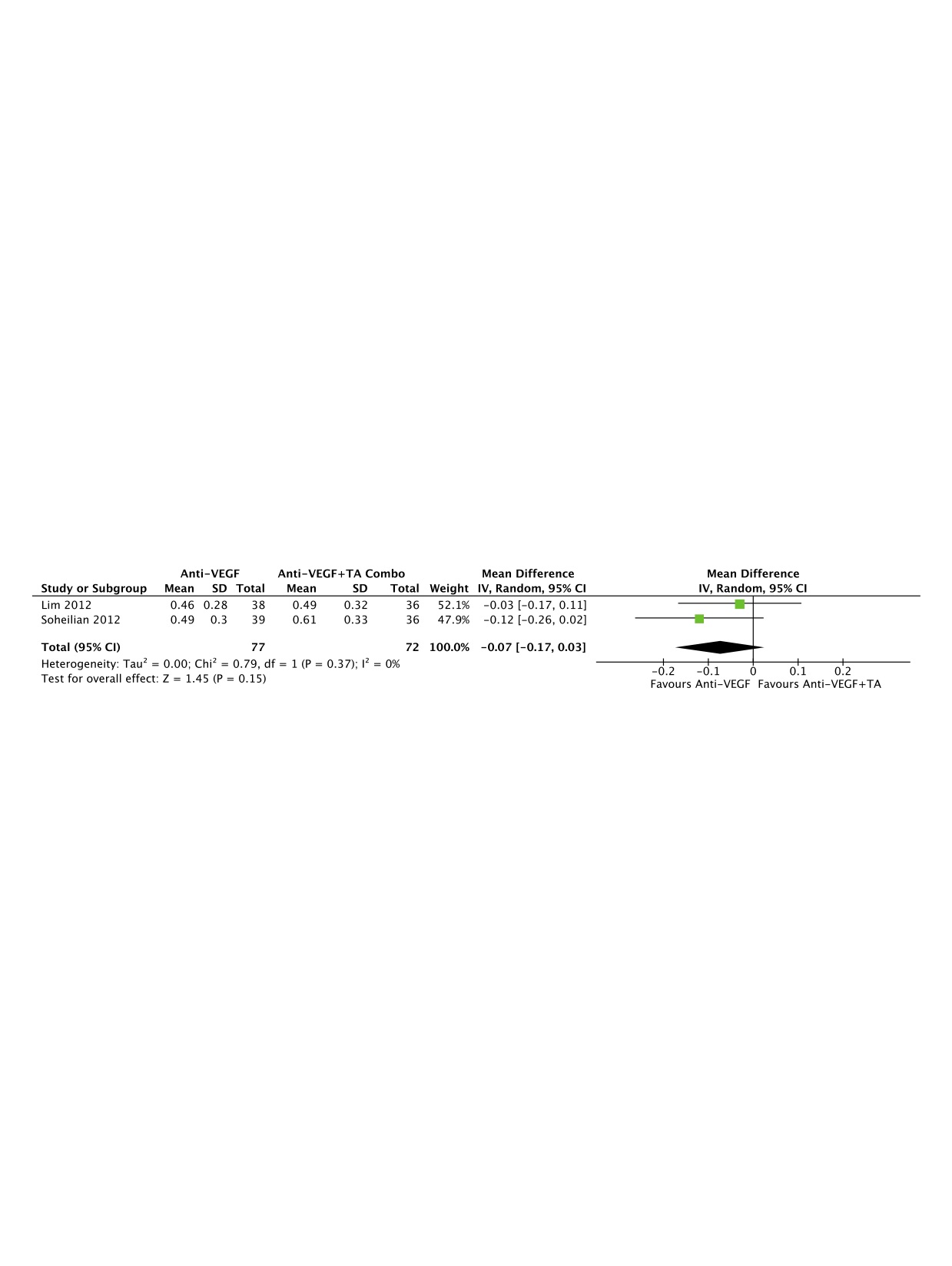

Supplement: sj-zip-1-vrd-10.1177_24741264241280597 – Supplemental material for Anti-VEGF Monotherapy vs Anti-VEGF and Steroid Combination Therapy for Diabetic Macular Edema: A Meta-analysis [file sj-zip-1-vrd-10.1177_24741264241280597.zip › Supplemental Figure 6. h.jpg]

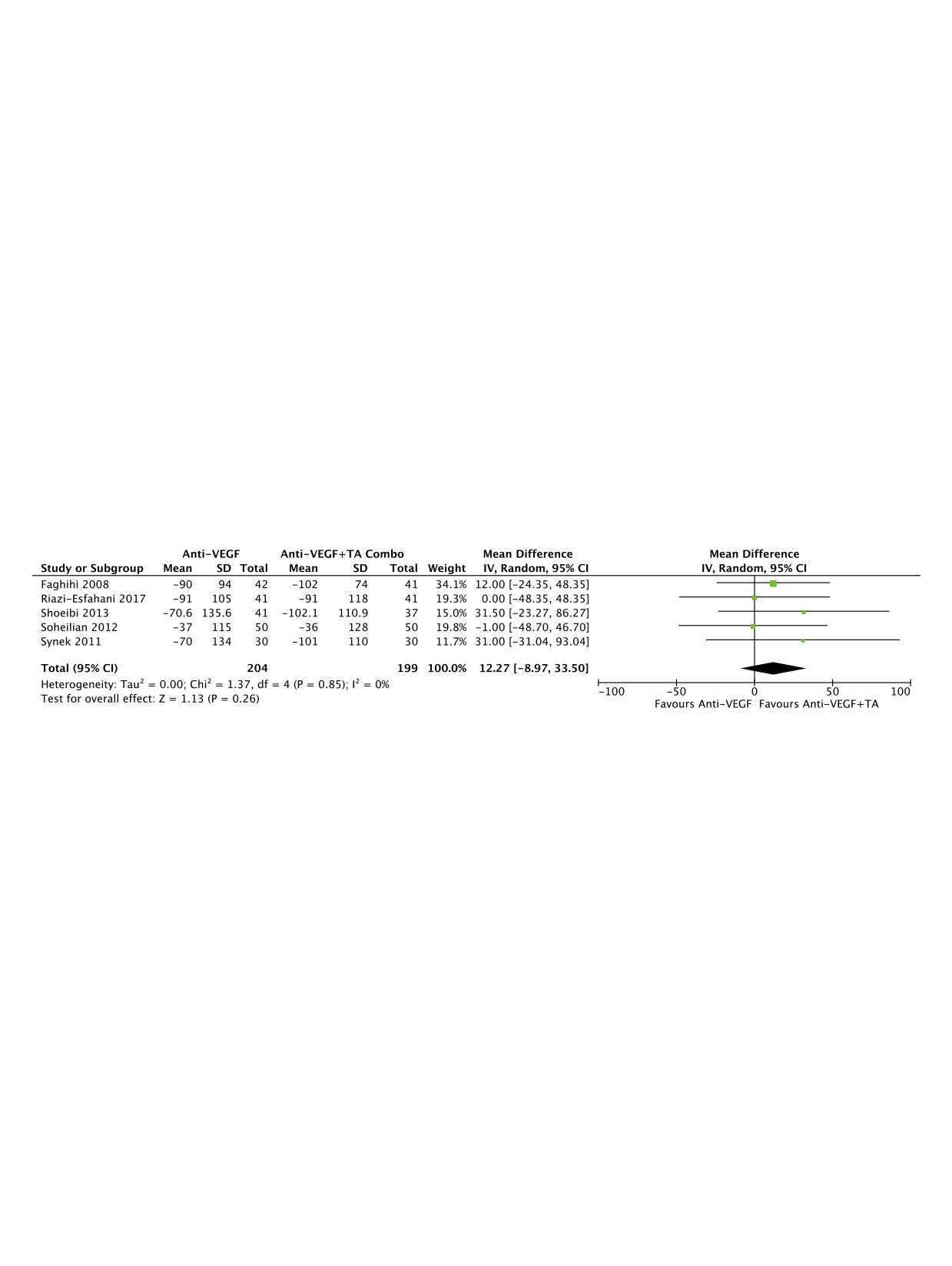

Supplement: sj-zip-1-vrd-10.1177_24741264241280597 – Supplemental material for Anti-VEGF Monotherapy vs Anti-VEGF and Steroid Combination Therapy for Diabetic Macular Edema: A Meta-analysis [file sj-zip-1-vrd-10.1177_24741264241280597.zip › Supplemental Figure 6. m.jpg]

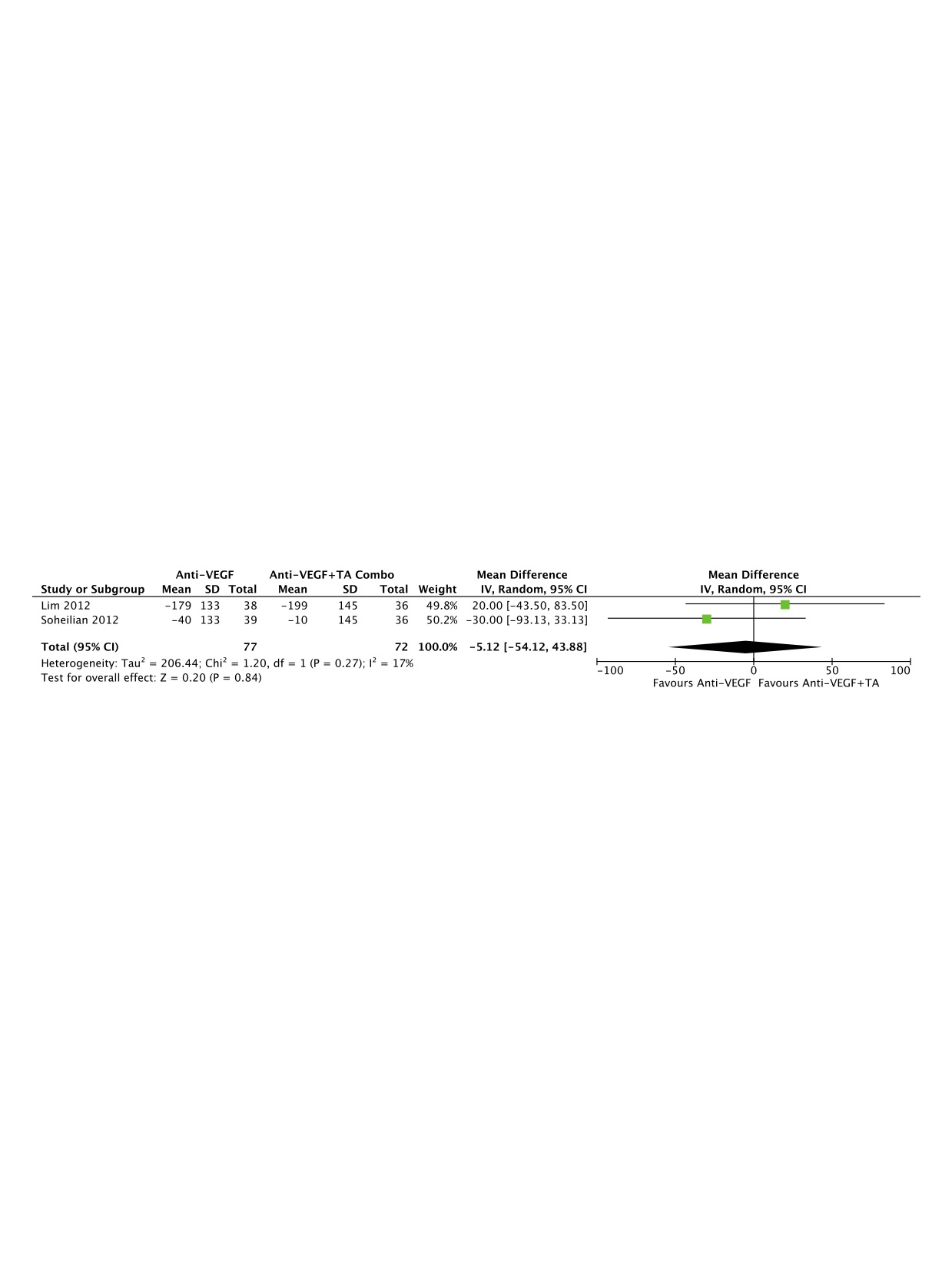

Supplement: sj-zip-1-vrd-10.1177_24741264241280597 – Supplemental material for Anti-VEGF Monotherapy vs Anti-VEGF and Steroid Combination Therapy for Diabetic Macular Edema: A Meta-analysis [file sj-zip-1-vrd-10.1177_24741264241280597.zip › Supplemental Figure 6. n.jpg]

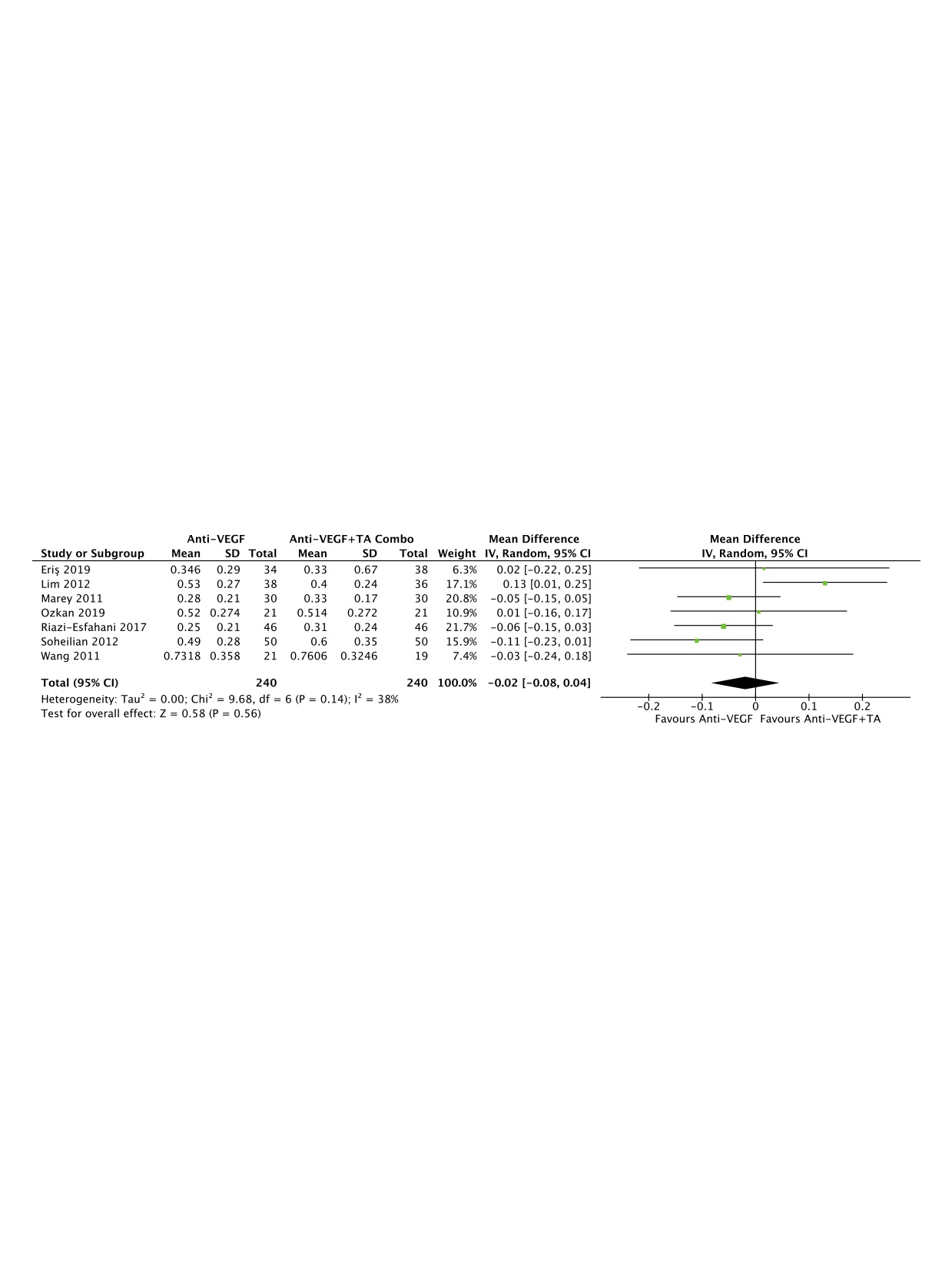

Supplement: sj-zip-1-vrd-10.1177_24741264241280597 – Supplemental material for Anti-VEGF Monotherapy vs Anti-VEGF and Steroid Combination Therapy for Diabetic Macular Edema: A Meta-analysis [file sj-zip-1-vrd-10.1177_24741264241280597.zip › Supplemental Figure 6. i.jpg]

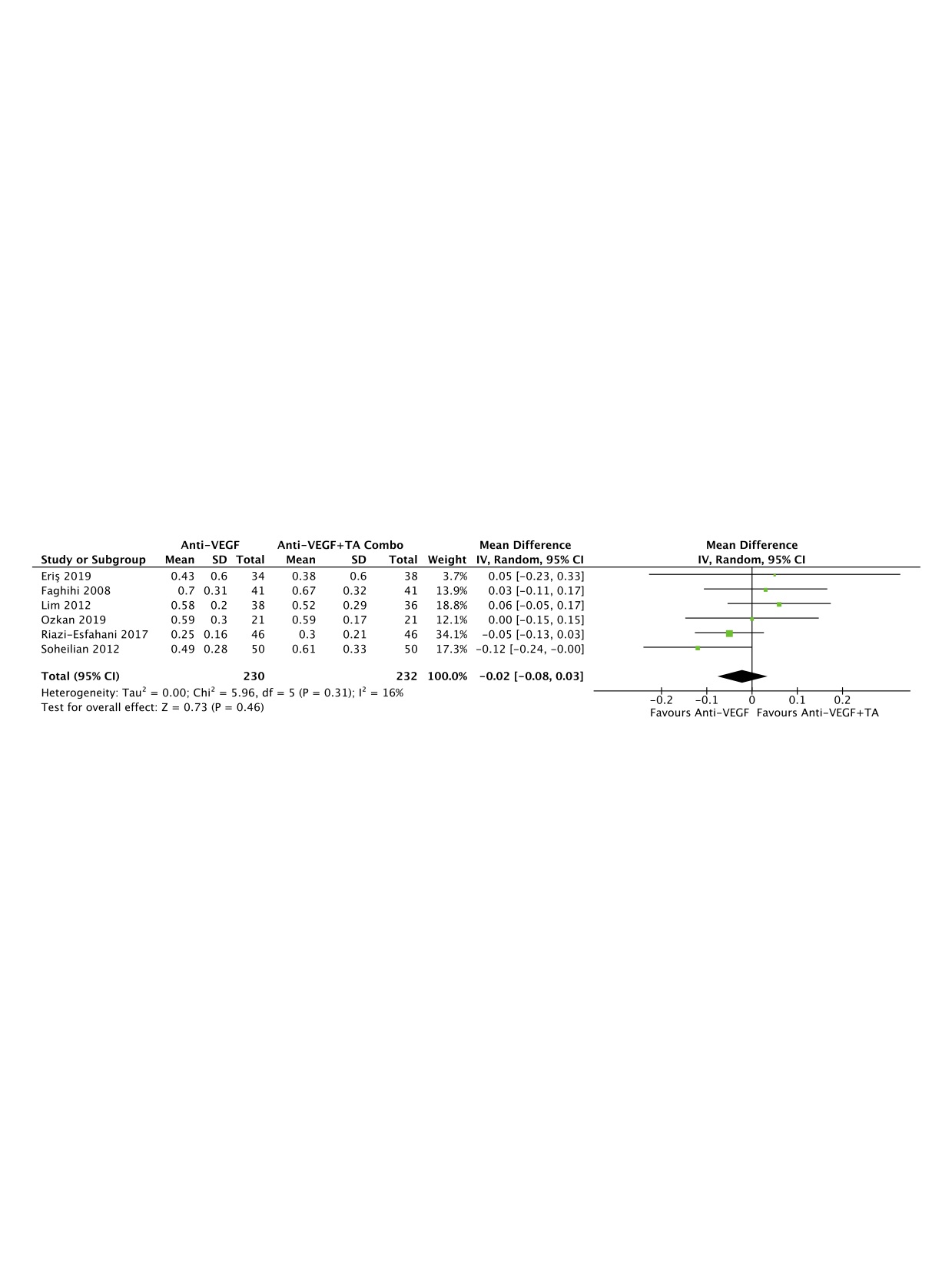

Supplement: sj-zip-1-vrd-10.1177_24741264241280597 – Supplemental material for Anti-VEGF Monotherapy vs Anti-VEGF and Steroid Combination Therapy for Diabetic Macular Edema: A Meta-analysis [file sj-zip-1-vrd-10.1177_24741264241280597.zip › Supplemental Figure 6. j.jpg]

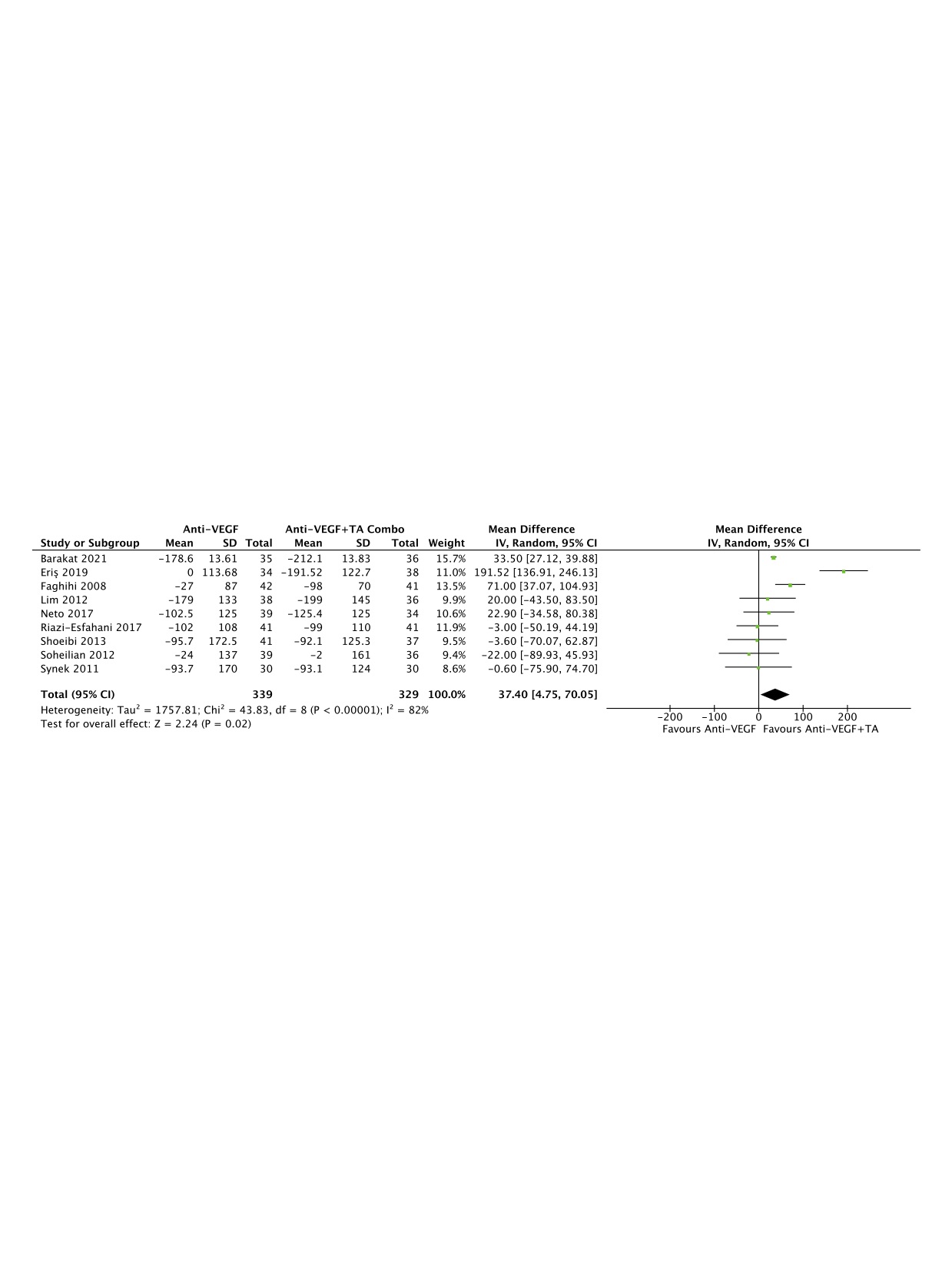

Supplement: sj-zip-1-vrd-10.1177_24741264241280597 – Supplemental material for Anti-VEGF Monotherapy vs Anti-VEGF and Steroid Combination Therapy for Diabetic Macular Edema: A Meta-analysis [file sj-zip-1-vrd-10.1177_24741264241280597.zip › Supplemental Figure 6. k.jpg]

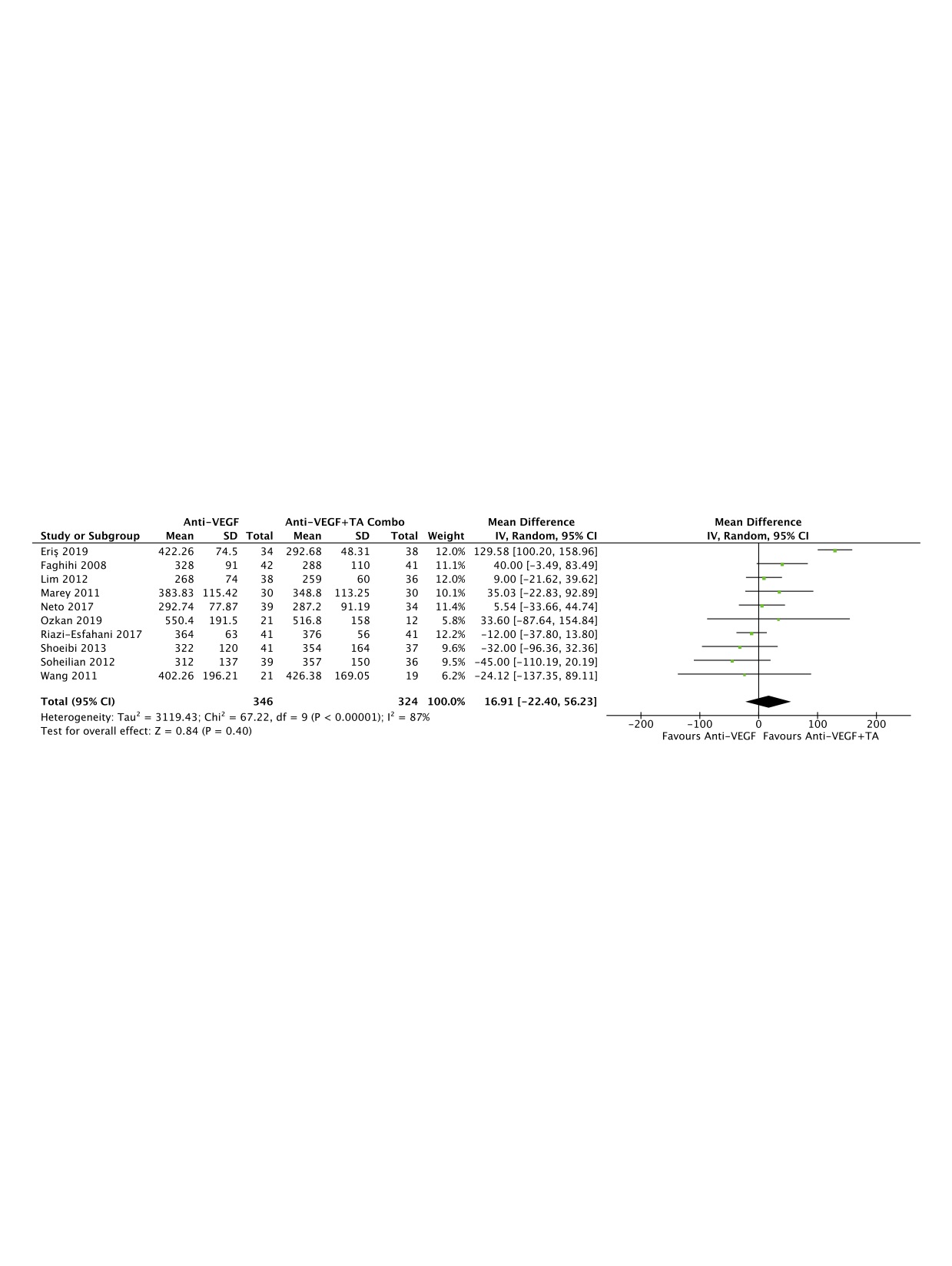

Supplement: sj-zip-1-vrd-10.1177_24741264241280597 – Supplemental material for Anti-VEGF Monotherapy vs Anti-VEGF and Steroid Combination Therapy for Diabetic Macular Edema: A Meta-analysis [file sj-zip-1-vrd-10.1177_24741264241280597.zip › Supplemental Figure 6. o.jpg]

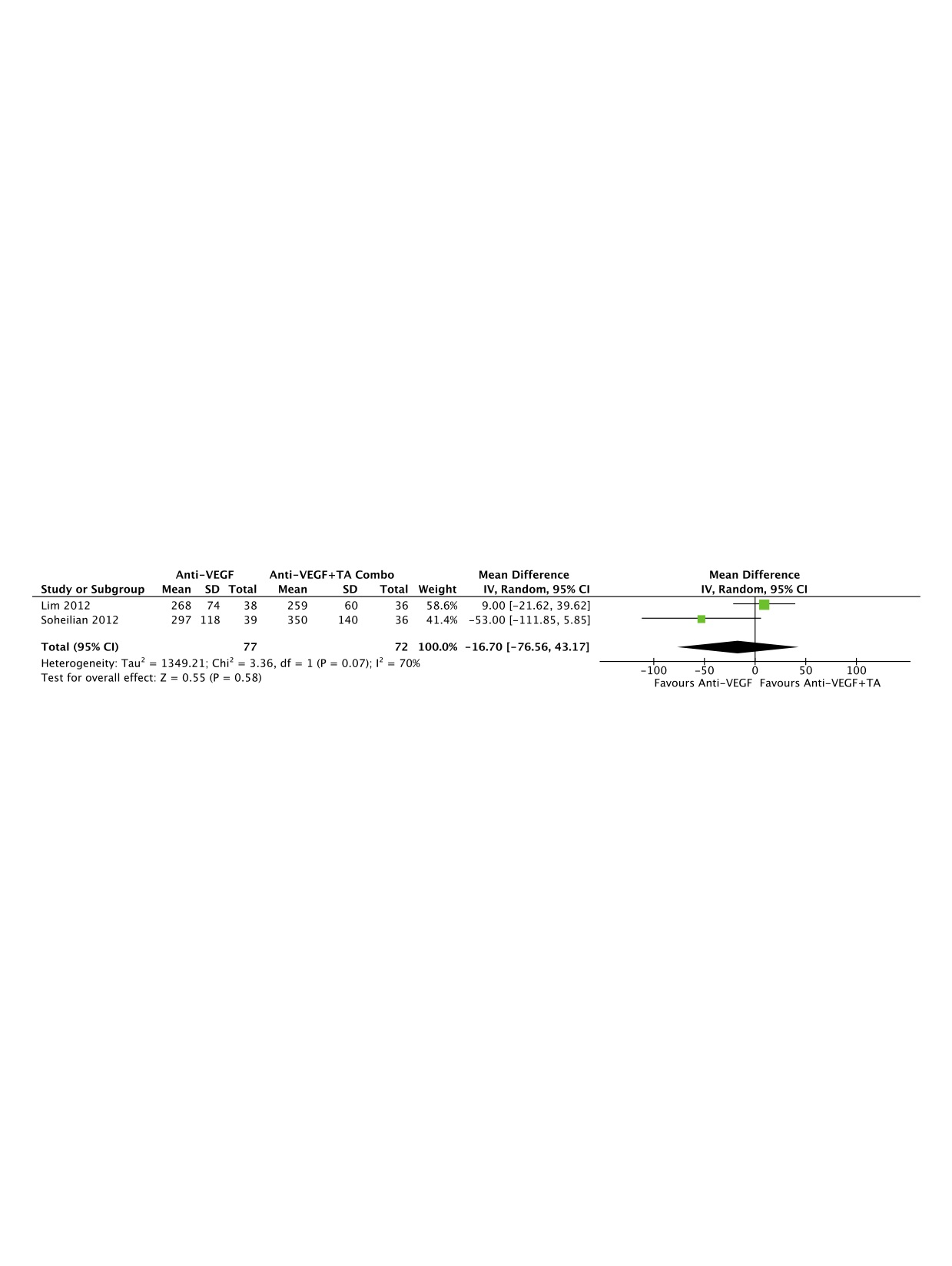

Supplement: sj-zip-1-vrd-10.1177_24741264241280597 – Supplemental material for Anti-VEGF Monotherapy vs Anti-VEGF and Steroid Combination Therapy for Diabetic Macular Edema: A Meta-analysis [file sj-zip-1-vrd-10.1177_24741264241280597.zip › Supplemental Figure 6. q.jpg]

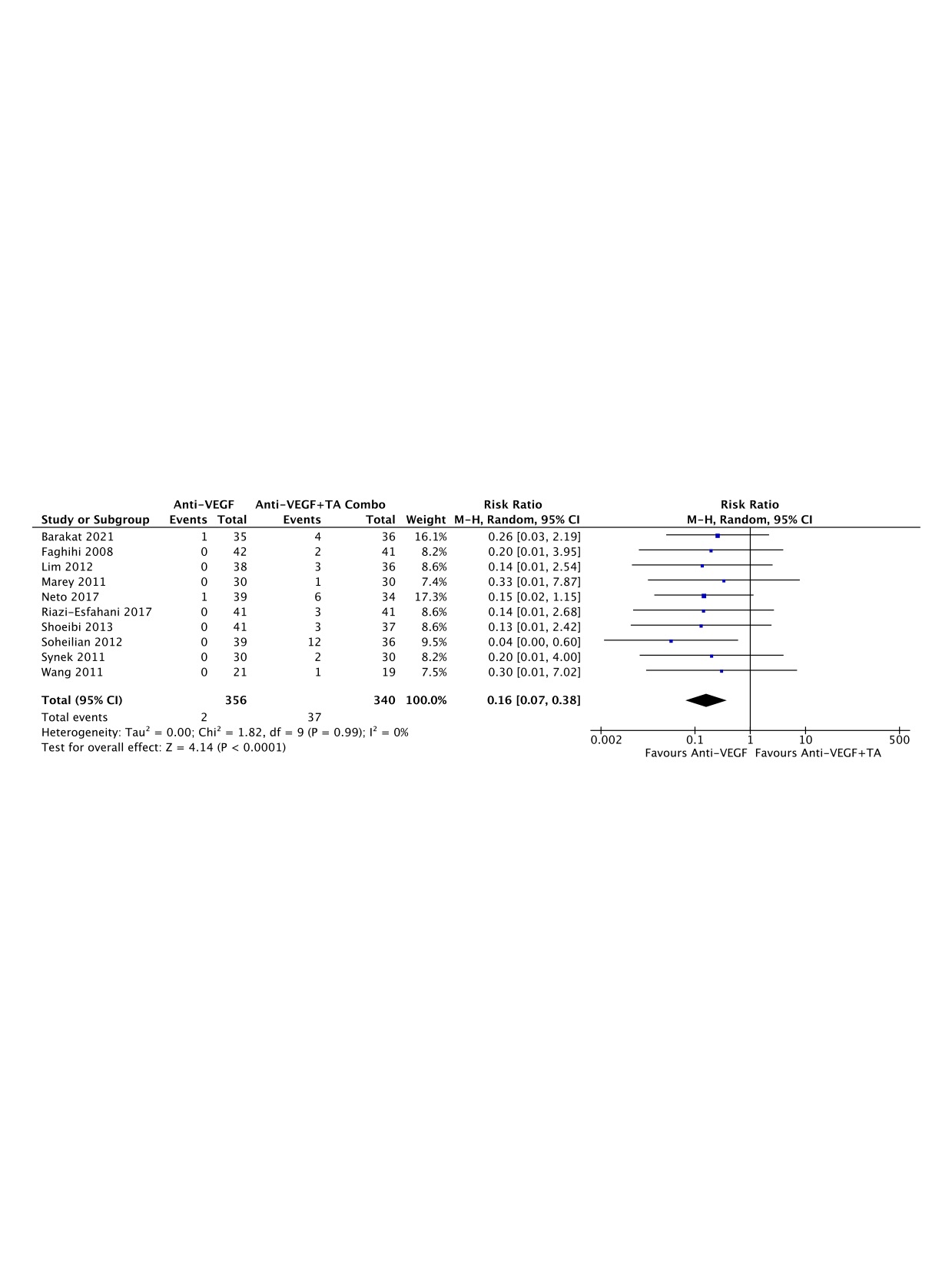

Supplement: sj-zip-1-vrd-10.1177_24741264241280597 – Supplemental material for Anti-VEGF Monotherapy vs Anti-VEGF and Steroid Combination Therapy for Diabetic Macular Edema: A Meta-analysis [file sj-zip-1-vrd-10.1177_24741264241280597.zip › Supplemental Figure 6. s.jpg]

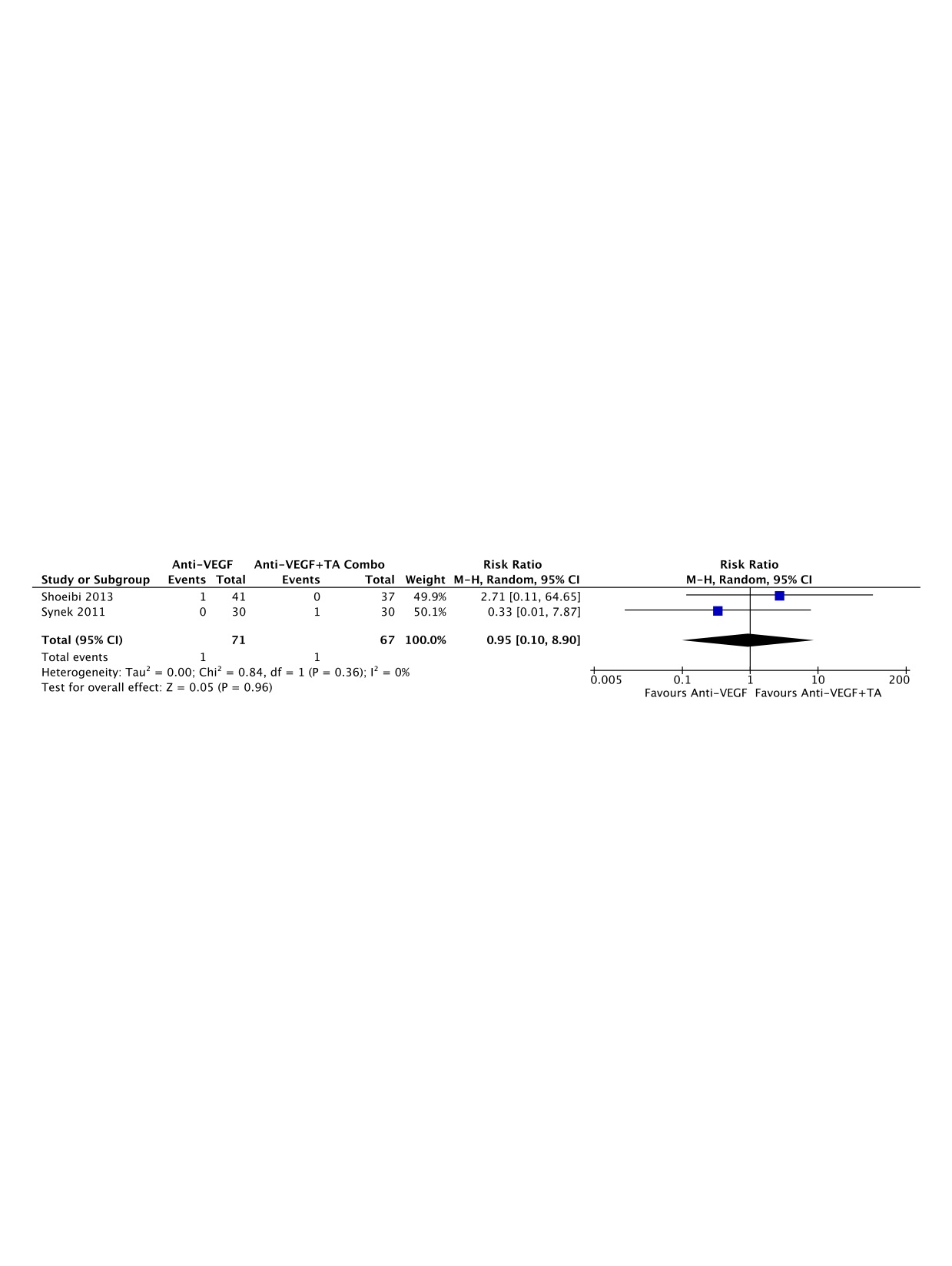

Supplement: sj-zip-1-vrd-10.1177_24741264241280597 – Supplemental material for Anti-VEGF Monotherapy vs Anti-VEGF and Steroid Combination Therapy for Diabetic Macular Edema: A Meta-analysis [file sj-zip-1-vrd-10.1177_24741264241280597.zip › Supplemental Figure 6. t.jpg]
